# The Effect of Protein Intake on Bone Disease, Kidney Disease, and Sarcopenia: A Systematic Review

**DOI:** 10.1016/j.cdnut.2025.104546

**Published:** 2025-01-21

**Authors:** Toyin Lamina, Sallee Brandt, Hamdi I Abdi, Hawking Yam, Ashenafi G Hayi, Romil Parikh, Chelsey Kirkland, Amy M Claussen, Kendal M Burstad, Joanne L Slavin, Levi Teigen, Lyn M Steffen, Kathleen M Hill Gallant, Tasma Harindhanavudhi, Anne Kouri, Sue Duval, Jamie Stang, Mary Butler

**Affiliations:** 1 Minnesota Evidence-Based Practice Center, School of Public Health, University of Minnesota, Minneapolis, MN, United States; 2 Division of Health Policy and Management, School of Public Health, University of Minnesota, Minneapolis, MN, United States; 3 Center for Public Health Systems, School of Public Health, University of Minnesota, Minneapolis, MN, United States; 4 Department of Food Science and Nutrition, University of Minnesota, Saint Paul, MN, United States; 5 Division of Epidemiology and Community Health, School of Public Health, University of Minnesota, Minneapolis, MN, United States; 6 Division of Diabetes, Endocrinology, and Metabolism, Medical School, University of Minnesota, Minneapolis, MN, United States; 7 Division of Pediatric Nephrology, Medical School, University of Minnesota, Minneapolis, MN, United States; 8 Cardiovascular Division, Medical School, University of Minnesota, Minneapolis, MN, United States

**Keywords:** dietary reference intakes, health, muscle, nutrition, renal, risk factors

## Abstract

**Background:**

Protein is essential for optimal growth, function, and maintenance of health. Its impact on bone, kidney health, and sarcopenia progression remains debated.

**Objectives:**

This review examines the association between dietary protein intake and the risk of bone disease, kidney disease, and sarcopenia to inform protein dietary reference intake updates.

**Methods:**

We searched Medline, EMBASE, AGRICOLA, and Scopus from January 2000 to May 2024, supplemented by citation searching for relevant reviews and original research. We included randomized and nonrandomized controlled trials, prospective cohort studies, and nested case-control studies examining dietary protein intake without exercise. We assessed the risk of bias (RoB), performed a qualitative synthesis of low to moderate RoB studies, and evaluated the strength of evidence.

**Results:**

Of 82 articles detailing 81 unique studies, only 13 were assessed with low to moderate RoB and synthesized, comprising bone disease [4 randomized controlled trials (RCTs) and 1 prospective cohort study], kidney disease (1 RCT), and sarcopenia (9 RCTs). The overarching evidence was insufficient, largely due to the limited number of low to moderate RoB studies, the diversity of dietary protein interventions, and the broad range of outcomes, which complicated synthesis and comparison. Notably, sparse literature addressed children and adolescents, and only a single study each examined the impact of dietary protein intake on bone disease risk (yielding mixed findings) in these populations and on kidney disease risk (showing no significant effects) in adults. The findings on the impact of protein intake on bone disease in adults and sarcopenia risk were mixed; some studies showed no effect, whereas others indicated benefits.

**Conclusions:**

The evidence since 2000 on associations between dietary protein intake and the risks of bone disease, kidney disease, and sarcopenia is unclear, indicating a need for more rigorous research.

This trial was registered at PROSPERO as CRD42023446621.

## Introduction

Protein is crucial for optimal growth, development, function, and the maintenance of human health [1]. Certain chronic conditions linked to dietary protein intake, such as bone disease, kidney disease, and sarcopenia, have been studied extensively. Protein is vital for maintaining bone health at all life stages. During childhood and adolescence, adequate dietary protein intake supports robust growth and development, helps achieve peak bone mass, and lays a strong foundation for future bone health [2,3]. In adults, the impact of dietary protein on bone health is more complex, with studies showing both positive and negative effects, suggesting a nuanced relationship [[4], [5], [6], [7]].

Similar concerns exist around dietary protein and kidney health, with unanswered questions about whether protein can negatively affect kidney health in the general population [[8], [9], [10], [11], [12]]. Sarcopenia is an age-related condition marked by the progressive loss of muscle strength, muscle mass, and/or physical performance [13]. Although it can occur earlier in life, sarcopenia is most common among older adults. Its progression is linked to malnutrition, frailty, disability, reduced cardiopulmonary function, metabolic syndrome, insulin resistance, cognitive impairment, falls, fractures, depressive symptoms, hospitalization, and death [14,15]. Dietary protein might help slow the progression of sarcopenia [16,17].

Dietary reference intakes (DRIs) are scientifically developed reference values for nutrients, expanding on the periodic recommended dietary allowances (RDAs), which have been published since 1941 by the National Academy of Sciences [18]. Jointly developed by the United States and Canada since the mid-1990s, DRIs include values such as the RDA, estimated average requirement (EAR), adequate intake, tolerable upper intake level, acceptable macronutrient distribution range (AMDR), and chronic disease risk reduction intake (CDRR) [18]. DRIs are intended for the general healthy population and are used by nutrition experts, governments, nongovernmental organizations, and academic institutions for various activities, including developing dietary guidelines, food guides, nutrition labels, dietary counseling, and educational materials.

DRIs for protein were first published in 2005, setting intake recommendations for apparently healthy adults (19 y and older) at 0.66 and 0.8 g/kg/d for the EAR and RDA, respectively[18], and 10–35% of energy intake for the AMDR [18]. Generally, for protein, a higher EAR and RDA are required during vital periods of growth and development such as infancy, childhood, and adolescence (EAR: 0.71–1.0 g/kg/d, RDA: 0.85–1.2 g/kg/d) [18]. The AMDR for protein is 5–20% of energy intake for children 1–3 y of age and 10–30% of total calories for children 4–18 y [18]. No tolerable upper intake level for protein was established due to insufficient data. Current DRIs for protein lack a reference value for CDRR, which was developed after the establishment of the most recent protein DRI values.

Efforts to update DRIs aim to incorporate evidence on chronic diseases and establish a new category of values specific to CDRR [19]. Since the last protein DRIs, new research has emerged on the relationship between dietary protein intake and chronic disease risk. This review aims to evaluate the association between dietary protein intake and the risk of bone disease, kidney disease, and sarcopenia to inform updates to the protein DRIs.

## Methods

This review was conducted in accordance with the Agency for Healthcare Research and Quality Methods Guide for Effectiveness and Comparative Effectiveness Reviews [20] and the PRISMA guideline [21]. The protocol of this review is registered at the PROSPERO website (CRD42023446621) and can be found at (https://effectivehealthcare.ahrq.gov/products/effect-protein-intake/protocol). The full report can be found at (https://effectivehealthcare.ahrq.gov/products/effect-protein-intake/draft-report).

### Data sources and searches

We searched Medline, EMBASE, AGRICOLA, and Scopus databases from January 2000 through May 2024 (Supplemental Methods) to capture all relevant published literature since the current protein DRIs were established in 2005. We supplemented our bibliographic database searches with citation searching of relevant systematic reviews and original research. Search strategies were peer-reviewed by a reference librarian who was not a team member.

### Study selection

Eligible studies included randomized and nonrandomized trials, prospective cohorts, and nested case-control studies involving apparently healthy individuals—those without acute or chronic diseases affecting nutrient metabolism or requirements and free from significant health conditions that could alter normal physiological needs. The studies examined the impact of dietary protein intake on bone disease, kidney disease, and sarcopenia without an exercise intervention (i.e., a structured program or protocol designed to assess the effects of physical activity on health outcomes). This approach ensured that the findings were specific to the effects of dietary protein intake and not confounded by other co-interventions such as exercise. For bone disease, studies involving infants, children, and adolescents (0–18 y) as well as adults (18+ y) were included. For kidney disease and sarcopenia, only studies involving adults (18+ y) were included. Studies were selected from countries where food products or dietary supplements are widely available to United States consumers and rated high or very high on the Human Development Index [22]. This approach ensured greater generalizability to the United States and Canada although excluding studies conducted in countries classified as medium or low on the human development index. Detailed eligibility criteria are provided in Supplemental Table 1. Search results were downloaded to EndNote X9 and screened using PICO Portal software [23]. Two independent investigators screened titles and abstracts. After training the machine learning system, 1 investigator screened once a 90% recall rate was achieved for citations eligible for full-text screening, stopping at a 95% recall rate. Full-text screening was conducted by 2 independent investigators, with differences resolved through consultation or, if necessary, a third reviewer.

### Data extraction

The systematic review data repository (SRDR) online system [24] was used for study-level data extraction. One reviewer extracted data, and a second senior systematic reviewer conducted quality checks on 20% of the studies. Data from eligible studies were extracted into evidence tables presented in Supplemental Tables 2–7. Outcomes details were extracted from only studies rated as low to moderate risk of bias (RoB) (i.e., studies less prone to biases affecting the robustness of their findings - the analytic set).

### RoB assessment

We assessed the methodological RoB using the Cochrane RoB tool 2.0 for randomized controlled trials (RCTs) [25,26] and the RoB in nonrandomized studies of exposure tool for observational studies [27] detailed in Supplemental Methods. Each study was independently assessed by 1 reviewer, with a second investigator reviewing each assessment. Discrepancies were resolved through consultation. The overall RoB for each study outcome was classified as low, moderate, or high for RCTs and low, moderate, high, or very high for observational studies.

### Data analysis

Findings were synthesized from studies rated as low to moderate RoB (i.e., the analytic set) and were organized by population, study design, outcomes, and comparisons. Due to heterogeneity, including varied outcome measures and dietary protein intake interventions, and sparse outcome data distribution across studies, we were unable to collate and compare findings quantitatively in a meta-analysis. Thus, a qualitative synthesis was provided. Outcomes were grouped into broad categories for better summarization. For bone disease, outcomes included bone turnover markers, axial and appendicular skeleton bone mineral density (BMD) and bone mineral content (BMC), total body BMD and BMC, osteoporotic fractures, and fracture risk, fracture at specific sites, and bone geometry and strength indices. Outcomes for kidney disease included kidney function, kidney stones, electrolytes, proteinuria, and hyperfiltration. For sarcopenia, outcomes were categorized as muscle mass, physical performance, and muscle strength. For each comparison, we presented a summary of findings tables for the outcomes in the Results section.

### Grading the strength of evidence

Strength of evidence (SoE) is the extent of our confidence in drawing a specific conclusion and is based on causal inference criteria. The overall SoE was rated as insufficient, low, moderate, or high for identified outcomes and was evaluated based on 5 required domains: *1*) study limitations (RoB); *2*) consistency (similarity of effect direction and size); *3*) directness (single, direct link between intervention and outcome); *4*) precision (degree of certainty around an estimate); and *5*) reporting bias [28], detailed in the Supplemental Methods.

## Results

**FIGURE 1:**
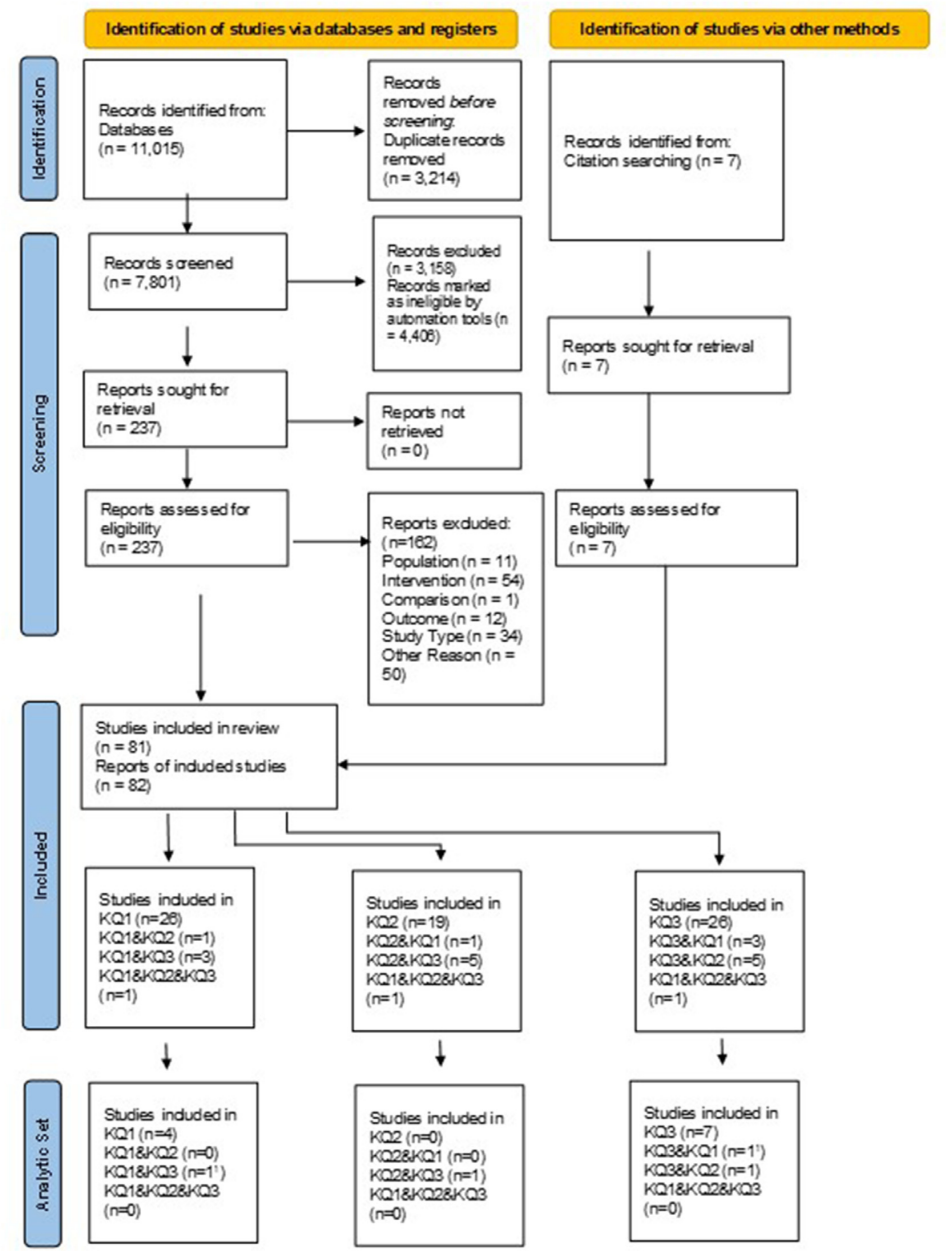
Search and screening process from the identification to the inclusion of studies, based on the PRISMA process [110]. KQ1–KQ3; PRISMA, preferred reporting items for systematic reviews and meta-analyses. ^1^One study reported data on KQ1, KQ2, and KQ3, but only the KQ1 and KQ3 outcomes had a low risk of bias and were included in the analytic set. Abbreviations: KQ1; Key Question 1; KQ2, Key Question 2; KQ3, Key Question 3.

Our literature search identified 11,015 studies for review, and 82 articles detailing 81 distinct studies met our inclusion criteria [15,[29], [30], [31], [32], [33], [34], [35], [36], [37], [38], [39], [40], [41], [42], [43], [44], [45], [46], [47], [48], [49], [50], [51], [52], [53], [54], [55], [56], [57], [58], [59], [60], [61], [62], [63], [64], [65], [66], [67], [68], [69], [70], [71], [72], [73], [74], [75], [76], [77], [78], [79], [80], [81], [82], [83], [84], [85], [86], [87], [88], [89], [90], [91], [92], [93], [94], [95], [96], [97], [98], [99], [100], [101], [102], [103], [104], [105], [106], [107], [108], [109], [110]] (Figure 1); 13 of these were rated as low to moderate RoB and comprise our analytic set [32,36,45,51,65,75,92,93,100,101,103,107,108]. Characteristics and findings for the analytic set are described below. The overall RoB scores and the scores for single domains are summarized in Supplemental Tables 8–10. For each comparison, we presented the SoE for the outcomes in a SoE table (Supplemental Tables 11–14) and in the summary of findings tables below.

### Bone disease in adults

#### Characteristics and findings of the analyzed studies

The analytic set included bone disease studies [3 RCTs in adults [36,65,100] (2 low and 1 moderate RoB), 1 prospective cohort study in adults [93] (moderate RoB)]. Characteristics and findings of the analytic set are summarized in Table 1 [36,65,93,100]. Studies were conducted in various countries, including France [36], Denmark [100], United States [65], and Mexico [93]. Dietary protein intake interventions and comparators were different across RCTs. Study follow-up ranged from 6 wk to 1.5 y. Different cut-off points (such as high and low) were used to describe protein intake levels. Protein measurement units also differed, including absolute protein intake in grams (g) or protein intake per energy intake (%). For adults, the evidence was insufficient to draw conclusions about any outcome related to the risk of bone disease.

**TABLE 1:** Summary of findings for bone disease outcomes in adults.

Outcome comparisons	Author, (year) (*n* analyzed) study duration	Country Age Sex Obesity status Menopausal status	Protein assessment method	Baseline protein	Outcome baseline	Outcome follow-up	Direction of effect^1^	Strength of the evidence^2^
OC I: treated group (test food – 13.8 g protein) vs. C: usual diet	Bonjour, (2012) [36] 1 RCT (*n* = 71) 6 wk	France Mean age (SD): I: 57.1 (3.9) y C: 56.1 (3.9) y Sex: 100% female NR Postmenopausal	Food frequency questionnaire	I: mean (SD) 72 (17) g/d C: mean (SD) 199 (79) g/d	I: mean (SD): 25.9 (9.7) mg/L C: mean (SD): 26.9 (9.6) mg/L	I: change in osteocalcin: mean (SD): –0.39 (3.6) mg/L C: change in osteocalcin: mean (SD): 0.77 (3.4) mg/L	No difference	Insufficient
CTX I: treated group (test food – 13.8 g protein) vs. C: usual diet	Bonjour, (2012) [36] 1 RCT (*n* = 71) 6 wk	France Mean age (SD): I: 57.1 (3.9) y C: 56.1 (3.9) y Sex: 100% female NR Postmenopausal	Food frequency questionnaire	I: mean (SD) 72 (17) g/d C: mean (SD) 199 (79) g/d	I: mean (SD) 3.56 (1.6) nmol/L C: mean (SD) 3.56 (1.58) nmol/L	I: change in CTX:mean (SD): –0.18 (0.70) nmol/L C: change in CTX: mean (SD): 0.06 (0.85) nmol/L	No difference	Insufficient
TRAP Treated group (test food – 13.8 g protein) vs. usual diet	Bonjour, (2012) [36] 1 RCT (*n* = 71) 6 wk	France Mean age (SD): I: 57.1 (3.9) y C: 56.1 (3.9) y Sex: 100% female NR Postmenopausal	Food frequency questionnaire	I: mean (SD) 72 (17) g/d C: mean (SD) 199 (79) g/d	I: mean (SD) 5.49 (1.42) U/L C: mean (SD) 5.35 (1.38) U/L	I: change in TRAP: mean (SD): –0.64 (0.56) U/L C: change in TRAP: mean (SD): –0.34 (0.59) U/L	Found benefit	Insufficient
BAP Treated group (test food – 13.8 g protein) vs. usual diet	Bonjour, (2012) [36] 1 RCT (*n* = 71) 6 wk	France Mean age (SD): I: 57.1 (3.9) y C: 56.1 (3.9) y Sex: 100% female NR Postmenopausal	Food frequency questionnaire	I: mean (SD) 72 (17) g/d C: mean (SD) 199 (79) g/d	I: mean (SD) 11.3 (3.8) mg/L C: mean (SD) 10.8 (3.2) mg/L	I: mean (SD): –1.2 (1.8) mg/L C: mean (SD): –0.9 (1.2) mg/L	No difference	Insufficient
P1NP Treated group (test food – 13.8 g protein) vs. usual diet	Bonjour, (2012) [36] 1 RCT (*n* = 71) 6 wk	France Mean age (SD): I: 57.1 (3.9) y C: 56.1 (3.9) y Sex: 100% female NR Postmenopausal	Food frequency questionnaire	I: mean (SD) 72 (17) g/d C: mean (SD) 199 (79) g/d	I: mean (SD) 52.0 (19.7) mg/L C: mean (SD) 54.2 (20.3) mg/L	I: mean (SD): 0.25 (9.3) mg/L C: mean (SD): 2.8 (10.8) mg/L	No difference	Insufficient
Lumbar spine BMD I: high protein (45 g whey protein supplement isolate) vs. C: low protein (carbohydrate-isocaloric maltodextrin control supplement)	Kerstetter, (2015) [65] 1 RCT (*n* = 171) 18 mo	United States Mean age (SD): I: 69.9 (6.1) y C: 70.5 (6.4) y I: 84% females C: 87.3% females NR NR	3-d food record	I: mean (SEM) 73.8 (1.9) g/d C: mean (SEM) 72.9 (1.8) g/d	I: mean (SD) 1.09 (0.01) g/cm^2^ C: mean (SD) 1.10 (0.01) g/cm^2^	I: mean (SD): 1.10 (0.01) g/cm^2^ C: mean (SD): 1.11 (0.02) g/cm^2^	No difference	Insufficient
Lumbar spine BMD I: high protein diet (protein – 25% of total energy) vs. C: low protein diet (protein – 12% of total energy)	Skov, (2002) [100] 1 RCT (*n* = 50) 6 mo	Denmark Mean age (SD): I: 39.4 (2.0) y C: 39.8 (1.9) y I: 76% females C: 76% females Overweight or obese NR	Shop computer system	I: mean (SEM) 89.1 (3.9) g/d C: mean (SEM) 87.8 (5.0) g/d	I: mean (SEM)1.03 (0.02) g/cm^2^ C: mean (SEM) 1.17 (0.01) g/cm^2^	I: mean (SEM): 1.04 (0.02) g/cm^2^ C: mean (SEM): 1.01 (0.03) g/cm^2^	No difference	Insufficient
Lumbar spine BMD (L1-L4) No comparison arm	Rivera-Paredez, (2021) [93] 1 non-RCT (*n* = 317) 6.4 y	Mexico Mean age (SD): 57 y 100% females 26.5% Postmenopausal	Food frequency questionnaire	Whole cohort: median (IQR): 66.4 (51.1–86.0) g/d	Whole cohort: mean (SD): 1.035 (0.171) g/cm^2^	Whole cohort: mean (SD): 0.999 (0.893) g/cm^2^	No difference	Insufficient
Total hip BMD I: high protein (45 g whey protein supplement isolate) vs. C: low protein (carbohydrate -isocaloric maltodextrin control supplement)	Kerstetter, (2015) [65] 1 RCT (*n* = 171) 18 mo	United States Mean age (SD): I: 69.9 (6.1) y C: 70.5 (6.4) y I: 84% females C: 87.3% females NR NR	3-d food record	I: mean (SEM) 73.8 (1.9) g/d C: mean (SEM) 72.9 (1.8) g/d	I: mean (SD) 1.09 (0.01) g/cm^2^ C: mean (SD) 1.10 (0.01) g/cm^2^	I: mean (SD): 1.10 (0.01) g/cm^2^ C: mean (SD): 1.11 (0.02) g/cm^2^	No difference	Insufficient
Femoral neck BMD I: high protein (45 g whey protein supplement isolate) vs. C: low protein (carbohydrate -isocaloric maltodextrin control supplement)	Kerstetter, (2015) [65] 1 RCT (*n* = 171) 18 mo	United States Mean age (SD): I: 69.9 (6.1) y C: 70.5 (6.4) y I: 84% females C: 87.3% females NR NR	3-d food record	I: mean (SEM) 73.8 (1.9) g/d C: mean (SEM) 72.9 (1.8) g/d	I: mean (SD) 1.09 (0.01) g/cm^2^ C: mean (SD) 1.10 (0.01) g/cm^2^	I: mean (SD): 1.10 (0.01) g/cm^2^ C: mean (SD): 1.11 (0.02) g/cm^2^	No difference	Insufficient
Total hip BMD No comparison arm	Rivera-Paredez, (2021) [93] 1 non-RCT (*n* = 317) 6.4 y	Mexico Mean age (SD): 57 y 100% females 26.5% Postmenopausal	Food frequency questionnaire	Whole cohort: median (IQR): 66.4 (51.1–86.0) g/d	Whole cohort: mean (SD): 1.035 (0.171) g/cm^2^	Whole cohort: mean (SD): 0.999 (0.893) g/cm^2^	No difference	Insufficient
Femoral neck BMD No comparison arm	Rivera-Paredez, (2021) [93] 1 non-RCT (*n* = 317) 6.4 y	Mexico Mean age (SD): 57 y 100% females 26.5% Postmenopausal	Food frequency questionnaire	Whole cohort: median (IQR): 66.4 (51.1–86.0) g/d	Whole cohort: mean (SD): 1.035 (0.171) g/cm^2^	Whole cohort: mean (SD): 0.999 (0.893) g/cm^2^	No difference	Insufficient
Total body BMD I: high protein diet (protein – 25% of total energy) vs. C: low protein diet (protein – 12% of total energy)	Skov, (2002) [100] 1 RCT (*n* = 50) 6 mo	Denmark Mean age (SD): I: 39.4 (2.0) y C: 39.8 (1.9) y I: 76% females C: 76% females Overweight or obese NR	Shop computer system	I: mean (SEM) 89.1 (3.9) g/d C: mean (SEM) 87.8 (5.0) g/d	I: mean (SEM)1.03 (0.02) g/cm^2^ C: mean (SEM) 1.17 (0.01) g/cm^2^	I: mean (SEM): 1.04 (0.02) g/cm^2^ C: mean (SEM): 1.01 (0.03) g/cm^2^	No difference	Insufficient
Total body BMC I: high protein diet (protein – 25% of total energy) vs. C: low protein diet (protein – 12% of total energy)	Skov, (2002) [100] 1 RCT (*n* = 50) 6 mo	Denmark Mean age (SD): I: 39.4 (2.0) y C: 39.8 (1.9) y I: 76% females C: 76% females Overweight or obese NR	Shop computer system	I: mean (SEM) 89.1 (3.9) g/d C: mean (SEM) 87.8 (5.0) g/d	I: mean (SEM)1.03 (0.02) g/cm^2^ C: mean (SEM) 1.17 (0.01) g/cm^2^	I: mean (SEM): 1.04 (0.02) g/cm^2^ C: mean (SEM): 1.01 (0.03) g/cm^2^	No difference	Insufficient

Abbreviations: BAP, bone alkaline phosphatase; BMC, bone mineral content; BMD, bone mineral density; C, control; CTX, C-terminal peptide of collagen; I, intervention; IQR, interquartile range; L1, lumbar vertebrae 1; L4, lumbar vertebrae 4; non-RCT, nonrandomized controlled trial; NR, not reported; OC, osteocalcin; P1NP, procollagen type 1 N-terminal propeptide; RCT, randomized controlled trial; TRAP, tartrate-resistant alkaline phosphatase.

1 Indicates significant difference.

2 Strength of evidence was evaluated based on 5 designated domains outlined in the Methods section and was insufficient. The main reasons for this insufficient rating were that the evidence was derived from a single study, making it impossible to assess consistency, and in some instances, the outcome effect estimate was imprecise.

#### Bone turnover markers

One RCT [36] reported no difference in bone turnover markers osteocalcin (OC), C-terminal peptide of collagen, bone alkaline phosphatase, and procollagen type 1 N-terminal propeptide between the intervention and comparator. An inverse effect of protein intake on tartrate-resistant alkaline phosphatase was reported (Table 1).

#### BMD of the axial skeleton (lumbar spine)

Two RCTs reported no difference in the lumbar spine BMD between the intervention and comparator (Table 1) [65,100]. Also, evidence from a prospective cohort study reported no difference in lumbar spine BMD (Table 1) [93].

#### BMD of the appendicular skeleton (total hip and femoral neck)

One RCT reported no difference in the total hip BMD and femoral neck BMD between the intervention and comparator [65]. Also, evidence from a prospective cohort study reported no difference in total hip BMD and a positive association between protein intake and femoral neck BMD [93].

#### Total body BMD and BMC

One RCT reported no difference in total body BMD between the intervention and comparator; however, a positive effect of protein intake on total body BMC was found [100].

### Bone disease in children

#### Characteristics and findings of the analyzed study

The analytic set included 1 RCT in children and adolescents (low RoB and the only eligible child study) [103]. Characteristics and findings of the 1 analyzed RCT are summarized in Table 2 [103]. This study was conducted in Denmark. The dietary protein intake intervention and comparator consisted of 2 intervention arms that had a high protein intake with vitamin D or placebo intervention and 2 comparator arms that had normal protein intake with vitamin D or placebo intervention. The study follow-up was 24 wk. Based on the results from this 1 study, the evidence was insufficient to conclude whether protein intake was associated with changes in bone outcomes among children and adolescents.

**TABLE 2:** Summary of findings for bone disease outcomes in children and adolescents.

Outcome comparisons	Author, (year) (*n* analyzed) Study duration	Country Age Sex Obesity status Menopausal status	Protein assessment method	Baseline protein	Outcome baseline	Outcome follow-up	Direction of effect^1^	Strength of the evidence^2^
OC I: high protein (9–11 g protein/100 g) vs. C: normal protein (3.0–3.9 g protein/100 g)	Stounbjerg, (2021) [103] 1 RCT (*n* = 152) 24 wk	Denmark Median age: I1: 7.8 y I2: 7.8 y C1: 7.6 y C2: 7.6 y I1: 48% females I2: 44% females C1: 53% females C2: 61% females 14% obese	Food frequency questionnaire	I1: mean (SD) 15.4 (2.4)% of energy I2: mean (SD) 15.7 (2.3)% of energy C1: mean (SD) 15.0 (2.2)% of energy C2: mean (SD) 15.7 (2.6)% of energy	I1: mean (SD): 38.3 (9.1) μg/L I2: mean (SD): 37.1 (10.8) μg/L C1: mean (SD): 38.1 (11.9) μg/L C2: mean (SD) 37.1 (9.5) μg/L	I1: mean (SD): 38.3 (9.1) μg/L I2: mean (SD): 38.2 (10.0) μg/L C1: mean (SD): 5.3 (8.5) μg/L C2: mean (SD): 39.8 (9.8) μg/L	Found benefit	Insufficient
Lumbar spine BMD (L1-L4) I: high protein (9–11 g protein/100 g) vs. C: normal protein (3.0–3.9 g protein/100 g)	Stounbjerg, (2021) [103] 1 RCT (*n* = 184) 24 wk	Denmark Median age: I1: 7.8 y I2: 7.8 y C1: 7.6 y C2: 7.6 y I1: 48% females I2: 44% females C1: 53% females C2: 61% females 14% obese	Food frequency questionnaire	I1: mean (SD) 15.4 (2.4)% of energy I2: mean (SD) 15.7 (2.3)% of energy C1: mean (SD) 15.0 (2.2)% of energy C2: mean (SD) 15.7 (2.6)% of energy	I1: mean (SD): 0.681 (0.074) g/cm^2^ I2: mean (SD): 0.682 (0.084) g/cm^2^ C1: mean (SD): 0.691 (0.078) g/cm^2^ C2: mean (SD): 0.679 (0.074) g/cm^2^	I1: mean (SD): 0.681 (0.074) g/cm^2^ I2: mean (SD): 0.692 (0.082) g/cm^2^ C1: mean (SD): 0.702 (0.086) g/cm^2^ C2: mean (SD): 0.695 (0.078) g/cm^2^	Found benefit	Insufficient
Lumbar spine BMD (L1-L4) *z*-score I: high protein (9–11 g protein/100 g) vs. C: normal protein (3.0–3.9 g protein/100 g)	Stounbjerg, (2021) [103] 1 RCT (*n* = 184) 24 wk	Denmark Median age: I1: 7.8 y I2: 7.8 y C1: 7.6 y C2: 7.6 y I1: 48% females I2: 44% females C1: 53% females C2: 61% females 14% obese	Food frequency questionnaire	I1: mean (SD) 15.4 (2.4)% of energy I2: mean (SD) 15.7 (2.3)% of energy C1: mean (SD) 15.0 (2.2)% of energy C2: mean (SD) 15.7 (2.6)% of energy	I1: mean (SD): 0.056 (0.807) I2: mean (SD): 0.077 (0.955) C1: mean (SD): 0.152 (0.918) C2: mean (SD): 0.022 (0.836)	I1: mean (SD): 0.056 (0.807) I2: mean (SD): 0.066 (0.908) C1: mean (SD): 0.145 (0.980) C2: mean (SD): 0.073 (0.852)	Found benefit	Insufficient
Lumbar spine BMC (L1-L4) I: high protein (9–11 g protein/100 g) vs. C: normal protein (3.0–3.9 g protein/100 g)	Stounbjerg, (2021) [103] 1 RCT (*n* = 184) 24 wk	Denmark Median age: I1: 7.8 y I2: 7.8 y C1: 7.6 y C2: 7.6 y I1: 48% females I2: 44% females C1: 53% females C2: 61% females 14% obese	Food frequency questionnaire	I1: mean (SD) 15.4 (2.4)% of energy I2: mean (SD) 15.7 (2.3)% of energy C1: mean (SD) 15.0 (2.2)% of energy C2: mean (SD) 15.7 (2.6)% of energy	I1: mean (SD): 21.5 (4.4) g I2: mean (SD): 21.8 (4.2) g C1: mean (SD): 22.4 (4.6) g C2: mean (SD): 22.3 (4.1) g	I1: mean (SD): 21.5 (4.4) g I2: mean (SD): 23.2 (4.3) g C1: mean (SD): 23.8 (5.2) g C2: mean (SD): 23.6 (4.5) g	No difference	Insufficient
Lumbar spine BA (L1-L4) I: high protein (9–11 g protein/100 g) vs. C: normal protein (3.0–3.9 g protein/100 g)	Stounbjerg, (2021) [103] 1 RCT (*n* = 184) 24 wk	Denmark Median age: I1: 7.8 y I2: 7.8 y C1: 7.6 y C2: 7.6 y I1: 48% females I2: 44% females C1: 53% females C2: 61% females 14% obese	Food frequency questionnaire	I1: mean (SD) 15.4 (2.4)% of energy I2: mean (SD) 15.7 (2.3)% of energy C1: mean (SD) 15.0 (2.2)% of energy C2: mean (SD) 15.7 (2.6)% of energy	I1: mean (SD): 1.3 (4.2) cm^2^ I2: mean (SD): 31.9 (3.7) cm^2^ C1: mean (SD): 32.2 (3.8) cm^2^ C2: mean (SD): 32.7 (3.4) cm^2^	I1: mean (SD): 1.3 (4.2) cm^2^ I2: mean (SD): 33.3 (3.8) cm^2^ C1: mean (SD): 33.8 (4.3) cm^2^ C2: mean (SD): 33.8 (3.6) cm^2^	No difference	Insufficient

Abbreviations: BA, bone area; BMC, bone mineral content; BMD, bone mineral density; C, control; I, intervention; OC, osteocalcin; RCT, randomized controlled trial; SD, standard deviation.

1 Indicates statistical significance.

2 Strength of evidence was evaluated based on 5 designated domains outlined in the Methods section and was insufficient. The main reasons for this insufficient rating were that the evidence was derived from a single study, making it impossible to assess consistency, and in some instances, the outcome effect estimate was imprecise.

#### Bone turnover markers, BMD and BMC of the axial skeleton, and bone geometry and strength indices

One RCT reported findings of inverse effect of protein intake on OC (a bone turnover marker), positive effects of protein intake on lumbar spine BMD (L1-L4) and lumbar spine BMD (L1-L4) *z*-score, no effect of protein intake on lumbar spine BMC (L1-L4), and no effect of protein intake on lumbar spine bone area (BA) (L1-L4) [103].

### Kidney disease in adults

#### Characteristics and findings of the analyzed study

The analytic set included 1 kidney disease study [1 RCT (moderate RoB)] [107]. Characteristics and findings of this study, conducted in Australia, are summarized in Table 3 [107]. The dietary protein intake intervention and comparator arm consisted of high protein intake compared to low protein intake, respectively. The study follow-up was 52 wk. Based on the results from this 1 study, the evidence was insufficient to draw conclusions on the effect of protein intake on kidney function (determined by creatinine clearance). The study reported no difference in creatinine clearance between the intervention and comparator [107].

**TABLE 3:** Summary of findings for kidney disease outcomes in adults in adults.

Outcome comparisons	Author, (year) (*n* analyzed) Study duration	Country Age Sex Obesity status Menopausal status	Protein assessment method	Baseline protein	Outcome baseline	Outcome follow-up	Direction of effect^1^	Strength of the evidence^2^
Creatinine clearance I: high protein (35% energy from protein) vs. C: low protein (high carbohydrate – 17% energy from protein)	Wycherley, (2012) [107] 1 RCT (*n* = 120)^3^ 52 wk	Australia Mean age (SD): I: 51.3 (9.4) y C: 50.2 (9.3) y 0% females 100% overweight or obese NA	2-wk food record	NR^3^	NR^3^	NR^3^	No difference	Insufficient

Abbreviations: C, control; I, intervention; NA, not applicable; NR, not reported; RCT, randomized controlled trial; SD, standard deviation.

1 Indicates statistical significance.

2 Strength of evidence was evaluated based on 5 designated domains outlined in the Methods section and was insufficient. The main reasons for this insufficient rating were that the evidence was derived from a single study, making it impossible to assess consistency, and in some instances, the outcome effect estimate was imprecise.

3 Baseline characteristics and follow-up information were presented for participants who completed the 52-wk intervention, but intention-to-treat evaluation was conducted for the full sample (*n* = 120).

### Sarcopenia in adults

#### Characteristics and findings of the analyzed studies

The analytic set included 9 sarcopenia studies [32,45,51,65,75,92,101,107,108] [9 RCTs (7 low and 2 moderate RoB)]. Characteristics and findings of the analytic set are summarized in Table 4 [32,45,51,65,75,92,101,107,108]. Studies were conducted in various countries, including Australia [107,108], Netherlands [32], Netherlands and Finland [92], China [75], Germany [45], Iran [51], and the United States [65,101]. The dietary protein intake interventions and comparators were different across RCTs. The study follow-up ranged from 12 wk to 2 y. Different cut-off points (such as low, normal, and high) were used to describe protein intake levels. Protein measurement units also differed, including absolute protein intake in grams (g), protein intake per body weight (g/kg body weight), or protein intake per energy intake (%). The evidence was insufficient to draw conclusions about any outcome related to the risk of sarcopenia.

**TABLE 4:** Summary of findings for sarcopenia outcomes in adults.

Outcome comparisons	Author, (year) (*n* analyzed) Study duration	Country Age Sex Obesity status Menopausal status	Protein assessment method	Baseline protein	Outcome baseline	Outcome follow-up	Direction of effect^1^	Strength of the evidence^2^
Total body lean mass I: high protein diet (contains 1.7 g of protein/kg/d) vs. C: normal protein diet (contains 0.9 g protein/kg/d)	Backx, (2016) [32] (*n* = NR) 12 wk	Netherlands Mean age (SD): I: 63 (4.8) y C: 62 (4.8) y I: 41.9% female C: 40% female All overweight or obese with a BMI (in kg/m^2^) between 27 and 40 Postmenopausal	Chemical analysis of the duplicate meals, food diaries, and frequency questionnaire	I: mean (SD): 1.1 (0.4) g/kg/d; 14% of energy C: Mean (SD): 1.1 (0.4) g/kg/d; 14% of energy	I: mean (SD): 54.8 (12.2) kg C: mean (SD): 54.5 (9.3) kg	I: mean (SD): 53.1 (11.4) kg C: mean (SD): 52.4 (9.1) kg	No difference	Insufficient
Total body lean mass I: high protein (45 g whey protein supplement isolate) vs. C: low protein (carbohydrate -isocaloric maltodextrin control supplement)	Kerstetter, (2015) [65] (*n* = 207) 18 mo	United States Mean age (SD): I: 69.9 (6.1) y C: 70.5 (6.4) y I: 84% female C: 87.3% female NR NR	3-d food record	I: least square mean (SEM): 73.8 (1.9) g/d C: least square mean (SEM): 72.9 (1.8) g/d	I: mean (SEM): 42.6 (0.8) kg C: mean (SEM): 42.0 (0.8) kg	I: mean (SEM): 42.6 (0.8) kg C: mean (SEM): 41.5 (0.8) kg	No difference	Insufficient
Total body lean mass I1: whey protein (whey protein blended supplement), I2: soy protein (soy protein blended supplement), I3: whey-soy protein group (1:1 ratio of whey and soy blended supplement) vs. C: control (no supplementation)	Li, (2021) [75] (*n* = 123) 6 mo	China Mean age (SD): I1: 71 (4) y I2: 69 (4) y I3: 70 (4) y C: 71 (4) y I1: 48.4% female I2: 51.6% female I3: 45.2% female C: 56.7% female NR NR	Food frequency questionnaire	I1: mean (SD): 62.7 (20.7) g/d; 1.14 (0.36) g/kg/d I2: mean (SD): 59.6 (19.1) g/d; 1.11 (0.33) g/kg/d I3: mean (SD): 61.1 (19.1) g/d; 1.14 (0.37) g/kg/d C: mean (SD): 59.3 (18.8) g/d; 1.17 (0.30) g/kg/d	I1: mean (SD): 34.96 (6.75) kg I2: mean (SD): 34.66 (6.83) kg I3: mean (SD): 35.49 (6.49) kg C: mean (SD): 33.79 (6.17) kg	I1: mean (SD): 35.13 (6.4) kg I2: mean (SD): 34.84 (6.78) kg I3: mean (SD): 35.77 (6.57) kg C: mean (SD): 33.32 (6.0) kg	Found benefit	Insufficient
Total body lean mass I: weight loss plus whey protein supplement (hypocaloric diet with increased protein intake 1.2 g/kg/d) vs. C: weight loss plus recommended protein (hypocaloric diet with 0.8 g/kg/d protein)	Smith, (2018) [101] (*n* = 52) 6 mo	United States NR 100% female 100% obese Postmenopausal	1-wk food record	NR	I: mean (SEM): 44.4 (1.0) kg C: mean (SEM): 45.7 (0.9) kg	I: mean (SEM): 43.3 (1.0) kg C: mean (SEM): 44.2 (1.0) kg	No difference	Insufficient
Total body skeletal muscle mass I: high protein (high protein snack (50 g of soybeans, protein: 18.2 g) vs. C: low protein (∼3.5 servings of fruit, protein: <2 g)	Haghighat, (2021) [51] (*n* = 107) 6 mo	Iran Mean age (SD): 24 (3) y 100% female Normal weight obesity (body fat percentage >30%) Premenopausal	24-h food dietary recall questionnaire	I: mean (SD): 51.37 (7.36) g/d; 0.84 (0.15) g/kg/d C: mean (SD): 48.80 (7.21) g/d; 0.79 (0.14) g/kg/d	NR	I: mean increase 1.2 kg (95% CI: 1.5, 1) C: mean increase 0.3 kg (95% CI: 0.7, 0.02)	Found benefit	Insufficient
Total body FFM I: high protein (1.5 g/kg body weight) vs. C: normal protein (0.8 g/kg body weight)	Englert, (2021) [45] (*n* = 54) 12 wk	Germany Mean age (SD): I: 59.0 (6) y C: 58.7 (6) y 100% female All females overweight, BMI ≥30 or ≥27 and waist circumference >88 cm Postmenopausal	Food diary and food checklists	NR	I: mean (SD): 46.8 (6.9) kg C: mean (SD): 46.7 (5.0) kg	I: mean (SD) (change at 12 wk): –0.9 (1.1) kg C: mean (SD) (change at 12 wk): –1.0 (1.3) kg	No difference	Insufficient
Total body FFM I: protein advice (advised to increase protein intake to ≥1.2 g/kg aBW/d) vs. C: control (no advice to increase protein consumption)	Reinders, (2022) [92] (*n* = 187) 6 mo	Finland, Netherlands Mean age (SD): I: 75.9 (5.0) y C: 75.0 (4.4) y I: 52.1% female C: 54.9% female NR NR	3-d food diary and 24-h food dietary recall questionnaire	I: mean (SD): 60.4 (1.3) g/d; 0.82 (0.01) g/kg aBW/d C: mean (SD): 60.5 (1.2) g/d; 0.82 (0.01) g/kg aBW/d	I: mean (SE): 52.0 (1.06) kg C: mean (SE): 51.8 (0.97) kg	I: mean (SE): 52.6 (1.15) kg C: mean (SE): 52.1 (0.99) kg	No difference	Insufficient
Total body FFM I: weight loss plus whey protein supplement (hypocaloric diet with increased protein intake 1.2 g/kg/d) vs. C: weight loss plus recommended protein (hypocaloric diet with 0.8 g/kg/d protein)	Smith, (2018) [101] (*n* = 52) 6 mo	United States NR 100% female 100% obese Postmenopausal	1-wk food record	NR	I: mean (SEM): 46.9 (1.0) kg C: mean (SEM): 48.2 (1.0) kg	I: mean (SEM): 45.8 (1.0) kg C: mean (SEM): 46.7 (1.0) kg	No difference	Insufficient
Total body FFM I: high protein (35% energy from protein) vs. C: low protein (high carbohydrate - 17% energy from protein)	Wycherley, (2012) [107] (*n* = 120) 52 wk	Australia Mean age (SD): I: 51.3 (9.4) y C: 50.2 (9.3) y 0% female 100% overweight or obese NA	2-wk food record	NR^3^	NR^3^	NR^3^	No difference	Insufficient
Appendicular lean mass/skeletal muscle mass I: high protein diet (contain 1.7 g of protein/kg/d) vs. C: normal protein diet (contain 0.9 g protein/kg/d)	Backx, (2016) [32] (*n* = NR) 12 wk	Netherlands Mean age (SD): I: 63 (4.8) y C: 62 (4.8) y I: 41.9% female C: 40% female All overweight or obese with BMI between 27 and 40 Postmenopausal	Chemical analysis of the duplicate meals, food diaries, and frequency questionnaire	I: mean (SD): 1.1 (0.4) g/kg/d; 14% of energy C: mean (SD): 1.1 (0.4) g/kg/d; 14% of energy	I: mean (SD): 23.8 (5.5) kg C: mean (SD): 23.8 (4.8) kg	I: mean (SD): 23.1 (5.4) kg C: mean (SD): 22.8 (4.6) kg	No difference	Insufficient
Appendicular lean mass/skeletal muscle mass I1: whey protein (whey protein blended supplement), I2: soy protein (soy protein blended supplement), I3: whey-soy protein group (1:1 ratio of whey and soy blended supplement) vs. C: control (no supplementation)	Li, (2021) [75] (*n* = 123) 6 mo	China Mean age (SD): I1: 71 (4) y I2: 69 (4) y I3: 70 (4) y C: 71 (4) y I1: 48.4% female I2: 51.6% female I3: 45.2% female C: 56.7% female NR NR	Food frequency questionnaire	I1: mean (SD): 62.7 (20.7) g/d; 1.14 (0.36) g/kg/d I2: mean (SD): 59.6 (19.1) g/d; 1.11 (0.33) g/kg/d I3: mean (SD): 61.1 (19.1) g/d; 1.14 (0.37) g/kg/d C: mean (SD): 59.3 (18.8) g/d; 1.17 (0.30) g/kg/d	I1: mean (SD): 14.47 (3.34) kg I2: mean (SD): 14.46 (3.27) kg I3: mean (SD): 15.07 (3.33) kg C: mean (SD): 14.13 (3.03) kg	I1: mean (SD): 14.62 (3.10) kg I2: mean (SD): 14.54 (3.27) kg I3: mean (SD): 15.26 (3.38) kg C: mean (SD): 13.76 (2.98) kg	Found benefit	Insufficient
Appendicular lean mass/skeletal muscle mass I: high protein (supplement drink - 30 g of protein/d) vs. C: placebo supplement (high-carbohydrate drink supplement drink - 2.1 g of protein/d)	Zhu, (2015) [108] (*n* = 181) 2 y	Australia Mean age (SD): I: 74.2 (2.8) y C: 74.3 (2.6) y 100% female NR Postmenopausal	3-d weighed food record	I: mean (SD): 76 (18) g/d; 1.2 (0.3) g/kg/d C: mean (SD): 76 (16) g/d; 1.1 (0.3) g/kg/d	I: mean (SD): 16.2 (2.4) kg C: mean (SD): 16.6 (2.4) kg	I: mean (SEM) (change at 2 y): –0.03 (0.07) kg C: mean (SEM) (change at 2 y): 0.03 (0.08) kg	No difference	Insufficient
Appendicular skeletal muscle mass index I1: whey protein (whey protein blended supplement), I2: soy protein (soy protein blended supplement), I3: whey-soy protein group (1:1 ratio of whey and soy blended supplement) vs. C: control (no supplementation)	Li, (2021) [75] (*n* = 123) 6 mo	China Mean age (SD): I1: 71 (4) y I2: 69 (4) y I3: 70 (4) y C: 71 (4) y I1: 48.4% female I2: 51.6% female I3: 45.2% female C: 56.7% female NR NR	Food frequency questionnaire	I1: mean (SD): 62.7 (20.7) g/d; 1.14 (0.36) g/kg/d I2: mean (SD): 59.6 (19.1) g/d; 1.11 (0.33) g/kg/d I3: mean (SD): 61.1 (19.1) g/d; 1.14 (0.37) g/kg/d C: mean (SD): 59.3 (18.8) g/d; 1.17 (0.30) g/kg/d	I1: mean (SD): 5.70 (0.92) kg/m^2^ I2: mean (SD): 5.62 (0.83) kg/m^2^ I3: mean (SD): 5.68 (0.81) kg/m^2^ C: mean (SD): 5.65 (0.84) kg/m^2^	I1: mean (SD): 5.76 (0.81) kg/m^2^ I2: mean (SD): 5.65 (0.84) kg/m^2^ I3: mean (SD): 5.75 (0.80) kg/m^2^ C: mean (SD): 5.50 (0.81) kg/m^2^	Found benefit	Insufficient
Appendicular skeletal muscle mass index I: high protein (supplement drink - 30 g of protein/d) vs. C: placebo supplement (high-carbohydrate drink supplement drink - 2.1 g of protein/d)	Zhu, (2015) [108] (*n* = 181)2 y	Australia Mean age (SD): I: 74.2 (2.8) y C: 74.3 (2.6) y 100% female NR Postmenopausal	3-d weighed food record	I: mean (SD): 76 (18) g/d; 1.2 (0.3) g/kg/d C: mean (SD): 76 (16) g/d; 1.1 (0.3) g/kg/d	I: mean (SD): 6.3 (0.7) kg/m^2^ C: mean (SD): 6.5 (0.8) kg/m^2^	I: mean (SEM) (change at 2 y): 0.02 (0.03) kg/m^2^ C: mean (SEM) (change at 2 y): 0.05 (0.03) kg/m^2^	No difference	Insufficient
TUG I: high protein (supplement drink - 30 g of protein/d) vs. C: placebo supplement (high-carbohydrate drink supplement drink - 2.1 g of protein/d)	Zhu, (2015) [108] 1 RCT (*n* = 181) 2 y	Australia Mean age (SD): I: 74.2 (2.8) y C: 74.3 (2.6) y 100% female NR Postmenopausal	3-d weighed food record	I: mean (SD): 76 (18) g/d; 1.2 (0.3) g/kg/d C: mean (SD): 76 (16) g/d; 1.1 (0.3) g/kg/d	I: mean (SD): 7.9 (1.3) s C: mean (SD): 8.0 (1.5) s	I: mean (SEM) (change at 2 y): 0.46 (0.12) s C: mean (SEM) (change at 2 y): –0.55 (0.12) s	No difference	Insufficient
4 m gait speed I1: whey protein (whey protein blended supplement), I2: soy protein (soy protein blended supplement), I3: whey-soy protein group (1:1 ratio of whey and soy blended supplement) vs. C: control (no supplementation)	Li, (2021) [75] (*n* = 123) 6 mo	China Mean age (SD): I1: 71 (4) y I2: 69 (4) y I3: 70 (4) y C: 71 (4) y I1: 48.4% female I2: 51.6% female I3: 45.2% female C: 56.7% female NR NR	Food frequency questionnaire	I1: mean (SD): 62.7 (20.7) g/d; 1.14 (0.36) g/kg/d I2: mean (SD): 59.6 (19.1) g/d; 1.11 (0.33) g/kg/d I3: mean (SD): 61.1 (19.1) g/d; 1.14 (0.37) g/kg/d C: mean (SD): 59.3 (18.8) g/d; 1.17 (0.30) g/kg/d	I1: mean (SD):1.12 (0.2) min/s I2: mean (SD): 1.17 (0.16) min/s I3: mean (SD): 1.15 (0.20) min/s C: mean (SD): 1.12 (0.1) min/s	I1: mean (SD): 1.14 (0.12) min/s I2: mean (SD): 1.15 (0.14) min/s I3: mean (SD): 1.13 (0.17) min/s C: mean (SD): 0.96 (0.16) min/s	Found benefit	Insufficient
400 m walk speed I: high protein diet (contain 1.7 g of protein/kg/d) vs. C: normal protein diet (contain 0.9 g protein/kg/d)	Backx, (2016) [32] (*n* = 59) 12 wk	Netherlands Mean age (SD): I: 63 (4.8) y C: 62 (4.8) y I: 41.9% female C: 40% female All overweight or obese with a BMI between 27 and 40 Postmenopausal	Chemical analysis of the duplicate meals, food diaries, and frequency questionnaire	I: mean (SD): 1.1 (0.4) g/kg/d; 14% of energy C: mean (SD): 1.1 (0.4) g/kg/d; 14% of energy	I: mean (SD): 1.46 (0.19) min/s C: mean (SD): 1.45 (0.19) min/s	I: mean (SD): 1.5 (0.2) min/s C: mean (SD): 1.47 (0.22) min/s	No difference	Insufficient
400 m walk speed I: high protein (1.5 g/kg body weight) vs. C: normal protein (0.8 g/kg body weight)	Englert, (2021) [45] (*n* = 54) 12 wk	Germany Mean age (SD): I: 59.0 (6) y C: 58.7 (6) y 100% female All females overweight, BMI ≥30 or ≥27 and waist circumference >88 cm Postmenopausal	Food diary and food checklists	NR	I: mean (SD): 4:10 (0:33) min:s C: mean (SD): 4:11 (0:31) min:s	I: mean (SD) (change at 12 wk): –0:00 (0:07) min:s C: mean (SD) (change at 12 wk): –0:05 (0:12) min:s	No difference	Insufficient
400 m walk speed I: protein advice (advised to increase protein intake to ≥1.2 g/kg aBW/d) vs. C: control (no advice to increase protein consumption)	Reinders, (2022) [92] (*n* = 187) 6 mo	Finland, Netherlands Mean age (SD): I: 75.9 (5.0) y C: 75.0 (4.4) y I: 52.1% female C: 54.9% female NR NR	3-d food diary and 24-h food dietary recall questionnaire	I: mean (SD): 60.4 (1.3) g/d; 0.82 (0.01) g/kg aBW/d C: mean (SD): 60.5 (1.2) g/d; 0.82 (0.01) g/kg aBW/d	I: mean (SE): 311.3 (7.2) s C: mean (SE): 311.1 (9.3) s	I: mean (SE): 306.0 (6.85) s C: mean (SE): 318.2 (11.0) s	Found benefit	Insufficient
SPPB I: high protein diet (contain 1.7 g of protein/kg/d) vs. C: normal protein diet (contain 0.9 g protein/kg/d)	Backx, (2016) [32] (*n* = 60) 12 wk	Netherlands Mean age (SD): I: 63 (4.8) y C: 62 (4.8) y I: 41.9% female C: 40% female All overweight or obese with a BMI between 27 and 40 Postmenopausal	Chemical analysis of the duplicate meals, food diaries, and frequency questionnaire	I: mean (SD): 1.1 (0.4) g/kg/d; 14% of energy C: mean (SD): 1.1 (0.4) g/kg/d; 14% of energy	I: mean (SD): 11.6 (0.7) C: mean (SD): 11.4 (0.9)	I: mean (SD): 11.7 (0.5) C: mean (SD): 11.6 (0.6)	No difference	Insufficient
SPPB I: high protein (1.5 g/kg body weight) vs. C: normal protein (0.8 g/kg body weight)	Englert, (2021) [45] (*n* = 54) 12 wk	Germany Mean age (SD): I: 59.0 (6) y C: 58.7 (6) y 100% female All females overweight, BMI ≥30 or ≥27 and waist circumference >88 cm Postmenopausal	Food diary and food checklists	NR	I: mean (SD): 9.4 (1.1) C: mean (SD): 9.9 (1.0)	I: mean (SD) (change at 12 wk): +0.4 (0.09) C: mean (SD) (change at 12 wk): +0.6 (0.8)	No difference	Insufficient
SPPB I: protein advice (advised to increase protein intake to ≥1.2 g/kg aBW/d) vs. C: control (no advice to increase protein consumption)	Reinders, (2022) [92] (*n* = 187) 6 mo	Finland, Netherlands Mean age (SD): I: 75.9 (5.0) y C: 75.0 (4.4) y I: 52.1% female C: 54.9% female NR NR	3-d food diary and 24-h food dietary recall questionnaire	I: mean (SD): 60.4 (1.3) g/d; 0.82 (0.01) g/kg aBW/d C: mean (SD): 60.5 (1.2) g/d; 0.82 (0.01) g/kg aBW/d	I: mean (SE): 9.8 (0.14) C: mean (SE): 9.7 (0.17)	I: mean (SE): 10.0 (0.14) C: mean (SE): 10.0 (0.17)	No difference	Insufficient
SPPB I1: whey protein (whey protein blended supplement), I2: soy protein (soy protein blended supplement), I3: whey-soy protein group (1:1 ratio of whey and soy blended supplement) vs. C: control (no supplementation)	Li, (2021) [75] (*n* = 123) 6 mo	China Mean age (SD): I1: 71 (4) y I2: 69 (4) y I3: 70 (4) y C: 71 (4) y I1: 48.4% female I2: 51.6% female I3: 45.2% female C: 56.7% female NR NR	Food frequency questionnaire	I1: mean (SD): 62.7 (20.7) g/d; 1.14 (0.36) g/kg/d I2: mean (SD): 59.6 (19.1) g/d; 1.11 (0.33) g/kg/d I3: mean (SD): 61.1 (19.1) g/d; 1.14 (0.37) g/kg/d C: mean (SD): 59.3 (18.8) g/d; 1.17 (0.30) g/kg/d	I1: mean (SD): 11.23 (0.8) I2: mean (SD): 11.58 (0.56) I3: mean (SD): 11.39 (0.88) C: mean (SD): 11.51 (0.62)	I1: mean (SD): 11.65 (0.61) I2: mean (SD): 11.52 (0.63) I3: mean (SD): 11.71 (0.78) C: mean (SD): 10.61 (1.28)	Found benefit	Insufficient
Handgrip strength I: high protein diet (contain 1.7 g of protein/kg/d) vs. C: normal protein diet (contain 0.9 g protein/kg/d)	Backx, (2016) [32] (*n* = 60) 12 wk	Netherlands Mean age (SD): I: 63 (4.8) y C: 62 (4.8) y I: 41.9% female C: 40% female All overweight or obese with a BMI between 27 and 40 Postmenopausal	Chemical analysis of the duplicate meals, food diaries, and frequency questionnaire	I: mean (SD): 1.1 (0.4) g/kg/d; 14% of energy C: mean (SD): 1.1 (0.4) g/kg/d; 14% of energy	I: mean (SD): 40 (11) kg C: mean (SD): 41 (10) kg	I: mean (SD): 37 (9) kg C: mean (SD): 40 (11) kg	No difference	Insufficient
Handgrip strength I: high protein (1.5 g/kg body weight) vs. C: normal protein (0.8 g/kg body weight)	Englert, (2021) [45] (*n* = 54) 12 wk	Germany Mean age (SD): I: 59.0 (6) y C: 58.7 (6) y 100% female All females overweight, BMI ≥30 or ≥27 and waist circumference >88 cm Postmenopausal	Food diary and food checklists	NR	I: mean (SD): 28.7 (7.2) kg C: mean (SD): 29.0 (4.9) kg	I: mean (SD) (change at 12 wk): +0.01 (2.6) kg C: mean (SD) (change at 12 wk): –1.6 (3.3) kg	Found benefit	Insufficient
Handgrip strength I1: whey protein (whey protein blended supplement), I2: soy protein (soy protein blended supplement), I3: whey-soy protein group (1:1 ratio of whey and soy blended supplement) vs. C: control (no supplementation)	Li, (2021) [75] (*n* = 123) 6 mo	China Mean age (SD): I1: 71 (4) y I2: 69 (4) y I3: 70 (4) y C: 71 (4) y I1: 48.4% female I2: 51.6% female I3: 45.2% female C: 56.7% female NR NR	Food frequency questionnaire	I1: mean (SD): 62.7 (20.7) g/d; 1.14 (0.36) g/kg/d I2: mean (SD): 59.6 (19.1) g/d; 1.11 (0.33) g/kg/d I3: mean (SD): 61.1 (19.1) g/d; 1.14 (0.37) g/kg/d C: mean (SD): 59.3 (18.8) g/d; 1.17 (0.30) g/kg/d	I1: mean (SD): 27.06 (7.78) kg I2: mean (SD): 26.88 (6.93) kg I3: mean (SD): 28.42 (8.81) kg C: mean (SD): 24.90 (7.33) kg	I: mean (SD): 26.78 (7.93) kg I2: mean (SD): 27.48 (7.03) kg I3: mean (SD): 28.45 (8.17) kg C: mean (SD): 25.33 (6.63) kg	No difference	Insufficient
Handgrip strength I: protein advice (advised to increase protein intake to ≥1.2 g/kg aBW/d) vs. C: control (no advice to increase protein consumption)	Reinders, (2022) [92] (*n* = 187) 6 mo	Finland, Netherlands Mean age (SD): I: 75.9 (5.0) y C: 75.0 (4.4) y I: 52.1% female C: 54.9% female NR NR	3-d food diary and 24-h food dietary recall questionnaire	I: mean (SD): 60.4 (1.3) g/d; 0.82 (0.01) g/kg aBW/d C: mean (SD): 60.5 (1.2) g/d; 0.82 (0.01) g/kg aBW/d	I: mean (SE): 30.2 (1.04) kg C: mean (SE): 29.2 (0.96) kg	I: mean (SE): 29.3 (1.05) kg C: mean (SE): 27.8 (0.93) kg	No difference	Insufficient
Handgrip strength I: high protein (supplement drink - 30 g of protein/d) vs. C: placebo supplement (high-carbohydrate drink supplement drink - 2.1 g of protein/d)	Zhu, (2015) [108] (*n* = 181) 2 y	Australia Mean age (SD): I: 74.2 (2.8) y C: 74.3 (2.6) y 100% female NR Postmenopausal	3-d weighed food record	I: mean (SD): 76 (18) g/d; 1.2 (0.3) g/kg/d C: mean (SD): 76 (16) g/d; 1.1 (0.3) g/kg/d	I: mean (SD): 21.7 (5.2) kg C: mean (SD): 21.7 (5.5) kg	I: mean (SEM) (change at 2 y): –1.09 (0.41) kg C: mean (SEM) (change at 2 y): –1.53 (0.42) kg	No difference	Insufficient
1-RM leg press I: high protein diet (contain 1.7 g of protein/kg/d) vs. C: normal protein diet (contain 0.9 g protein/kg/d)	Backx, (2016) [32] (*n* = 53) 12 wk	Netherlands Mean age (SD): I: 63 (4.8) y C: 62 (4.8) y I: 41.9% female C: 40% female All overweight or obese with a BMI between 27 and 40 Postmenopausal	Chemical analysis of the duplicate meals, food diaries, and frequency questionnaire	I: mean (SD): 1.1 (0.4) g/kg/d; 14% of energy C: mean (SD): 1.1 (0.4) g/kg/d; 14% of energy	I: mean (SD): 152 (44) kg C: mean (SD): 157 (33) kg	I: mean (SD): 143 (39) kg C: mean (SD): 148 (30) kg	No difference	Insufficient
Knee flexor strength I: high protein (supplement drink - 30 g of protein/d) vs. C: placebo supplement (high-carbohydrate drink supplement drink - 2.1 g of protein/d)	Zhu, (2015) [108] (*n* = 181) 2 y	Australia Mean age (SD): I: 74.2 (2.8) y C: 74.3 (2.6) y 100% female NR Postmenopausal	3-d weighed food record	I: mean (SD): 76 (18) g/d; 1.2 (0.3) g/kg/d C: mean (SD): 76 (16) g/d; 1.1 (0.3) g/kg/d	I: mean (SD): 9.1 (3.6) kg C: mean (SD): 9.7 (3.7) kg	I: mean (SEM) (change at 2 y): 3.18 (0.38) kg C: mean (SEM) (change at 2 y): 2.36 (0.49) kg	No difference	Insufficient
Leg extensor strength (1-RM leg extension) I: high protein diet (contain 1.7 g of protein/kg/d) vs. C: normal protein diet (contain 0.9 g protein/kg/d)	Backx, (2016) [32] (*n* = 53) 12 wk	Netherlands Mean age (SD): I: 63 (4.8) y C: 62 (4.8) y I: 41.9% female C: 40% female All overweight or obese with a BMI between 27 and 40 Postmenopausal	Chemical analysis of the duplicate meals, food diaries, and frequency questionnaire	I: mean (SD): 1.1 (0.4) g/kg/d; 14% of energy C: mean (SD): 1.1 (0.4) g/kg/d; 14% of energy	I: mean (SD): 93 (31) kg C: mean (SD): 98 (25) kg	I: mean (SD): 91 (29) kg C: mean (SD): 94 (25) kg	No difference	Insufficient
Leg extensor strength (knee extensor strength) I: high protein (supplement drink - 30 g of protein/d) vs. C: placebo supplement (high-carbohydrate drink supplement drink - 2.1 g of protein/d)	Zhu, (2015) [108] (*n* = 181) 2 y	Australia Mean age (SD): I: 74.2 (2.8) y C: 74.3 (2.6) y 100% female NR Postmenopausal	3-d weighed food record	I: mean (SD): 76 (18) g/d; 1.2 (0.3) g/kg/d C: mean (SD): 76 (16) g/d; 1.1 (0.3) g/kg/d	I: mean (SD): 15.4 (5.3) kg C: mean (SD): 16.1 (7.2) kg	I: mean (SEM) (change at 2 y): 3.36 (0.68) kg C: mean (SEM) (change at 2 y): 3.17 (0.80) kg	No difference	Insufficient
Leg extensor strength I: protein advice (advised to increase protein intake to ≥1.2 g/kg aBW/d) vs. C: control (no advice to increase protein consumption)	Reinders, (2022) [92] (*n* = 187) 6 mo	Finland, Netherlands Mean age (SD): I: 75.9 (5.0) y C: 75.0 (4.4) y I: 52.1% female C: 54.9% female NR NR	3-d food diary and 24-h food dietary recall questionnaire	I: mean (SD): 60.4 (1.3) g/d; 0.82 (0.01) g/kg aBW/d C: mean (SD): 60.5 (1.2) g/d; 0.82 (0.01) g/kg aBW/d	I: mean (SE): 309.4 (14.5) N C: mean (SE): 311.4 (12.9) N	I: mean (SE): 326.1 (14.2) N C: mean (SE): 295.5 (12.4) N	Found benefit	Insufficient
Sum 1-RM strength I: weight loss plus whey protein supplement (hypocaloric diet with increased protein intake 1.2 g/kg/d) vs. C: weight loss plus recommended protein (hypocaloric diet with 0.8 g/kg/d protein)	Smith, (2018) [101] (*n* = 52) 6 mo	United States NR 100% female 100% obese Postmenopausal	1-wk food record	NR	I: mean (SEM): 170 (6) kg C: mean (SEM): 163 (6) kg	I: mean (SEM): 173 (6) kg C: mean (SEM): 164 (6) kg	No difference	Insufficient
Sum knee extension peak torque I: weight loss plus whey protein supplement (hypocaloric diet with increased protein intake 1.2 g/kg/d) vs. C: weight loss plus recommended protein (hypocaloric diet with 0.8 g/kg/d protein)	Smith, (2018) [101] (*n* = 52) 6 mo	United States NR 100% female 100% obese Postmenopausal	1-wk food record	NR	I: mean (SEM): 326 (14) Nm C: mean (SEM): 305 (13) Nm	I: mean (SEM): 309 (13) Nm C: mean (SEM): 303 (13) Nm	No difference	Insufficient
Sum knee flexion peak torque I: weight loss plus whey protein supplement (hypocaloric diet with increased protein intake 1.2 g/kg/d) vs. C: weight loss plus recommended protein (hypocaloric diet with 0.8 g/kg/d protein)	Smith, (2018) [101] (*n* = 52) 6 mo	United States NR 100% female 100% obese Postmenopausal	1-wk food record	NR	I: mean (SEM): 188 (7) Nm C: mean (SEM): 178 (7) Nm	I: mean (SEM): 183 (6) Nm C: mean (SEM): 177 (7) Nm	No difference	Insufficient
Chair stand I1: whey protein (whey protein blended supplement), I2: soy protein (soy protein blended supplement), I3: whey-soy protein group (1:1 ratio of whey and soy blended supplement) vs. C: control (no supplementation	Li, (2021) [75] (*n* = 123) 6 mo	China Mean age (SD): I1: 71 (4) y I2: 69 (4) y I3: 70 (4) y C: 71 (4) y I1: 48.4% female I2: 51.6% female I3: 45.2% female C: 56.7% female NR NR	Food frequency questionnaire	I1: mean (SD): 62.7 (20.7) g/d; 1.14 (0.36) g/kg/d I2: mean (SD): 59.6 (19.1) g/d; 1.11 (0.33) g/kg/d I3: mean (SD): 61.1 (19.1) g/d; 1.14 (0.37) g/kg/d C: mean (SD): 59.3 (18.8) g/d; 1.17 (0.30) g/kg/d	I1: mean (SD): 8.95 (1.54) s I2: mean (SD): 8.43 (1.63) s I3: mean (SD): 8.68 (1.37) s C: mean (SD): 8.32 (1.32) s	I1: mean (SD): 8.22 (1.48) s I2: mean (SD): 7.60 (1.71) s I3: mean (SD): 8.25 (1.36) s C: mean (SD): 9.72 (1.89) s	Found benefit	Insufficient

Abbreviations: aBW, adjusted body weight; BMI, body mass index; C, control; CI, confidence interval; FFM, fat-free mass; I, intervention; N, newtons; NA, not applicable; Nm, newton meter; NR, not reported; RCT, randomized controlled trial; RM, repetition maximum; SD, standard deviation; SE, standard error; SEM, standard error of the mean; SPPB, short physical performance battery; TUG, timed up-and-go.

1 Indicates statistical significance.

2 Strength of evidence was evaluated based on 5 designated domains outlined in the Methods section and was insufficient. The main reasons for this insufficient rating were that the evidence was derived from a single study, making it impossible to assess consistency, and in some instances, the outcome effect estimate was imprecise.

3 Baseline characteristics and follow-up information were presented for participants who completed the 52-wk intervention, but intention-to-treat evaluation was conducted for the full sample (*n* = 120).

#### Muscle mass

Nine RCTs reported muscle mass outcomes (Table 4) [32,45,51,65,75,92,101,107,108]. Muscle strength outcomes included total body lean mass, appendicular lean or skeletal mass, appendicular skeletal muscle index (ASMi), total body skeletal muscle mass, and fat-free mass. Of the 4 RCTs [32,65,75,101] that reported findings for protein intake on total body lean mass, 1 found a positive effect [75]. The remaining 3 RCTs reported no difference between the intervention and comparator [32,65,101]. Three RCTs [32,75,108] reported findings on appendicular lean, or skeletal mass, of which 1 RCT reported a maintained appendicular lean mass for the intervention groups and a reduction in the control group [75]. However, the other 2 reported no difference between the intervention and comparator groups [32,108]. Two RCTs reported findings on ASMi defined as appendicular mass scaled to height squared (kg/m^2^) [75,108]. Of these, 1 reported a maintained ASMi for the intervention group and a reduction in the control group [75], and 1 found no difference in ASMi between groups [108]. One RCT reported a positive effect of protein intake on total body skeletal muscle mass between the intervention and comparator [51]. Four studies reported findings on fat-free mass and found no difference between the interventions and comparators [45,92,101,107].

#### Physical performance

Five RCTs reported physical performance outcomes (Table 4) [32,45,75,92,108]. Physical performance outcomes included timed up-and-go, 4 m walk gait speed, 400 m walk speed, and short physical performance battery (SPPB). One RCT reported no difference in timed up-and-go between the intervention and comparator [108]. One RCT found maintained a 4 m gait speed for the intervention groups and a reduction in the control group [75]. Three RCTs [32,45,92] reported findings on 400 m walk speed, and 1 RCT found a positive effect of protein intake on 400 m walk speed [92]. The remaining 2 RCTs found no difference between the interventions and comparator groups [32,45]. Four RCTs [32,45,75,92] reported findings on SPPB, of which 1 found a positive effect of protein intake on SPPB [75]. The remaining 3 RCTs found no difference between the interventions and comparators [32,45,92].

#### Muscle strength

Six RCTs reported muscle strength outcomes (Table 4) [32,45,75,92,101,108]. Muscle strength outcomes included handgrip strength, -1-repetition maximum (RM) leg press, knee flexor strength, leg extensor strength, sum 1-RM strength, sum knee extension peak torque, and sum knee flexion peak torque, and chair stand test. Of 5 RCTs [32,45,75,92,108] that reported findings on protein intake and handgrip strength, 1 found a positive effect [45], and the other 4 RCTs found no difference between the interventions and comparators [32,75,92,108]. One RCT reported no difference in 1-RM for leg press between the intervention and comparator [32]. Another RCT found no difference in knee flexor strength between the intervention and comparator [108]. Three RCTs reported findings on leg extensor strength using 1-RM leg extension, knee extensor strength, and leg extension strength measures each [32,92,108]. One RCT found no difference in 1-RM leg extension between the intervention and comparator [32]. Another RCT found no difference in knee extensor strength between the intervention and comparator [108]. However, 1 RCT found a positive effect of protein intake on leg extension strength [92]. One RCT reported findings on sum 1-RM strength, sum knee extension peak torque, and sum knee flexion peak torque, and no difference was found between the intervention and comparator [101]. One RCT found a positive effect of protein intake on the chair stand test [75].

## Discussion

### Principal findings

Our review sought to assess evidence from 2000 onwards regarding the association between dietary protein intake and the risks of bone disease, kidney disease, and sarcopenia. To achieve this, we focused on identifying and synthesizing data from studies rated as having low to moderate RoB. Overall, the evidence was insufficient, with few studies rated as low to moderate RoB. Research on children and adolescents was particularly sparse. Our review found mixed findings from a single study on the association between dietary protein intake and bone health in children and adolescents, examining bone turnover marker (OC), BMD, content, and BA of the lumbar spine. Studies on adult bone disease yielded inconsistent results, with some reporting no difference and others beneficial effects on bone turnover markers (OC, C-terminal peptide of collagen, tartrate-resistant alkaline phosphatase, bone alkaline phosphatase, and procollagen type 1 N-terminal propeptide), BMD of the lumbar spine, total hip, femoral neck, and total body BMD and BMC. The association between dietary protein intake and kidney disease risk was informed by a single study that found no significant effects on kidney function as measured by creatinine clearance. The assessment of sarcopenia risk also showed inconsistent findings concerning muscle mass, physical performance, and muscle strength. Additionally, studies used intermediate markers for disease risk assessment rather than directly investigating chronic conditions, complicating the determination of dietary protein intake’s impact on these health outcomes.

### Findings in the context of the literature

For bone disease, the findings of our review on bone turnover markers in adults align with previous studies by Wallace and Frankenfeld [111], Groenendijk et al. [112], and Tsagari [113], which found no effect of dietary protein intake on overall turnover markers, bone formation markers, or bone resorption markers. Regarding BMD, our results are consistent with Darling et al. [114], who found no association between protein intake and lumbar spine BMD, and Wallace and Frankenfeld [111], who reported inconsistent findings for femoral neck BMD. Notably, Darling et al. [114] included studies regardless of RoB, whereas Wallace and Frankenfeld [111] conducted qualitative evaluations without bias assessment. Similar to our findings, Darling et al. [114] and Tsagari [113] observed no effect of dietary protein intake on total body BMD but did not focus on studies with high methodological rigor. For children and adolescents, the single study included in our review showed mixed effects of dietary protein intake on bone health outcomes, including bone turnover markers, BMD, BMC, and BA of the lumbar spine. We found no previous reviews with comparable findings for this age group.

Our review examined the relationship between dietary protein intake and kidney disease, drawing from a single study of healthy adults that found no significant effects on kidney function as measured by creatinine clearance. No prior reviews reported similar findings.

For sarcopenia, the findings of our review were consistent with those of Hanach et al. [115], who reported no effects of dietary protein intake on muscle strength determined by 1-RM leg press and inconsistent results for physical performance evaluated by SPPB. Hanach et al. [115] included all studies regardless of RoB and presented findings qualitatively. Our review’s results on the association between dietary protein intake and appendicular lean mass/skeletal muscle mass reflected the inconsistent findings reported by Yaeghashi et al. [116]. However, our review focused on RCTs and prospective cohort studies, whereas Yaeghashi et al. [116] primarily examined cross-sectional studies, which are strongly challenged to show causal relationships.

### Strengths and limitations

Our systematic review had several strengths, including a unique emphasis on multiple chronic diseases. Additionally, our review is notable for examining the relationship between dietary protein intake and bone disease risk in children and adolescents. However, our exclusion of pre-2000 studies might have omitted important foundational research. However, given the continuous advancement in research methodologies, it is reasonable to assume that earlier studies may have faced greater challenges in rigor compared to more recent ones. Therefore, this exclusion is unlikely to have significantly impacted our findings. Further, by focusing only on studies rated low to moderate RoB, we limited the size of our body of evidence; however, including high RoB studies would have lessened the robustness of our findings and left SoE unchanged as we would have traded gains in the consistency domain for greater study limitations and likely lower precision.

Our review identified several limitations in the evidence base. Many studies relied on intermediate markers for bone and kidney disease and sarcopenia, which may not fully reflect the presence and progression of these chronic diseases. Most studies primarily focused on postmenopausal females due to their increased risk of bone disease and sarcopenia, which limits the generalizability of the findings. Extrapolations to other subpopulations should, therefore, be made with caution. Future research should include older males to enhance the generalizability of findings. The baseline diets generally met or exceeded protein recommendations, meaning results could differ if low or very-high protein intake populations were included. Many studies compared noninterventional protein intakes (e.g., 0.8 g/kg/d, similar to the current RDA) against higher intakes (1.2–1.7 g/kg/d), potentially overlooking a plateau effect. Studies did not address the broader implications of protein quality, other key nutrients, overall diet quality, and dietary patterns, which may influence the effect of dietary protein on health [[117], [118], [119]].

Additionally, studies did not report sarcopenia as an endpoint outcome. Despite the growing prevalence of sarcopenia in older adults, no universally accepted diagnostic criterion exists. Recent efforts have produced 2 notable definitions: 1 from the European Working Group on Sarcopenia in Older People’s Second Meeting [13] and another by the Sarcopenia Definition and Outcomes Consortium [120]. Establishing a consensus on the definition of sarcopenia is crucial for advancing research in this area.

Conducting nutrition research is complex, with several challenges impacting both study quality and RoB [121]. “Quality” refers to how well research adheres to its design, whereas “risk of bias” focuses on potential systematic errors. Our review rated many prospective cohort studies as high RoB due to unreported follow-up protein intake and high dropout rates, leading to potential misclassification and attrition biases. These biases would persist regardless of the specific RoB tool applied, including tools developed specifically for nutrition studies, such as the Nutrition QUality Evaluation Strengthening Tools [122] and NutriGrade [123]. To address these issues, regular follow-up assessments, consistent dietary data collection, leveraging technology for data submission, and transparent communication are recommended. Despite focusing on high-quality studies, predominantly RCTs, we encountered challenges. Some RCTs exhibited a high RoB due to attrition, where significant participant loss may have skewed results, compromising validity, statistical power, and confounder balance. To mitigate this, studies should incorporate regular quality control checks and include dropout participants in analyses via an intention-to-treat approach.

In conclusion, studies conducted since 2000 on the association between dietary protein and the risks of bone disease, kidney disease, and sarcopenia have yielded unclear yet potentially significant findings. Ambiguities arise from study limitations, lack of research on vital populations like children, varying protein intake levels, inconsistent outcome measures, and the absence of sarcopenia as a study endpoint. This underscores the need for a consensus definition of sarcopenia and more comprehensive, high-quality, long-term research to strengthen the evidence base essential for assessing dietary protein's impact on these chronic conditions.

## Author contributions

The authors’ responsibilities were as follows – TL, SB, HIA: wrote the manuscript; TL, SB, HIA, HY, AGH, RP, CK, AMC: screened, extracted data, and/or assessed risk of bias independently; AMC: searched databases; TL, SB, KMB, JLS, LT, LMS, KMHG, TH, AK, JS, MB: designed the protocol and provided input to the evidence synthesis, discussion, and conclusion statements; SD, TL: evaluated literature set for potential statistical analysis and provided input on the strength of evidence; MB, TL: responsible for the final content of the manuscript; and all authors: read and approved the final manuscript.

## Data availability

Data described in this systematic review is publicly and freely available without restriction in the systematic review data repository online system (http://srdr.ahrq.gov) and can be found at https://srdrplus.ahrq.gov/public_data?id=4625&type=project.

## Funding

The USDA and the Office of the Assistant Secretary for Health (OASH) requested this review, on behalf of the Joint United States-Canadian Dietary Reference Intake working group, from the evidence-based Practice Center program at the Agency for Healthcare Research and Quality (AHRQ), United States Department of Health and Human Services (HHS). AHRQ assigned this report to the Minnesota Evidence-based Practice Center (contract number 75Q80120D00008, task order number 75Q80123F32009). The authors of this document are responsible for its content. The content does not necessarily represent the official views of or imply endorsement by AHRQ or HHS. USDA, OASH, AHRQ, and HHS did not participate in the study design, data collection, analysis, interpretation, or report writing, and they imposed no restrictions on publication submission.

## Conflict of interest

The authors report no conflicts of interest.
