# Supplementary material for: The Effect of Protein Intake on Bone Disease, Kidney Disease, and Sarcopenia: A Systematic Review
Source: Curr Dev Nutr. 2025 Jan 21;9(3):104546. doi: 10.1016/j.cdnut.2025.104546 (PMC11894306; doi:10.1016/j.cdnut.2025.104546)
Supplement: Multimedia component 1 [file mmc1.docx]

Supplementary Methods: Search Strategy and Data Sources

Search Details and Sources

The search strategy was designed and conducted by an experienced systematic review Librarian with input from the investigators. Another Librarian peer reviewed the draft MEDLINE search strategy using the PRESS Checklist. The MEDLINE search included a combination of relevant keywords and MeSH search terms, the search was translated in each database’s specified controlled vocabulary. To find additional relevant studies, included studies from relevant systematic reviews were manually screened. We applied the following limits or filters to the database searches:

- Date. Investigators considered a literature search starting in 2000 sufficient for the purpose of this review.
- Language. Publications were excluded if they were written in a language other than English. This was due to resource constraints.
- Publication status. We searched for published studies.
- Human or organism. The search was limited to human studies.
- Study design. The search was restricted to randomized controlled trials and observational cohort studies.
- Filters. For Embase (Ovid), we created a modified filter based on a EMBASE RCT filter for Ovid. Reference: ISSG Search Filter Resource [Internet]. Glanville J, Lefebvre C, Manson P, Robinson S, Brbre I and Woods L, editors. York (UK): The InterTASC Information Specialists' Sub-Group; 2006 [updated 18 February 2024; cited 18 February 2024]. Available from https://sites.google.com/a/york.ac.uk/issg-search-filters-resource/home
- Filters. For Scopus, we created a modified RCT filter based on a CADTH search filter All Clinical Trials - Scopus. Reference: CADTH Search Filters Database. Ottawa: CADTH; 2023: https://searchfilters.cadth.ca/link/106. Accessed 2023-12-05.
- Filters: We created a modified filter based on a CADTH search filter to remove Embase and MEDLINE records in Scopus. Reference: Scopus NOT Medline/PubMed NOT Embase - Scopus. In: CADTH Search Filters Database. Ottawa: CADTH; 2024: https://searchfilters.cadth.ca/link/97. Accessed 2024-03-08. ([https://searchfilters.cadth.ca/link/97](https://searchfilters.cadth.ca/link/97%20Accessed%202023-12-05) )

We conducted a comprehensive literature search in May 2023, January 2024, March 2024, and May 2024. We searched the following databases:

- MEDLINE (Ovid) and Epub Ahead of Print, In-Process & Other Non-Indexed Citations and Daily Date searched May 31, 2023, January 17, 2024, March 26, 2024, and May 6, 2024
- Embase (Ovid) Date searched: May 31, 2023, January 17, 2024, March 26, 2024, and May 6, 2024
- Agricola (Ovid) Date searched: May 31, 2023, January 17, 2024, March 26, 2024, and May 6, 2024
- Scopus (Elsevier) Date serached: May 31, 2023, January 17, 2024, March 26, 2024, and May 6, 2024

Ovid MEDLINE(R) ALL <1946 to May 5, 2024>

1 animal proteins, dietary/ or dietary proteins/ or egg proteins, dietary/ or fish proteins, dietary/ or fruit proteins/ or grain proteins/ or meat proteins/ or milk proteins/ or nut proteins/ or plant proteins, dietary/ or pea proteins/ or poultry proteins/ or shellfish proteins/ or Soybean Proteins/ or whey proteins/ or Diet, High-Protein/ or diet, high-protein low-carbohydrate/ or (protein? adj3 (ate or animal? or bean? or beef or cheese? or consume* or consumption or content or dairy or diet* or eat or eating or egg? or fish or food or foods or fruit? or goat or grain? or high or increase* or intake* or lacto-vegetarian or lamb or legume? or lentils or macronutrient? or meat? or milk or miso or nut? or nutrition* or nutrient* or pea or peas or pescatarian or pescavegan or plant? or poultry or pork or recommend* or seed? or shellfish? or soy? or soybean? or supplement* or tofu or tempeh or veal or vegan or vegetable? or vegetarian or whey or yog?urt or yolk?)).ti,ab.

2 "Bone and Bones"/ or Bone Density/ or bone diseases/ or bone diseases, metabolic/ or bone demineralization, pathologic/ or bone resorption/ or Fractures, Bone/ or (bone disease? or bone densit* or bone demineralization or bone health or bone mass or bone mineral or bone resorption or bone fracture* or osteoporosis or osteopenia).ti,ab.

3 kidney calculi/ or Kidney Diseases/ or kidney failure, chronic/ or nephrolithiasis/ or renal insufficiency/ or renal insufficiency, chronic/ or ureterolithiasis/ or ureteral calculi/ or (chronic kidney failure or kidney calculi or kidney disease? or kidney function or kidney insufficiency or kidney stone? or nephrolithiasis or ureteral calculi or ureteral stone? or ureterolithiasis or renal calculi or renal disease? or renal function or renal insufficiency).ti,ab.

4 Muscular Atrophy/ or exp Muscle Strength/ or Muscle Weakness/ or sarcopenia/ or (muscle adj3 (atrophy or loss or mass or strength or wasting or weak* or sarcopenia)).ti,ab.

5 or/2-4

6 1 and 5

7 (randomized controlled trial or controlled clinical trial).pt. or randomi?ed.ti,ab. or placebo.ti,ab. or randomly.ab. or trial.ab. or groups.ab.

8 allocated.ti,ab,hw.

9 ((singl* or doubl* or triple) adj (blind* or dumm* or mask*)).ti,ab,hw,kf.

10 ((equivalence or superiority or non-inferiority or noninferiority) adj3 (study or studies or trial*)).ti,ab,hw,kf.

11 (Nonrandom* or non random* or non-random* or quasi-random* or quasirandom*).ti,ab,hw,kf.

12 or/7-11

13 case-control studies/ or clinical trial/ or cohort studies/ or controlled before-after studies/ or cross-over studies/ or pragmatic clinical trial/ or prospective studies/ or (before-after or between group* or clinical study or clinical trial or crossover design or cross-over design or crossover study or cross-over study or nested case-control* or prospectiv* or quasi-experiment*).mp.

14 Cohort analy*.tw.

15 (Follow up adj (study or studies)).tw.

16 (observational adj (study or studies)).tw.

17 or/13-16

18 12 or 17

19 6 and 18

20 limit 19 to (english language and yr="2000 -Current")

21 case reports/ or comment/ or editorial/ or letter/

22 20 not 21

23 Animals/ not (Animals/ and Humans/)

24 22 not 23

Embase <1974 to 2024 week 18>

1 animal protein/ or avian protein/ or fish protein/ or meat protein/ or milk protein/ or pea protein/ or plant protein/ or protein diet/ or protein intake/ or shellfish protein/ or soybean protein/ or whey protein/ or high-protein low-carbohydrate diet/ or (protein? adj3 (ate or animal? or bean? or beef or cheese? or consume* or consumption or content or dairy or diet* or eat or eating or egg? or fish or food or foods or fruit? or goat or grain? or high or increase* or intake* or lacto-vegetarian or lamb or legume? or lentils or macronutrient? or meat? or milk or miso or nut? or nutrition* or nutrient* or pea or peas or pescatarian or pescavegan or plant? or pork or poultry or recommend* or seed? or shellfish? or soy? or soybean? or supplement* or tofu or tempeh or veal or vegan or vegetable? or vegetarian or whey or yog?urt or yolk?)).ti,ab.

2 bone/ or bone density/ or bone disease/ or demineralization/ or fracture/ or metabolic bone disease/ or osteolysis/ or (bone disease? or bone densit* or bone demineralization or bone health or bone mass or bone mineral or bone resorption or bone fracture* or osteolysis or osteoporosis or osteopenia).ti,ab.

3 kidney disease/ or "chronic kidney disease-mineral and bone disorder"/ or exp chronic kidney failure/ or nephrolithiasis/ or ureter stone/ or urolithiasis/ or (kidney disease? or kidney function or kidney insufficiency or kidney stone? or nephrolithiasis or ureter* calculi or ureter* stone? or ureterolithiasis or renal calculi or renal disease? or renal function or renal insufficiency).ti,ab.

4 muscle atrophy/ or muscle function/ or muscle strength/ or muscle weakness/ or sarcopenia/ or (muscle adj3 (atrophy or loss or mass or strength or wasting or weak*)).ti,ab. or sarcopenia.ti,ab.

5 or/2-4

6 1 and 5

7 controlled clinical trial/ or intermethod comparison/ or exp randomized controlled trial/ or randomization/ or (placebo or random*).ti,ab.

8 (compare or compared or comparison).ti.

9 ((evaluated or evaluate or evaluating or assessed or assess) and (compare or compared or comparing or comparison)).ab.

10 ((open adj label) or ((double or single or doubly or singly) adj (blind or blinded or blindly))).ti,ab. or double blind procedure/ or parallel group$1.ti,ab. or (crossover or cross over).ti,ab. or ((assign* or match or matched or allocation) adj5 (alternate or group? or intervention* or patient? or subject? or participant?)).ti,ab.

11 (assigned or allocated or (controlled adj7 (study or design or trial))).ti,ab.

12 human experiment/ or trial.ti.

13 or/7-12

14 6 and 13

15 case control study/ or clinical trial/ or clinical trial/ or cohort analysis/ or crossover procedure/ or pragmatic trial/ or (before-after or between group* or crossover design or cross over design or crossover procedure or crossover study or cross over study or nested case-control* or prospectiv* or quasi-experiment*).ti,ab.

16 Cohort analy*.tw.

17 (Follow up adj (study or studies)).tw.

18 (observational adj (study or studies)).tw.

19 or/15-18

20 13 or 19

21 6 and 20

22 ((rat or rats or mouse or mice or swine or porcine or murine or sheep or lambs or pigs or piglets or rabbit or rabbits or cat or cats or dog or dogs or cattle or bovine or monkey or monkeys).ti. and animal experiment/) or (Animal experiment/ not (human experiment/ or human/))

23 21 not 22

24 limit 23 to (english language and yr="2000 -Current")

25 (Book or Chapter or Conference Abstract or Conference Paper or Conference Review or Preprint).pt. or book/ or case report/ or editorial/ or letter/ or note/

26 24 not 25

AGRICOLA <1970 to May 2024>

1 animal source protein/ or dairy protein/ or egg source protein/ or high protein foods/ or legume protein/ or meat protein/ or exp high protein diet/ or exp plant source protein/ or soy protein/ or textured proteins/ or (protein? adj3 (ate or animal? or bean? or beef or consume* or consumption or content or dairy or diet* or eat or eating or egg? or fish or food or foods or fruit? or goat or grain? or high or increase* or intake* or lacto-vegetarian or lamb or legume? or lentil? or macronutrient? or meat? or milk or nut? or nutrition* or nutrient* or pea or peas or pescatarian or pescavegan or plant? or poultry or pork or recommend* or seafood or seed? or shellfish or soy? or soybean? or supplement* or tempeh or tofu or veal or vegan or vegetable? or vegetarian or whey or yog?urt or yolk?)).ti,ab.

2 bone diseases/ or bone density/ or bone fractures/ or bone health/ or bone resorption/ or osteopenia/ or osteoporosis/ or (bone disease? or bone densit* or bone demineralization or bone health or bone mass or bone resorption or fracture* or osteoporosis or osteopenia).ti,ab.

3 kidney diseases/ or renal calculi/ or ureteral calculi/ or (kidney disease? or kidney function or kidney stone? or nephrolithiasis or ureteral calculi or ureteral stone? or ureterolithiasis or renal calculi or renal disease? or renal function or renal insufficiency).ti,ab.

4 muscular atrophy/ or muscle strength/ or hand strength/ or sarcopenia/ or (muscle adj3 (atrophy or loss or mass or strength or wasting or weak* or sarcopenia)).ti,ab.

5 or/2-4

6 1 and 5

7 limit 6 to (english language and yr="2000 -Current")

8 clinical trials/ or cross-over studies/ or randomized clinical trials/ or (cross-over study or nonrandom* or non random* or non-random* or quasi-random* or quasirandom* or random* or placebo or trial or groups).ti,ab,hw.

9 allocated.ti,ab,hw.

10 ((singl* or doubl*) adj (blind* or dumm* or mask*)).ti,ab,hw.

11 ((equivalence or superiority or non-inferiority or noninferiority) adj3 (study or studies or trial*)).ti,ab,hw.

12 case-control studies/ or cohort studies/ or observational studies/ or prospective studies/

13 (observational adj (study or studies)).tw.

14 (Follow up adj (study or studies)).tw.

15 Cohort analy*.tw.

16 or/8-15

17 7 and 16

18 exp human nutrition/ or exp people/

19 17 and 18

Scopus (Elsevier)

( ( INDEXTERMS ( "Muscular Atrophy" ) OR INDEXTERMS ( "Muscle Strength" ) OR INDEXTERMS ( "Muscle Weakness" ) OR INDEXTERMS ( sarcopenia ) OR TITLE-ABS ( ( muscle OR muscular ) W/3 ( atrophy OR loss OR mass OR strength OR wasting OR weak* ) ) OR TITLE-ABS ( sarcopenia ) ) OR ( INDEXTERMS ( "kidney calculi" ) OR INDEXTERMS ( "Kidney Diseases" ) OR INDEXTERMS ( "kidney failure, chronic" ) OR INDEXTERMS ( nephrolithiasis ) OR INDEXTERMS ( "renal insufficiency" ) OR INDEXTERMS ( "renal insufficiency, chronic" ) OR INDEXTERMS ( ureterolithiasis ) OR INDEXTERMS ( "ureteral calculi" ) OR TITLE-ABS ( "chronic kidney failure" OR "kidney calculi" OR "kidney disease*" OR "kidney function" OR "kidney insufficiency" OR "kidney stone*" OR nephrolithiasis OR "ureteral calculi" OR "ureteral stone*" OR ureterolithiasis OR "renal calculi" OR "renal disease*" OR "renal function" OR "renal insufficiency" ) ) OR ( INDEXTERMS ( "Bone and Bones" ) OR INDEXTERMS ( "Bone Density" ) OR INDEXTERMS ( "bone diseases" ) OR INDEXTERMS ( "bone diseases, metabolic" ) OR INDEXTERMS ( "bone demineralization, pathologic" ) OR INDEXTERMS ( "bone resorption" ) OR INDEXTERMS ( "Fractures, Bone" ) OR TITLE-ABS ( "bone disease*" OR "bone densit*" OR "bone demineralization" OR "bone health" OR "bone mass" OR "bone mineral" OR "bone resorption" OR "bone fracture*" OR osteoporosis OR osteopenia ) ) ) AND ( INDEXTERMS ( "Dietary Proteins" ) OR INDEXTERMS ( "dairy proteins" ) OR INDEXTERMS ( "egg protein" ) OR INDEXTERMS ( "Diet, High-Protein" ) OR INDEXTERMS ( "diet, high-protein low-carbohydrate" ) OR INDEXTERMS ( "animal protein" ) OR INDEXTERMS ( "soy proteins" ) OR INDEXTERMS ( "plant proteins" ) OR TITLE-ABS ( ( protein* ) W/3 ( ate OR animal* OR bean* OR beef OR beverage* OR cheese OR consume* OR consumption OR content OR dairy OR diet* OR drink* OR eat OR eating OR egg* OR fish OR food OR foods OR fruit* OR goat OR grain* OR high OR increase* OR intake* OR lacto-vegetarian OR lamb OR legumes OR lentils OR macronutrient* OR meat* OR milk OR nut* OR nutrition* OR nutrient* OR pea OR peas OR pescatarian OR pescavegan OR plant* OR pork OR poultry OR recommend* OR seeds OR shellfish OR soy* OR soybean OR supplement* OR tempeh OR tofu OR veal OR vegan OR vegetable* OR vegetarian OR whey OR yogurt OR yolk* ) ) ) AND PUBYEAR > 1999 AND PUBYEAR < 2024 AND NOT INDEX ( medline ) AND NOT ( PMID ( 0* OR 1* OR 2* OR 3* OR 4* OR 5* OR 6* OR 7* OR 8* OR 9* ) ) AND NOT INDEX ( embase ) ) AND ( ( ( INDEXTERMS ( "clinical trial" ) OR INDEXTERMS ( "cross-over studies" ) OR INDEXTERMS ( "pragmatic clinical trial" ) OR INDEXTERMS ( "case-control studies" ) OR INDEXTERMS ( "cohort studies" ) OR INDEXTERMS ( "prospective studies" ) OR INDEXTERMS ( "controlled before-after studies" ) OR TITLE-ABS-KEY ( observational W/3 ( study OR studies OR design OR analysis OR analyses ) ) OR TITLE-ABS-KEY ( prospective W/7 ( study OR studies OR design OR analysis OR analyses ) ) OR TITLE-ABS-KEY ( "follow up" OR followup W/7 ( study OR studies OR design OR analysis OR analyses ) ) OR TITLE-ABS-KEY ( cohort AND analy* ) OR TITLE-ABS-KEY ( nested AND case AND control* ) OR TITLE-ABS-KEY ( quasi W/1 ( experiment OR experiments OR experimental ) ) OR TITLE-ABS-KEY ( cohort AND analy* ) ) OR ( TITLE-ABS-KEY ( random* OR sham OR placebo* ) OR TITLE-ABS-KEY ( ( singl* OR doubl* ) W/1 ( blind* OR dumm* OR mask* ) ) OR TITLE-ABS-KEY ( ( tripl* OR trebl* ) W/1 ( blind* OR dumm* OR mask* ) ) OR TITLE-ABS-KEY ( control* W/3 ( study OR studies OR trial* OR group* ) ) OR TITLE-ABS-KEY ( clinical W/3 ( study OR studies OR trial* ) ) OR TITLE-ABS-KEY ( nonrandom* OR "non random*" OR non-random* OR quasi-random* OR quasirandom* ) OR TITLE-ABS-KEY ( phase W/3 ( study OR studies OR trial* ) ) OR TITLE-ABS-KEY ( ( crossover OR cross-over ) W/3 ( study OR studies OR trial* ) ) OR TITLE-ABS-KEY ( ( multicent* OR multi-cent* ) W/3 ( study OR studies OR trial* ) ) OR TITLE-ABS ( allocated ) OR TITLE-ABS-KEY ( ( "open label" OR open-label ) W/5 ( study OR studies OR trial* ) ) OR TITLE-ABS-KEY ( ( equivalence OR superiority OR non-inferiority OR noninferiority ) W/3 ( study OR studies OR trial* ) ) OR TITLE-ABS-KEY ( "pragmatic study" OR "pragmatic studies" ) OR TITLE-ABS-KEY ( ( pragmatic OR practical ) W/3 trial* ) OR TITLE-ABS-KEY ( ( quasiexperimental OR quasi-experimental ) W/3 ( study OR studies OR trial* ) ) OR TITLE ( trial ) OR KEY ( trial ) ) ) OR ( TITLE-ABS-KEY ( observational W/3 ( study OR studies OR design OR analysis OR analyses ) ) OR TITLE-ABS-KEY ( prospective W/7 ( study OR studies OR design OR analysis OR analyses ) ) OR TITLE-ABS-KEY ( "follow up" OR followup W/7 ( study OR studies OR design OR analysis OR analyses ) ) OR TITLE-ABS-KEY ( cohort AND analy* ) OR TITLE-ABS-KEY ( nested AND case AND control* ) OR TITLE-ABS-KEY ( quasi W/1 ( experiment OR experiments OR experimental ) ) OR TITLE-ABS-KEY ( cohort AND analy* ) ) ) AND ( LIMIT-TO ( SRCTYPE , "j" ) ) AND ( LIMIT-TO ( DOCTYPE , "ar" ) ) AND ( LIMIT-TO ( LANGUAGE , "English" ) )

Supplementary Methods: Grading the Strength of Evidence for Major Comparisons and Outcomes

The strength of evidence (SoE) is the extent of our confidence in drawing a specific conclusion, and is based on causal inference criteria.

The overall SoE for comparisons and outcomes identified for Key Questions 1 – 3 were evaluated based on five required domains: 1) study limitations (risk of bias); 2) consistency (similarity of effect direction and size); 3) directness (single, direct link between intervention and outcome); 4) precision (degree of certainty around an estimate); and 5) reporting bias (1).

Based on study design and risk of bias, we rated study limitations as low, moderate, high, or very high. Consistency was rated as consistent, inconsistent, or unknown/not applicable (e.g., single study) based on whether intervention effects were similar in direction and magnitude, and statistical significance of all studies. Directness was rated as either direct or indirect based on the need for indirect comparisons when inference requires observations across studies (i.e., more than one step was needed to reach the conclusion). Precision was rated as precise or imprecise based on the degree of certainty surrounding each effect estimate or qualitative finding. An imprecise estimate is one for which the confidence interval is wide enough to include clinically distinct conclusions.

An outcome with an overall rating of “high strength of evidence” implies that the included contributing studies were randomized controlled trials with both a low risk of bias, and with consistent, direct, and precise domains. If we had found any outcome to have at least moderate or high strength of evidence, we would have evaluated reporting bias by the potential for publication bias, selective outcome reporting bias, and selective analysis reporting bias. We would have done this by comparing reported results with those mentioned in the methods section and an assessment of the grey literature to assess potentially unpublished studies. However, no findings rose to this level. Other factors considered in assessing strength of evidence included dose-response relationship, the presence of confounders, and strength of association.

Based on these factors, we rated the overall strength of evidence for each outcome as:

**High**: Very confident that estimate of effect lies close to true effect. Few or no deficiencies in body of evidence, findings are believed to be stable.

**Moderate**: Moderately confident that estimate of effect lies close to true effect. Some deficiencies in body of evidence; findings likely to be stable, but some doubt.

**Low**: Limited confidence that estimate of effect lies close to true effect; major or numerous deficiencies in body of evidence. Additional evidence necessary before concluding that findings are stable or that estimate of effect is close to true effect.

**Insufficient**: No evidence, unable to estimate an effect, or no confidence in estimate of effect. Available evidence or lack of evidence precludes judgment.

Notably, an assessment of insufficient evidence does not mean that the intervention is ineffective. Rather, it means that due to the uncertainty of the evidence, we could not draw meaningful conclusions about its effectiveness at this time.

Supplementary Methods: Assessing Methodological Risk of Bias of Individual Studies

Risk of bias is the extent to which the design and conduct of a study are unlikely to have prevented bias in the results.

We implemented the Cochrane Risk of Bias tool 2.0 parallel design version (2, 3) to assess risk of bias of parallel RCTs, as low risk, some concerns (moderate risk), or high risk for each of the following domains: 1) Bias arising from randomization process; 2) Bias due to deviations from intended interventions; 3) Bias due to missing outcome data; 4) Bias in measurement of the outcome; 5) Bias in selection of reported result. In addition, we used the Cochrane Risk of Bias tool 2.0 crossover design version(2) to assess risk of bias of crossover RCTs, as low risk, some concerns (moderate risk), or high risk for each of the following domains: 1) Bias arising from randomization process; 2) Bias from period and carryover effects; 3). Bias due to deviations from intended interventions; 4) Bias due to missing outcome data; 5) Bias in measurement of the outcome; 6) Bias in selection of reported result.

For observational studies (including prospective cohort studies with or without comparison group and nested case-control studies), risk of bias by outcomes were rated using the Risk Of Bias In Non-randomized Studies – of Exposure (ROBINS-E) tool (4) as low, moderate, serious, critical, or no information for each of the following domains: 1) Bias due to confounding; 2) Bias in selection of participants into the study; 3) Bias due exposure classification; 4) Bias due to deviations from intended interventions; 5) Bias due to missing data; 6) Bias in measurement of outcomes; 7) Bias in selection of the reported result; and an overall risk of bias judgment option low, moderate, high (serious) or very high (critical).

When using the ROBINS-E tool, we carried out our assessment using a two-step process for all eligible studies. We first assessed only domain 3 – 7. When at least one domain was assessed as high risk or very high risk of bias, we determined that a study had an overall risk of bias judgement of high risk or very high risk of bias (based on the ROBINS-E algorithm for reaching overall risk of bias judgement); and we decided that no further assessment was required using domains 1 – 2. When domains 3 – 7 were not assessed as high risk or very high risk of bias (i.e., where the domains were either low or moderate risk of bias), we decided to carry out further assessment using domains 1– 2. Given the number of eligible studies, we chose to use the two-step process to proceed with our ROBINS-E risk of bias assessment in a timely manner.

One reviewer independently assessed the risk of bias for eligible studies by outcome; a second investigator reviewed each risk of bias assessment. Investigators consulted to reconcile any discrepancies in the risk of bias assessments. For RCTs, we classified the overall risk of bias assessments for each study outcome as low risk, moderate risk, or high risk. For observational studies, we classified the overall risk of bias assessments for each study outcome as low, moderate, high (serious) or very high (critical).

We based overall risk of bias assessments on the collective risk of bias across components and confidence that the study results for given outcomes were believable given the study’s limitations. When determining the overall strength of evidence, we considered any quality issues pertinent to the specific outcomes of interest.

Supplementary Table 1: Inclusion and Exclusion Criteria by Population, Intervention, Comparator, Outcome, Timing, Setting/Study Design (PICOTS)

| Element | Inclusion | Exclusion |
| --- | --- | --- |
| Population KQ1 | - Participants who are healthy and/or have chronic diseases or chronic disease risk factors, including those with obesity. - Participants who are pregnant and lactating - Age of participants (at intervention or exposure):   - Infants, children, and adolescents (0-18 years)   - Adults (19-64 years)   - Older adults (65 years and older) | - Participants sample exclusively diagnosed with a disease or hospitalized or in a long-term care facility with an illness or injury - Participants who have already been diagnosed with bone disease - Participants with existing conditions that clearly are known to alter nutrient metabolism or requirements, or those being treated with medications that alter nutrient metabolism - Participant sample exclusively undernourished - Participant sample exclusively with a baseline diet deficient in protein (i.e below the recommended daily allowance of protein (RDA) per age) - Participant sample exclusively pre-term infant - Participant sample exclusively post-bariatric surgery subjects - Participant sample exclusively elite athletes - Non-human participants (e.g., animal studies, in-vitro models) |
| Population KQ2&3 | - Participants who are healthy and/or have chronic diseases or chronic disease risk factors, including those with obesity. - Participants who are pregnant and lactating - Age of participants (at intervention or exposure):   - Adults (19-64 years)   - Older adults (65 years and older) | - Participants sample exclusively diagnosed with a disease or hospitalized or in a long-term care facility with an illness or injury - Participants who have already been diagnosed with kidney disease and/or sarcopenia - Participants with existing conditions that clearly are known to alter nutrient metabolism or requirements, or those being treated with medications that alter nutrient metabolism - Participant sample exclusively undernourished - Participant sample exclusively with a baseline diet deficient in protein (i.e below the recommended dietary allowance of protein (RDA) per age) - Participant sample exclusively post-bariatric surgery subjects - Participant sample exclusively elite athletes - Non-human participants (e.g., animal studies, in-vitro models) |
| Interventions KQ1-3 | - Total dietary protein intake from food, beverages, and dietary supplements with or without energy restriction - Assessment of % AMDR for protein with or without the % from the other macronutrients (carbohydrate and fat) | - No specification on the amount of protein intake (e.g., only the type of protein or source of protein reported) - Protein intake via parenteral nutrition or intravenous nutrition support - Food products or dietary supplements not widely available to U.S. consumers - Protein intake evaluated with exercise |
| Comparison KQ1-3 | - Consumption of different levels of total dietary protein intake - No comparator | Comparison of different sources of protein (i.e., animal versus plant protein) without specification on the levels of total dietary protein intake |
| Outcomes KQ1 | Bone outcomes, including but not limited to:   - Osteoporosis - Osteopenia - Fracture - Bone mass including bone mineral density, bone mineral content etc. | No relevant exclusion criteria |
| Outcomes KQ2 | Kidney outcomes including but not limited to:   - Incidence of kidney stones or ureteral stones - Incidence of CKD (including evaluations from estimated glomerular filtration (eGFR) rate with or without a parameter for race) - Kidney insufficiency | No relevant exclusion criteria |
| Outcomes KQ3 | Aging associated sarcopenia (any definition) and its diagnostic indicators, including but not limited to:   - Muscle mass (such as skeletal muscle mass, lean body mass, and fat free mass) - Physical performance (such as Timed Up-and-Go [TUG], gait speed, and Short Physical Performance Battery [SPPB] etc.) - Muscle strength | No relevant exclusion criteria |
| Timing KQ1-3 | All duration and followup | No relevant exclusion criteria |
| Setting KQ1-3 | All settings | No relevant exclusion criteria |
| Study design KQ1-3 | - Randomized controlled trials (RCTs) - Non-randomized controlled trials (Non-RCTs), including quasi-experimental and controlled before-and-after studies - Prospective cohort studies with or without comparison group with appropriate analytic technique - Nested case-control studies | - Narrative reviews - Systematic reviews, meta-analyses, umbrella reviews, scoping reviews - Systematic reviews or meta-analyses that exclusively include cross-sectional and/or uncontrolled studies - Retrospective cohort studies - All other study designs |
| **Language KQ1-3** | English only (due to resource limitations) | Non-English publications |
| **Geographic**  **Location KQ1-3** | Locations with food products or dietary supplements widely available to U.S. consumers, including those rated high and very high on the Human Development Index | Locations with less than high HDI |
| **Study size KQ1-3** | Studies with N > 50 participants (for RCTs – 25 participants analyzed per study arm) | Studies with N < 50 participants (for RCTs – 25 participants analyzed per study arm), and without power calculation |
| **Publication date KQ1-3** | 2000 to present | Prior to 2000 |
| **Publication status KQ1-3** | Articles published in peer-reviewed journals | Articles that have not been peer reviewed and are not published in peer-reviewed journals (e.g., unpublished data, manuscripts, pre-prints, reports, abstracts, conference proceedings) |

**Abbreviations**: AMDR = Acceptable macronutrient distribution range; CKD = chronic kidney disease; eGFR = estimated glomerular filtration rate; GI = gastrointestinal; HDI = human development index; SPBB = Short Physical Performance Battery; KQ = key question; N = number; RCT = randomized controlled trial; U.S. = United States

Supplementary Table 2. Evidence table for Bone Disease Randomized Controlled Trials (Adults and Children and Adolescents)

| Study | Participants | Interventions/Exposure and Comparator (Content, administrator, and duration) | Intervention (s) (Methods of administration and assessment) | Outcome (Measures and methods of assessment) |
| --- | --- | --- | --- | --- |
| PMID: NR **Aoyagi 2010 (5)** Location/Country: Japan HDI: Very high Setting: Community dwelling Urban/ Rural: NR Study design: RCT (parallel) Funding source: Nonprofit **Risk of bias score: High** | Study of: Adults Total sample N: 79  **Intervention**: High Protein Experimental: 40 mg MBP Supplement  N: 44 % Female: 100% Mean Age (SD): 72 (4) y Race/ Ethnicity: Japanese  Menopausal status: Postmenopausal Obesity status: NR Mean BMI (SD): NR Income level: NR Education level: NR Mean physical activity level (SD): 17.5 (10.3) year-averaged duration of exercise Health status/ Comorbidities: No history of conditions affecting bone metabolism (e.g., ovariectomy, cancer, renal disease or rheumatoid arthritis). Medication use: No current treatment with hormonal preparations (e.g., estrogens) or other drugs (e.g., bisphosphonates) likely to influence bone health. Supplement use: NR Pregnant or lactating: NA  **Comparator:** Normal Protein N: 35 % Female: 100% Mean Age (SD): 72 (6) y Race/ Ethnicity: Japanese  Menopausal status: Postmenopausal Obesity status: NR Mean BMI (SD): NR Income level: NR Education level: NR Mean physical activity level (SD): 14.5 (9.2) year-averaged duration of exercise Health status/Co-morbidities: No history of conditions affecting bone metabolism (e.g., ovariectomy, cancer, renal disease or rheumatoid arthritis). Medication use: No current treatment with hormonal preparations (e.g., estrogens) or other drugs (e.g., bisphosphonates) likely to influence bone health. Supplement use: NR Pregnant or lactating: NA | **Intervention:** High Protein Intended Protein Amount: 40 mg of milk basic protein  Carbohydrate: NR Fat: NR  Baseline Protein Amount Mean (SD): 71.9 (17.8) g/d Carbohydrate Mean (SD): NR Fat Mean (SD): NR  Actual Protein Amount at the end of the study Mean (SD): NR Carbohydrate Mean (SD): NR Fat Mean (SD): NR  Dietary Protein Intake Compliance (%): 88%  Protein type/source: Animal; milk basic protein  Energy balance status: Eucaloric  **Comparator**: Normal Protein  Intended Protein Amount: NR Carbohydrate: NR Fat: NR  Baseline Protein Amount Mean (SD): 74.1 (19.6) g/d Carbohydrate Mean (SD): NR Fat Mean (SD): NR  Actual Protein Amount at the end of the study Mean (SD): NR Carbohydrate Mean (SD): NR Fat Mean (SD): NR  Dietary Protein Intake Compliance (%): 40%  Protein type/source: Mixed  Energy balance status: Eucaloric  Study duration: 12 months | **Intervention:** High Protein **How protein was administered:** Participants drank 1 bottle (50 mL) a day of 40mg milk basic protein (Mainichi Hone Kea MBP® Snow Brand Milk Products Co., Ltd., Shinjuku, Tokyo, Japan)  **Protein Assessment Method:** Baseline protein was determined through a 1-wk retrospective dietary questionnaire **Dietary Protein Intake Compliance:** Participants kept diaries and returned empty bottles at their monthly laboratory visits.   Actual Protein Amount was not calculated, though empty bottles returned by participants were counted and food diaries were kept by participants to ensure compliance with intended treatment.  **Comparator:** Normal Protein  **How protein was administered:** Participants were instructed to keep their dietary habits the same  **Protein Assessment Method:** Same as above  **Dietary Protein Intake Compliance:** Same as above | **Bone Turnover Marker (Overall Turnover) -** Osteocalcin  Measure/Method of Assessment: Blood assays  **Bone Formation Marker -** Bone specific alkaline phosphatase   Measure/Method of Assessment: Blood and urine assays  **Bone Resorption Marker -** Urinary excretion of deoxypyridinoline  Measure/Method of Assessment: Blood and urine assays  **Bone Resorption Marker –** NTx (N-teleopeptides of type I collagen)  Measure/Method of Assessment: Blood assay   **BMD of the Appendicular Skeleton -** Bone mineral density (forearm, total) Measure/Method of Assessment: Peripheral DXA, using a bone densitometer ([DTX-200, Osteometer MediTEch, Inc.]). |
| PMID: 15727682 **Arjmandi 2005 (6)** Location/Country: USA HDI: Very high Setting: Community dwelling Urban/ Rural: NR Study design: RCT (parallel)  Funding source: Industry, state agency **Risk of bias score: High** | Study of: Adults Total sample N: 62  **Intervention:** High Protein N: 35 % Female: 100% Mean Age (SE): 53 (6) y Race/ Ethnicity: NR Menopausal status: Postmenopausal Obesity status: NR Mean BMI (SE): 28.6 (0.9) kg/m^2^ Income level: NR Education level: NR Physical activity level: NR Health status/ Comorbidities: Women with cancer, liver disease, hypo- or hyperthyroidism, gastrointestinal disorders, insulin-dependent diabetes mellitus, pelvic inflammatory disease, and endometrial polyps were excluded from the study Medication use: Study participants were not on any prescription medication. Supplement use: Herbal supplement was exclusion criteria Pregnant or lactating: NA  **Comparator:** Normal Protein N: 27 % Female: 100% Mean Age (SE): 56 (5) y Race/ Ethnicity: NR Menopausal status: Postmenopausal Obesity status: NR Mean BMI (SE): 27.3 (1.0) kg/m^2^ Income level: NR Education level: NR Physical activity level: NR Health status/ Comorbidities: Women with cancer, liver disease, hypo- or hyperthyroidism, gastrointestinal disorders, insulin-dependent diabetes mellitus, pelvic inflammatory disease, and endometrial polyps were excluded from the study Medication use: Study participants were not on any prescription medication. Supplement use: Herbal supplement was exclusion criteria Pregnant or lactating: NA | **Intervention:** High Protein Intended Protein Amount: 25 g/d of supplement Carbohydrate: NR Fat: NR  Baseline Protein Amount Mean (SE): 75.8 (3.6) g/d Carbohydrate Mean (SE): 243 (12) g/d Fat Mean (SE): 62.5 (4.1) g/d  Actual Protein Amount at the end of the study Mean (SE): 87.3 (3.6) g/d Carbohydrate Mean (SE): 202 (12) g/d Fat Mean (SE): 57.0 (4.2) g/d  Dietary Protein Intake Compliance (%): NR  Protein type/source: Plant; soy products  Energy balance status: Eucaloric  **Comparator:** Normal Protein  Intended Protein Amount: NR Carbohydrate: NR Fat: NR  Baseline Protein Amount Mean (SE): 64.2 (4.1) g/d Carbohydrate Mean (SE): 207 (14) g/d Fat Mean (SE): 56.6 (4.8) g/d  Actual Protein Amount at the end of the study Mean (SE): 87.8 (4.1) g/d Carbohydrate Mean (SE): 247 (14) g/d Fat Mean (SE): 59.0 (4.8) g/d  Dietary Protein Intake Compliance (%): NR  Protein type/source: Mixed  Energy balance status: Eucaloric  Study duration: 1 y | **Intervention:** High Protein **How protein was administered:** Participants ate a test food of 25 g soy products (donated by DrSoy Nutrition Irvine, CA) in the form of a snack bar, drink mix, or cereal **Protein Assessment Method:** Baseline and end-of-study protein was obtained from a 1-wk food frequency questionnaire via interview by a registered dietitian. Actual protein was determined by analysis of customized calendars for participants to record amount of test food consumed.   **Dietary Protein Intake Compliance:** Participants recorded how much of each of the cereal, the snack bar, or the drink mix they consumed on a customized calendar. Participants also returned any unconsumed foods.  **Comparator:** Normal Protein  **How protein was administered:** Participants consumed comparative controls  **Protein Assessment Method:** Same as above  **Dietary Protein Intake Compliance:** Same as above | **Total Body BMD -** Bone mineral density (total body) Measure/Method of Assessment: DXA (Hologic QDR-4500C)  **BMD of the Axial Skeleton -** Bone mineral density (L1-L4 (lumbar spine)) Measure/Method of Assessment: DXA (Hologic QDR-4500C)  **BMD of the Appendicular Skeleton -** Bone mineral density (hip, total) Measure/Method of Assessment: DXA (Hologic QDR-4500C)  **Total Body BMC -** Bone mineral content (total body) Measure/Method of Assessment: DXA (Hologic QDR-4500C)  **BMC of the Axial Skeleton -** Bone mineral content (L1-L4 (lumbar spine)) Measure/Method of Assessment: DXA (Hologic QDR-4500C)  **BMC of the Appendicular Skeleton -** Bone mineral content (hip, total) Measure/Method of Assessment: DXA (Hologic QDR-4500C)  **Bone Turnover Marker (Overall Turnover) -** Osteocalcin Measure/Method of Assessment: Blood sample (serum)  **Bone Formation Marker -** Bone specific alkaline phosphatase Measure/Method of Assessment: Blood sample (serum)  **Bone Resorption Marker -** Urinary excretion of deoxypyridinoline  Measure/Method of Assessment: Blood sample (serum) |
| PMID: 22357739 **Bonjour 2012 (7)** Location/Country: France HDI: Very high Setting: Community dwelling Urban/ Rural: NR Study design: RCT (parallel) Funding source: Industry  **Risk of bias score: Moderate** | Study of: Adults Total sample N: 71  **Intervention**: Treated group N: 36 % Female: 100% Mean Age (SD): 57.1 (3.9) y Race/ Ethnicity: NR  Menopausal status: Postmenopausal Obesity status: NR Mean BMI (SD): 23.1 (2.2) kg/m^2^ Income level: NR Education level: NR Physical activity level: NR Health status/ Comorbidities: Exclusion criteria: disorders influencing calcium-phosphate and/or bone metabolism, such as hyperparathyroidism, Paget disease, or chronic condition requiring cortisone therapy. Medication use: Inclusion criteria: no antiosteoporotic medication, such as bisphosphonates, raloxifen, strontium ranelate, teriparatide, and/or denosumab Supplement use: exclusion criteria: use of calcium and or vitamin D supplement, taken as pharmaceutical preparation or fortified foods, during the preceding 6 months Pregnant or lactating: NA  **Comparator:** Usual diet N: 35 % Female: 100 Mean Age (SD): 56.1 (3.9) y Race/ Ethnicity: NR Menopausal status: Postmenopausal Obesity status: NR Mean BMI (SD): 22.9 (2.5) kg/m^2^ Income level: NR Education level: NR Physical activity level: NR Health status/ Comorbidities: Exclusion criteria: disorders influencing calcium-phosphate and/or bone metabolism, such as hyperparathyroidism, Paget disease, or chronic condition requiring cortisone therapy. Medication use: Inclusion criteria: no antiosteoporotic medication, such as bisphosphonates, raloxifen, strontium ranelate, teriparatide, and/or denosumab Supplement use: exclusion criteria: use of calcium and or vitamin D supplement, taken as pharmaceutical preparation or fortified foods, during the preceding 6 months Pregnant or lactating: NA | **Intervention:** Treated group Intended Protein Amount: Test food supplement: 13.8 g protein Carbohydrate: NR Fat: NR  Baseline Protein Amount Mean (SD): 72 (17) g/d Carbohydrate Mean (SD): 193 (73) g/d Fat Mean (SD): 79 (19) g/d  Actual Protein Amount at the end of the study (change) Mean (SD): 11.4 (18.5) g/d Carbohydrate (change) Mean (SD): -11.3 (61.4) g/d Fat (change) Mean (SD): 9.4 (24.4) g/d  Dietary Protein Intake Compliance (%): 100%  Protein type/source: Animal; skimmed-milk, soft, plain cheese  Energy balance status: Eucaloric  **Comparator:** Usual diet  Intended Protein Amount: NR Carbohydrate: NR Fat: NR  Baseline Protein Amount Mean (SD): 199 (79) g/d Carbohydrate Mean (SD): 199 (79) g/d Fat Mean (SD): 78 (28) g/d  Actual Protein Amount at the end of the study (change) Mean (SD): 0.9 (16.5) g/d  Carbohydrate (change) Mean (SD): -11.6 (61.4) g/d Fat (change) Mean (SD): 5.5 (22.3) g/d  Dietary Protein Intake Compliance (%): 100%  Protein type/source: Mixed  Energy balance status: Eucaloric   Study duration: 6 weeks | **Intervention:** Treated group **How protein was administered:** Dairy (skimmed-milk, soft, plain cheese fortified with vitamin D and calcium) test food given to participants, 2 servings, 100 g each, once daily. **Protein Assessment Method:** Baseline protein, intermediary, and post protein amounts were derived from a dietary follow-up questionnaire **Dietary Protein Intake Compliance:** Participants completed a self-rating diary, which had to be completed every day.   **Comparator:** Usual diet  **How protein was administered:** Participants advised to maintain their usual diet   **Protein Assessment Method:** Same as above **Dietary Protein Intake Compliance:** Same as above | **Bone Resorption Marker -** CTX (carboxy terminal crosslinked telopeptide of type I collagen) Measure/Method of Assessment: Blood sample serum  **Bone Resorption Marker -** TRAP (5b, tartrate resistant acid phosphatase, isoform 5) Measure/Method of Assessment: Blood sample serum  **Bone Turnover Marker (Overall Turnover) -** Osteoclacin  Measure/Method of Assessment: Blood sample serum  **Bone Formation Marker –** Bone specific alkaline phosphatase Measure/Method of Assessment: Blood sample serum  **Bone Formation Marker –** P1NP (Procollagen type 1 N-terminal propeptide) Measure/Method of Assessment: Blood sample serum |
| PMID: 24047916 **Jesudason 2013^1^ (8)** Location/Country: Australia HDI: Very high Setting: Community dwelling Urban/Rural: NR Study Design: RCT (parallel)  Funding source: Government **Risk of bias score: High** | Study of: Adults Total sample N: 323   **Intervention:** High Protein N: 164 % Female: 100% Mean Age (SE): 59.5 (0.4) y Race/ Ethnicity: NR Menopausal status: Postmenopausal Obesity status: Obese Mean BMI (SE): 34.0 (0.4) kg/m^2^ Income level: NR Education level: NR Physical activity level: NR Health status/ Comorbidities: Subjects with parathyroid disease, a vitamin D concentration, 60 nmol/L with secondary hyperparathyroidism, or unstable metabolic, cardiac, gastrointestinal, renal, or other significant disease, including malignancies, were excluded Medication use: Women were ineligible if they were taking hormone-replacement therapy, bisphosphonates, steroids, diuretics, calcium, or vitamin D Supplement use: Women were ineligible if they were taking calcium or vitamin D Pregnant or lactating: NA  **Comparator**: Normal Protein N: 159 % Female: 100% Mean Age (SE): 59.4 (0.4) y Race/ Ethnicity: NR Menopausal status: Postmenopausal Obesity status: Obese Mean BMI (SE): 33.4 (0.4) kg/m^2^ Income level: NR Education level: NR Physical activity level: NR Health status/ Comorbidities: Subjects with parathyroid disease, a vitamin D concentration, 60 nmol/L with secondary hyperparathyroidism, or unstable metabolic, cardiac, gastrointestinal, renal, or other significant disease, including malignancies, were excluded Medication use: Women were ineligible if they were taking hormone-replacement therapy, bisphosphonates, steroids, diuretics, calcium, or vitamin D Supplement use: Women were ineligible if they were taking calcium or vitamin D Pregnant or lactating: NA | **Intervention:** High Protein  Intended Protein Amount: 32% of energy Carbohydrate: 44% of energy Fat: 24% of energy  Baseline Protein Amount  Mean (SE): 92.5 (2.2) g/d; 18.6 (0.2) % of energy Carbohydrate Mean (SE): 230 (6) g/d; 42.9 (0.5) % of energy Fat Mean (SE): 79.2 (2.7) g/d; 33.3 (0.4) % of energy  Actual Protein Amount at the end of the study Mean (SE): 91.5 (2.2) g/d; 21.9 (0.3) % of energy Carbohydrate Mean (SE): 196 (6) g/d; 43.9 (0.7) % of energy Fat Mean (SE): 55.5 (2.3) g/d; 28.2 (0.7) % of energy  Dietary Protein Intake Compliance (%): NR  Protein type/source: Mixed  Energy balance status: Hypocaloric  **Comparator:** Normal Protein   Intended Protein Amount: 22% of energy Carbohydrate: 55% of energy Fat: 23% of energy  Baseline Protein Amount Mean (SE): 91.2 (1.9) g/day; 18.4 (0.2) % of energy Carbohydrate Mean (SE): 228 (5) g/day; 42.9 (0.5) % of energy Fat Mean (SE): 77.7 (2.1) g/day; 33.4 (0.4) % of energy  Actual Protein Amount at the end of the study Mean (SE): 80.6 (2.2) g/day; 18.9 (0.3) % of energy Carbohydrate Mean (SE): 214 (5) g/day; 47.2 (0.6) % of energy Fat Mean (SE): 57.9 (2.5) g/day; 28.6 (0.7) % of energy  Protein type/source: Mixed  Dietary Protein Intake Compliance (%): NR  Energy balance status: Hypocaloric  Study duration: 24 months | **Intervention:** High Protein  **How protein was administered:** Participants received monthly group dietetic education and support for the first 6 months and then every 3 months for the next 18 months. Sample food packs of $20 vouchers were provided to participants at baseline and 12 and 26 weeks. Each diet group was allocated to a protein target that was based on key protein foods as a compliance measure.  **Protein Assessment Method:** Participants recorded dietary intakes using a protein counter and checklist. Protein compliance checklists were collected from each participant at each group session. Subjects also completed a FFQ at baseline and 1 and 2 y.   **Dietary Protein Intake Compliance:** Compliance was assessed by (1) blood urea nitrogen and 24h urine for urea nitrogen excretion (2) allocated to a protein target for each diet group and (3) protein-compliance checklists were collected from each participant at each group session.   **Comparator:** Normal Protein  **How protein was administered:** Participants received monthly group dietetic education and support for the first 6 months and then every 3 months for the next 18 months. Sample food packs of $20 vouchers were provided to participants at baseline and 12 and 26 weeks. Each diet group was allocated to a protein target that was based on key protein foods as a compliance measure. **Protein Assessment Method:** Same as above **Dietary Protein Intake Compliance:** Same as above | **BMD of the Axial Skeleton -** Bone mineral density L2-L4 (lumbar spine vertebra) Measure/Method of Assessment: DXA (Norland XR-800)  **BMD of the Appendicular Skeleton -** Bone mineral density (distal forearm, total) Measure/Method of Assessment: DXA (Norland XR-800)  **BMD of the Appendicular Skeleton -** Bone mineral density (hip, total) Measure/Method of Assessment: DXA (Norland XR-800)  **BMD of the Appendicular Skeleton -** Bone mineral density (femoral neck) Measure/Method of Assessment: DXA (Norland XR-800)  **Bone Resorption Marker -** Bone marker (C-terminal telopeptide) Measure/Method of Assessment: Blood assay  **Bone Turnover Marker (Overall Turnover) -** Osteocalcin Measure/Method of Assessment: Blood sample  **Bone Formation Marker –** Bone specific alkaline phosphatase  Measure/Method of Assessment: Blood sample |
| PMID: 25844619 **Kerstetter 2015^1,3^ (9)** Location/Country: USA HDI: Very high Setting: NR Urban/ Rural: NR Study design: RCT (parallel) Funding source: Government, academic **Risk of bias score: Low/High** | Study of: Adults Total sample N: 208  **Intervention:** High Protein N: 106 % Female: 84% Mean Age (SD): 69.9 (6.1) y Race/ Ethnicity: NR Menopausal status: NR Obesity status: NR Mean BMI (SD): 26.1 (3.4) kg/m^2^  Income level: NR Education level: NR Mean Physical activity level score (SD): 6.7 (2.1) Health status/ Comorbidities: Healthy older adults Medication use: Excluded if using long-term chemotherapeutic drugs, aromatase inhibitors or tamoxifen, methotrexate, phenytoin, phenobarbital or inhaled corticosteroids (greater than 800 ug/day), actively being treated for leukemia or multiple myeloma, a change in thyroid medications, medications known to affect calcium metabolism or use of proton pump inhibitors twice daily Supplement use: Daily multivitamin mineral supplement (contained 400 IU of vitamin D); Ca carbonate supplement (300 mg tablets) Pregnant or lactating: NR  **Comparator:** Low Protein N: 102 % Female: 87.3% Mean Age (SD): 70.5 (6.4) y Race/ Ethnicity: NR Menopausal status: NR Obesity status: NR Mean BMI (SD): 26.4 (4.0) kg/m^2^ Income level: NR Education level: NR Mean physical activity level score (SD): 6.8 (1.9) Health status/ Comorbidities: Healthy older adults Medication use: Excluded if using long-term chemotherapeutic drugs, aromatase inhibitors or tamoxifen, methotrexate, phenytoin, phenobarbital or inhaled corticosteroids (greater than 800 ug/day), actively being treated for leukemia or multiple myeloma, a change in thyroid medications, medications known to affect calcium metabolism or use of proton pump inhibitors twice daily Supplement use: Daily multivitamin mineral supplement (contained 400 IU of vitamin D); Ca carbonate supplement (300 mg tablets) Pregnant or lactating: NR | **Intervention:** High Protein Intended Protein Amount: 40 g of protein from the supplement; total daily protein goal NR Carbohydrate: Test food protein NR Fat: Test food protein NR  Baseline Protein Amount Least Square Mean (SEM): 73.8 (1.9) g Carbohydrate Least Square Mean (SEM): 214.1 (5.2) g Fat Mean (SEM): 59.4 (2.1) g  Actual Protein Amount at the end of the study Least Square Mean (SEM): 90.7 (3.3) g Carbohydrate Least Square Mean (SEM): 196.9 (6.6) g Fat Least Square Mean (SEM): 55.6 (2.0) g  Dietary Protein Intake Compliance (%): NR  Protein type/source: Animal; whey supplement   Energy balance status: Eucaloric  **Comparator:** Low Protein  Intended Protein Amount: Test food protein NR Carbohydrate: Test food protein NR Fat: Test food protein NR  Baseline Protein Amount  Least Square Mean (SEM): 72.9 (1.8) g/day; 1.06 (0.03) g/kg/day (total daily) Carbohydrate Least Square Mean (SEM): 206.2 (5.8) g/day (total daily) Fat Least Square Mean (SEM): 61.3 (2.5) g/day (total daily)  Actual Protein Amount at end of the study  Least Square Mean (SEM): 72.7 (2.4) g/day; 1.05 (0.04) g/kg/day (total daily) Carbohydrate Least Square Mean (SEM): 229.0 (9.5) g/day (total daily) Fat Least Square Mean (SEM): 58.8 (2.4) g/day (total daily)  Dietary Protein Intake Compliance (%): NR  Protein type/source: Mixed  Energy balance status: Eucaloric   Study duration: 18 months | **Intervention:** High Protein  **How protein was administered:** Participants received a dietary whey protein supplement (protein group; Provon 290; Glambia Nutritionals) that was closely matched for composition, color, kilocalories, sodium, potassium, phosphorus, fiber, and calcium. **Protein Assessment Method:** Participants completed a 3-day food record prior to baseline, 6 months, and 18 months and were analyzed using the ESHA Food Processor software program (ESHA Research; version 10.1.0).  **Dietary Protein Intake Compliance:** Urinary area was a compliance measure.   **Comparator:** Low Protein  **How protein was administered:**  Participants received a maltodextrin supplement Maltrin M100; Grain Processing Corp) that was closely matched for composition, color, kilocalories, sodium, potassium, phosphorus, fiber, and calcium.  **Protein Assessment Method:** Same as above **Dietary Protein Intake Compliance:** Same as above | **BMD of the Axial Skeleton -** Bone mineral density (lumbar spine) Measure/Method of Assessment: DXA (Hologic 4500W or Lunar Prodigy DPX-IQ)  **BMD of the Appendicular -** Bone mineral density (hip, total) Measure/Method of Assessment: DXA (Hologic 4500W or Lunar Prodigy DPX-IQ)  **BMD of the Appendicular -** Bone mineral density (femoral neck) Measure/Method of Assessment: DXA (Hologic 4500W or Lunar Prodigy DPX-IQ) |
| PMID: 21194471 **Li 2010^1^ (10)** Location/Country: United States HDI: Very high Setting: Community dwelling Urban/ Rural: NR Study Design: RCT (parallel) Funding source: Industry **Risk of bias score: High** | Study of: Adults Total sample N: 85  **Intervention:** High Protein N: 44 % Female: 81.8% Mean Age (SD): 48.9 (11.8) y Race/ Ethnicity:  Asian: 9.1% Black: 20.5% Caucasian: 59.1% Hispanic: 9.1% Other: 2.2% Menopausal status: NR Obesity status: Obese Mean BMI (SD): 34.7 (6.8) kg/m^2^ Income level: NR Education level: NR Physical activity level: NR Health status/ Comorbidities: Inclusion: good health history; participants reported to be obese; Exclusion type 2 diabetes or glucose intolerance Medication use: NR Supplement use: NR Pregnant or lactating: NR  **Comparator:** Normal Protein N: 42 % Female: 63.4% Mean Age (SD): 49.7 (9.1) y Race/ Ethnicity: Asian: 2.4% Black: 19.5% Caucasian: 68.3% Hispanic: 4.9% Other: 4.9% Menopausal status: NR Obesity status: Obese Mean BMI (SD): 34.3 (10.3) kg/m^2^  Income level: NR Education level: NR Physical activity level: NR Health status/ Comorbidities: Inclusion: good health history; participants reported to be obese; Exclusion type 2 diabetes or glucose intolerance Medication use: NR Supplement use: NR Pregnant or lactating: NR | **Intervention:** High Protein Intended Protein Amount: 2.2 g per kg of lean body mass; 30% of energy Carbohydrate: 40% of energy Fat: 30% of energy   Baseline Protein Amount Mean (SD): NR Carbohydrate Mean (SD): NR Fat Mean (SD): NR  Actual Protein Amount at the end of the study Mean (SD): NR Carbohydrate Mean (SD): NR Fat Mean (SD): NR  Dietary Protein Intake Compliance (%): NR  Protein type/source: Meal replacement protein: NR Diet: Mixed  Energy balance status: Eucaloric   **Comparator:** Normal Protein  Intended Protein Amount: 1.1 g per kg of lean body mass (15% total energy) Carbohydrate: 55% total energy Fat: 30% total energy  Baseline Protein Amount Mean (SD): NR  Carbohydrate Mean (SD): NR Fat Mean (SD): NR  Actual Protein Amount at the end of the study Mean (SD): NR Carbohydrate Mean (SD: NR Fat Mean (SD): NR  Dietary Protein Intake Compliance (%): NR  Protein type/source: Mixed  Energy balance status: Eucaloric  Study duration: 12 months | **Intervention:** High Protein **How protein was administered:** Participants received isocaloric MR (Formula 1, Herbalife Intl., Los Angeles) with a protein supplement (Performance Protein Powder, Herbalife Intl., Los Angeles)  **Protein Assessment Method:** Protein was assessed through qualitative food logs and reviewed with dietitians at follow-ups. Protein intake was measured at each follow-up visit; baseline, week 2, and months 1, 2, 3, 6, 9, 12.  **Dietary Protein Intake Compliance:** No special efforts were made to assess compliance.  **Comparator:** Normal Protein  **How protein was administered:** Participants received the isocaloric MR (Formula 1, Herbalife Intl., Los Angeles) with matched carbohydrate placebo containing maltodextrin and flavoring  **Protein Assessment Method:** Same as above **Dietary Protein Intake Compliance:** Same as above | **Total Body BMD -** Bone mineral density (total body) Measure/Method of Assessment: DXA (Lunar Prodigy DEXA) |
| PMID: 12055318 **Skov 2002 (11)** Location/Country: Denmark HDI: Very high Setting: NR Urban/ Rural: NR Study design: RCT (parallel)  Funding source: Foundation **Risk of bias score: Low** | Study of: Adults Total sample N: 65  **Intervention**: High Protein N: 25 % Female: 76% Mean Age (SD): 39.4 (2.0) y Race/ Ethnicity: 100% White Menopausal status: NR  Obesity status: Overweight or obese Mean BMI (SD): 30.8 (0.4) kg/m^2^  Income level: NR Education level: NR Physical activity level: NR Health status/Co-morbidities: Participants were overweight or obese. Exclusion was current or previous disorders, primarily concerning renal function, metabolic diseases, and cardiovascular disease. Medication use: NR Supplement use: NR Pregnant or lactating: NR  **Comparator:** Low Protein N: 25 % Female: 76% Mean Age (SD): 39.8 (1.9) y Race/ Ethnicity: 100% White Menopausal status: NR Obesity status: Overweight or obese Mean BMI (SD): 30.0 (0.4) kg/m^2^ Income level: NR Education level: NR Physical activity level: NR Health status/Co-morbidities: Participants were overweight or obese. Exclusion was current or previous disorders, primarily concerning renal function, metabolic diseases, and cardiovascular disease. Medication use: NR Supplement use: NR Pregnant or lactating: NR | **Intervention:** High Protein Intended Protein Amount: 25% of energy Carbohydrate: NR Fat: 30% of energy  Baseline Protein Amount Mean (SEM): 89.1 (3.9) g/d Carbohydrate Mean (SEM): 256.2 (13.7) g/d Fat Mean (SEM): 96.6 (5.8) g/d  Actual Protein Amount at the end of the study Mean (SEM): 102.5 (6.6) g/d Carbohydrate Mean (SEM): 316.5 (18.0) g/d Fat Mean (SEM): 76.9 (3.2) g/d  Dietary Protein Intake Compliance (%): NR  Protein type/source: Mixed  Energy balance status: Eucaloric  **Comparator:** Low Protein  Intended Protein Amount: 12% of energy Carbohydrate: NR Fat: NR  Baseline Protein Amount Mean (SEM): 87.8 (5.0) g/day Carbohydrate Mean (SEM): 256.5 (11.6) g/d Fat Mean (SEM): 107.6 (7.9) g/d  Actual Protein Amount at the end of the study Mean (SEM): 70.5 (6.7) g/d Carbohydrate Mean (SEM): 302.4 (19.7) g/d Fat Mean (SEM): 72.9 (3.5) g/d  Dietary Protein Intake Compliance (%): NR  Protein type/source: Mixed  Energy balance status: Eucaloric  Study duration: 6 months | **Intervention:** High Protein  **How protein was administered:** Participants shopped for foods from a store designed for the study. Dietitians assured macronutrient distribution of selected items; energy contents of groceries were unknown to participants.  **Protein Assessment Method:** Baseline protein assessment method was not reported. Actual Protein Amount during the intervention was derived from dieticians scanning the food participants chose from the intervention store. **Dietary Protein Intake Compliance:** Compliance to the diets was measured by 24-hour urinary nitrogen excretion.  **Comparator:** Low Protein  **How protein was administered:** Participants shopped for foods from a store designed for the study. Dietitians assured macronutrient distribution of selected items; energy contents of groceries were unknown to participants.  **Protein Assessment Method:** Same as above **Dietary Protein Intake Compliance:** Same as above | **Total Body BMC -** Bone mineral content (total body) Measure/Method of Assessment: DXA (Hologic 1000/W, software version 5.61)  **BMD of the Axial Skeleton –** Bone mineral density (regional lumbar)   Measure/Method of Assessment: DXA (Hologic 1000/W, software version 5.61)  **Whole Body BMD -** Bone mineral density (whole body)   Measure/Method of Assessment: DXA (Hologic 1000/W, software version 5.61)  **Whole Body BMC -** Bone mineral content (whole body) Measure/Method of Assessment: DXA (Hologic 1000/W, software version 5.61) |
| PMID: 34581765 **Stounbjerg 2021^2^ (12)** Location/Country: Denmark HDI: Very high Setting: Community dwelling Urban/ Rural: Urban Study design: RCT (parallel) Funding source: Public-private partnership **Risk of bias score: Low** | **Study of: Children and adolescents** Total sample N: 200  **Intervention 1:** Placebo-HP N: 50 % Female: 48% Median Age (IQR): 7.8 (7.0–8.5) y Race/ Ethnicity: 100% White Pubertal status: 4% in puberty Obesity status: 14% obese Mean BMI-for-age z-score (SD): 0.02 (1.10) Income level: NR Parental education level: ≤Vocational or short academic: 16% Bachelor’s degree: 36% ≥Master’s degree: 48% Physical activity level: NR Health status/ Comorbidities: Exclusion criteria were an allergy or intolerance to milk or milk components, chronic disease. Medication use: Exclusion criteria: use of medication that might affect study outcomes. Supplement use: Exclusion criteria: habitual use of vitamin D–containing supplements >3 days/week for the prior 2 months and at all in the month immediately preceding the start of the intervention. Pregnant or lactating: NA  **Intervention 2:** Vitamin D-HP N: 50 % Female: 44% Median Age (IQR): 7.8 (7.3–8.2) y Race/ Ethnicity: 100% White Pubertal status: 4% in puberty Obesity status: 4%) obese Mean BMI-for-age z-score (SD): − 0.15 (0.75) Income level: NR Parental education level:  ≤Vocational or short academic: 16% Bachelor’s degree: 28% ≥Master’s degree: 56% Physical activity level: NR Health status/ Comorbidities: Exclusion criteria were an allergy or intolerance to milk or milk components, chronic disease. Medication use: Exclusion criteria: use of medication that might affect study outcomes. Supplement use: Exclusion criteria: habitual use of vitamin D–containing supplements >3 days/week for the prior 2 months and at all in the month immediately preceding the start of the intervention. Pregnant or lactating: NA  **Comparator 1:** Placebo-NP N: 51 % Female: 53% Median Age (IQR): 7.6 (7.0–8.2) y Race/ Ethnicity: 100% White Pubertal status: 0% in puberty Obesity status: 16% obese Mean BMI-for-age z-score (SD): − 0.02 (1.12)  Income level: NR Parental Education level: ≤Vocational or short academic: 20% Bachelor’s degree: 22% ≥Master’s degree: 59% Physical activity level: NR Health status/ Comorbidities: Exclusion criteria were an allergy or intolerance to milk or milk components, chronic disease. Medication use: Exclusion criteria: use of medication that might affect study outcomes. Supplement use: Exclusion criteria: habitual use of vitamin D–containing supplements >3 days/week for the prior 2 months and at all in the month immediately preceding the start of the intervention. Pregnant or lactating: NA  **Comparator 2:** Vitamin D-NP N: 46 % Female: 61% Median Age (IQR): 7.6 (7.1–8.2) y Race/ Ethnicity: 100% White Pubertal status: 8% in puberty Obesity status: 16% obese Mean BMI-for-age z-score (SD): 0.34 (1.04)  Income level: NR Parental education level:  ≤Vocational or short academic: 10% Bachelor’s degree: 24% ≥Master’s degree: 65% Physical activity level: NR Health status/ Comorbidities: Exclusion criteria were an allergy or intolerance to milk or milk components, chronic disease. Medication use: Exclusion criteria: use of medication that might affect study outcomes. Supplement use: Exclusion criteria: habitual use of vitamin D–containing supplements >3 days/week for the prior 2 months and at all in the month immediately preceding the start of the intervention. Pregnant or lactating: NA | **Intervention 1:** Placebo-HP Intended Protein Amount: 9.6 g/100g Carbohydrate: 5 g/100 g Fat: 0.2 g/100 g  Baseline Protein Amount Mean (SD): 15.4 (2.4) % of energy Carbohydrate Mean (SD): 52.5 (4.7) % of energy Fat Mean (SD): 32.1 (4.7) % of energy  Actual Protein Amount at the end of the study Mean (SD): 17.7 (3.3) % of energy Carbohydrate Mean (SD): 53.5 (5.6) % of energy Fat Mean (SD): 28.8 (4.9) % of energy  Dietary Protein Intake Compliance (%): NR  Protein type/source: Animal; low-fat yogurt  Energy balance status: Eucaloric  **Intervention 2:** Vitamin D-HP Intended Protein Amount: 9.6 g/100g Carbohydrate: 5 g/100 g Fat: 0.2 g/100 g  Baseline Protein Amount Mean (SD): 15.7 (2.3) % of energy Carbohydrate Mean (SD): 52.0 (4.6) % of energy Fat Mean (SD): 32.4 (4.5) % of energy  Actual Protein Amount at the end of the study Mean (SD): 19.0 (3.4) % of energy Carbohydrate Mean (SD): 49.6 (5.1) % of energy Fat Mean (SD): 31.4 (4.6) % of energy  Dietary Protein Intake Compliance (%): NR  Protein type/source: Animal; low-fat yogurt  Energy balance status: Eucaloric  **Comparator 1:** Placebo-NP Intended Protein Amount: 3.6 g/100g Carbohydrate: 8.6 g/100 g Fat: 2.3 g/100 g  Baseline Protein Amount Mean (SD): 15.0 (2.2) % of energy Carbohydrate Mean (SD): 54.6 (4.8) % of energy Fat Mean (SD): 30.3 (4.5) % of energy  Actual Protein Amount at the end of the study Mean (SD): 15.8 (2.7) % of energy  Carbohydrate Mean (SD): 52.9 (4.9) % of energy Fat Mean (SD): 31.3 (4.3) % of energy  Protein type/source: Animal; yogurt  Energy balance status: Eucaloric  **Comparator 2:** Vitamin D-NP Intended Protein Amount: 3.6 g/100g Carbohydrate: 8.6 g/100 g Fat: 2.3 g/100 g  Baseline Protein Amount Mean (SD): 15.7 (2.6) % of energy Carbohydrate Mean (SD): 54.5 (4.2) % of energy Fat Mean (SD): 29.7 (4.3) % of energy  Actual Protein Amount at the end of the study Mean (SD): 16.0 (2.2) % of energy Carbohydrate Mean (SD): 51.7 (5.0) % of energy Fat Mean (SD): 32.3 (4.8) % of energy  Protein type/source: Animal; yogurt  Energy balance status: Eucaloric  Study duration: 24 weeks | **Intervention 1:** Placebo-HP **How protein was administered:** Participants took 300g/d for 6 days/week of a drained low-fat yogurt (“skyr”) with a high protein content of 9-11 g protein/100 g plus a chewable placebo (from Oy Verman Ab) of identical appearance and taste were provided in identical, white tablet bottles containing 200 tablets. **Protein Assessment Method:** Protein amounts were derived from a dietary recording with a minimum of 3 recording days (4-day dietary record coving 3 consecutive weekdays and 1 weekend day) where parents weighed and recorded everything the child ate and drank (except water) in the web-based software Madlog (if weighing not possible, household measures were used). Protein intake was measured prior to baseline and at endpoint visits.   **Dietary Protein Intake Compliance:** Parents recorded the child’s daily intake of the specific yogurts during the intervention in recording sheets.   **Intervention 2:** Vitamin D-HP  **How protein was administered:** Participants took 300g/d for 6 days/week of a drained low-fat yogurt (“skyr”) with a high protein content of 9-11 g protein/100 g plus a chewable 20 μg of vitamin D3 (Minisun; from Oy Verman Ab) of identical appearance and taste were provided in identical, white tablet bottles containing 200 tablets.  **Protein Assessment Method:** Same as above **Dietary Protein Intake Compliance:** Same as above  **Comparator 1:** Placebo-NP  **How protein was administered:** Participants took 300g/d for 6 days/week of a regular yogurt with protein content of 3.0 – 3.9 g protein/100 g plus a chewable placebo (from Oy Verman Ab) of identical appearance and taste were provided in identical, white tablet bottles containing 200 tablets.  **Protein Assessment Method:** Same as above **Dietary Protein Intake Compliance:** Same as above  **Comparator 2:** Vitamin D-NP  **How protein was administered:** Participants took 300g/d for 6 days/week of a regular yogurt with protein content of 3.0 – 3.9 g protein/100 g plus a chewable placebo (from Oy Verman Ab) of identical appearance and taste were provided in identical, white tablet bottles containing 200 tablets.  **Protein Assessment Method:** Same as above **Dietary Protein Intake Compliance:** Same as above | **Bone Turnover Marker (Overall Turnover) -** Osteocalcin Measure/Method of Assessment: Venous blood samples  **BMD of the Axial Skeleton -** Bone mineral density (L1–L4 (lumbar spine vertebrae))  Measure/Method of Assessment: DXA (GE Lunar Prodigy scanner)   **BMC Axial Skeleton -** Bone mineral content (L1-L4 (lumbar spine vertebrae))   Measure/Method of Assessment: DXA (GE Lunah Prodigy scanner)   **Bone Geometry and Strength Indices -** Bone area (L1–L4 (lumbar spine vertebrae)   Measure/Method of Assessment: DXA (GE Lunah Prodigy scanner)   **BMD of the Axial Skeleton -** Bone mineral density z-score **(**L1–L4 (lumbar spine vertebrae)   Measure/Method of Assessment: DXA (GE Lunah Prodigy scanner) software computed zscores |
| PMID: 21590739 **Zhu 2011 (13)** Location/Country: Australia HDI: Very high Setting: Community dwelling Urban/ Rural: Metropolitan  Study design: RCT (parallel) Funding source: Government, academic **Risk of bias score: High** | Study of: Adults Total sample N: 192  **Intervention:** High Protein N: 101  % Female: 100% Mean Age (SD): 74.2 (2.8) y Race/ Ethnicity: NR Menopausal status: Postmenopausal Obesity status: NR Mean BMI (SD): 26.1 (3.8) kg/m^2^ Income level: NR Education level: NR Mean physical activity level (SD): 449 (391) MET-min/wk Health status/ Comorbidities: No previous osteoporotic fracture, currently or within last year taking medication for osteoporosis apart from calcium or vitamin D, or have taken more than 7 g in total in lifetime, metabolic bone disease apart from osteoporosis, total-hip bone density more than 2 SD below the mean for age, malabsorption disorders, celiac disease, clinical hepatic or renal insufficiency, clinical diagnosis of diabetes Medication use: Exclusion criteria: taking medication for osteoporosis apart from calcium or vitamin D, taking steroid tablets in the past 3 months or have taken more than 7 g in total in lifetime Supplement use: NR Pregnant or lactating: NA  **Comparator:** Normal Protein N: 91 % Female: 100% Mean Age (SD): 74.3 (2.6) y Race/ Ethnicity: NR Menopausal status: Postmenopausal Obesity status: NR Mean BMI (SD): 27.2 (4.0) kg/m^2^ Income level: NR Education level: NR Mean physical activity level (SD): 398 (376) MET-min/wk Health status/ Comorbidities: No previous osteoporotic fracture, currently or within last year taking medication for osteoporosis apart from calcium or vitamin D, or have taken more than 7 g in total in lifetime, metabolic bone disease apart from osteoporosis, total-hip bone density more than 2 SD below the mean for age, malabsorption disorders, celiac disease, clinical hepatic or renal insufficiency, clinical diagnosis of diabetes Medication use: Exclusion criteria: taking medication for osteoporosis apart from calcium or vitamin D, taking steroid tablets in the past 3 months or have taken more than 7 g in total in lifetime Supplement use: NR Pregnant or lactating: NA | **Intervention:** High Protein Intended Protein Amount: 30.1 g Carbohydrate: 13.2 g Fat: 2.3  Baseline Protein Amount Mean (SD): 76 (18) g/d Carbohydrate Mean (SD): 185 (45) g/d Fat Mean (SD): 63 (18) g/d  Actual Protein Amount at the end of the study Mean (SD): 95 (20) g/d Carbohydrate Mean (SD): 183 (52) g/d Fat Mean (SD): 62 (21) g/d  Dietary Protein Intake Compliance (%): 81.1%  Protein type/source: Animal; skim milk plus whey protein isolate  Energy balance status: Eucaloric  **Comparator:** Normal Protein  Intended Protein Amount: 2.1 g Carbohydrate: 42.3 Fat: 2.0 g  Baseline Protein Amount Mean (SD): 76 (16) g/d Carbohydrate Mean (SD): 190 (42) g/d Fat Mean (SD): 63 (20) g/d  Actual Protein Amount at the end of the study Mean (SD): 73 (17) g/d Carbohydrate Mean (SD): 204 (47) g/d Fat Mean (SD): 60 (17) g/d  Dietary Protein Intake Compliance (%): 80.8%  Protein type/source: Animal; skim milk plus whey protein isolate  Energy balance status: Eucaloric  Study duration: 2 y | **Intervention:** High Protein **How protein was administered:** 250-mL skim milk–based high-protein supplement drink reconstituted with cold water from a powder that provided 30 g of protein (skim milk plus whey protein isolate; Alacen 894, Fonterra Brands, Ltd., Palmerston North, New Zealand), 600mg of calcium, and 3.2 kJ/mL of energy. **Protein Assessment Method:** Protein was reported at baseline, 1 year, and 2 years; assessed through 3-day weighed food records (2 weekdays, 1 weekend day)  **Dietary Protein Intake Compliance:** Compliance was determined from empty test drink containers.  **Comparator:** Normal Protein  **How protein was administered:** 250-mL skim milk–based high-protein supplement drink reconstituted with cold water from a powder that provided 2.1 g of protein (skim milk plus whey protein isolate; Alacen 894, Fonterra Brands, Ltd., Palmerston North, New Zealand), 600mg of calcium, and 3.2 kJ/mL of energy **Protein Assessment Method:** Same as above **Dietary Protein Intake Compliance:** Same as above | **aBMD of the Appendicular Skeleton -** Areal bone mineral density (hip, total)   Measure/Method of Assessment: DXA (Hologic Discovery A fan-beam densitometer])  **aBMD of the Appendicular Skeleton -** Areal bone mineral density (femoral neck)  Measure/Method of Assessment: DXA (Hologic Discovery A fan-beam densitometer)  **Total body vBMD -** Volumetric bone mineral density (total body)  Measure/Method of Assessment: QCT scans (Hologic Discovery A fan-beam densitometer)  **vBMD of the Appendicular Skeleton -** Volumetric bone mineral density (femoral neck)   Measure/Method of Assessment: QCT scans (Hologic Discovery A fan-beam densitometer)  **Bone Geometry and Strength Indices -** Femoral neck cross-sectional area Measure/Method of Assessment: QCT scans (Hologic Discovery A fan-beam densitometer)  **Bone Geometry and Strength Indices -** Femoral neck buckling ratio Measure/Method of Assessment: QCT scans (Hologic Discovery A fan-beam densitometer)  **Bone Geometry and Strength Indices -** Femoral neck polar CSMI (cross-sectional moment of inertia) Measure/Method of Assessment: QCT scans (Hologic Discovery A fan-beam densitometer) |

**Abbreviations:** μg/L = micrograms per liter; BAP = Bone alkaline phosphatase; BMI = body mass index; BMC = bone mineral content; BMD = bone mineral density; CTX = carboxy terminal crosslinked telopeptide of type I collagen; DXA = Dual-energy x-ray absorptiometry; e.g. = exempli gratia; FFQ = food frequency questionnaire; g = grams; g/100g = grams per 100 grams; g/cm2 = grams per centimeter squared; g/d = grams per day; HDI = human development index; HP = high protein; IQR = interquartile range; IU= international units; kg/m2 = kilograms per meter squared; min/wk = minutes per week; mg = milligrams; mL = milliliter; MPB = milk based protein; MR= meal replacement; NA = not applicable; NP = normal protein; NR = not reported; P1NP = Procollagen type 1 N-terminal propeptide; PMID = PubMed Identification Number; RCT = randomized controlled trail; RoB = Risk of Bias; SD = standard deviation; SE = standard error; SEM = standard error of the mean; TRAP = (5b, tartrate resistant acid phosphatase, isoform 5); USA = United States of America; wk = week; y = year
^1^Studies overlap KQs; ^2^Child and Adolescent study; ^3^Kerstetter, 2015 reported on KQ1, KQ2, and KQ3 outcomes: KQ1 outcomes were assessed as both low (including BMD lumbar, hip and femoral outcomes) and high risk of bias (including all other reported outcomes)

Supplementary Table 3. Evidence table for Bone Disease Non-Randomized Controlled Trials (Adults)

| Study | Participants | Intervention(s) (Content) | Intervention(s) (Methods of assessment | Outcome (Measures and methods assessment) |
| --- | --- | --- | --- | --- |
| PMID: 20219968 **Beasley 2010 (14)** Location/Country: USA HDI: Very high  Setting: NR Urban/Rural: NR Study design: Prospective cohort study Funding source: Government **Risk of bias score: High** | Study of: Adults  Total sample N: 560  **Tertile 1:** Protein intake 5.7-14.3% of energy (low)  N: 186 % Female: 100% Mean Age (SD): 24.2 (6.6) y Race/ Ethnicity:  White: 77% Black: 11% Other: 12% Menopausal status: Premenopausal Obesity status: NR Mean BMI (SD): 24.9 (5.2) kg/m^2^  Income level: NR Education level: NR Mean physical activity level: 78.2 physical activity score Health status/ Comorbidities: Women with conditions known to affect bone mass were excluded.  Medication use: Women taking medications known to affect bone mass were excluded. Supplement use: NR Pregnant or lactating: NR  **Tertile 2:** Protein intake 14.4-17.1% of energy (medium) N: 187 % Female: 100% Mean Age (SD): 24.3 (6.9) y Race/ Ethnicity:  White: 82% Black: 10% Other 8% Menopausal status: Premenopausal Obesity status: NR Mean BMI (SD): 24.5 (5.9) kg/m^2^ Income level: NR Education level: NR Mean physical activity level: 74.5 physical activity score Health status/ Comorbidities: Women with conditions known to affect bone mass were excluded.  Medication use: Women taking medications known to affect bone mass were excluded. Supplement use: NR Pregnant or lactating: NR  **Tertile 3:** Protein intake 17.2-27.6% of energy (high) N: 187 % Female: 100% Mean Age (SD): 25.4 (7.4) y Race/ Ethnicity:  White: 76%  Black: 15% Other 9% Menopausal status: Premenopausal Obesity status: NR Mean BMI (SD): 25.9 (5.3) kg/m^2^ Income level: NR Education level: NR Mean physical activity level: 81.6 physical activity score Health status/ Comorbidities: Women with conditions known to affect bone mass were excluded.  Medication use: Women taking medications known to affect bone mass were excluded. Supplement use: NR Pregnant or lactating: NR | **Tertile 1:** Protein intake 5.7-14.3% of energy (low)  Baseline Protein Amount  Mean (SD):  Animal protein: 33.2 (18.1) g Vegetable protein: 18.6 (10.2) g Carbohydrate Mean (SD): 55% of energy Fat Mean (SD): 33% of energy  Protein Amount at the end of the study Mean (SD): NR Carbohydrate Mean (SD): NR Fat Mean (SD): NR  **Tertile 2:** Protein intake 14.4-17.1% of energy (medium)  Intended protein amount: NR Carbohydrate: NR Fat: NR  Baseline Protein Amount Mean (SD):  Animal protein: 43.3 (18.8) g Vegetable protein: 19.9 (9.2) g Carbohydrate Mean (SD): 52% of energy Fat Mean (SD): 33% of energy  Protein Amount at the end of the study Mean (SD): NR Carbohydrate Mean (SD): NR Fat Mean (SD): NR   **Tertile 3:** Protein intake 17.2-27.6% of energy (high)  Intended Protein Amount: NR Carbohydrate: NR Fat: NR  Baseline Protein Amount Mean (SD): Animal protein: 58.7 (25.8) g Vegetable protein: 18.6 (8.2) g Carbohydrate Mean (SD): 49% of energy Fat Mean (SD): 32% of energy  Protein Amount at the end of the study Mean (SD): NR Carbohydrate Mean (SD): NR Fat Mean (SD): NR  Protein type/source: Mixed  Energy balance status: Eucaloric   Study duration: Up to 3 y | **Protein Assessment Method:** Protein intake was measured using an FFQ developed and evaluated in the Women’s Health Initiative at the Fred Hutchinson Cancer Research Center. Consistent with current dietary guidelines, protein was evaluated as a percentage of total energy. Protein intake was assessed at baseline and at annual follow-up visits. | **BMD of the Axial Skeleton -** Bone mineral density (lumbar spine)  Measure/Method of Assessment: DXA (Hologic 2000 and Hologic 4500)  **BMD of the Appendicular Skeleton -** Bone mineral density (hip, total)   Measure/Method of Assessment: DXA (Hologic 2000 and Hologic 4500)  **Total Body BMD -** Bone mineral density (total body)  Measure/Method of Assessment: DXA (Hologic 2000 and Hologic 4500) |
| PMID: 24552750 **Beasley** **2014 (15)** Location/Country: USA HDI: Very high  Setting: NR Urban/ Rural: NR Study design: Prospective cohort study Funding source: Government **Risk of bias score: High** | Study of: Adults Total sample N: 144,580  **Tertile 1:** Protein intake <13.3% of energy N: NR % Female: 100% Mean Age (SD): 66 (7.2) y Race/ Ethnicity:  White: 77.2% Black: 14.4% Hispanic: 3.9% American Indian: 0.5% Asian/Pacific Islander: 2.3% Unknown: 1.6%  Menopausal status: Postmenopausal Obesity status: 37.5%  Mean BMI (SD): NR Income level: NR Education level: NR Mean physical activity level: 9.9 METs/wk Health status/ Comorbidities:  Fair/poor: 12.5% Good: 37.8%  Excellent/very good: 49.7% Medication use: NR Supplement use: All participants supplemented with calcium and vitamin D  Pregnant or lactating: NA  **Tertile 2:** Protein intake 14.2-14.8% of energy N: NR % Female: 100% Mean Age (SD): 63.7 (6.9) y Race/ Ethnicity:  White: 84.7%  Black: 7.4%  Hispanic: 3.4% American Indian: 0.3%  Asian/ Pacific Islander: 2.7% Unknown: 1.4% Menopausal status: Postmenopausal Obesity status: 30.6%  Mean BMI (SD): NR Income level: NR Education level: NR Mean physical activity level: 12.6 METs/w Health status/ Comorbidities:  Fair/poor: 8.1%  Good: 33.0%  Excellent/very good: 58.9% Medication use: NR Supplement use: All participants supplemented with calcium and vitamin D Pregnant or lactating: NA  **Tertile 3:** Protein intake ≥15.6% of energy N: NR % Female: 100% Mean Age (SD): 59.6 (6.4) y Race/ Ethnicity:  White: 85.1%  Black: 6.0%  Hispanic: 4.1%  American Indian: 0.4% Asian/ Pacific Islander: 3.1%  Unknown: 1.3%  Menopausal status: Postmenopausal Obesity status: 21.1% Mean BMI (SD): NR Income level: NR Education level: NR Mean physical activity level: 15.0 METs/wk Health status/ Comorbidities:  Fair/poor: 6.5%  Good: 27.3% Excellent/very good: 66.2% Medication use: NR Supplement use: All participants supplemented with calcium and vitamin D Pregnant or lactating: NA | **Tertile 1:** Protein intake <13.3% of energy  Baseline Protein Amount: <13.3% of energy Carbohydrate: NR Fat: NR  Actual Protein Amount at the end of the study: NR Carbohydrate: NR Fat: NR  **Tertile 2:** Protein intake 14.2-14.8% of energy  Baseline Protein Amount: 14.2-14.8% of energy Carbohydrate: NR Fat: NR  Protein Amount at the end of the study: NR Carbohydrate: NR Fat: NR  **Tertile 3:** Protein intake ≥15.6% of energy  Baseline Protein Amount: ≥15.6% of energy Carbohydrate: NR Fat: NR  Protein Amount at the end of the study: NR Carbohydrate: NR Fat: NR  Protein type/source: Mixed  Energy balance status: Eucaloric  Study duration: 6 y | **Protein Assessment Method:** Self-administered FFQ that included 122 items for individual foods and food groups, 19 adjustment items, and summary questions. Protein intake was assessed at baseline. | **Total Body BMD -** Bone mineral density (total body)   Measure/Method of Assessment: DXA (Hologic QDR densitometer)  **BMD of the Appendicular Skeleton -** Bone mineral density (hip, total)   Measure/Method of Assessment: DXA (Hologic QDR densitometer)  **BMD of the Axial Skeleton -** Bone mineral density (spine)  Measure/Method of Assessment: DXA (Hologic QDR densitometer)  **Osteoporotic Fractures and Fracture Risk -**  Fragility fracture (osteoporotic and low-trauma fracture)  Hip fracture Spine fracture Forearm fracture  Measure/Method of Assessment: Self-report (CTs when available) |
| PMID: 26988112 **Cauley** **2016 (16)** Location/Country: USA  HDI: Very high Setting: Community dwelling Urban/ Rural: Urban Study design: Prospective cohort study Funding source: Government **Risk of bias score: High** | Study of: Adults Total sample N: 5,876  **Arm 1:** No hip fracture N: 5,698 % Female: 0% Mean Age (SD): 73.48 (5.81) y Race/ Ethnicity: NR Menopausal status: NA Obesity status: NR Mean BMI (SD): NR Income level: NR Education level: 76.11% ≥High school Mean physical activity level: 147.58 PASE score Health status/ Comorbidities: Participants who could not walk without the assistance of another or had bilateral hip replacements excluded. Medication use: Participants taking osteoporosis medications excluded.  Supplement use: NR Pregnant or lactating: NA  **Arm 2:** Hip fracture N: 178 % Female: 0% Mean Age (SD): 77.81 (6.08) y Race/ Ethnicity: NR Menopausal status: NA Obesity status: NR Mean BMI (SD): NR Income level: NR Education level: 72.47% ≥High school Mean physical activity level: 131.89 PASE score Health status/ Comorbidities: Participants who could not walk without the assistance of another or had bilateral hip replacements excluded.  Medication use: Participants taking osteoporosis medications excluded. Supplement use: NR Pregnant or lactating: NA | **Arm 1:** No hip fracture  Baseline Protein Amount  Mean (SD): 16.13 (2.91) % of energy Carbohydrate Mean (SD): NR Fat Mean (SD): NR  Protein Amount at the end of the study Mean (SD): NR Carbohydrate Mean (SD): NR Fat Mean (SD): NR  **Arm 2:** Hip fracture  Baseline Protein Amount Mean (SD): 15.3 (2.55) % of energy Carbohydrate Mean (SD): NR Fat Mean (SD): NR  Protein Amount at the end of the study Mean (SD): NR Carbohydrate Mean (SD): NR Fat Mean (SD): NR  Protein type/source: Mixed  Energy balance status: Eucaloric  Study duration: 8.6 y | **Protein Assessment Method:** Block 98 semiquantitative FFQ was administered. Diet quality was calculated using the validated Quality Index Revised. Protein intake was assessed at baseline. | **Osteoporotic Fractures and Fracture Risk -** Hip fracture   Measure/Method of Assessment: Self-report & physician adjudicated of medical records |
| PMID: 21437561 **Chan** **2011 (17)** Location/Country: China HDI: Very high  Setting: Community dwelling Urban/ Rural: Urban Study design: Prospective cohort study Funding source: Government **Risk of bias score: High** | Study of: Adults Total sample N: 2,217  **Arm 1:** Men N: 1,225 % Female: 0% Mean Age (SD): 71.6 (4.6) y Race/ Ethnicity: NR Menopausal status: NA Obesity status: NR Mean BMI (SD): 23.5 (3.1) kg/m^2^ Income level: NR Education level: 41.1% secondary school or above Mean physical activity level: 101.7 PASE score Health status/ Comorbidities: Participants who had any detectable disease or medication known to affect bone mass were excluded.  Medication use: Participants who were taking medication known to affect bone mass were excluded.  Supplement use: 9.6% used calcium supplements Pregnant or lactating: NA  **Arm 2:** Women  N: 992 % Female: 100% Mean Age (SD): 72.0 (5.1) y Race/ Ethnicity: NR Menopausal status: NR Obesity status: NR Mean BMI (SD): 24.0 (3.5) kg/m^2^ Income level: NR Education level: 17.4% secondary school or above Mean physical activity level: 87.6 PASE score Health status/ Comorbidities: Participants who had any detectable disease or medication known to affect bone mass were excluded. Medication use: Participants who were taking medication known to affect bone mass were excluded. Supplement use: 15.0% used calcium supplements Pregnant or lactating: NR | **Arm 1:** Men  Baseline Protein Amount  Mean (SD): 88.8 (35.3) g/d Carbohydrate Mean (SD): NR Fat Mean (Range): 23.4 (16.4-28.1) g/d  Protein Amount at the end of the study Mean (SD): NR Carbohydrate Mean (SD): NR Fat Mean (Range): NR  **Arm 2**: Women  Baseline Protein Amount Mean (SD): 65.7 (27.5) g/d Carbohydrate Mean (SD): NR  Fat Mean (Range): 16.4 (9.4-21.6) g/d  Protein Amount at the end of the study Mean (SD): NR Carbohydrate Mean (SD): NR Fat Mean (Range): NR  Protein type/source: Mixed  Energy balance status: Eucaloric  Study duration: 4 y | **Protein Assessment Method:** Dietary intake was assessed at baseline using an FFQ. Mean nutrient quantification per day was calculated using food tables derive from McCance and Widdowson and the Chinese Medical Sciences Institute. | **BMD of the Appendicular Skeleton -** Bone mineral density (hip, total)  Measure/Method of Assessment: DXA (Hologic QDR-4500 W densitometers)  **BMD of the Appendicular Skeleton -** Bone mineral density (femoral neck)   Measure/Method of Assessment: DXA (Hologic QDR-4500 W densitometers) |
| PMID: 18665794 **Dargent-Molina** **2008 (18)** Location/Country: France HDI: Very high Setting: NR Urban/ Rural: NR Study design: Prospective cohort study Funding source: Government **Risk of bias score: High** | Study of: Adults Total sample N: 36,217  **Arm 1:** No fractures N: 33,809 % Female: 100% Mean Age (SD): 56.1 (5.5) y Race/ Ethnicity: NR Menopausal status: Postmenopausal Obesity status: NR Mean BMI (SD): 23.2 (3.3) kg/m^2^  Income level: NR Education level: NR Mean physical activity level: 52.7 METS/d Health status/ Comorbidities: NR Medication use: NR Supplement use: 20.8% used calcium supplements Pregnant or lactating: NA  **Arm 2:** Fractures N: 2,408 % Female: 100% Mean Age (SD): 57.1 (5.6) y Race/ Ethnicity: NR Menopausal status: Postmenopausal Obesity status: NR Mean BMI (SD): 23.3 (3.4) kg/m^2^ Income level: NR Education level: NR Mean physical activity level: 53.7 METS/d Health status/ Comorbidities: NR Medication use: NR Supplement use: 12.2% used calcium supplements Pregnant or lactating: NA | **Arm 1:** No fractures  Baseline Protein Amount Mean (SD): 45.7 (7.3) g/1000 kcal/d Carbohydrate Mean (SD): NR Fat Mean (SD): NR  Protein Amount at the end of the study Mean (SD): NR Carbohydrate Mean (SD): NR Fat Mean (SD): NR  **Arm 2:** Fractures  Baseline Protein Amount  Mean (SD): 46.0 (7.6) g/1000 kcal/d Carbohydrate Mean (SD): NR Fat Mean (SD): NR  Protein Amount at the end of the study Mean (SD): NR Carbohydrate Mean (SD): NR Fat Mean (SD): NR  Protein type/source: Mixed  Energy balance status: Eucaloric  Study duration: 15 y | **Protein Assessment Method:** The dietary questionnaire was composed of two parts, the first including questions on the consumption (quantity and frequency) of food groups and the second qualitative questions allowing detailing the food groups into food items. The questionnaire assessed dietary consumption of 208 items. It was sent with a booklet of photos to facilitate the estimation of portion sizes. Protein intake was assessed at baseline. | **Osteoporotic Fractures and Fracture Risk -** Fragility fracture (osteoporotic and low-trauma fracture) Measure/method of assessment: Self-report |
| PMID: 15941897 **Devine**  **2005 (19)** Location/Country: Australia HDI: Very high Setting: NR Urban/ Rural: NR Study design: Prospective cohort study Funding source: Nonprofit, government **Risk of bias score: High** | Study of: Adults Total sample N: 1,077  **Arm 1:** Whole cohort N: 1,077 % Female: 100% Mean Age (SD): 75 (3) y Race/ Ethnicity: NR  Menopausal status: Postmenopausal  Obesity status: NR Mean BMI (SD): 27.1 (4.5) kg/m^2^ Income level: NR Education level: NR Physical activity level: NR Health status/ Comorbidities: Participants excluded if they had significant current illness. Medication use: Participants excluded if receiving pharmaceutical agents that act on bone, including calcium supplements.  Supplement use: Participants excluded if receiving pharmaceutical agents that act on bone, including calcium supplements. Pregnant or lactating: NA | **Arm 1:** Whole cohort  Baseline Protein Amount Mean (SD): 80.5 (27.8) g Carbohydrate Mean (SD): 192 (59) g Fat Mean (SD): 64.5 (24.5) g  Protein Amount at the end of the study Mean (SD): NR Carbohydrate Mean (SD): NR Fat Mean (SD): NR   Protein type/source: Mixed   Energy balance status: Eucaloric   Study duration: 1 y | **Protein Assessment Method:** Each subject completed a self-administered, semiquantitative FFQ developed by the Anti-Cancer Council of Victoria (ACCV) from which information on the daily dietary intakes of energy, carbohydrate, protein, fat, and calcium was derived. Protein intake was assessed at baseline. | **BMD of the Appendicular Skeleton -** Bone mineral density (hip, total)   Measure/Method of Assessment: DXA (Acclaim QDR 4500A fan-beam densitometer)  **BMD of the Appendicular Skeleton -** Bone mineral density (trochanter)   Measure/Method of Assessment: DXA (Acclaim QDR 4500A fan-beam densitometer)  **BMD of the Appendicular Skeleton -** Bone mineral density (intertrochanter)   Measure/Method of Assessment: DXA (Acclaim QDR 4500A fan-beam densitometer)  **BMD of the Appendicular Skeleton -** Bone mineral density (femoral neck)  Measure/Method of Assessment: DXA (Acclaim QDR 4500A fan-beam densitometer) |
| PMID: 11127216 **Hannan** **2000 (20)** Location/Country: USA HDI: Very high  Setting: NR Urban/ Rural: NR Study design: Prospective cohort study Funding source: Government **Risk of bias score: High** | Study of: Adults Total sample N: 855  **Arm 1:** Attended both exams N: 615 % Female: 64% Mean Age (SD): 74.5 (4.4) y Race/ Ethnicity: NR Menopausal status: NR Obesity status: NR Mean BMI (SD): NR Income level: NR Education level: NR Physical activity level: NR Health status/ Comorbidities: NR Medication use: 7% estrogen use Supplement use: NR Pregnant or lactating: NR  **Arm 2:** Attended only baseline exam N: 240 % Female: 55% Mean Age (SD): 77.2 (5.3) y Race/ Ethnicity: NR Menopausal status: NR Obesity status: NR Mean BMI (SD): NR Income level: NR Education level: NR Physical activity level: NR Health status/ Comorbidities: NR Medication use: 3% estrogen use Supplement use: NR Pregnant or lactating: NR | **Arm 1:** Attended both exams  Baseline Protein Amount Mean (SD): 68.5 (23.6) g/d Carbohydrate Mean (SD): NR  Fat Mean (SD): NR  Protein Amount at the end of the study Mean (SD): NR Carbohydrate Mean (SD): NR Fat Mean (SD): NR   **Arm 2:** Attended only baseline exam   Baseline Protein Amount Mean (SD): 66.8 (24.4) g/d Carbohydrate Mean (SD): NR  Fat Mean (SD): NR  Protein Amount at the end of the study Mean (SD): NR Carbohydrate Mean (SD): NR Fat Mean (SD): NR   Protein type/source: Mixed   Energy balance status: Eucaloric   Study duration: 4 y | **Protein Assessment Method:** Dietary intake was assessed using the 126-item Willett FFQ. Data were converted to food and nutrient intake data. Protein intake was assessed at baseline and two years later. | **BMD of the Axial Skeleton -** Bone mineral density, mean percent bone loss (spine)  Measure/Method of Assessment: DXA (DPX-L densitometer)  **BMD of the Appendicular Skeleton -** Bone mineral density, mean percent bone loss (hip)  Measure/Method of Assessment: DXA (DPX-L densitometer)  **BMD of the Appendicular Skeleton -** Bone mineral density, mean percent bone loss (radius)  Measure/Method of Assessment: DXA (DPX-L densitometer) |
| PMID: 25192416 **Hu** **2014 (21)** Location/Country: USA HDI: Very high  Setting: Community dwelling Urban/ Rural: NR Study design: Prospective cohort study Funding source: Nonprofit, government, academic  **Risk of bias score: High** | Study of: Adults Total sample N: 1,658  **Quartile 1:** Protein intake 6.1–13.6% of energy N: 414 % Female: 45.8% Mean Age (SD): 62.2 (9.9) y Race/ Ethnicity:  White: 46.1% Chinese: 6.1%  Black: 21.7% Hispanic: 26.1% Menopausal status: NR Obesity status: NR Mean BMI (SD): 28.2 (5.2) kg/m^2^ Income level: NR Education level:  ≥College-level education: 64.4% Mean physical activity level: 5,249 MET-min/week Health status/ Comorbidities: Participants free of CVD included. Medication use: NR Supplement use: 63.4% take multivitamin  Pregnant or lactating: NR  **Quartile 2:** Protein intake 13.7–15.7% of energy N: 415 % Female: 44.5% Mean Age (SD): 63.4 (10) y Race/ Ethnicity: White: 45.2% Chinese: 8.6% Black: 21.3%  Hispanic: 24.9%  Menopausal status: NR Obesity status: NR Mean BMI (SD): 28.2 (5.0) kg/m^2^ Income level: NR Education level: ≥College-level education: 65.3% Mean physical activity level: 5,399 MET-min/week Health status/ Comorbidities: Participants free of CVD included. Medication use: NR Supplement use: 61.4% take multivitamin  Pregnant or lactating: NR  **Quartile 3:** Protein intake 15.8–17.9% of energy N: 413 % Female: 49.6% Mean Age (SD): 61.5 (10) y Race/ Ethnicity: White: 39.2% Chinese: 15.3% Black: 17.3% Hispanic: 28.2%  Menopausal status: NR Obesity status: NR Mean BMI (SD): 27.9 (5.0) kg/m^2^ Income level: NR  Education level: ≥College-level education: 62.8% Mean physical activity level: 5,101 MET-min/week Health status/ Comorbidities: Participants free of CVD included. Medication use: NR Supplement use: 59.9% take multivitamin  Pregnant or lactating: NR  **Quartile 4:** Protein intake 18.0–33.5% of energy N: 416 % Female: 53.4% Mean Age (SD): 62.1 (9.3) y Race/ Ethnicity:  White: 36.5% Chinese: 26.0%  Black: 12.5% Hispanic: 25.0% Menopausal status: NR Obesity status: NR Mean BMI (SD): 27.8 (5.2) kg/m^2^ Income level: NR  Education level: ≥College-level education: 66.6% Mean physical activity level: 4,989 MET-min/week Health status/ Comorbidities: Participants free of CVD included. Medication use: NR Supplement use: 65.9% take multivitamin  Pregnant or lactating: NR | **Quartile 1:** Protein intake 6.1–13.6% of energy  Baseline Protein Amount  Mean (SD): 49.6 (24.5) g Carbohydrate Mean (SD): 55.8 (10.4) % of energy Fat Mean (SD): 30.8 (7.9) % of energy  Protein Amount at the end of the study Mean (SD): NR Carbohydrate Mean (SD): NR Fat Mean (SD): NR   **Quartile 2:** Protein intake 13.7–15.7% of energy  Baseline Protein Amount  Mean (SD): 59.2 (28.7) (g) Carbohydrate Mean (SD): 53.5 (7.8) % of energy Fat Mean (SD): 31.5 (6.6) % of energy  Protein Amount at the end of the study Mean (SD): NR Carbohydrate Mean (SD): NR Fat Mean (SD): NR   **Quartile 3:** Protein intake 15.8–17.9% of energy  Baseline Protein Amount  Mean (SD): 64.5 (30.4) g Carbohydrate Mean (SD): 52.1 (7.5) % of energy Fat Mean (SD): 31.4 (6.6) % of energy  Protein Amount at the end of the study Mean (SD): NR Carbohydrate Mean (SD): NR Fat Mean (SD): NR   **Quartile 4:** Protein intake 18.0–33.5% of energy  Baseline Protein Amount  Mean (SD): 71.5 (35.1) g Carbohydrate Mean (SD): 49.6 (8.2) % of energy Fat Mean (SD): 30.7 (6.9) % of energy  Protein Amount at the end of the study Mean (SD): NR Carbohydrate Mean (SD): NR Fat Mean (SD): NR   Protein type/source: Mixed  Energy balance status: Eucaloric   Study duration: 5 y | **Protein Assessment Method:** A 120-item FFQ was used to assess usual food intake of specific foods and beverages over the past year. For each food item, the consumption frequency (times/d, week or month) and serving size (small, medium or large) were recorded. Protein intake was assessed at baseline. | **vBMD of the Axial Skeleton -** Volumetric bone mineral density (lumbar spine)  Measure/Method of Assessment: CT scan ([Imatron C-150 or a multi-detector CT system that utilized helical scanning with reconstruction in 5 mm thick cuts and 350 mm field of view]) |
| PMID: 17381900 **Key** **2007 (22)** Location/Country: Australia  HDI: Very high  Setting: Community dwelling Urban/ Rural: Urban Study design: Prospective cohort study Funding source: Government, nonprofit  **Risk of bias score: High** | Study of: Adults Total sample N: 34,696  **Arm 1:** Women  N: 26,749 % Female: 100% Mean Age (SD): 45.8 (13.1) y Race/ Ethnicity: NR Menopausal status: NR Obesity status: NR Mean BMI (SD): 23.6 (3.9) kg/m^2^ Income level: NR Education level: NR Physical activity level: 27.6% (≥ 3 hours vigorous exercise per week) Health status/ Comorbidities: NR Medication use: 13.5% hormone replacement therapy Supplement use: 61.2% take dietary supplements  Pregnant or lactating: NR  **Arm 2:** Men N: 7,947 % Female: 0% Mean Age (SD): 49.5 (13.5) y Race/ Ethnicity: NR Menopausal status: NA Obesity status: NR Mean BMI (SD): 24.2 (3.3) kg/m^2^ Income level: NR Education level: NR Physical activity level: 34.3% (≥ 3 hours vigorous exercise per week) Health status/ Comorbidities: NR Medication use: NR Supplement use: 44.2% take dietary supplements Pregnant or lactating: NA | **Arm 1:** Women  Baseline Protein Amount Mean (SD): 73.1 (21.6) g/d Carbohydrate Mean (SD): NR  Fat Mean (SD): NR  Protein Amount at the end of the study Mean (SD): NR Carbohydrate Mean (SD): NR Fat Mean (SD): NR   **Arm 2:** Men   Baseline Protein Amount Mean (SD): 77.8 (22.6) g/d Carbohydrate Mean (SD): NR  Fat Mean (SD): NR  Protein Amount at the end of the study Mean (SD): NR Carbohydrate Mean (SD): NR Fat Mean (SD): NR   Protein type/source: Plant  Energy balance status: Eucaloric   Study duration: 6 y | **Protein Assessment Method:** An FFQ was used to estimate participants average frequency intake of each of 130 foods and drinks. Nutrient intakes were estimated by multiplying the nutrient content of a specific portion size of each food by the frequency of consumption, using food composition tables. Protein intake was assessed over the previous 12 months. | **Osteoporotic Fractures and Fracture Risk -** Fragility fracture (osteoporotic and low-trauma fracture)Fracture Measure/method of assessment: Self-report |
| PMID: 26412291 **Langsetmo 2015 (23)** Location/Country: Canada HDI: Very high  Setting: Community dwelling Urban/ Rural: Urban Study design: Prospective cohort study Funding source: Government, industry, pharmaceutical  **Risk of bias score: High** | Study of: Adults  Total sample N: 6,510  **Arm 1:** Men N: 1,919 % Female: 0% Mean Age (SD): NR Race/ Ethnicity: NR Menopausal status: NA Obesity status: NR Mean BMI (SD): NR Income level: NR Education level: NR Physical activity level: NR Health status/ Comorbidities: NR Medication use: NR Supplement use: NR Pregnant or lactating: NA  **Arm 2:** Women N: 4,591 % Female: 100% Mean Age (SD): NR Race/ Ethnicity: NR Menopausal status: Premenopausal and postmenopausal Obesity status: NR Mean BMI (SD): NR Income level: NR Education level: NR Physical activity level: NR Health status/ Comorbidities: NR Medication use: NR Supplement use: NR Pregnant or lactating: NR | **Arm 1:** Men  Baseline Protein Amount Median (IQR): 13.6 (12.0-15.1) % of energy Carbohydrate Median (IQR): NR  Fat Median (IQR): NR  Protein Amount at the end of the study Median (IQR): NR Carbohydrate Median (IQR): NR Fat Median (IQR): NR   **Arm 2:** Women  Baseline Protein Amount Median (IQR): 14.3 (12.8-15.9) % of energy Carbohydrate Median (IQR): NR  Fat Median (IQR): NR  Protein Amount at the end of the study Median (IQR): NR Carbohydrate Median (IQR): NR Fat Median (IQR): NR  Protein type/source: Mixed   Energy Balance status: Eucaloric   Study duration: 5 y | **Protein Assessment Method:** A FFQ was derived from items on the short form Block questionnaire with modifications according to the Canadian diet. A standard portion size was specified with frequency ranging from never/ less than once a month to 6 or more times per day. Total energy intake (TEI) and protein intake were calculated by using the frequency and specified portion size from the questionnaire together with content information from the Canadian Nutrient File. Protein intake was assessed during Year 2 follow-up. | **BMD of the Appendicular Skeleton -** Bone mineral density (hip, total)   Measure/Method of Assessment: DXA (Hologic densitometers)  **BMD of the Axial Skeleton -** Bone mineral density (L1-L4 (lumbar spine))  Measure/Method of Assessment: DXA (Hologic densitometers)  **Osteoporotic Fractures and Fracture Risk –** Fragility fracture (osteoporotic and low-trauma fracture)  Measure/Method of Assessment: Self-report |
| PMID: 27943394 **Langsetmo**  **2017 (24)** Location/Country: USA HDI: Very high Setting: Community dwelling Urban/ Rural: Urban Study design: Prospective cohort study Funding source: Government **Risk of bias score: High** | Study of: Adults Total sample N: 5,875  **Quartile 1:** Protein intake 6.0-14.1% of energy N: 1,469 % Female: 0% Mean Age (SD): 73.6 (5.9) y Race/ Ethnicity: 87.3% non-Hispanic white Menopausal status: NA Obesity status: NR Mean BMI (SD): 27.3 (3.8) kg/m^2^ Income level: NR Education level:  Post-secondary degree: 45.3% Mean physical activity level: 147.9 PASE score Health status/ Comorbidities: Osteoporosis: 3.3%  Medication use: Corticosteroid medication: 2.3% Supplement use: Calcium/Vitamin D supplements: 32.5% Pregnant or lactating: NA  **Quartile 2:** Protein intake 14.2-15.8% of energy N: 1,469 % Female: 0% Mean Age (SD): 74.0 (5.8) y Race/ Ethnicity: 90.5% non-Hispanic white Menopausal status: NA Obesity status: NR Mean BMI (SD): 27.3 (3.6) kg/m^2^ Income level: NR Education level: Post-secondary degree: 52.0% Mean physical activity level: 145.0 PASE score Health status/ Comorbidities: Osteoporosis: 3.8%  Medication use: Corticosteroid medication: 2.0% Supplement use: Calcium/Vitamin D supplements: 36%  Pregnant or lactating: NA  **Quartile 3:** Protein intake 15.9-17.7% of energy N: 1,469 % Female: 0% Mean Age (SD): 73.6 (5.9) y Race/ Ethnicity: 91.0% non-Hispanic white Menopausal status: NA Obesity status: NR Mean BMI (SD): 27.4 (3.9) kg/m^2^ Income level: NR Education level:  Post-secondary degree: 57.3% Mean physical activity level: 149.0 PASE score Health status/ Comorbidities: Osteoporosis: 2.8% Medication use: Corticosteroid medication: 2.5% Supplement use: Calcium/Vitamin D supplements: 37.8% Pregnant or lactating: NA  **Quartile 4:** Protein intake 17.8-29.3% of energy N: 1,468 % Female: 0% Mean Age (SD): 73.4 (5.9) y Race/ Ethnicity: 90.2% non-Hispanic white Menopausal status: NA Obesity status: NR Mean BMI (SD): 27.5 (4.1) kg/m^2^ Income level: NR Education level:  Post-secondary degree: 58.6%  Mean physical activity level: 144.1 PASE score Health status/ Comorbidities: Osteoporosis: 4.2% Medication use: Corticosteroid medication: 1.6% Supplement use: Calcium/Vitamin D supplements: 36.7% Pregnant or lactating: NA | **Quartile 1:** Protein intake 6.0-14.1% of energy  Baseline Protein Amount  Range: 6.0-14.1% of energy Carbohydrate Range: NR  Fat Range: NR  Protein Amount at the end of the study Range: NR Carbohydrate Range: NR Fat Range: NR   **Quartile 2:** Protein intake 14.2-15.8% of energy  Baseline Protein Amount Range: 14.2-15.8% of energy Carbohydrate Range: NR  Fat Range: NR  Protein Amount at the end of the study Range: NR Carbohydrate Range: NR Fat Range: NR   **Quartile 3:** Protein intake 15.9-17.7% of energy  Baseline Protein Amount Range: 15.9-17.7% of energy Carbohydrate Range: NR  Fat Range: NR  Protein Amount at the end of the study Range: NR Carbohydrate Range: NR Fat Range: NR   **Quartile 4:** Protein intake 17.8-29.3% of energy  Baseline Protein Amount Range: 17.8-29.3% of energy Carbohydrate Range: NR  Fat Range: NR  Protein Amount at the end of the study Range: NR Carbohydrate Range: NR Fat Range: NR   Protein type/source: Mixed   Energy balance status: Eucaloric   Study duration: 15 y | **Protein Assessment Method:** Participants completed a modified version of the original Block FFQ. The FFQ asked 69 individual food item questions, including an additional 13 questions about food preparation and low-fat foods which were used to refine nutrient calculations. Total energy intake, total protein intake, and protein intake by source were derived from the responses to the questionnaire by Block Dietary Data Systems. Protein intake was assessed at baseline. | **Osteoporotic Fractures and Fracture Risk -** Incident fracture   Measure/Method of Assessment: Radiographic reports.  **Osteoporotic Fractures and Fracture Risk –** Fragility fracture (osteoporotic and low-trauma fracture)  Hip fracture Spine fracture  Measure/Method of Assessment: Radiographic reports  **BMD of the Appendicular Skeleton -** Bone mineral density (hip, total)   Measure/Method of Assessment: QDR 4500 fanbeam densitometers |
| PMID: 36986162 **Liu** **2023 (25)** Location/Country: China HDI: High  Setting: Community dwelling Urban/ Rural: Urban Study design: Prospective cohort study Funding source: Nonprofit, government, academic  **Risk of bias score: High** | Study of: Adults Total sample N: 1,987  **Quartile 1:** Protein intake <0.96 g of protein/kg/d N: 497 % Female: 65.8% Mean Age (SD): 60.3 (5.0) y Race/ Ethnicity: NR Menopausal status: NR Obesity status: NR Mean BMI (SD): 26.1 (3.1) kg/m^2^ Income level: 58.3% (income <3,000 Yuan (month-person) Education level: NR Mean physical activity level: 24.1 METS/wk Health status/ Comorbidities:  Stroke: 2.0% Hypertension: 29.4% Hyperlipidemia: 38.7%  Medication use: NR Supplement use:  Calcium supplements: 30.2%  Multivitamin supplements: 17.1%  Pregnant or lactating: NR  **Quartile 2:** Protein intake ~0.96 g of protein/kg/d N: 497 % Female: 69.8% Mean Age (SD): 60.4 (4.7) y Race/ Ethnicity: NR Menopausal status: NR Obesity status: NR Mean BMI (SD): 24.1 (2.4) kg/m^2^ Income level: 54.1% (income <3,000 Yuan (month-person) Education level: NR Mean physical activity level: 25.3 METS/wk Health status/ Comorbidities:  Stroke: 1.6%  Hypertension: 24.6%  Hyperlipidemia: 41.7%  Medication use: NR Supplement use: Calcium supplements: 27.8% Multivitamin supplements: 17.5%  Pregnant or lactating: NR  **Quartile 3:** Protein intake 1.10~ g of protein/kg/d N: 497 % Female: 72.2% Mean Age (SD): 60.3 (4.8) y Race/ Ethnicity: NR Menopausal status: NR Obesity status: NR Mean BMI (SD): 22.8 (2.3) kg/m^2^ Income level: 56.1% (income <3,000 Yuan (month-person) Education level: NR Mean physical activity level: 25.2 METS/wk Health status/ Comorbidities:  Stroke: 1.8% Hypertension: 25.5%  Hyperlipidemia: 38.0%  Medication use: NR Supplement use:  Calcium supplements: 30.8%  Multivitamin supplements: 22.3%  Pregnant or lactating: NR  **Quartile 4:** Protein intake ≥1.26 g of protein/kg/d N: 496 % Female: 78.8% Mean Age (SD): 60.1 (5.1) y Race/ Ethnicity: NR Menopausal status: NR Obesity status: NR Mean BMI (SD): 21.1 (2.4) kg/m^2^ Income level: 62.3% (income <3,000 Yuan (month-person) Education level: NR Mean physical activity level: 25.7 METS/wk Health status/ Comorbidities:  Stroke: 1.8% Hypertension: 25.5% Hyperlipidemia: 39.3% Medication use: NR Supplement use:  Calcium supplements: 30.6%  Multivitamin supplements: 22.0% Pregnant or lactating: NR | **Quartile 1:** Protein intake <0.96 g of protein/kg/d  Baseline Protein Amount Mean (SD): NR Carbohydrate Mean (SD): NR  Fat Mean (SD): NR  Protein Amount at the end of the study Mean (SD): 14.9 (4.7) % of energy  Carbohydrate Mean (SD): 57.6 (22.1) % of energy Fat Mean (SD): 21.2 (14.4) % of energy  **Quartile 2:** Protein intake 0.96~ g of protein/kg/d  Baseline Protein Amount Mean (SD): NR Carbohydrate Mean (SD): NR  Fat Mean (SD): NR  Protein Amount at the end of the study Mean (SD): 16.9 (4.9) % of energy Carbohydrate Mean (SD): 59.1 (25.4) % of energy Fat Mean (SD): 32.1 (16.3) % of energy  **Quartile 3:** Protein intake ~1.10 g of protein/kg/d  Baseline Protein Amount Mean (SD): NR Carbohydrate Mean (SD): NR  Fat Mean (SD): NR  Protein Amount at the end of the study Mean (SD): 17.8 (5.5) % of energy Carbohydrate Mean (SD): 58.3 (24.9) % of energy Fat Mean (SD): 31.6 (14.8) % of energy  **Quartile 4:** Protein intake ≥1.26 g of protein/kg/d  Baseline Protein Amount Mean (SD): NR Carbohydrate Mean (SD): NR  Fat Mean (SD): NR  Protein Amount at the end of the study Mean (SD): 20.3 (6.6) % of energy Carbohydrate Mean (SD): 61.2 (25.8) % of energy Fat Mean (SD): 32.5 (15.6) % of energy  Protein type/source: Mixed  Energy balance status: Eucaloric  Study duration: 6 y | **Protein Assessment Method:** Dietary intake was assessed using a validated 79-item FFQ to estimate habitual food intakes. For each food item, its frequency (never or per year, month, week, or day) of consumption and the regular serving size were estimated. The dietary intake of total energy, protein, amino acids, and other nutrients was calculated according to the Chinese Food Composition Table 2009. Protein intake was assessed at the first follow-up. | **Total Body BMD -** Bone mineral density (total body)  Measure/Method of Assessment: DXA (Hologic QDR1000, version 6.10)  **BMD of the Axial Skeleton -** Bone mineral density ( L1-L4 (lumbar spine))  Measure/Method of Assessment: DXA (Hologic QDR1000, version 6.10)  **BMD of the Appendicular Skeleton -** Bone mineral density (hip, total)  Measure/Method of Assessment: DXA (Hologic QDR1000, version 6.10)  **BMD of the Appendicular Skeleton -** Bone mineral density (femoral neck)  Measure/Method of Assessment: DXA (Hologic QDR1000, version 6.10)  **BMD of the Appendicular Skeleton -** Bone mineral density (trochanter)   Measure/Method of Assessment: DXA (Hologic QDR1000, version 6.10) |
| PMID: 28179224 **Mangano** **2017^1^ (26)** Location/Country: USA HDI: Very high Setting: NR Urban/ Rural: NR Study design: Prospective cohort study Funding source: Government **Risk of bias score: High** | Study of: Adults  Total sample N: 2,986   **Arm 1:** Protein food cluster (Fast food, full-fat dairy) N: 458 % Female: 44% Mean Age (SD): 39.3 (8.5) y Race/ Ethnicity: NR Menopausal status: 6% nonestrogenic  Obesity status: NR Mean BMI (SD): 26.5 (5.0) kg/m^2^ Income level: NR Education level: NR Mean physical activity level: 37.2 PAI Health status/ Comorbidities: NR Medication use: NR Supplement use: Calcium supplements: 19%, Vitamin D supplements: 40% Pregnant or lactating: NR   **Arm 2:** Protein food cluster 2 (Fish) N: 605 % Female: 58% Mean Age (SD): 42.2 (9.0) y Race/ Ethnicity: NR Menopausal status: 14% nonestrogenic Obesity status: NR Mean BMI (SD): 26.8 (5.3) kg/m^2^ Income level: NR Education level: NR Mean physical activity level: 37.4 PAI Health status/ Comorbidities: NR Medication use: NR Supplement use:  Calcium supplements: 43%, Vitamin D supplements: 53% Pregnant or lactating: NR   **Arm 3:** Protein food cluster 3 (Red meat) N: 640  % Female: 48% Mean Age (SD): 41.5 (8.3) y Race/ Ethnicity: NR Menopausal status: 13% nonestrogenic Obesity status: NR Mean BMI (SD): 27.4 (5.6) kg/m^2^ Income level: NR Education level: NR Mean physical activity level: 37.5 PAI Health status/ Comorbidities: NR Medication use: NR Supplement use: Calcium supplements: 30% Vitamin D supplements: 39% Pregnant or lactating: NR   **Arm 4:** Protein food cluster 4 (Chicken) N: 735 % Female: 58% Mean Age (SD): 39.3 (8.3) y Race/ Ethnicity: NR Menopausal status: 7% nonestrogenic Obesity status: NR Mean BMI (SD): 26.7 (5.3) kg/m^2^ Income level: NR Education level: NR Mean physical activity level: 37.0 PAI Health status/ Comorbidities: NR Medication use: NR Supplement use: Calcium supplements: 36%, Vitamin D supplements: 46% Pregnant or lactating: NR   **Arm 5:** Protein food cluster 5 (Low-fat milk) N: 434 % Female: 58% Mean Age (SD): 40.9 (8.6) y Race/ Ethnicity: NR Menopausal status: 11% nonestrogenic Obesity status: NR Mean BMI (SD): 26.8 (5.0) kg/m^2^ Income level: NR  Education level: NR Mean physical activity level: 37.8 PAI Health status/ Comorbidities: NR Medication use: NR Supplement use:  Calcium supplements: 40%, Vitamin D supplements: 50% Pregnant or lactating: NR   **Arm 6:** Protein food cluster 6 (Legumes)  N: 114 % Female: 79% Mean Age (SD): 38.6 (9.4) y Race/ Ethnicity: NR Menopausal status: 7% nonestrogenic Obesity status: NR Mean BMI (SD): 23.9 (4.6) kg/m^2^ Income level: NR Education level: NR Mean physical activity level: 36.1 PAI Health status/ Comorbidities: NR Medication use: NR Supplement use: Calcium supplements: 47%, Vitamin D supplements: 56% Pregnant or lactating: NR | **Arm 1:** Protein food cluster (Fast food, full-fat dairy)   Baseline Protein Amount Mean (SD): 88 (31) g/d Carbohydrate Mean (SD): NR Fat Mean (SD): NR   Protein Amount at the end of the study Mean (SD): NR Carbohydrate Mean (SD): NR Fat Mean (SD): NR    **Arm 2:** Protein food cluster 2 (Fish)   Baseline Protein Amount Mean (SD): 90 (31) g/d Carbohydrate Mean (SD): NR  Fat Mean (SD): NR   Protein Amount at the end of the study Mean (SD): NR Carbohydrate Mean (SD): NR Fat Mean (SD): NR    **Arm 3:** Protein food cluster 3 (Red meat)   Baseline Protein Amount Mean (SD): 97 (29) g/d Carbohydrate Mean (SD): NR Fat Mean (SD): NR   Protein Amount at the end of the study Mean (SD): NR Carbohydrate Mean (SD): NR Fat Mean (SD): NR    **Arm 4:** Protein food cluster 4 (Chicken)   Baseline Protein Amount Mean (SD): 95 (35) g/d Carbohydrate Mean (SD): NR Fat Mean (SD): NR   Protein Amount at the end of the study Mean (SD): NR Carbohydrate Mean (SD): NR Fat Mean (SD): NR    **Arm 5:** Protein food cluster 5 (Low-fat milk)   Baseline Protein Amount Mean (SD): 98 (31) g/d Carbohydrate Mean (SD): NR Fat Mean (SD): NR   Protein Amount at the end of the study Mean (SD): NR Carbohydrate Mean (SD): NR Fat Mean (SD): NR    **Arm 6:** Protein food cluster 6 (Legumes)   Baseline Protein Amount Mean (SD): 83 (34) g/d Carbohydrate: NR Fat: NR   Protein Amount at the end of the study Mean (SD): NR Carbohydrate Mean (SD): NR Fat Mean (SD): NR    Protein type/source: Mixed   Energy balance status: Eucaloric    Study duration: 9 y | **Protein Assessment Method:** Typical dietary intakes of foods and nutrients were assessed with the use of the Harvard 126-item semiquantitative and validated general population 88 FFQ. Protein intake was assessed during the years 2002-2005. | **BMD of the Appendicular Skeleton -** Bone mineral density (femoral neck)  Measure/Method of Assessment: GE Lunar Prodigy fan-beam densitometer   **BMD of the Appendicular Skeleton -** Bone mineral density (hip, total)   Measure/Method of Assessment: GE Lunar Prodigy fan-beam densitometer   **BMD of the Appendicular Skeleton -** Bone mineral density (trochanter)  Measure/Method of Assessment: GE Lunar Prodigy fan-beam densitometer   **BMD of the Axial Skeleton -** Bone mineral density (lumbar spine)   Measure/Method of Assessment: GE Lunar Prodigy fan-beam densitometer |
| PMID: 19419320 **Meng** **2009^1^ (27)** Location/Country: Australia HDI: Very high Setting: Community dwelling Urban/ Rural: NR Study design: Prospective cohort study Funding source: Nonprofit, government  **Risk of bias score: High** | Study of: Adults Total sample N: 862   **Tertile 1:** Protein intake <66 g/ d N: 287 % Female: 100% Mean Age (SD): 74.9 (2.5) y Race/ Ethnicity: 100% white origin Menopausal status: Postmenopausal  Obesity status: NR Mean BMI (SD): 26.4 (4.2) kg/m^2^ Income level: NR Education level: NR Physical activity level: 466 (median kilojoules expended per day) Health status/ Comorbidities: Participants were excluded if they had a medical condition likely to influence 5-year survival.  Medication use: Participants were excluded if they were taking bone active medications including calcium supplements, estrogen, bisphosphonates, and vitamin D.  Supplement use: Participants were excluded if they were taking bone active medications including calcium supplements, estrogen, bisphosphonates, and vitamin D. Pregnant or lactating: NR   **Tertile 2:** Protein intake 66-87 g/d N: 287 % Female: 100% Mean Age (SD): 75.0 (2.6) y  Race/ Ethnicity: 100% white origin Menopausal status: Postmenopausal Obesity status: NR Mean BMI (SD): 26.7 (4.7) kg/m^2^ Income level: NR Education level: NR Physical activity level: 530 (median kilojoules expended per day) Health status/ Comorbidities: Participants were excluded if they had a medical condition likely to influence 5-year survival. Medication use: Participants were excluded if they were taking bone active medications including calcium supplements, estrogen, bisphosphonates, and vitamin D. Supplement use: Participants were excluded if they were taking bone active medications including calcium supplements, estrogen, bisphosphonates, and vitamin D. Pregnant or lactating: NR   **Tertile 3:** Protein intake >87 g/d N: 288 % Female: 100%  Mean Age (SD): 74.7 (2.7) y Race/ Ethnicity: 100% white origin Menopausal status: Postmenopausal Obesity status: NR Mean BMI (SD): 27.3 (4.3) kg/m^2^ Income level: NR Education level: NR Physical activity level: 614 (median kilojoules expended per day) Health status/ Comorbidities: Participants were excluded if they had a medical condition likely to influence 5-year survival. Medication use: Participants were excluded if they were taking bone active medications including calcium supplements, estrogen, bisphosphonates, and vitamin D. Supplement use: Participants were excluded if they were taking bone active medications including calcium supplements, estrogen, bisphosphonates, and vitamin D. Pregnant or lactating: NR | **Tertile 1:** Protein intake <66 g/d   Baseline Protein Amount Mean (SD): 54.4 (9.1) g/d Carbohydrate Mean (SD): 146.8 (30.9) g/d Fat Mean (SD): 46.4 (13.3) g/d   Protein Amount at the end of the study Mean (SD): NR Carbohydrate Mean (SD): NR Fat Mean (SD): NR    **Tertile 2:** Protein intake 66-87 g/d   Baseline Protein Amount Mean (SD): 76.6 (6.2) g/d Carbohydrate Mean (SD): 186.4 (34.1) g/d Fat Mean (SD): 63.0 (13.3) g/d   Protein Amount at the end of the study Mean (SD): NR Carbohydrate Mean (SD): NR Fat Mean (SD): NR    **Tertile 3:** Protein intake >87 g/d   Baseline Protein Amount Mean (SD): 110.9 (23.4) g/d Carbohydrate Mean (SD): 249.5 (61.9) g/d Fat Mean (SD): 85.1 (25.7) g/d   Protein Amount at the end of the study Mean (SD): NR Carbohydrate Mean (SD): NR Fat Mean (SD): NR    Protein type/source: Mixed   Energy balance status: Eucaloric    Study duration: 5 years | **Protein Assessment Method:** Participants completed a self-administered, quantitative FFQ. This FFQ has been designed to measure eating habits over the past 12-mo period and calibrated and validated according to the foods and on intake for a 12-mo period. The daily dietary intakes were derived from the questionnaire. Protein intake was assessed at baseline. | **Total Body BMC -** Bone mineral content (total body)   Measure/Method of Assessment: DXA (Hologic 4500A) |
| PMID: 20442986 **Misra** **2011 (28)** Location/country: USA HDI: Very high Setting: Community dwelling Urban/ Rural: Urban Study design: Prospective cohort study Funding source: Government **Risk of bias score: High** | Study of: Adults Total sample N: 946  **Arm 1:** No hip fracture N: 846 % Female: 58.6% Mean Age (SD): 75 (5.0) y Race/ Ethnicity: NR Menopausal status: NR Obesity status: NR Mean BMI (SD): NR Income level: NR Education level: NR Mean physical activity level: 33 PAI score Health status/ Comorbidities: NR Medication use: NR Supplement use: NR Pregnant or lactating: NR  **Arm 2:** Hip fracture N: 100 % Female: 80.0% Mean Age (SD): 76 (5.2) y Race/ Ethnicity: NR Menopausal status: NR Obesity status: NR Mean BMI (SD): NR Income level: NR Education level: NR Mean physical activity level: 34 PAI score Health status/ Comorbidities: NR Medication use: NR Supplement use: NR Pregnant or lactating: NR | **Arm 1:** No hip fracture   Baseline Protein Amount Mean (SD): 64.2 g/d Carbohydrate Mean (SD): NR  Fat Mean (SD): NR  Protein Amount at the end of the study Mean (SD): NR Carbohydrate Mean (SD): NR Fat Mean (SD): NR   **Arm 2:** Hip fracture  Baseline Protein Amount Mean (SD): 63.6 g/d Carbohydrate Mean (SD): NR  Fat Mean (SD): NR  Protein Amount at the end of the study Mean (SD): NR Carbohydrate Mean (SD): NR Fat Mean (SD): NR   Protein type/source: Mixed   Energy balance status: Eucaloric   Study duration: 16-17 y | **Protein Assessment Method:** FFQ was used to assess usual dietary intake by self-report. Total protein intake (g/day) was adjusted for total energy (from FFQ) to reduce error due to variation in total energy requirement, body size, and portion sizes, allowing interpretation of the effect of total protein intake. Protein intake was assessed at baseline. | **Osteoporotic Fractures and Fracture Risk -** Hip fracture   Measure/Method of Assessment: Self-report |
| PMID: 36715763 **Nakano** **2023 (29)** Location/Country: Japan HDI: Very high Setting: NR Urban/ Rural: Urban Study design: Prospective cohort study Funding source: Government **Risk of bias score: High** | Study of: Adults Total sample N: 1,070  **Arm 1:** Whole cohort  N: 1,070 % Female: 100% Mean Age (SD): 69.3 (10.9) y Race/ Ethnicity: NR Menopausal status: Postmenopausal  Obesity status: NR Mean BMI (SD): 22.6 (3.4) kg/m^2^ Income level: NR Education level: NR Physical activity level: NR Health status/ Comorbidities: Participants with critical or acute illness (e.g., terminal cancer, cardiovascular disease, or infectious diseases such as pneumonia) or secondary osteoporosis (e.g., due to primary hyperparathyroidism, end-stage renal failure, or long-term steroid use) were excluded.  Diabetes: 14.3% Dyslipidemia: 48.0% Hypertension: 58.1% Medication use: Participants under any kind of treatment for primary osteoporosis were included. Participants with long-term steroid use were excluded.  Supplement use: NR Pregnant or lactating: NR | **Arm 1:** Whole cohort   Baseline Protein Amount Mean (SD): 73.4 (15.1) g/d Carbohydrate Mean (SD): 200 (39) g/d Fat Mean (SD): 59.5 (12.2) g/d  Protein Amount at the end of the study Mean (SD): NR Carbohydrate Mean (SD): NR Fat Mean (SD): NR   Protein type/source: Mixed  Energy balance status: Eucaloric  Study duration: 5.8 y | **Protein Assessment Method:** Dietary nutrient intake was assessed by using an FFQ method for the prevention and management of osteoporosis (FFQPOP). The FFQPOP comprised a total of 28 food items. Subjects were asked to select the grade of intake frequency in the previous 1 month for each item. Nutrients and energy intakes were estimated by the frequency grade and relevant coefficients determined on the basis of the Standard Tables of Food Composition in Japan. Protein intake was assessed at baseline. | **Osteoporotic Fractures and Fracture Risk -** Fragility fracture (osteoporotic fracture)  Measure/Method of Assessment: X-ray films |
| PMID: 11914191 **Promislow 2002 (30)** Location/Country: USA HDI: Very high  Setting: Community dwelling Urban/ Rural: Urban Study design: Prospective cohort study Funding source: Government **Risk of bias score: High** | Study of: Adults Total sample N: 960  **Arm 1:** Women N: 572 % Female: 100% Mean Age (SD): 71.2 (8.7) y Race/ Ethnicity: Caucasian  Menopausal status: Postmenopausal Obesity status: NR Mean BMI (SD): 24.6 (3.7) kg/m^2^ Income level: NR Education level: NR Physical activity level: Exercise ≥3x per week: 70.9% Health status/ Comorbidities: NR Medication use:  Thiazides: 24.7% Thyroid hormones: 21.0% Steroids: 3.9% Estrogen: 39.9% Supplement use: NR Pregnant or lactating: NR  **Arm 2:** Men  N: 388 % Female: 0% Mean Age (SD): 70.0 (8.5) y Race/ Ethnicity: Caucasian Menopausal status: NA Obesity status: NR Mean BMI (SD): 26.4 (3.4) kg/m^2^ Income level: NR Education level: NR Physical activity level: Exercise ≥3x per week: 78.9% Health status/ Comorbidities: NR Medication use: Thiazides: 15.0% Thyroid hormones: 4.4% Steroids: 2.1% Supplement use: NR Pregnant or lactating: NA | **Arm 1:** Women  Baseline Protein Amount Mean (SD): 71.2 (24.8) g/d Carbohydrate Mean (SD): NR Fat Mean (SD): NR  Protein Amount at the end of the study Mean (SD): NR Carbohydrate Mean (SD): NR Fat Mean (SD): NR   **Arm 2:** Men   Baseline Protein Amount Mean (SD): 73.8 (23.4) g/d Carbohydrate Mean (SD): NR Fat Mean (SD): NR  Protein Amount at the end of the study Mean (SD): NR Carbohydrate Mean (SD): NR Fat Mean (SD): NR   Protein type/source: Mixed  Energy Balance status: Eucaloric   Study duration: 4 y | **Protein Assessment Method:** Harvard-Willett diet assessment questionnaire was used to collect information on dietary intake. The questionnaire was self-administered and contained questions regarding portion size and consumption frequency of 128 common food items. Protein intake was assessed at baseline. | **BMD of the Appendicular Skeleton -** Bone mineral density (hip, total)  Measure/Method of Assessment: DXA (Hologic QDR, model 1000)  **BMD of the Appendicular Skeleton -** Bone mineral density (femoral neck)   Measure/Method of Assessment: DXA (Hologic QDR, model 1000)  **BMD of the Axial Skeleton -** Bone mineral density (lumbar spine)  Measure/Method of Assessment: DXA (Hologic QDR, model 1000) |
| PMID: 33847345 **Rivera-Paredez** **2021 (31)** Location/Country: Mexico HDI: High  Setting: Community dwelling Urban/ Rural: Urban Study design: Prospective cohort study Funding source: Government **Risk of bias score: Moderate** | Study of: Adults Total sample N: NR  **Arm 1**: Whole Cohort  N: NR % Female: 100% Mean Age (SD): 57 y Race/ Ethnicity: NR Menopausal status: Postmenopausal  Obesity status: 26.5%  Mean BMI (SD): 27.1 kg/m^2^ Income level: NR Education level: NR  Mean physical activity level: 13.0 (leisure time physical activity, min/d) Health status/ Comorbidities: NR Medication use: Hormone replacement therapy: 7.8% Supplement use: 25.2% dietary supplement  Pregnant or lactating: NA | **Arm 1**: Whole Cohort  Baseline Protein Amount Median (IQR): 66.4 (51.1-86.0) g/d Carbohydrate Median (IQR): NR  Fat Median (IQR): NR  Protein Amount at the end of the study Median (IQR): 50.1 (37.8- 66.9 g/d Carbohydrate Median (IQR): NR Fat Median (IQR): NR   Protein type/source: Mixed   Energy balance status: Eucaloric  Study duration: 6.4 y | **Protein Assessment Method:** A semi-quantitative FFQ was used to collect data on the consumption frequency of 116 food items during the previous year. Average daily nutrient intake was calculated by multiplying the frequency of consumption of each food by the nutrient content. Protein intake was assessed at baseline. | **BMD of the Axial Skeleton -** Bone mineral density (L1-L4 (lumbar spine))   Measure/Method of Assessment: DXA Lunar DPX NT instrument (Lunar Radiation Corp.)  **BMD of the Appendicular Skeleton -** Bone mineral density (femoral neck)  Measure/Method of Assessment: DXA Lunar DPX NT instrument (Lunar Radiation Corp.)  **BMD of the Appendicular Skeleton -** Bone mineral density (hip, total) Measure/Method of Assessment: DXA Lunar DPX NT instrument (Lunar Radiation Corp.) |
| PMID: 20662074 **Sahni** **2010 (32)** Location/Country: USA HDI: Very high Setting: NR Urban/ Rural: NR Study design: Prospective cohort study Funding source: Government **Risk of bias score: High** | Study of: Adults Total sample N: 3,656  **Arm 1:** Men  N: 1,725 % Female: 0% Mean Age (SD): 55.3 (9.9) y Race/ Ethnicity: NR Menopausal status: NA Obesity status: NR Mean BMI (SD): 28.1 (4.1) kg/m^2^ Income level: NR Education level: NR Physical activity level: NR Health status/ Comorbidities: NR Medication use: NR Supplement use:  Calcium supplements: 13.0% Pregnant or lactating: NA  **Arm 2:** Women  N: 1,931 % Female: 100% Mean Age (SD): 54.9 (9.8) y Race/ Ethnicity: NR Menopausal status: 68.9% postmenopausal Obesity status: NR Mean BMI (SD): 26.8 (5.5) kg/m^2^ Income level: NR Education level: NR Physical activity level: NR Health status/ Comorbidities: NR Medication use: NR Supplement use:  Calcium supplements: 29.2% Pregnant or lactating: NR | **Arm 1:** Men  Baseline Protein Amount Mean (SD): 79.0 (27) g/d Carbohydrate Mean (SD): NR Fat Mean (SD): NR  Protein Amount at the end of the study Mean (SD): NR Carbohydrate Mean (SD): NR Fat Mean (SD): NR   **Arm 2:** Women   Baseline Protein Amount Mean (SD): 75.7 (27) g/d Carbohydrate Mean (SD): NR Fat Mean (SD): NR   Protein Amount at the end of the study Mean (SD): NR Carbohydrate Mean (SD): NR Fat Mean (SD): NR   Protein type/source: Mixed  Energy balance status: Eucaloric  Study duration: 7-10 y | **Protein Assessment Method:** Usual dietary intake was assessed with the semi-quantitative 126-item Willett FFQ. Intakes of total protein (g/day), plant protein (g/day), and animal protein (g/day) were assessed using the food list section of the FFQ. Animal/plant protein intake ratio was calculated. Protein intake was assessed at baseline. | **Osteoporotic Fractures and Fracture Risk -** Hip fracture   Measure/Method of Assessment: Self-report & confirmed by review of medical records |
| PMID: 24168918 **Sahni** **2014 (33)** Location/Country: USA HDI: Very high  Setting: NR Urban/ Rural: NR Study design: Prospective cohort study Funding source: Government **Risk of bias score: High** | Study of: Adults  Total sample N: 1,175  **Arm 1:** Men N: 495 % Female: 0% Mean Age (SD): 61 (9.0) y Race/ Ethnicity: NR Menopausal status: NA Obesity status: NR Mean BMI (SD): 28.8 (4.4) kg/m^2^ Income level: NR Education level: NR Physical activity level: NR Health status/ Comorbidities: NR Medication use: Osteoporosis medication: 0.2% Supplement use: 24% used Calcium supplements: 24% Vitamin D supplements: 40% Pregnant or lactating: NA  **Arm 2:** Women N: 680 % Female: 100% Mean Age (SD): 60 (9.2) y Race/ Ethnicity: NR Menopausal status: 86% postmenopausal Obesity status: NR Mean BMI (SD): 27.4 (5.6) kg/m^2^ Income level: NR Education level: NR Physical activity level: NR Health status/ Comorbidities: NR Medication use: Osteoporosis medication: 3.6% Supplement use: Calcium supplements: 56% Vitamin D supplements: 53% Pregnant or lactating: NR | **Arm 1:** Men  Baseline Protein Amount Mean (SD): 81 (28) g/d Carbohydrate Mean (SD): NR Fat Mean (SD): NR   Protein Amount at the end of the study Mean (SD): NR Carbohydrate Mean (SD): NR Fat Mean (SD): NR   **Arm 2:** Women   Baseline Protein Amount Mean (SD): 77 (26) g/d Carbohydrate Mean (SD): NR Fat Mean (SD): NR   Protein Amount at the end of the study Mean (SD): NR Carbohydrate Mean (SD): NR Fat Mean (SD): NR   Protein type/source: Mixed  Energy balance status: Eucaloric   Study duration: 1.5-8 y | **Protein Assessment Method:** Usual dietary intake was assessed with a semi-quantitative, 126-item Willett FFQ. Intakes of total protein (g/d) were assessed using the food list section of the FFQ. Protein intake was assessed at baseline. | **BMD of the Appendicular Skeleton -** Bone mineral density (hip, total)  Measure/Method of Assessment: DXA (LUNAR DPX-L)  **BMD of the Axial Skeleton -** Bone mineral density (lumbar spine)  Measure/Method of Assessment: DXA (LUNAR DPX-L) |
| PMID: 11124760 **Sellmeyer** **2001 (34)** Location/Country: USA HDI: Very high Setting: Community dwelling Urban/ Rural: NR Study design: Prospective cohort study) Funding source: Government **Risk of bias score: High** | Study of: Adults Total sample N: 1,035  **Tertile 1:** Low ratio of animal to vegetable protein N: NR % Female: 100% Mean Age (SD): 74.3 (5.4) y Race/ Ethnicity: 100% white Menopausal status: Postmenopausal  Obesity status: NR Mean BMI (SD): 25.6 (4.6) kg/m^2^ Income level: NR Education level: NR Physical activity level: NR Health status/ Comorbidities: NR Medication use: NR Supplement use: NR Pregnant or lactating: NR  **Tertile 2:** Medium ratio of animal to vegetable protein N: NR % Female: 100% Mean Age (SD): 73.2 (4.9) y Race/ Ethnicity: 100% white Menopausal status: Postmenopausal Obesity status: NR Mean BMI (SD): 26.5 (4.7) kg/m^2^ Income level: NR Education level: NR Physical activity level: NR Health status/ Comorbidities: NR Medication use: NR Supplement use: NR Pregnant or lactating: NR  **Tertile 3:** High ratio of animal to vegetable protein N: NR % Female: 100% Mean Age (SD): 72.5 (4.5) y Race/ Ethnicity: 100% white Menopausal status: Postmenopausal Obesity status: NR Mean BMI (SD): 26.7 (4.9) kg/m^2^ Income level: NR Education level: NR Physical activity level: NR Health status/ Comorbidities: NR Medication use: NR Supplement use: NR Pregnant or lactating: NR | **Tertile 1:** Low ratio of animal to vegetable protein  Baseline Protein Amount Mean (SD): NR Carbohydrate Mean (SD): NR Fat Mean (SD): NR  Protein Amount at the end of the study Mean (SD): 42.0 (15.9) g Carbohydrate Mean (SD): NR Fat Mean (SD): NR   **Tertile 2:** Medium ratio of animal to vegetable protein  Baseline Protein Amount Mean (SD): NR Carbohydrate Mean (SD): NR Fat Mean (SD): NR  Protein Amount at the end of the study Mean (SD): 49.2 (16.9) g Carbohydrate Mean (SD): NR Fat Mean (SD): NR   **Tertile 3:** High ratio of animal to vegetable protein  Baseline Protein Amount Mean (SD): NR Carbohydrate Mean (SD): NR Fat Mean (SD): NR  Protein Amount at the end of the study Mean (SD): 58.3 (20.0) g Carbohydrate Mean (SD): NR Fat Mean (SD): NR   Protein type/source: Mixed  Energy balance status: Eucaloric   Study duration: 7 y | **Protein Assessment Method:** Nutrient intake data were obtained from a 24-h dietary recall and an FFQ. The 24-h dietary recall consisted in registering all the meals and beverages consumed during the 24 h before the subject awoke on the day of the interview, assessing, for each food item, the portion sizes and their weight correspondence according to a book of photographs. Food intake data were estimated from a detailed FFQ, the frequency of consumption of 148 foods and nonalcoholic beverages for each of the three main meals and three between-meals snacks was recorded in 11 classes. Protein intake was assessed at baseline. | **BMD of the Appendicular Skeleton -** Bone mineral density (hip and subregions)  Measure/Method of Assessment: DXA (Hologic QDR-1000, version 6.10)  **Osteoporotic Fractures and Fracture Risk -** Hip fracture   Measure/Method of Assessment: Self-report & confirmed with radiographs |
| PMID: 33677533 **Weaver** **2021 (35)** Location/Country: USA HDI: Very high Setting: Community dwelling Urban/ Rural: Urban Study design: Prospective cohort study Funding source: Government **Risk of bias score: High** | Study of: Adults Total sample N: 2,160  **Tertile 1:** Protein intake <13% of energy N: 718 % Female: 47.0% Mean Age (SD): 73.5 (2.9) y Race/ Ethnicity: Black: 47.0% Menopausal status: NR Obesity status: NR Mean BMI (SD): 27.1 (4.7) kg/m^2^ Income level: NR Education level:  Less than high school: 23.3% High school: 35.1% Postsecondary education: 41.7% Physical activity level:  0 min walking/wk: 43.9%  1–149 min walking/wk: 29.3% >150 min walking/wk: 26.8% Health status/ Comorbidities: Participants free of life-threatening illness were included  Medication use: Osteoporosis medication: 3.4% Supplement use: 15.0% Calcium supplements: 15.0% Vitamin D supplements: 6.6% Pregnant or lactating: NR  **Tertile 2:** Protein intake 13-15% of energy N: 703 % Female: 52.2% Mean Age (SD): 73.4 (2.8) y Race/ Ethnicity: Black: 52.2% Menopausal status: NR Obesity status: NR Mean BMI (SD): 27.0 (4.4) kg/m^2^ Income level: NR Education level:  Less than high school: 21.7% High school: 31.2% Postsecondary education: 47.1% Physical activity level:  0 min walking/wk: 40.1%  1–149 min walking/wk: 33.8% >150 min walking/wk: 26.2% Health status/ Comorbidities: Participants free of life-threatening illness were included Medication use: Osteoporosis medication: 5.2% Supplement use:  Calcium supplements: 19.7% Vitamin D supplements: 9.8% Pregnant or lactating: NR  **Tertile 3:** Protein intake >15% of energy N: 739 % Female: 55.3% Mean Age (SD): 73.7 (2.9) y Race/ Ethnicity:  Black: 55.3% Menopausal status: NR Obesity status: NR Mean BMI (SD): 27.5 (4.8) kg/m^2^ Income level: NR Education level:  Less than high school: 19.8% High school: 33.6% Postsecondary education: 46.6% Physical activity level: 0 min walking/wk: 37.0%  1–149 min walking/wk: 30.3% >150 min walking/wk: 32.7% Health status/ Comorbidities: Participants free of life-threatening illness were included. Medication use:  Osteoporosis medication: 5.2% Supplement use:  Calcium supplements: 24.0% Vitamin D supplements: 10.5% Pregnant or lactating: NR | **Tertile 1:** Protein intake <13% of energy  Baseline Protein Amount Mean (SD): 12 (1) % of energy Carbohydrate Mean (SD): 55 (8) % of energy Fat Mean (SD): 34 (7) % of energy  Protein Amount at the end of the study Mean (SD): NR Carbohydrate Mean (SD): NR Fat Mean (SD): NR   **Tertile 2:** Protein intake 13-15% of energy  Baseline Protein Amount Mean (SD): 14 (1) % of energy Carbohydrate Mean (SD): 53 (8) % of energy Fat Mean (SD): 34 (7) % of energy  Protein Amount at the end of the study Mean (SD): NR Carbohydrate Mean (SD): NR Fat Mean (SD): NR   **Tertile 3:** Protein intake >15% of energy  Baseline Protein Amount Mean (SD): 18 (2) % of energy Carbohydrate Mean (SD): 52 (8) % of energy Fat Mean (SD): 32 (8) % of energy  Protein Amount at the end of the study Mean (SD): NR Carbohydrate Mean (SD): NR Fat Mean (SD): NR   Protein type/source: Mixed  Energy balance status: Eucaloric   Study duration: 5 y | **Protein Assessment Method:** Participants completed a 108-item, interviewer-administered modified version of the FFQ. Wood blocks, food models, standard kitchen measures, and flash cards were used by trained interviewers to assist participants in estimating food portion sizes. Energy intake and macronutrient and micronutrient content were calculated from the FFQ by Block Dietary Data Systems (Berkeley, CA). Total protein intake, as well as the source of protein (e.g., animal or vegetable), was computed. Protein intake was assessed one year from baseline. | **BMD of the Appendicular Skeleton -** Bone mineral density (hip, total)  Measure/Method of Assessment: DXA (Hologic 4500A, software v.9.03)  **BMD of the Appendicular Skeleton -** Bone mineral density (femoral neck)  Measure/Method of Assessment: DXA (Hologic 4500A, software v.9.03)  **aBMD of the total body –** Areal bone mineral density (total body)   Measure/Method of Assessment: DXA (Hologic 4500A, software v.9.03)  **Osteoporotic Fractures and Fracture Risk -** Fragility fracture (low-trauma)  Measure/Method of Assessment: Self-report & confirmed with radiographs  **Osteoporotic Fractures and Fracture Risk -** Incident hip fracture  Measure/Method of Assessment: Self-report & confirmed with radiographs |

**Abbreviations:** BMC = bone mineral content; BMD = bone mineral density; BMI = body mass index; CT = computed tomography; CVD = cardiovascular disease; d = day; DXA = Dual-energy x-ray absorptiometry; e.g. = exempli gratia; FFQ = food frequency questionnaire; FFQPOP = food frequency questionnaire method for the prevention and management of osteoporosis; HDI = human development index; g = grams; g/100g = grams per 100 grams; g/cm^2^ = grams per centimeter squared; g/d = grams per day; h = hour; IU = international units; IQR = interquartile range; kcal = kilocalories; kg/m^2^ = kilograms per meter squared; METs = metabolic equivalents; min/d = minutes per day; mg = milligrams; mL = milliliter; MPB = milk based protein; MR= meal replacement; NA = not appliable; NR = not reported; PA = physical activity; PAI = physical activity index; PASE = Physical activity scale for the elderly; PMID = PubMed Identification Number; RCT = randomized controlled trail; RoB = Risk of Bias; SD = standard deviation; SE = standard error; SEM = standard error of the mean; μg/L = micrograms per liter; USA = United States of America; wk = week; vBMD = volumetric bone mineral density; y = year
^1^Studies overlap KQs

Supplementary Table 4. Evidence table for Kidney Disease Randomized Controlled Trials (Adults)

| Study | Participants | Interventions/Exposure and Comparator (Content, administrator, and duration) | Intervention (s) (Methods of administration and assessment) | Outcome (Measures and methods of assessment) |
| --- | --- | --- | --- | --- |
| PMID: 20578205 **Flechtner-Mors** **2010^1^ (36)** Location/Country: Germany HDI: Very high Setting: Outpatient clinic Urban/Rural: NR Study design: RCT (parallel) Funding source: Industry, academic **Risk of bias score: High** | Study of: Adults Total sample N: 110  **Intervention:** High Protein  N: 55 % Female: 78.2% Mean Age (SD): 49.3 (12.3) y Race/ Ethnicity: NR Menopausal status: NR Obesity status: Obese Mean BMI (SD): 36.2 (4.4) kg/m^2^ Income level NR Education level: NR Physical activity level: Received instructions to maintain their usual physical activity during the study and not to undertake any new exercise programs, but exercise was not monitored Health status/ Comorbidities: Included: Those that met the criteria for metabolic syndrome Medication use: Exclude: anti-obesity medications Supplement use: NR Pregnant or lactating: Excluded  **Comparator:** Conventional Diet N: 55 % Female: 81.2% Mean Age (SD): 50 (13) y Race/ Ethnicity: NR Menopausal status: NR Obesity status: Obese Mean BMI (SD): 36.3 (5.0) kg/m^2^ Income level NR Education level: NR Physical activity level: Received instructions to maintain their usual physical activity during the study and not to undertake any new exercise programs, but exercise was not monitored Health status/ Comorbidities: Included: Those that met the criteria for metabolic syndrome Medication use: Exclude: anti-obesity medications Supplement use: NR Pregnant or lactating: Excluded | **Intervention:** High Protein  Intended Protein Amount: 1.34 g/kg/d; 30% of energy Carbohydrate: 40% of energy Fat: 30% of energy  Baseline Protein Amount Mean (SD):18.0 (4.9) % of energy; 72.7 (24.3) g/d Carbohydrate Mean (SD): 46.7 (9.4) % of energy; 194 (73) g/d Fat: 35.2 (7.6) % of energy; 64 (25) g/d  Actual Protein Amount at the end of the study Mean (SD): 30.0 (7.0) % of energy; 92.2 (14.8) g/d Carbohydrate Mean (SD): 36.9 (7.9) % of energy; 119 (45) g/d Fat Mean (SD): 29.9 (5.7) % of energy; 42 (13) g/d  Dietary Protein Intake Compliance (%): 56.3%  Protein type/source: Mixed  Energy balance status: Hypocaloric  **Comparator:** Conventional Diet  Intended Protein Amount: 0.8 g/kg/d; 15% energy  Carbohydrate: 30% energy Fat: 55% energy  Baseline Protein Amount Mean (SD): 17.0 (4.7) % of energy; 66.4 (22.7) g/d Carbohydrate Mean (SD): 48.2 (9.4) % of energy; 188 (64) g/d Fat Mean (SD): 34.6 (7.3) % of energy; 60 (26) g/d  Actual Protein Amount at the end of the study Mean (SD): 21.4 (7.4) % of energy; 65.7 (14.7) g/d Carbohydrate Mean (SD): 47.6 (7.5) % of energy; 154 (44) g/d Fat: 29.6 (5.7) % of energy; 44 (16) g/d  Dietary Protein Intake Compliance (%): NR  Protein type/source: Mixed  Energy balance status: Hypocaloric  Study duration: 12 months | **Intervention:** High Protein   **How protein was administered:**  First 3 months: Consumed two protein-enriched meal replacements, one conventional meal, and two snacks as either a protein bar or a low-fat curd with fruit.  After the first 3 months: Consumed one protein-enriched meal replacement, two meals, and two snacks  **Protein Assessment Method:** Subjects kept 3-day food records at baseline, 3 months, 6 months, 9 months, and 12 months. Food quantities were recorded using standard household measures, and a trained assessment dietician reviewed the food records in person. Nutrient calculations were carried out using the PRODI program which is based on German food-composition tables.  **Dietary Protein Intake Compliance:** Food records yielded data that revealed adherence to the dietary recommendations during the study  **Comparator:** Conventional Diet  **How protein was administered:**  First 3 months: Consumed three meals and two snacks with no replacements  After 3 months: Consumed one standard meal replacement, two meals, and two snacks per day  **Protein Assessment Method:** Same as above  **Dietary Protein Intake Compliance:** Same as above | **Kidney Function —** Blood urea nitrogen  Measure/Method of Assessment: Venous blood samples were collected every three months to measure blood urea nitrogen and serum creatinine using standard assays.  **Kidney Function —** Serum creatinine  Measure/Method of Assessment: Venous blood samples were collected every three months to measure blood urea nitrogen and serum creatinine using standard assays. |
| PMID: 18371214 **Frestedt** **2008^1^ (37)** Location/Country: USA HDI: Very high Setting: Community dwelling  Urban/Rural: NR Study design: RCT (parallel) Funding source: Industry **Risk of bias score: High** | Study of: Adults Total sample N: 59  **Intervention**: Prolibra  N: 31 % Female: NR Mean Age (SE): 43.6 (1.1) y Race/ Ethnicity: NR Menopausal status: NR Obesity status: Obese Mean BMI (SE): 35.7 (0.7) kg/m^2^ Income level NR Education level: NR Physical activity level: NR Health status/ Comorbidities: NR Medication use: NR Supplement use: NR Pregnant or lactating: NR  **Comparator**: Placebo N: 28 % Female: NR Mean Age (SE): 42 (1.2) y Race/ Ethnicity: NR Menopausal status: NR Obesity status: Obese Mean BMI (SE): 35.4 (0.7) kg/m^2^ Income level NR Education level: NR Physical activity level: NR Health status/ Comorbidities: NR Medication use: NR Supplement use: NR Pregnant or lactating: NR | **Intervention:** Prolibra  Intended Protein Amount: Supplement 20 g of protein/d (1-10g protein supplement twice daily); 15% of energy Carbohydrate: 55% of energy Fat: 30% of energy  Baseline Protein Amount Mean (SD): 73 (3) g/d; 0.74 g/kg/d Carbohydrate Mean (SD): 222 (11) g/d Fat Mean (SD): 75 (5) g/d  Actual Protein Amount at the end of the study Mean (SD): 0.81 g/kg/d (with supplement); 0.60 g/kg/d and 57 (3) g/d (w/o supplement) Carbohydrate Mean (SD): 178 (8) g/d (w/o supplement) Fat Mean (SD): 49 (3) g/d (w/o supplement)  Dietary Protein Intake Compliance (%): NR  Protein type/source: Animal; whey protein   Energy balance status: Hypocaloric  **Comparator:** Placebo  Intended Protein Amount: 15% of energy Carbohydrate: 55% of energy Fat: 30% of energy  Baseline Protein Amount Mean (SD): 74 (4) g/d; 0.76 g/kg/d Carbohydrate Mean (SD): 211 (10) g/d Fat Mean (SD): 71 (5) g/d  Actual Protein Amount at the end of the study Mean (SD): 0.61 g/kg/d (with supplement); 58 (2) g/d (w/o supplement) Carbohydrate Mean (SD): 182 (9) g/d (w/o supplement) Fat Mean (SD): 47 (3) g/d (w/o supplement)  Dietary Protein Intake Compliance (%): NR  Protein type/source: Mixed  Energy balance status: Hypocaloric  Study duration: 12 weeks | **Intervention:** Prolibra   **How protein was administered:** One Prolibra supplement before breakfast and one before dinner. Each supplement contained 10 g of protein. Subjects were assigned a diet plan with a certain number of servings for various food groups similar to the standard paradigm set by the American Heart Association.  **Protein Assessment Method:** Total protein in Prolibra was measuring using Kjeldahl (AOAC 945.01). Subjects completed diet diaries on at least 5 days each month.  **Dietary Protein Intake Compliance:** Compliance was assessed by supplement count and diet diary review. Participants were also contacted by telephone between visits to review diet and supplement compliance.  **Comparator:** Placebo  **How protein was administered:** Subjects received an iso-caloric beverage containing maltodextrin. Subjects were assigned a diet plan with a certain number of servings for various food groups similar to the standard paradigm set by the American Heart Association.   **Protein Assessment Method:** Same as above  **Dietary Protein Intake Compliance:** Same as above | **Kidney Function —** Blood urea nitrogen Measure/Method of Assessment: Venous blood samples were collected from each subject at weeks 0 and 12 to measure blood urea nitrogen. |
| PMID: 19167797 **Jacobs** **2009 (38)** Location/Country: USA HDI: Very high Setting: Community dwelling Urban/ Rural: NR Study design: RCT (parallel) Funding Source: Government **Risk of bias score: High** | Study of: Adults Total sample N: 378  **Intervention:** DASH Diet N: 127 % Female: 50% Mean Age (SD): 44.2 (10.2) y Race/ Ethnicity: White: 35% African American: 60% Other: 5% Menopausal status: NR Obesity status: NR Mean BMI (SD): 28.5 (4.0) kg/m^2^ Income level: NR Education level: NR Physical activity level: NR Health status/ Comorbidities: Considered healthy but with untreated prehypertension or stage I hypertension (32% hypertensive). Those with diabetes and decreased glomerular filtration rate were excluded Medication use: Use of medications that could affect blood pressure were excluded; no other medication use was reported Supplement use: Use of nutritional supplements that could affect blood pressure were excluded; no other nutritional supplement use was reported Pregnant or lactating: Excluded if current or planned pregnancy prior to end of study or breast-feeding   **Comparator 1:** Fruit/vegetable diet N: 127 % Female: 49% Mean Age (SD): 45.7 (10.6) y Race/ Ethnicity:  White: 38% African American: 57% Other: 5% Menopausal status: NR Obesity status: NR Mean BMI (SD): 28.3 (3.9) kg/m^2^ Income level: NR Education level: NR Physical activity level: NR Health status/ Comorbidities: Considered healthy but with untreated prehypertension or stage I hypertension (32% hypertensive). Those with diabetes and decreased glomerular filtration rate were excluded Medication use: Use of medications that could affect blood pressure were excluded; no other medication use was reported Supplement use: Use of nutritional supplements that could affect blood pressure were excluded; no other nutritional supplement use was reported Pregnant or lactating: Excluded if current or planned pregnancy prior to end of study or breast-feeding  **Comparator 2:** Control diet N: 127 % Female: 45% Mean Age (SD): 49 (11.1) y Race:  White: 38% African American: 57% Other: 5% Menopausal status: NR Obesity status: NR Mean BMI (SD): 27.9 (3.8) kg/m^2^ Income level: NR Education level: NR Physical activity level: NR Health status/ Comorbidities: Considered healthy but with untreated prehypertension or stage I hypertension (32% hypertensive). Those with diabetes and decreased glomerular filtration rate were excluded Medication use: Use of medications that could affect blood pressure were excluded; no other medication use was reported Supplement use: Use of nutritional supplements that could affect blood pressure were excluded; no other nutritional supplement use was reported Pregnant or lactating: Excluded if current or planned pregnancy prior to end of study or breast-feeding | **Intervention:** DASH Diet  Intended Protein Amount: 17.9% of energy Carbohydrate: 56.5% of energy  Fat: 25.6% of energy   Baseline Protein Amount Mean (SD): NR Carbohydrate: NR Fat: NR  Actual Protein Amount at the end of the study: 17.8% of energy  Carbohydrate: 56.5% of energy Fat: 25.6% of energy  Dietary Protein Intake Compliance (%): NR  Protein type/source: Mixed   Energy balance status: Eucaloric  **Comparator 1**: Fruit/vegetable diet  Intended Protein Amount: 15.1% of energy Carbohydrate: 49.2% of energy  Fat: 35.7% of energy  Baseline Protein Amount: NR Carbohydrate: NR Fat: NR  Actual Protein Amount at the end of the study: 15.1% of energy Carbohydrate: 49.2% of energy  Fat: 35.7% of energy   Dietary Protein Intake Compliance (%): NR  Protein type/source: Mixed   Energy balance status: Eucaloric  **Comparator 2:** Control diet  Intended Protein Amount: 13.8% of energy  Carbohydrate: 50.5% of energy Fat: 35.7% of energy   Baseline Protein Amount: NR Carbohydrate: NR Fat: NR  Actual Protein Amount at the end of the study: 14.0% of energy Carbohydrate: 50.5% of energy Fat: 35.7% of energy  Dietary Protein Intake Compliance (%): NR  Protein type/source: mixed   Study Duration/Follow up: 8 weeks | **Intervention**: DASH Diet  **How protein was administered:** NR   **Protein assessment method:** NR  **Dietary Protein Intake Compliance**: NR  **Comparator 1:** Fruit/vegetable diet  **How protein was administered:** NR   **Protein assessment method:** NR  **Dietary Protein Intake Compliance**: NR  **Comparator 2:** Control diet   **How protein was administered:** NR  **Protein assessment method:** NR  **Dietary Protein Intake Compliance**: NR | **Proteinuria —** Urinary albumin excretion  Measure/Method of Assessment: In 24-hour urine samples, albumin concentration was measured by a nephelometer with an enhanced sensitivity and a coefficient of variation in 50 masked duplicate samples; albumin excretion rate was computed as the urinary albumin concentration (mg/L) times the urine volume (L) times 24 hours/collection hours. |
| PMID: 23219108 **Juraschek** **2013 (39)** Location/Country: USA HDI: Very high Setting: Community dwelling  Urban/ Rural: Urban Study Design: RCT (crossover) Funding source: Government **Risk of bias score: High** | Study of: Adults Total sample N: 164  **Overall** N: 164 % Female: 45% Mean Age (SD): 53.5 (10.8) y Race/ Ethnicity:  African American: 55% Non-Hispanic White: 40% Other: 5%  Menopausal status: NR Obesity status: NR Mean BMI (SD): 30.2 (6.1) kg/m^2^ Income level: NR Education level: Education less than or equal to a HS diploma: 20.1% Physical activity level: Participants encouraged to maintain the same activity level Health status/Co-morbidities: Generally healthy Medication use: NR; excluded if taking medications that affect blood pressure or blood lipid levels Supplement use: Excluded if unwilling to stop taking vitamin and mineral supplements Pregnant or lactating: NR | **Intervention:** Protein diet  Intended Protein Amount: 25% of energy Carbohydrate: 48% of energy Fat: 27% of energy  Baseline Protein Amount: NR Carbohydrate: NR Fat: NR  Actual Protein Amount at the end of the study: 25% of energy Carbohydrate: 48% of energy Fat: 27% of energy  Dietary Protein Intake Compliance (%): NR. Overall compliance in the trial was 83.8%.  Protein type/source: Mixed   Energy balance status: Eucaloric  **Comparator 1:** Carbohydrate diet  Intended Protein Amount: 15% of energy Carbohydrate: 58% of energy Fat: 27% of energy  Baseline Protein Amount: NR Carbohydrate: NR Fat: NR  Actual Protein Amount at the end of the study: 15% of energy Carbohydrate: 58% of energy Fat: 27% of energy  Dietary Protein Intake Compliance (%): NR. Overall compliance in the trial was 83.8%.  Protein type/source: Mixed   Energy balance status: Eucaloric  **Comparator 2:** Unsaturated diet  Intended Protein Amount: 15% of energy Carbohydrate: 48% of energy Fat: 37% of energy   Baseline Protein Amount: NR Carbohydrate: NR Fat: NR  Actual Protein Amount at the end of the study: 15% of energy Carbohydrate: 48% of energy Fat: 37% of energy   Dietary Protein Intake Compliance (%): NR. Overall compliance in the trial was 83.8%.  Protein type/source: Mixed   Energy balance status: Eucaloric  Study duration: 6 weeks  **Crossover details:** Number of intakes per participant: 3 Total intakes: 468 Wash out period: 2-4 weeks | **Intervention:** Protein diet  **How protein was administered:** A 7-day menu cycle at 5 caloric levels was developed for each diet. Participants were provided all of their food, which was prepared in research kitchens. For each day of controlled feeding, participants completed a diary in which they indicated whether they ate any nonstudy foods and whether they did not eat all study foods.  **Protein Assessment Method:** For each menu of each diet, sample validation meals were prepared and composited. The nutrient profile of each composite was analyzed for all target nutrients at Covance Laboratories  **Dietary Protein Intake Compliance**: Participants kept a diary in which they listed their consumption of nonprotocol foods; in other words, any foods not given by study staff.  **Comparator 1**: Carbohydrate diet  **How protein was administered:** A 7-day menu cycle at 5 caloric levels was developed for each diet. Participants were provided all of their food, which was prepared in research kitchens. For each day of controlled feeding, participants completed a diary in which they indicated whether they ate any nonstudy foods and whether they did not eat all study foods.  **Protein Assessment Method:** Same as above  **Dietary Protein Intake Compliance**: Participants kept a diary in which they listed their consumption of nonprotocol foods; in other words, any foods not given by study staff.  **Comparator 2**: Unsaturated diet  **How protein was administered:** A 7-day menu cycle at 5 caloric levels was developed for each diet. Participants were provided all of their food, which was prepared in research kitchens. For each day of controlled feeding, participants completed a diary in which they indicated whether they ate any nonstudy foods and whether they did not eat all study foods.  **Protein Assessment Method:** Same as above  **Dietary Protein Intake Compliance**: Participants kept a diary in which they listed their consumption of nonprotocol foods; in other words, any foods not given by study staff. | **Kidney function —** Serum creatinine  Measure/Method of Assessment:  Serum creatinine was measured from serum specimens using standardized laboratory assays.  **Kidney function —** eGFR  Measure/Method of Assessment: eGFR was calculated using the CKD Epidemiology Collaboration (CKD-EPI) cystatin C equation  Creatinine-based eGFR was calculated using the CKD-EPI creatinine equation.  eGFR measurement (with or without race): with race |
| PMID: 25844619 **Kerstetter 2015^1,2^ (9)** Location/Country: USA HDI: Very high Setting: NR Urban/ Rural: NR Study design: RCT (parallel) Funding source: Government, academic **Risk of bias score: High** | Study of: Adults Total sample N: 208  **Intervention:** High Protein N: 106 % Female: 84% Mean Age (SD): 69.9 (6.1) y Race/ Ethnicity: NR Menopausal status: NR Obesity status: NR Mean BMI (SD): 26.1 (3.4) kg/m^2^  Income level: NR Education level: NR Mean physical activity level score (SD): 6.7 (2.1) Health status/ Comorbidities: Healthy older adults Medication use: Excluded if using long-term chemotherapeutic drugs, aromatase inhibitors or tamoxifen, methotrexate, phenytoin, phenobarbital or inhaled corticosteroids (greater than 800 ug/day), actively being treated for leukemia or multiple myeloma, a change in thyroid medications, medications known to affect calcium metabolism or use of proton pump inhibitors twice daily Supplement use: Daily multivitamin mineral supplement (contained 400 IU of vitamin D); Ca carbonate supplement (300 mg tablets) Pregnant or lactating: NR  **Comparator:** Low Protein N: 102 % Female: 87.3% Mean Age (SD): 70.5 (6.4) y Race/ Ethnicity: NR Menopausal status: NR Obesity status: NR Mean BMI (SD): 26.4 (4.0) kg/m^2^ Income level: NR Education level: NR Mean physical activity level score (SD): 6.8 (1.9) Health status/ Comorbidities: Healthy older adults Medication use: Excluded if using long-term chemotherapeutic drugs, aromatase inhibitors or tamoxifen, methotrexate, phenytoin, phenobarbital or inhaled corticosteroids (greater than 800 ug/day), actively being treated for leukemia or multiple myeloma, a change in thyroid medications, medications known to affect calcium metabolism or use of proton pump inhibitors twice daily Supplement use: Daily multivitamin mineral supplement (contained 400 IU of vitamin D); Ca carbonate supplement (300 mg tablets) Pregnant or lactating: NR | **Intervention:** High Protein Intended Protein Amount: 40 g of protein from the supplement; total daily protein goal NR Carbohydrate: Test food protein NR Fat: Test food protein NR  Baseline Protein Amount Least Square Mean (SEM): 73.8 (1.9) g/d Carbohydrate Least Square Mean (SEM): 214.1 (5.2) g/d Fat Least Square Mean (SEM): 59.4 (2.1) g/d  Actual Protein Amount at the end of the study Least Square Mean (SEM): 90.7 (3.3) g/d Carbohydrate Least Square Mean (SEM): 196.9 (6.6) g/d Fat Least Square Mean (SEM): 55.6 (2.0) g/d  Dietary Protein Intake Compliance (%): NR  Protein type/source: Animal; whey supplement   Energy balance status: Eucaloric  **Comparator:** Low Protein  Intended Protein Amount: Test food protein NR Carbohydrate: Test food protein NR Fat: Test food protein NR  Baseline Protein Amount  Least Square Mean (SEM): 72.9 (1.8) g/d; 1.06 (0.03) g/kg/d (total daily) Carbohydrate Least Square Mean (SEM): 206.2 (5.8) g/d (total daily) Fat Least Square Mean (SEM): 61.3 (2.5) g/d (total daily)  Actual Protein Amount at end of the study  Least Square Mean (SEM): 72.7 (2.4) g/d; 1.05 (0.04) g/kg/d (total daily) Carbohydrate Least Square Mean (SEM): 229.0 (9.5) g/d (total daily) Fat Least Square Mean (SEM): 58.8 (2.4) g/d (total daily)  Dietary Protein Intake Compliance (%): NR  Protein type/source: Mixed  Energy balance status: Eucaloric   Study duration: 18 months | **Intervention:** High Protein  **How protein was administered:** Participants received a dietary whey protein supplement (protein group; Provon 290; Glambia Nutritionals) that was closely matched for composition, color, kilocalories, sodium, potassium, phosphorus, fiber, and calcium. **Protein Assessment Method:** Participants completed a 3-day food record prior to baseline, 6 months, and 18 months and were analyzed using the ESHA Food Processor software program (ESHA Research; version 10.1.0).  **Dietary Protein Intake Compliance:** Urinary area was a compliance measure.   **Comparator:** Low Protein  **How protein was administered:**  Participants received a maltodextrin supplement Maltrin M100; Grain Processing Corp) that was closely matched for composition, color, kilocalories, sodium, potassium, phosphorus, fiber, and calcium.  **Protein Assessment Method:** Same as above **Dietary Protein Intake Compliance:** Same as above | **Kidney Function —** eGFR Measure/Method of Assessment:  Serum creatinine measured using an ACE Wasserman autoanalyzer, eGFR calculated from serum creatinine, age, sex, and race using the National Kidney Foundation online calculator.   eGFR measurement (with or without race): with race |
| PMID: 21194471 **Li 2010^1^ (10)** Location/Country: United States HDI: Very high Setting: Community dwelling Urban/ Rural: NR Study design: RCT (parallel) Funding source: Industry **Risk of bias score: High** | Study of: Adults Total sample N: 85  **Intervention:** High Protein N: 44 % Female: 81.8% Mean Age (SD): 48.9 (11.8) y Race/ Ethnicity:  Asian: 9.1% Black: 20.5% Caucasian: 59.1% Hispanic: 9.1% Other: 2.2% Menopausal status: NR Obesity status: Obese Mean BMI (SD): 34.7 (6.8) kg/m^2^ Income level: NR Education level: NR Physical activity level: NR Health status/ Comorbidities: Inclusion: good health history; participants reported to be obese; Exclusion type 2 diabetes or glucose intolerance Medication use: NR Supplement use: NR Pregnant or lactating: NR  **Comparator:** Normal Protein N: 42 % Female: 63.4% Mean Age (SD): 49.7 (9.1) y Race/ Ethnicity: Asian: 2.4% Black: 19.5% Caucasian: 68.3% Hispanic: 4.9% Other: 4.9% Menopausal status: NR Obesity status: Obese Mean BMI (SD): 34.3 (10.3) kg/m^2^  Income level: NR Education level: NR Physical activity level: NR Health status/ Comorbidities: Inclusion: good health history; participants reported to be obese; Exclusion type 2 diabetes or glucose intolerance Medication use: NR Supplement use: NR Pregnant or lactating: NR | **Intervention:** High Protein Intended Protein Amount: 2.2 g per kg of lean body mass; 30% of energy Carbohydrate: 40% of energy Fat: 30% total energy   Baseline Protein Amount Mean (SD): NR Carbohydrate Mean (SD): NR Fat Mean (SD): NR  Actual Protein Amount at the end of the study Mean (SD): NR Carbohydrate Mean (SD): NR Fat Mean (SD): NR  Dietary Protein Intake Compliance (%): NR  Protein type/source: Meal replacement protein: NR, Diet: Mixed  Energy balance status: Eucaloric   **Comparator:** Normal Protein  Intended Protein Amount: 1.1 g per kg of lean body mass (15% total energy) Carbohydrate: 55% total energy Fat: 30% total energy  Baseline Protein Amount Mean (SD): NR  Carbohydrate Mean (SD): NR Fat Mean (SD): NR  Actual Protein Amount at the end of the study Mean (SD): NR Carbohydrate Mean (SD: NR Fat Mean (SD): NR  Dietary Protein Intake Compliance (%): NR  Protein type/source: Mixed  Energy balance status: Eucaloric  Study duration: 12 months | **Intervention:** High Protein **How protein was administered:** Participants received isocaloric MR (Formula 1, Herbalife Intl., Los Angeles) with a protein supplement (Performance Protein Powder, Herbalife Intl., Los Angeles)  **Protein Assessment Method:** Protein was assessed through qualitative food logs and reviewed with dietitians at follow-ups. Protein intake was measured at each follow-up visit; baseline, week 2, and months 1, 2, 3, 6, 9, 12.  **Dietary Protein Intake Compliance:** No special efforts were made to assess compliance.  **Comparator:** Normal Protein  **How protein was administered:** Participants received the isocaloric MR (Formula 1, Herbalife Intl., Los Angeles) with matched carbohydrate placebo containing maltodextrin and flavoring  **Protein Assessment Method:** Same as above **Dietary Protein Intake Compliance:** Same as above | **Kidney Function —** Serum creatinine Measure/Method of Assessment: Standard laboratory blood serum assay methods  **Kidney Function —** Urinary urea nitrogen Measure/Method of Assessment: Twenty-four hour urine samples were collected for urinary urea nitrogen. Urinary urea nitrogen was measured with an enzymatic method.  **Kidney Function —** Creatinine clearance Measure/Method of Assessment: Twenty-four hour urine samples and standard laboratory blood serum assay methods  **Kidney Function —** Serum urea nitrogen Measure/Method of Assessment: Standard laboratory blood serum assay methods |
| PMID: 33871558 **Murphy**  **2021^1^ (40)** Location/Country: Ireland HDI: Very high Setting: Community dwelling Urban/ Rural: Urban  Study design: RCT (parallel) Funding source: Government **Risk of bias score: High** | Study of: Adults Total sample N: 107  **Intervention 1:** Leucine-enriched Protein N: 38 % Female: 52.6% Mean Age (SD): 70 (5) y Race/Ethnicity: 100% White Menopausal status: NR Obesity status: NR Mean BMI (SD): 24.8 (3.4) kg/m^2^ Income level: NR Education level: NR Mean physical activity level (SD): 8354 (4125) steps/day  Health status/ Comorbidities:  Included: Low skeletal muscle mass; generally healthy according to responses to a standard health screening questionnaire Excluded: malignancy in the past 5 years, diabetes, advanced renal disease, neuromuscular disease, total walking incapacity Medication use: Mean (SD) number of mediations: 1 (2); Excluded if taking medications that interfere with the nutrition intervention - corticosteroids for systemic use, hormone replacement therapy, insulin, high-dose anti-inflammatories, simvastatin Supplement use: Excluded if consumed LC n-3 PUFA supplementation and were not willing to cease consumption ≥ 6 weeks prior to and for the duration of the 24-wk study Pregnant or lactating: NR  **Intervention 2**: Leucine-enriched Protein+ PUFAs N: 38 % Female: 55.3% Mean Age (SD): 73 (6) y Race/Ethnicity: 100% White Menopausal status: NR Obesity status: NR Mean BMI (SD): 26.7 (3.2) kg/m^2^  Income level: NR Education level: NR Mean physical activity level (SD): 8257 (3906) steps/d Health status/ Comorbidities:  Included: Low skeletal muscle mass; generally healthy according to responses to a standard health screening questionnaire Excluded: malignancy in the past 5 years, diabetes, advanced renal disease, neuromuscular disease, total walking incapacity Medication use: Mean (SD) number of medications: 2 (2); Excluded if taking medications that interfere with the nutrition intervention - corticosteroids for systemic use, hormone replacement therapy, insulin, high-dose anti-inflammatories, simvastatin Supplement use: Excluded if consumed LC n-3 PUFA supplementation and were not willing to cease consumption ≥ 6 weeks prior to and for the duration of the 24-wk study Pregnant or lactating: NR  **Comparator:** Normal Protein N: 31 % Female: 45.2% Mean Age (SD): 73 (7) y Race/Ethnicity: 100% White Menopausal status: NR Obesity status: NR Mean BMI (SD): 25.4 (2.8) kg/m^2^ Income level: NR Education level: NR Mean physical activity level (SD): 8192 (5142) steps/day Health status/ Comorbidities:  Included: Low skeletal muscle mass; generally healthy according to responses to a standard health screening questionnaire Excluded: malignancy in the past 5 years, diabetes, advanced renal disease, neuromuscular disease, total walking incapacity Medication use: Mean (SD) number of medications: 2 (3); Excluded if taking medications that interfere with the nutrition intervention - corticosteroids for systemic use, hormone replacement therapy, insulin, high-dose anti-inflammatories, simvastatin Supplement use: Excluded if consumed LC n-3 PUFA supplementation and were not willing to cease consumption ≥ 6 weeks prior to and for the duration of the 24-wk study Pregnant or lactating: NR | **Intervention 1:** Leucine-enriched Protein  Intended Protein Amount: 21.2 g in supplemental protein per day; total intake goals NR Carbohydrate: NR Fat: NR  Baseline Protein Amount Mean (SD): 84 (26) g/d; 17.1 (3.9) % of energy Carbohydrate Mean (SD): 226 (78) g/d; 45.0 (9.7) % of energy Fat Mean (SD): 82 (32) g/d; 36.3 (7.7) % of energy  Actual Protein Amount at the end of the study Mean (SD): 100 (23) g/d; 19.6 (3.3) % of energy Carbohydrate Mean (SD): 229 (60) g/d; 44.6 (6.7) % of energy Fat Mean (SD): 80 (24) g/d; 34.8 (6.3) % of energy  Dietary Protein Intake Compliance (%): Median (IQR): 89% (83-94%)  Protein type/source: Whey protein and a peptide carrier enriched with free leucine  Energy balance status: Eucaloric  **Intervention 2:** Leucine-enriched Protein +PUFAS  Intended Protein Amount: 21.2 g in supplemental protein per day; total intake goals NR Carbohydrate: NR Fat: NR  Baseline Protein Amount Mean (SD): 77 (25) g/d; 17.6 (4.5) % of energy Carbohydrate Mean (SD): 200 (66) g/d; 45.6 (8.4) % of energy Fat Mean (SD): 69 (25) g/d; 35.4 (8.8) % of energy  Actual Protein Amount at the end of the study Mean (SD): 92 (25) g/d; 19.9 (4.0) % of energy Carbohydrate Mean (SD): 200 (57) g/d; 43.5 (8.0) % of energy Fat Mean (SD): 76 (28) g/d; 36.2 (7.8) % of energy  Dietary Protein Intake Compliance (%): Median (IQR): 92% (87-97%)  Protein type/source: Animal; whey protein and a peptide carrier enriched with free leucine   Energy balance status: Eucaloric  **Comparator:** Normal Protein  Intended Protein Amount: NR Carbohydrate: NR Fat: NR  Baseline Protein Amount Mean (SD): 79 (34) g/d; 16.7 (5.3) % of energy Carbohydrate Mean (SD): 214 (62) g/d; 45.6 (7.5) % of energy Fat Mean (SD): 80 (34) g/d; 37.4 (9.3) % of energy  Actual Protein Amount at the end of the study Mean (SD): 83 (23) g/d; 15.2 (3.2) % of energy Carbohydrate Mean (SD): 268 (68) g/d; 49.8 (5.6) % of energy Fat Mean (SD): 85 (30) g/d; 34.8 (5.4) % of energy  Dietary Protein Intake Compliance (%): Median (IQR): 93% (87-95%)  Protein type/source: Mixed  Energy balance status: Eucaloric  Study duration: 24 weeks | **Intervention 1:** Leucine-enriched Protein  **How protein was administered:** Two supplements daily equaling 21.2 g protein per day (including 6.2 g leucine); one was consumed before breakfast and one before their second light meal of the day with habitual diet  **Protein Assessment Method:** Dietary intake was assessed via a 24-h recall using the 5-step multiple-pass method at pre-, mid-, and post intervention visits  **Dietary Protein Intake Compliance:** Compliance was derived using the self-report supplement logs  **Intervention 2:** Normal Protein  **How protein was administered:** Two supplements daily equaling 21.2 g protein per day (including 6.2 g leucine and 4 g LC n-3 PUFAs); one was consumed before breakfast and one before their second light meal of the day with habitual diet  **Protein Assessment Method:** Same as above  **Dietary Protein Intake Compliance:** Same as above  **Comparator:** Normal Protein  **How protein was administered:** Isocaloric maltodextrin supplement  **Protein Assessment Method:** Same as above  **Dietary Protein Intake Compliance:** Same as above | **Kidney Function —** eGFR Measure/Method of Assessment: Serum creatinine measured using a chemical autoanalyzer with standardized assays. eGFR was derived using CKD Epidemiology Collaboration (CKD-EPI) cystatin C equation.  eGFR measurement (with or without race): with race  **Kidney Function —** Serum creatinine Measure/Method of Assessment: Measured using a chemical autoanalyzer with standardized assays   **Kidney Function —** Serum cystatin C Measure/Method of Assessment: Measured using a chemical autoanalyzer with standardized assays   **Kidney Function —** Blood urea nitrogen Measure/Method of Assessment: Measured using a chemical autoanalyzer with standardized assays |
| PMID: 34098214 **Peng** **2021^1^ (41)** Location/Country: Taiwan/China HDI: High Setting: Community dwelling  Urban/ Rural: NR Study design: RCT (parallel) Funding source: Academic, industry **Risk of bias score: High** | Study of: Adults Total sample N: 52  **Intervention:** High Protein N: 27 % Female: 48.1% Mean Age (SD): 53.4 (8.1) y Race/ Ethnicity: NR Menopausal status: NR Obesity status: NR Mean BMI (SD): 25.1 (3.9) kg/m^2^ Income level: NR Mean education level (SD): 14.1 (2.9) y Mean physical activity level (SD): 1567.3 (1244.9) kcal/wk Health status/ Comorbidities: Excluded: (1) history of fracture or severe arthritis in recent 6 months, (2) known history of chronic kidney disease stage III and over, i.e. estimated glomerular filtered rate (eGFR) < 60 ml/min/1.73 m^2^, (3) contraindicated for magnetic resonance imaging, (4) using anabolic hormones in the past 3 months, (5) were disability or limited functional ability, (6) having advanced, active or uncontrolled diseases, and (6) dementia, cognitive impairment or other sensory impairment that limited communication and understanding of the study  Medication use: Excluded those using anabolic hormones Supplement use: NR Pregnant or lactating: NR  **Comparator:** Normal Protein N: 25 % Female: 44% Mean Age (SD): 54 (8.6) y Race/Ethnicity: NR Menopausal status: NR Obesity status: NR Mean BMI (SD): 25.6 (3.8) kg/m^2^  Income level: NR Mean education level (SD): 15.5 (2.7) y Mean physical activity level (SD): 1954.0 (1646.4) kcal/wk Health status/ Comorbidities: Excluded: (1) history of fracture or severe arthritis in recent 6 months, (2) known history of chronic kidney disease stage III and over, i.e. estimated glomerular filtered rate (eGFR) < 60 ml/min/1.73 m^2^, (3) contraindicated for magnetic resonance imaging, (4) using anabolic hormones in the past 3 months, (5) were disability or limited functional ability, (6) having advanced, active or uncontrolled diseases, and (6) dementia, cognitive impairment or other sensory impairment that limited communication and understanding of the study  Medication use: Excluded those using anabolic hormones Supplement use: NR Pregnant or lactating: NR | **Intervention:** High Protein  Intended Protein Amount: 25% of energy Carbohydrate: NR Fat: NR  Baseline Protein Amount Mean (SD): NR Carbohydrate Mean (SD): NR Fat Mean (SD): NR  Actual Protein Amount at the end of the study Mean (SD): NR Carbohydrate Mean (SD): NR Fat Mean (SD): NR  Dietary Protein Intake Compliance (%): 91.2%  Protein type/source: Mixed  Energy balance status: Eucaloric  **Comparator:** Normal Protein  Intended Protein Amount: 15% of energy Carbohydrate: NR Fat: NR  Baseline Protein Amount Mean (SD): NR Carbohydrate Mean (SD): NR Fat Mean (SD): NR  Actual Protein Amount at the end of the study Mean (SD): NR Carbohydrate Mean (SD): NR Fat Mean (SD): NR  Dietary Protein Intake Compliance (%): 79.5%  Protein type/source: Mixed  Energy balance status: Eualoric  Study duration: 12 weeks | **Intervention:** High Protein  **How protein was administered:** Received 10 frozen meals per week for 12 weeks containing 25% energy in protein.  **Protein Assessment Method:** NR  **Dietary Protein Intake Compliance:** Insufficient compliance to the study protocol (e.g low meal complete rate and vigorous changes of lifestyle)  **Comparator:** Normal Protein  **How protein was administered:** Received 10 frozen meals per week for 12 weeks containing 15% energy in protein.  **Protein Assessment Method:** Same as above  **Dietary Protein Intake Compliance:** Same as above | **Kidney Function —** eGFR Measure/Method of Assessment: Serum creatinine measured using a chemical autoanalyzer with standardized assays. Measurement methods for eGFR were not reported  eGFR cut off (with or without race): NR  **Kidney Function —** Serum creatinine Measured using a chemical autoanalyzer with standardized assays.  **Kidney Function —** Blood urea nitrogen Measure/Method of Assessment: Measured using a chemical autoanalyzer with standardized assays  Serum creatinine, eGFR, blood urea nitrogen. |
| PMID: 22406907 **Wycherley** **2012^1^ (42)** Location/Country: Australia HDI: Very high Setting: Community dwelling  Urban/Rural: NR Study design: RCT (parallel) Funding source: Industry **Risk of bias score: Moderate** | Study of: Adults Total sample N: 68  **Intervention:** High Protein N: 33 % Female: 0% Mean Age (SD): 51.3 (9.4) y Race/Ethnicity: NR  Menopausal status: NA  Obesity status: 100% overweight or obese Mean BMI (SD): 33.0 (3.9) kg/m^2^ (total study population mean) Income level: NR Education level: NR Physical activity level: NR Health status/ Comorbidities: Excluded: diabetes, uncontrolled hypertension; history of GI, renal, coronary, metabolic, or hepatic disease or malignancy Medication use: Excluded those taking hypoglycemic medication or drugs which affect insulin sensitivity Supplement use: NR Pregnant or lactating: NA  **Comparator:** Low Protein N: 35 % Female: 0% Mean Age (SD): 50.2 (9.3) y Race/Ethnicity: NR Menopausal status: NA Obesity status: 100% overweight or obese Mean BMI (SD): 33.0 (3.9) kg/m^2^ (total study population mean)  Income level: NR Education level: NR Physical activity level: NR Health status/ Comorbidities: Excluded: diabetes, uncontrolled hypertension; history of GI, renal, coronary, metabolic, or hepatic disease or malignancy Medication use: Excluded those taking hypoglycemic medication or drugs which affect insulin sensitivity Supplement use: NR Pregnant or lactating: NA | **Intervention:** High Protein  Intended Protein Amount: 35% of energy; 142 g/d; ~1.30 g/kg/d Carbohydrate: 40% of energy; 135 g/d Fat: 25% of energy (total 53 g/d, saturated 14 g/d)  Baseline Protein Amount Mean (SD): NR Carbohydrate Mean (SD): NR Fat Mean (SD): NR  Actual Protein Amount at the end of the study: Mean (SD): 0-12 weeks: 131.1 (15.4) g/d; 32.5 (3.3) % of energy 12-52 weeks: 132 (13.9) g/d; 30.7 (3.1) % of energy Carbohydrate Mean (SD):  0-12 weeks: 154.4 (31.8) g/d; 37.4 (3.8) % of energy 12-52 weeks: 157.9 (28.1) g/d; 35.9 (3.4) % of energy Fat Mean (SD):  0-12 weeks: 50.6 (6.5) g/d; 27.3 (3.0) % of energy 12-52 weeks: 60.0 (12.6) g/d; 29.8 (3.6) % of energy  Dietary Protein Intake Compliance (%): NR – good compliance rate stated  Protein type/source: Mixed  Energy balance status: Hypocaloric  **Comparator:** Low Protein  Intended Protein Amount: 17% of energy; 88 g/d; ~0.85 g/kg/d Carbohydrate: 58% of energy; 198 g/d Fat: 25% of energy (total 51 g/d, saturated 14 g/d)  Baseline Protein Amount Mean (SD): NR Carbohydrate Mean (SD): NR Fat Mean (SD): NR  Actual Protein Amount at the end of the study Mean (SD):  0-12 weeks: 82.7 (6.7) g/d; 20.5 (1.4) % of energy 12-52 weeks: 83.3 (10.3) g/d; 20.4 (1.0) % of energy Carbohydrate Mean (SD):  0-12 weeks: 208.4 (16.3) g/d; 51.0 (3.6) % of energy 12-52 weeks: 195.2 (23.4) g/d; 47.3 (3.9) % of energy Fat Mean (SD): 0-12 weeks: 46.7 (7.5) g/d; 25.0 (3.3) % of energy 12-52 weeks: 52.2 (8.7) g/d; 27.7 (3.2) % of energy  Dietary Protein Intake Compliance (%): NR – good compliance rate stated  Protein type/source: Mixed  Energy balance status: Hypocaloric  Study duration: 52 weeks | **Intervention:** High Protein  **How protein was administered:** Participants met with dietitian and received detailed dietary prescription, meal planning advice, and recipe information every 2 weeks for the first 12 weeks. They were supplied with a 2-week provision of diet-specific key foods (60% of energy intake) for the first 12 weeks. Participants met with dietician monthly and received detailed dietary prescription, meal planning advice, and recipe information for remainder of study duration.  **Protein Assessment Method:** Participants kept a daily semi-quantitative food record. Dietary intake was assessed using a computerized database (Foodworks Professional Edition, version 4, 1998; Xyris Software, Highgate Hill, Australia) based on the analysis of 3 non-consecutive days (1 weekend day and 2 weekdays) of each 2-week period. The intake was calculated as an average of the 2-week diet record data blocks for 0-12 weeks and 12-52 weeks.   **Dietary Protein Intake Compliance:** Food checklist  **Comparator:** Low Protein  **How protein was administered:** Participants met with dietitian and received detailed dietary prescription, meal planning advice, and recipe information every 2 weeks for the first 12 weeks. They were supplied with a 2-week provision of diet-specific key foods (60% of energy intake) for the first 12 weeks. Participants met with dietician monthly and received detailed dietary prescription, meal planning advice, and recipe information for remainder of study duration.  **Protein Assessment Method:** Same as above  **Dietary Protein Intake Compliance:** Same as above | **Kidney Function —** Creatinine Clearance Measure/Method of Assessment:  Creatinine clearance was calculated as (urine creatinine (mmol-1) x urine volume (ml))/(plasma creatinine (mmol-1) x minutes) and corrected for body surface. |

**Abbreviations:** AER = albumin excretion rate; BMI = Body Mass Index; CKD = chronic kidney disease; d = days; DASH = Dietary Approaches to Stop Hypertension; e.g. = exempli gratia; eGFR = estimated glomerular filtration rate; g = grams; HDI = human development index; IU = international units; kg = kilogram; kg/m^2^ = kilogram per meters squared; kcal = kilocalories; LC n-3 PUFA = n-3 long chain polyunsaturated fatty acids; mg = milligrams; NA = not applicable; NR = not reported; PMID = PubMed Identification Number; PUFA = polyunsaturated fatty acids; RCT = randomized controlled trial; RoB = Risk of Bias; SD = Standard deviation; SE = standard error; SEM = standard error of the mean; USA = United States of America; wk = week; y = year
^1^Studies overlap KQs ^2^Kerstetter, 2015 reported on KQ1, KQ2, and KQ3 outcomes: KQ1 outcomes were assessed as both low (including BMD lumbar, hip and femoral outcomes) and high risk of bias (including all other reported outcomes)

Supplementary Table 5. Evidence table for Kidney Disease Non-Randomized Controlled Trials (Adults)

| Study | Participants | Intervention(s) (Content) | Intervention (s) (Methods of assessment) | Outcome (Measures and methods of assessment) |
| --- | --- | --- | --- | --- |
| PMID: 33203389 **Alvirdizadeh  2020 (43)** Location/Country: Iran HDI: High Setting: Community dwelling Urban/ Rural: Urban Study Design: Prospective cohort study Funding source: Government **Risk of bias score: High** | Study of: Adults Total sample N: 1630   **Tertile 1:** Lower protein intake N: 544 % Female: 55.7% Mean Age (SD): 43.3 (11.3) y  Race/ Ethnicity: NR Menopausal status: NR Obesity status: NR Mean BMI (SD): 27.4 (4.5) kg/m^2^ Income level: NR Education level: NR Physical activity level: 68.2% low physical activity Health status/ Comorbidities: No history of myocardial infarction or stroke or CKD Medication use: NR Supplement use: NR Pregnant or lactating: NR   **Tertile 2:** Moderate protein intake N: 542 % Female: 52.8% Mean Age (SD): 42.8 (10.9) y Race/ Ethnicity: NR Menopausal status: NR Obesity status: NR Mean BMI (SD): 27.6 (4.7) kg/m^2^ Income level NR Education level: NR Physical activity level: 65.2% low physical activity Health status/ Comorbidities: no history of myocardial infarction or stroke or CKD Medication use: NR Supplement use: NR Pregnant or lactating: NR   **Tertile 3:** Higher protein intake N: 544 % Female: 43% Mean Age (SD): 42.4 (11.4) y Race/ Ethnicity: NR Menopausal status: NR Obesity status: NR Mean BMI (SD): 27.6 (4.7) kg/m^2^ Income level NR Education level: NR Physical activity level: 67.1% low physical activity Health status/ Comorbidities: no history of myocardial infarction or stroke or CKD Medication use: NR Supplement use: NR Pregnant or lactating: NR | **Tertile 1:** Lower protein intake   Baseline Protein Amount Mean (SD): 50.36 (9.83) g Carbohydrate Mean (SD): 58.1 (7.6) % of energy Fat Mean (SD): 31. (7.7) % energy   Protein Amount at the end of the study Mean (SD): NR Carbohydrate Mean (SD): NR Fat Mean (SD): NR   **Tertile 2:** Moderate protein intake  Baseline Protein Amount Mean (SD): 74.23 (6.43) g  Carbohydrate Mean (SD): 57.6 (6.8) % of energy Fat Mean (SD): 31.4 (6.9) % of energy  Protein Amount at the end of the study Mean (SD): NR Carbohydrate Mean (SD): NR Fat Mean (SD): NR   **Tertile 3:** Higher protein intake  Baseline Protein Amount Mean (SD): 114.44 (29.42) g Carbohydrate Mean (SD): 57.5 (7.3) % of energy Fat Mean (SD): 30.5 (6.5) % of energy  Protein Amount at the end of the study Mean (SD): NR Carbohydrate Mean (SD): NR Fat Mean (SD): NR  Protein source/type: Mixed  Energy balance: Eucaloric    Study duration: 6 years | **Protein Assessment Method:** The dietary intakes were assessed using a valid and reliable semi-quantitative FFQ by trained dietitians during face-to-face interviews. The United States Department of Agriculture food composition table was applied. Protein intake was only assessed at baseline | **Kidney Function** **—** Incident CKD  Measure/Method of Assessment: CKD derived from MDRD equation with serum creatinine, using eGFR < 60 ml/min/1.73 m^2^   eGFR CKD cut off point: eGFR< 60 ml/min/1.73m^2^  eGFR measurement (with or without race): with race |
| PMID: 29439930 **Cirillo  2018 (44)** Location/Country: Italy HDI: Very high Setting: Community dwelling Urban/ Rural: NR Study Design: Prospective cohort study  Funding source: Government, pharmaceutical **Risk of bias score: High** | Study of: Adults Total sample N: 4307    **Arm 1**: Low urine urea nitrogen (lowest quintile)  N: 861 % Female: 54.7% Mean Age (SD): 52 (20) y Race/ Ethnicity: NR Menopausal status: NR Obesity status: 12.3% Obese Mean BMI (SD): 25.5 (4.2) kg/m^2^ Income level: NR Education level: NR Median physical activity level (95% CI): 0.10 (0.19-0.25) h/d Health status/ Comorbidities: Hypertension: 37.6% Hypercholesterolemia: 29.3% Diabetes: 4.6% Previous cardiovascular disease: 6.4% Medication use: NR Supplement use: NR Pregnant or lactating: NR   **Arm 2:** Non-low urine urea nitrogen (quintile 2-5) N: 3446  % Female: 54.8% Mean Age (SD): 49 (17) y Race/ Ethnicity: NR Menopausal status: NR Obesity status: 22.1% Obese Mean BMI (SD): 27.0 (4.4) kg/m^2^ Income level NR Education level: NR Median physical activity level (95% CI): 0.10 (0.28-0.32) h/d Health status/ Comorbidities: UUN quintile 2: Hypertension: 34.1% Hypercholesterolemia: 31.3% Diabetes: 4.2% Previous cardiovascular disease: 5.5% UUN quintile 3: Hypertension: 31.9% Hypercholesterolemia: 29.9% Diabetes: 3.4% Previous cardiovascular disease: 5.4% UUN quintile 4: Hypertension: 33.7% Hypercholesterolemia: 29.2% Diabetes: 5.9% Previous cardiovascular disease: 4.7% UUN quintile 5: Hypertension: 36.1% Hypercholesterolemia: 32.7% Diabetes: 8.0% Previous cardiovascular disease: 3.7% Medication use: NR Supplement use: NR Pregnant or lactating: NR | **Arm 1:** Low urine urea nitrogen (lowest quintile)   Baseline Protein Amount Mean (SD): 34.0 g/d Carbohydrate Mean (SD): NR Fat Mean (SD): NR   Protein Amount at the end of the study Mean (SD): NR Carbohydrate Mean (SD): NR Fat Mean (SD): NR   **Arm 2:** Non-low urine urea nitrogen (quintile 2-5)  Baseline Protein Amount Mean (SD): UUN quintile 2: 52.7 g/d  UUN quintile 3: 65.0 g/d  UUN quintile 4: 78.6 g/d  UUN quintile 5: 117.0 g/d Carbohydrate Mean (SD): NR Fat Mean (SD): NR   Protein Amount at the end of the study Mean (SD): NR Carbohydrate Mean (SD): NR Fat Mean (SD): NR  Protein type/source: Mixed   Energy balance: Eucaloric    Study duration: 15.9 y | **Protein Assessment Method:** Overnight urinary sodium and potassium were used as indices of their dietary intake. Protein intake was only assessed at baseline. | **Kidney Function** **—** eGFR  Measure/Method of Assessment: eGFR was calculated by the Chronic Kidney Disease Epidemiology Collaboration equation with serum creatinine.   eGFR measurement (with or without race): with race |
| PMID: 30579675 **Farhadnejad 2019 (45)** Location/Country: Iran HDI: High  Setting: Community dwelling Urban/ Rural: Urban Study Design: Prospective cohort study Funding Source: Academic **Risk of bias score: High** | Study of: Adults Total sample N: 1797   **Tertile 1:** Lower Low-Carbohydrate High-Protein Diet Score N: 691 % Female: 41.4% Mean Age (SD): 39.0 (12.5) y Race/ Ethnicity: NR Menopausal status: NR Obesity status: NR Mean BMI (SD): 26.9 (4.8) kg/m^2^ Income level: NR Education level: NR Median physical activity level (IQR): 46.1 (31.7-56.4) MET-h/week Health status/ Comorbidities: Diabetes: 11.5% Hypertension: 19.0% Medication use: NR Supplement use: NR Pregnant or lactating: NR   **Tertile 2:** Moderate Low-Carbohydrate High-Protein Diet Score N: 685 % Female: 56% Mean Age (SD): 37.1 (12.1) y Race/ Ethnicity: NR Menopausal status: NR Obesity status: NR Mean BMI (SD): 26.7 (4.7) kg/m^2^  Income level NR Education level: NR Median physical activity level (IQR): 49.1 (37.3-59.7) MET-h/week Health status/ Comorbidities: Diabetes: 11.0%  Hypertension: 15.9% Medication use: NR Supplement use: NR Pregnant or lactating: NR   **Tertile 3**: Higher Low-Carbohydrate High-Protein Diet Score N: 421 % Female: 63.4% Mean Age (SD): 36.6 (12.3) y Race/ Ethnicity: NR Menopausal status: NR Obesity status: NR Mean BMI (SD): 26.6 (4.8) kg/m^2^ Income level NR Education level: NR Median physical activity level (IQR): 49.2 (49.1-67.4) MET-h/week Health status/ Comorbidities: Diabetes: 12.6%  Hypertension: 18.3% Medication use: NR Supplement use: NR Pregnant or lactating: NR | **Tertile 1:** Lower Low-Carbohydrate High-Protein Diet Score   Baseline Protein Amount: 3 (1-4) score Carbohydrate: NR Fat: NR   Protein Amount at the end of the study Mean (SD): 12.9 (1.7) % of energy Carbohydrate Mean (SD): 64.1 (4.0) % of energy Fat Mean (SD): 25.6 (4.2) % of energy  **Tertile 2**: Moderate Low-Carbohydrate High-Protein Diet Score  Baseline Protein Amount: 7 (7-8) score Carbohydrate: NR Fat: NR   Protein Amount at the end of the study Mean (SD): 13.0 (2.2) % of energy Carbohydrate Mean (SD): 54.5 (5.2) % of energy Fat Mean (SD): 35.0 (6.5) % of energy  **Tertile 3**: Higher Low-Carbohydrate High-Protein Diet Score  Baseline Protein Amount: 10 (9-11) Carbohydrate: NR Fat: NR   Protein Amount at the end of the study Mean (SD): 15.8 (2.1) % of energy Carbohydrate Mean (SD): 51.0 (4.1) % of energy Fat Mean (SD): 35.5 (4.3) % of energy   Protein type/source: Mixed  Energy balance status: Eucaloric   Study duration: 6.1 years | **Protein Assessment Method:** Food intakes of participants over the previous year were assessed using a valid and reliable semiquantitative food- frequency questionnaire, by expert interviewers in the third survey of the TLGS as baseline phase of the present study. This food-frequency questionnaire consisted of 168 food items commonly consumed by Iranians, with standard serving size. Their consumption frequency for each food item during the previous year on a daily, weekly, or monthly basis. The portion sizes of consumed foods were reported in household measures and then converted to grams. The United States Department of Agriculture (USDA) Food Composition Table (FCT) was used. For protein, those with the highest and lowest protein intakes received 4 and 0 points, respectively. Protein intake was only assessed at baseline. | **Kidney Function** **—** Incident CKD  Measure/Method of Assessment: Measured using eGFR < 60 ml/min/1.73 m^2^ from MDRD equation with serum creatinine.  eGFR CKD cut off point: eGFR<60 mL/minute/1.73 m2  eGFR measurement (with or without race): with race |
| PMID: 19443643 **Halbesma  2009 (46)** Location/Country: Netherlands HDI: Very high  Setting: Community dwelling Urban/ Rural: NR Study Design: Prospective cohort study Funding Source: Nonprofit **Risk of bias score: Very high** | Study of: Adults Total sample N: 8461   **Quintile 1:** 0.26-0.99 g of protein/kg/d (combined male and female) N: 1692 % Female: NR Mean Age (SD): 49.0 (13.3) y  Race/ Ethnicity: NR Menopausal status: NR Obesity status: NR Mean BMI (SD): 24.6 (3.8) kg/m^2^ Income level: NR Education level: NR Physical activity level: NR Health status/ Comorbidities: Cardiovascular disease history: 13.8%  Medication use: NR Supplement use: NR Pregnant or lactating: NR   **Quintile 2:** 0.96 to 1.13 g of protein/kg/d (combined male and female)  N: 1692 % Female: NR Mean Age (SD): 50.0 (13.3) y  Race/ Ethnicity: NR Menopausal status: NR Obesity status: NR Mean BMI (SD): 25.3 (3.6) kg/m^2^ Income level: NR Education level: NR Physical activity level: NR Health status/ Comorbidities: Cardiovascular disease history: 12.1% Medication use: NR Supplement use: NR Pregnant or lactating: NR   **Quintile 3:** 1.10 to 1.26 g of protein/kg/d (combined male and female) N: 1693 % Female: NR Mean Age (SD): 49.7 (12.9) y Race/ Ethnicity: NR Menopausal status: NR Obesity status: NR Mean BMI (SD): 25.7 (3.8) kg/m^2^ Income level: NR Education level: NR Physical activity level: NR Health status/ Comorbidities: Cardiovascular disease history: 11.9% Medication use: NR Supplement use: NR Pregnant or lactating: NR   **Quintile 4:** 1.22 to 1.42 g of protein/kg/d (combined male and female) N: 1692 % Female: NR  Mean Age (SD): 50.0 (12.4) y Race/ Ethnicity: NR Menopausal status: NR Obesity status: NR Mean BMI (SD): 26.6 (4.0) kg/m^2^ Income level: NR Education level: NR Physical activity level: NR Health status/ Comorbidities: Cardiovascular disease history: 9.7% Medication use: NR Supplement use: NR Pregnant or lactating: NR   **Quintile 5:** 1.38 to 3.27 g of protein/kg/d (combined male and female) N: 1692 % Female: NR Mean Age (SD): 50.2 (11.4) y Race /Ethnicity: NR Menopausal status: NR Obesity status: NR Mean BMI at baseline: 28.2 (4.2) kg/m^2^ Income level: NR Education level: NR Physical activity level: NR Health status/ Comorbidities: Cardiovascular disease history: 10.1% Medication use: NR Supplement use: NR Pregnant or lactating: NR | **Quintile 1:** 0.26-0.99 g of protein/kg/d (combined male and female)   Baseline Protein Amount Mean (SD): 0.26-0.99 g of protein/kg/d Carbohydrate Mean (SD): NR Fat Mean (SD): NR   Protein Amount at the end of the study Mean (SD): NR Carbohydrate Mean (SD): NR Fat Mean (SD): NR   **Quintile 2:** 0.96 to 1.13 g of protein/kg/d (combined male and female)  Baseline Protein Amount: Mean (SD: 0.96 to 1.13 g of protein/kg/d Carbohydrate Mean (SD): NR Fat Mean (SD): NR   Protein Amount at the end of the study Mean (SD): NR Carbohydrate Mean (SD): NR Fat Mean (SD): NR   **Quintile 3:** 1.10 to 1.26 g of protein/kg/d (combined male and female)  Baseline Protein Amount: mean: 1.10 to 1.26 g of protein/kg/d Carbohydrate Mean (SD): NR Fat Mean (SD): NR   Protein Amount at the end of the study Mean (SD): NR Carbohydrate Mean (SD): NR Fat Mean (SD): NR   **Quintile 4:** 1.22 to 1.42 g of protein/kg/d (combined male and female)   Baseline Protein Amount1.22 to 1.42 g of protein/kg/d Carbohydrate Mean (SD): NR Fat Mean (SD): NR   Protein Amount at the end of the study Mean (SD): NR Carbohydrate Mean (SD): NR Fat Mean (SD): NR   **Quintile 5:** 1.38 to 3.27 g of protein/kg/d (combined male and female)   Baseline Protein Amount: 1.38 to 3.27 g of protein/kg/d (combined male and female) Carbohydrate Mean (SD): NR Fat Mean (SD): NR   Protein Amount at the end of the study Mean (SD): NR Carbohydrate Mean (SD): NR Fat Mean (SD): NR  Protein type/source: Mixed   Energy balance status: Eucaloric   Study duration: 6.4 y | **Protein Assessment Method:** Protein intake was calculated by the method of Maroni and colleagues, in each of the two 24-h urine collections obtained during the first screening round. Protein intake was only assessed at baseline | **Kidney Function** **—** eGFR  Measure/Method of Assessment:  Derived using the MDRD study equation with serum creatinine.   eGFR measurement (with or without race): with race |
| PMID: 28065493 **Haring 2017 (47)** Location/Country: USA HDI: Very high  Setting: Community dwelling Urban/ Rural: Other Study Design: Prospective cohort study Funding Source: Government **Risk of bias score: High** | Study of: Adults Total sample N: 11952   **Quintile 1:** Protein intake 41.1 (7.3) g/d N: 2391 % Female: 64.2% Mean Age (SD): 53.8 (5.8) y Race/ Ethnicity:  Black: 23.3%  White: 76.7%  Menopausal status: NR Obesity status: NR Mean BMI (SD): 26.6 (5.1) kg/m^2^ Income level: NR Education level:  Less than high school: 24.8% High school or equivalent: 45.3%  College or above: 29.9%  Mean physical activity level (SD): Baecke's physical activity index: 2.4 (0.8)  Health status/ Comorbidities: Hypertension: 30.7%  Medication use: Antihypertensive medication: 22.6%,  Lipid lowering medication: 2.1% Supplement use: NR Pregnant or lactating: NR  **Quintile 2:** Protein intake 57.2 (3.6) g/d N: 2390 % Female: 56.9% Mean Age (SD): 53.8 (5.7) y Race/ Ethnicity:  Black: 22.6%  White: 77.4% Menopausal status: NR Obesity status: NR Mean BMI (SD): 26.8 (4.9) kg/m^2^  Income level: NR Education level:  Less than high school: 20.3%  High school or equivalent: 42.9%  College or above: 36.8%  Mean physical activity level (SD): Baecke's physical activity index: 2.5 (0.8)  Health status/ Comorbidities: Hypertension: 30.8%  Medication use: Antihypertensive medication: 22.2% Lipid lowering medication 2.3% Supplement use: NR Pregnant or lactating: NR  **Quintile 3**: Protein intake 69.0 (3.3) g/d N: 2391 % Female: 57.8% Mean Age (SD): 53.8 (5.7) y Race/ Ethnicity:  Black: 22.7% White: 77.3% Menopausal status: NR Obesity status: NR Mean BMI (SD): 27.1 (5.0) kg/m^2^ Income level: NR Education level:  Less than high school: 18.9%  High school or equivalent: 40.2%  College or above: 41.0% Mean physical activity level (SD): Baecke's physical activity index: 2.5 (0.8) Health status/ Comorbidities: Hypertension: 32.6%  Medication use: Antihypertensive medication: 24.5% Lipid lowering medication: 2.4% Supplement use: NR Pregnant or lactating: NR   **Quintile 4**: Protein intake 82.3 (4.5) g/d N: 2390 % Female: 55.4% Mean Age (SD): 53.8 (5.7) y Race/ Ethnicity: Black: 22.1% White: 77.9%  Menopausal status: NR Obesity status: NR Mean BMI (SD): 27.2 (5.0) kg/m^2^ Income level: NR Education level:  Less than high school: 18.9% High school or equivalent: 40.3%  College or above: 40.8%  Mean physical activity level (SD): Baecke's physical activity index: 2.5 (0.8) Health status/ Comorbidities: Hypertension: 31.0%  Medication use: Antihypertensive medication: 23.1% Lipid lowering medication 2.0% Supplement use: NR Pregnant or lactating: NR  **Quintile 5:** Protein intake 109.5 (18.3) g/d N: 2390 % Female: 47.0% Mean Age (SD): 53.7 (5.6) y Race/ Ethnicity:  Black: 23.5% White: 76.5%  Menopausal status: NR Obesity status: NR Mean BMI (SD): 27.6 (5.1) kg/m^2^ Income level: NR Education level:  Less than high school: 19.1%  High school or equivalent: 40.1%  College degree or above: 40.8%  Mean physical activity level (SD): Baecke's physical activity index: 2.5 (0.8) Health status/ Comorbidities: Hypertension: 28.7% Medication use: Antihypertensive medication: 20.3% Lipid lowering medication 2.1% Supplement use: NR Pregnant or lactating: NR | **Quintile 1:** Protein intake 41.1 (7.3) g/d  Baseline Protein Amount Mean (SD): 41.1 (7.3) g/d Carbohydrate Mean (SD): 135.2 (54.3) g/d Fat Mean (SD): 35.8 (11.3) g/d   Protein Amount at the end of the study Mean (SD): NR Carbohydrate Mean (SD): NR Fat Mean (SD): NR   **Quintile 2:** Protein intake 57.2 (3.6) g/d  Baseline Protein Amount Mean (SD): 57.2 (3.6) g/d Carbohydrate Mean (SD): 155.7 (55.1) g/d Fat Mean (SD): 47.7 (13.0) g/d  Protein Amount at the end of the study Mean (SD): NR Carbohydrate Mean (SD): NR Fat Mean (SD): NR  **Quintile 3**: Protein intake 69.0 (3.3) g/d  Baseline Protein Amount Mean (SD): 69.0 (3.3) g/d Carbohydrate Mean (SD): 178.4 (59.6) g/d Fat Mean (SD): 56.6 (14.7) g/d  Protein Amount at the end of the study Mean (SD): NR Carbohydrate Mean (SD): NR Fat Mean (SD): NR  **Quintile 4**: Protein intake 82.3 (4.5) g/d  Baseline Protein Amount Mean (SD): 82.3 (4.5) g/d Carbohydrate Mean (SD): 199.9 (62.2) g/d Fat Mean (SD): 66.2 (16.6) g/d  Protein Amount at the end of the study Mean (SD): NR Carbohydrate Mean (SD): NR Fat Mean (SD): NR  **Quintile 5:** Protein intake 109.5 (18.3) g/d  Baseline Protein Amount Mean (SD): 109.5 (18.3) g/d Carbohydrate Mean (SD): 244.6 (76.0) g/d Fat Mean (SD): 87.7 (25.2) g/d  Protein Amount at the end of the study Mean (SD): NR Carbohydrate Mean (SD): NR Fat Mean (SD): NR  Protein type/source: Mixed  Energy balance status: Eucaloric  Study duration: 25 years | **Protein Assessment Method:** Interviewer-administered, 66-item food frequency questionnaire. The FFQ was administered to all subjects at visit 1 (baseline, 1987–1989) and visit 3 (1993–1995). Protein intake was assessed at baseline and visit 3. | **Kidney Function** **—** Incident CKD   Measure/Method of Assessment: eGFR was calculated using the 2009 Chronic Kidney Disease Epidemiology (CKD-EPI) equation using serum creatinine.   eGFR CKD cut off point: eGFR<60 mL/minute/1.73 m2   eGFR measurement (with or without race): with race |
| PMID: 27935525 **Herber-Gast 2016 (48)** Location/Country: Netherlands HDI: Very high  Setting: Community dwelling Urban/ Rural: NR Study Design: Prospective cohort study Funding Source: Nonprofit **Risk of bias score: High** | Study of: Adults Total sample N: 3763  **Tertile 1:** Lower energy-adjusted total dairy intake N: 1213 % Female: 42.1% Mean Age (SD): 45 (9) y Race/ Ethnicity: NR Menopausal status: NR Obesity status: 10% Obese Mean BMI (SD): 25.5 (3.7) kg/m^2^ Income level: NR Education level:  Low education: 46.0% Physical activity level:  Inactive: 4.6%  Moderately inactive: 20.4% Moderately active: 27.1%  Active: 47.9%  Health status/ Comorbidities: Hypertension: 30.1% Hypercholesterolemia: 23.1% Diabetes: 1.2%  Obesity: 10.0% Medication use: NR Supplement use: NR Pregnant or lactating: pregnant women were censored at the round in which they reported to be pregnant  **Tertile 2:** Moderate energy-adjusted total dairy intake N: 1297 % Female: 48.8% Mean Age (SD): 45 (10) y Race/ Ethnicity: NR Menopausal status: NR Obesity status: 7.2% Obese Mean BMI (SD): 26.7 (4.7) kg/m^2^  Income level NR Education level:  Low education: 39.5%  Physical activity level:  Inactive: 2.7%  Moderately inactive: 17.1% Moderately active: 26.7%  Active: 53.5% Health status/ Comorbidities: Hypertension: 28.6% Hypercholesterolemia: 21.2% Diabetes: 1.0%  Obesity: 7.2% Medication use: NR Supplement use: NR Pregnant or lactating: NR  **Tertile 3:** Higher energy-adjusted total dairy intake N: 1253 % Female: 64.3% Mean Age (SD): 45 (10) y Race/ Ethnicity: NR Menopausal status: NR Obesity status: 9.1% Obese Mean BMI (SD): 25.3 (3.6) kg/m^2^ Income level NR Education level:  Low education: 41.2% Physical activity level:  Inactive: 2.7%  Moderately inactive: 15.9% Moderately active: 26.5% Active: 54.9%  Health status/ Comorbidities: Hypertension: 26.6% Hypercholesterolemia: 21.4% Diabetes: 1.5%  Obesity: 9.1% Medication use: NR Supplement use: NR Pregnant or lactating: NR | **Tertile 1:** Lower energy-adjusted total dairy intake  Baseline Protein Amount Mean (SD): 76.7 (9.6) g/d  Carbohydrate: NR Fat Mean (SD):  Monounsaturated fat: 33.8 (5.4) g/d  Polyunsaturated fat: 17.6 (4.0) g/d   Protein Amount at the end of the study Mean (SD): NR Carbohydrate Mean (SD): NR Fat Mean (SD): NR  **Tertile 2:** Moderate energy-adjusted total dairy intake  Baseline Protein Amount Mean (SD): 81.4 (8.7) g/d  Carbohydrate: NR Fat Mean (SD): Monounsaturated fat: 32.7 (4.7) g/d  Polyunsaturated fat: 16.9 (3.7) g/d  Protein Amount at the end of the study Mean (SD): NR Carbohydrate Mean (SD): NR Fat Mean (SD): NR  **Tertile 3:** Higher energy-adjusted total dairy intake  Baseline Protein Amount Mean (SD): 88.8 (9.6) g/d  Carbohydrate: NR Fat Mean (SD): Monounsaturated fat: 30.8 (4.7) g/d  Polyunsaturated fat: 15.4 (3.4) g/d   Protein Amount at the end of the study Mean (SD): NR Carbohydrate Mean (SD): NR Fat Mean (SD): NR  Protein type/source: Mixed  Energy balance status: Eucaloric  Study duration: 20 y | **Protein Assessment Method:** Diet was assessed at rounds 2–4 with the use of a self- administered semiquantitative food-frequency questionnaire (FFQ) that was developed for the European Prospective Investigation into Cancer and Nutrition study. Participants reported their usual in- takes of 178 food and beverage items over the previous 12 mo. Colored photographs were used to facilitate the estimation of portion sizes, and the seasonal variation in food consumption was taken into account. The consumption of food items (in grams per day) and nutrient intakes were calculated with the use of an extended version of the Dutch Food Composition database of 1996. Protein intake was assessed at study visits 2 (baseline for this analysis), 3, and 4. | **Kidney function** **—** eGFR  Measure/Method of Assessment: Cystatin C was based on a particle-enhanced turbidimetric immunoassay; eGFR was estimated with the use of the Chronic Kidney Disease Epidemiology Collaboration equation with cystatin C.   eGFR measurement (with or without race): with race |
| PMID: 30115136 **Hruby 2018 (49)** Location/Country: USA HDI: Very high  Setting: Community dwelling Urban/ Rural: NR Study Design: Prospective cohort study  Funding Source: Nonprofit, government **Risk of bias score: High** | Study of: Adults Total sample N: 3066  **Quartile 1**: Average 62.7 g of protein/d N: 940 % Female: 40% Mean Age (SE): 55.1 (0.3) y Race/ Ethnicity: NR Menopausal status: NR Obesity status: 23.8% Obese Mean BMI (SE): 26.7 (0.2) kg/m^2^ Income level: NR Education level: NR Mean physical activity level (SE): 35.3 (0.2) MET-h/wk Health status/ Comorbidities: Treatment for hypertension: 17.0% Treatment of CVD: 25.0%  Treatment of diabetes: 2.0% History of cancer: 16.0%  Medication use: NR Supplement use: NR Pregnant or lactating: NR  **Quartile 2:** Average 73.7 g of protein/d N: 742 % Female: 55% Mean Age (SE): 54.4 (0.3) y Race/ Ethnicity: NR Menopausal status: NR Obesity status: 23.8% Obese Mean BMI (SE): 27.1 (0.2) kg/m^2^ Income level: NR Education level: NR Mean physical activity level (SE): 34.6 (0.2) MET-h/wk Health status/ Comorbidities: Treatment for hypertension: 17.0%  Treatment for CVD: 24.0% Treatment for diabetes: 2.0%  History of cancer: 16.0%  Medication use: NR Supplement use: NR Pregnant or lactating: NR  **Quartile 3:** Average 82.4 g of protein/d N: 650 % Female: 59% Mean Age (SE): 54.2 (0.4) y Race/ Ethnicity: NR Menopausal status: NR Obesity status: 23.8% Obese Mean BMI (SE): 27.6 (0.2) kg/m^2^ Income level: NR Education level: NR Mean physical activity level (SE): 34.6 (0.2) MET-h/wk Health status/ Comorbidities: Treatment for hypertension: 17.0%  Treatment for CVD: 25.0% Treatment for diabetes: 4.0% History of cancer: 18.0% Medication use: NR Supplement use: NR Pregnant or lactating: NR  **Quartile 4:** Average 94.5 g of protein/d N: 734 % Female: 62% Mean Age (SE): 53.7 (0.4) y Race/ Ethnicity: NR Menopausal status: NR Obesity status: 23.8% Obese Mean BMI (SE): 28.2 (0.2) kg/m^2^ Income level: NR Education level: NR Mean physical activity level (SE): 34.7 (0.2) MET-h/wk Health status/ Comorbidities: Treatment for hypertension: 19.0% Treatment for CVD: 27.0% Treatment for diabetes: 5.0% History of cancer: 15.0% Medication use: NR Supplement use: NR Pregnant or lactating: NR | **Quartile 1**: Average 62.7 g of protein/d  Baseline Protein Amount Median: 62.7 g/d Carbohydrate Mean: 253.9 g/d Fat Mean: 59.8 g/d  Protein Amount at the end of the study Median: NR Carbohydrate Mean: NR Fat Mean: NR  **Quartile 2:** Average 73.7 g of protein/d  Baseline Protein Amount Median: 73.7 g/d Carbohydrate Mean: 242.8 g/d Fat Mean: 63.1 g/d  Protein Amount at the end of the study Median: NR Carbohydrate: NR Fat: NR  **Quartile 3:** Average 82.4 g of protein/d  Baseline Protein Amount Median: 82.4 g/d Carbohydrate Mean: 232.8 g/d Fat Mean: 63.7 g/d  Protein Amount at the end of the study Median: NR Carbohydrate: NR Fat: NR  **Quartile 4:** Average 94.5 g of protein/d  Baseline Protein Amount Median: 94.5 g/d Carbohydrate Mean: 219.1g/d Fat Mean: 65.0 g/d  Protein Amount at the end of the study Median: NR Carbohydrate: NR Fat: NR  Protein type/source: Mixed  Energy balance status: Eucaloric  Study duration: 20 y | **Protein Assessment Method:** The Harvard semi-quantitative, 126-item FFQ. Protein intake (g/d) was adjusted for total energy intake using the residual method. Quartile categories were created of the average of the reported intake at the beginning and end of each exam interval (e.g. mean of intake reported at exams 5 and 6, for change in outcome between exams 5 and 6). Protein was measured in exams five through nine, and each exam takes four years. | **Kidney function** **—** eGFR  Measure/Method of Assessment: Derived using the CKD Epidemiology Collaboration Equation with serum creatinine.  eGFR measurement (with or without race): without race |
| PMID: 31172186 **Jhee 2020 (50)** Location/Country: South Korea HDI: Very high  Setting: Community dwelling Urban/ Rural: Urban Study Design: Prospective cohort study Funding Source: Government **Risk of bias score: Very high** | Study of: Adults Total sample N: 9226  **Quartile 1:** Protein intake 0.6 g/kg/d  N: 2305 % Female: 63.5% Mean Age (SD): 54.7 (8.9) y Race/ Ethnicity: NR Menopausal status: NR Obesity status: NR Mean BMI (SD): 24.3 (3.3) kg/m^2^ Income level:  Low: 52.2% Intermediate: 37.9% High: 9.9%  Education level:  Low: 50% Intermediate: 43.4% High: 6.6%  Physical activity level: 28.1% Health status/ Comorbidities: Hypertension: 17.4% Diabetes: 5.6%  Dyslipidemia: 2.2% MI: 0.9%  CHF: 0.2% CAD: 0.9% Medication use: NR Supplement use: NR Pregnant or lactating: NR  **Quartile 2:** Protein intake 0.9 g/kg/d N: 2307 % Female: 52.4% Mean Age (SD): 52.2 (8.9) y Race/ Ethnicity: NR Menopausal status: NR Obesity status: NR Mean BMI (SD): 24.6 (3.1) kg/m^2^ Income level:  Low: 33.1% Intermediate: 49.2% High: 17.7%  Education level:  Low: 34.9% Intermediate: 52.5%  High:12.6%  Physical activity level: 38.4% Health status/ Comorbidities: Hypertension: 13.8% Diabetes: 6.5% Dyslipidemia: 2.5% MI: 0.7% CHF: 0.2% CAD: 0.8% Medication use: NR Supplement use: NR Pregnant or lactating: NR  **Quartile 3:** Protein intake 1.1 g/kg/d  N: 2307 % Female: 48% Mean Age (SD): 50.8 (8.5) y Race/ Ethnicity: NR Menopausal status: NR Obesity status: NR Mean BMI (SD): 24.6 (3.0) kg/m^2^ Income level:  Low: 2.2% Intermediate: 50.6%  High: 22.1%  Education level:  Low: 24.3% Intermediate: 58.2%  High: 17.5%  Physical activity level: 45.0% Health status/ Comorbidities: Hypertension: 14.0% Diabetes: 6.7% Dyslipidemia: 2.5% MI: 0.7% CHF: 0.1% CAD: 0.6% Medication use: NR Supplement use: NR Pregnant or lactating: NR   **Quartile 4:** Protein intake 1.7 g/kg/d N: 2307 % Female: 62% Mean Age (SD): 50.2 (8.2) y Race/ Ethnicity: NR Menopausal status: NR Obesity status: NR Mean BMI (SD): 24.7 (3.1) kg/m^2^ Income level:  Low: 25.1% Intermediate: 49.8%  High: 25.2%  Education level:  Low: 22.2% Intermediate: 58.8%  High: 19.0% Physical activity level: 47.5% Health status/ Comorbidities: Hypertension: 12.3% Diabetes: 7.0% Dyslipidemia: 2.4% MI: 0.9% CHF: 0.3%  CAD: 0.6% Medication use: NR Supplement use: NR Pregnant or lactating: NR | **Quartile 1:** Protein intake 0.6 g/kg/d   Baseline Protein Amount Mean (SD): 0.6 (0.1) g/kg/day Carbohydrate Mean (SD): 4.3 (1.2) g/kg/day Fat Mean (SD): 0.2 (0.1) g/kg/day  Protein Amount at the end of the study Mean (SD): NR Carbohydrate Mean (SD): NR Fat Mean (SD): NR  **Quartile 2:** Protein intake 0.9 g/kg/d  Baseline Protein Amount Mean (SD): 0.9 (0.1) g/kg/day Carbohydrate Mean (SD): 5.1 (1.3) g/kg/day Fat Mean (SD): 0.4 (0.1) g/kg/day  Protein Amount at the end of the study Mean (SD): NR Carbohydrate Mean (SD): NR Fat Mean (SD): NR  **Quartile 3:** Protein intake 1.1 g/kg/d  Baseline Protein Amount Mean (SD): 1.1 (0.2) g/kg/day Carbohydrate Mean (SD): 5.7 (1.5) g/kg/day Fat Mean (SD): 0.5 (0.1) g/kg/day  Protein Amount at the end of the study Mean (SD): NR Carbohydrate Mean (SD): NR Fat Mean (SD): NR  **Quartile 4:** Protein intake 1.7 g/kg/d  Baseline Protein Amount Mean (SD): 1.7 (0.6) g/kg/day Carbohydrate Mean (SD): 7.3 (2.8) g/kg/day Fat Mean (SD): 0.9 (0.5) g/kg/day  Protein Amount at the end of the study  Mean (SD): NR Carbohydrate Mean (SD): NR Fat Mean (SD): NR  Protein type/source: Mixed  Energy balance status: Eucaloric  Study duration: 13 y | **Protein Assessment Method:** Trained dietitians with a semiquantitative food frequency questionnaire. Based on the FFQ, the subjects were categorized into four groups according to quartiles of daily amount of protein intake at baseline. Protein intake was only assessed at baseline. | **Hyperfiltration**  Measure/Method of Assessment: Hyperfiltration was defined as a logarithm transformed eGFR larger than the 95th percentile in the distribution of residuals from the multivariable linear regression after the adjustment for logarithm-transformed age, sex, history of hypertension and/or diabetes, height and weight   eGFR measurement (with or without race): with race   **Kidney Function** **—** eGFR  Measure/Method of Assessment: Derived using the CKD Epidemiology Collaboration Equation with serum creatinine.  eGFR measurement (with or without race): with race |
| PMID: 12639078 **Knight 2003 (51)** Location/Country: USA HDI: Very high  Setting: Community dwelling Urban/ Rural: NR Study Design: Prospective cohort study  Funding Source: Government **Risk of bias score: Very high** | Study of: Adults Total sample N: 1624  **Arm 1:** Participants with Normal Renal Function (GFR >80 mL/min per 1.73 m^2^)  N: 1135 % Female: 100% Mean Age (SD): 54.8 (6.6) y Race/ Ethnicity:  White: 98%  African American: 1% Menopausal status: NR Obesity status: NR Mean BMI (SD): NR Income level: NR Education level: NR Physical activity level: NR Health status/ Comorbidities: Hypercholesterolemia: 50% Diabetes: 5% Hypertension: 36% Medication use: NR Supplement use: NR Pregnant or lactating: NR  **Arm 2:** Participants with Mild Renal Insufficiency (estimated GFR>55 mL/min per 1.73 m^2^ but<80 mL/min per 1.73 m^2^) N: 489 % Female: 100% Mean Age (SD): 56.8 (6.5) y Race/ Ethnicity:  White: 98% African American: 1% Menopausal status: NR Obesity status: NR Mean BMI (SD): NR Income level: NR Education level: NR Physical activity level: NR Health status/ Comorbidities: Hypercholesterolemia: 62% Diabetes: 3% Hypertension: 42% Medication use: NR Supplement use: NR Pregnant or lactating: NR | **Arm 1:** Participants with Normal Renal Function (GFR >80 mL/min per 1.73 m^2^)   Baseline Protein Amount Mean (SD): 76.7 (13.6) g/d Carbohydrate Mean (SD): NR Fat Mean (SD): 29.9 (9.1) g/d  Protein Amount at the end of the study Mean (SD): NR Carbohydrate Mean (SD): NR Fat Mean (SD): NR  **Arm 2:** Participants with Mild Renal Insufficiency (estimated GFR>55 mL/min per 1.73 m^2^ but<80 mL/min per 1.73 m^2^)   Baseline Protein Amount Mean (SD): 76.2 (13.3) g/d Carbohydrate Mean (SD): NR Fat Mean (SD): 30.0 (8.1) g/day  Protein Amount at the end of the study Mean (SD): NR Carbohydrate Mean (SD): NR Fat Mean (SD): NR  Protein type/source: Mixed  Energy balance status: Eucaloric  Study duration: 11 y | **Protein Assessment Method:** Reported frequency of consumption of each specified unit of food or beverage by using published data on the nutrient content of the specified portions. Protein were measured twice in 1990 and 1994. | **Kidney Function** **—** eGFR  Measure/Method of Assessment: Derived using the CKD Epidemiology Collaboration Equation with serum creatinine.   eGFR measurement (with or without race): with race |
| PMID: 37211392 **Kubo 2023 (52)** Location/Country: Japan HDI: Very high  Setting: Community dwelling Urban/ Rural: Other Study Design: Prospective cohort study Funding Source: Other **Risk of bias score: High** | Study of: Adults Total sample N: 3277  **Quartile 1:** Protein intake 12% of energy N: 819 % Female: 66.4% Mean Age (SD): 58.8 (7.4) y Race/ Ethnicity: NR Menopausal status: NR Obesity status: NR Mean BMI (SD): 23.2 (3.1) kg/m^2^ Income level: NR Education level: NR Physical activity level: NR Health status/ Comorbidities: Diabetes mellitus: 4.4%  Medication use: Cholesterol-lowering medication: 8.3% Antihypertensive medication: 17.5% Supplement use: NR Pregnant or lactating: NR  **Quartile 2:** Protein intake 14.2% of energy N: 819 % Female: 63.9% Mean Age (SD): 59.0 (8.5) y Race/ Ethnicity: NR Menopausal status: NR Obesity status: NR Mean BMI (SD): 23.4 (3.1) kg/m^2^ Income level: NR Education level: NR Physical activity level: NR Health status/ Comorbidities: Diabetes mellitus: 6.7%  Medication use:  Cholesterol-lowering medication: 9.3%  Antihypertensive medication: 18.8%  Supplement use: NR Pregnant or lactating: NR  **Quartile 3:** Protein intake 15.9% of energy  N: 820 % Female: 65% Mean Age (SD): 58.6 (8.5) year Race/ Ethnicity: NR Menopausal status: NR Obesity status: NR Mean BMI (SD): 23.4 (3.1) kg/m^2^ Income level: NR Education level: NR Physical activity level: NR Health status/ Comorbidities: Diabetes mellitus: 5.2%  Medication use:  Cholesterol-lowering medication: 10.6%  Antihypertensive medication: 17.6%  Supplement use: NR Pregnant or lactating: NR  **Quartile 4**: Protein intake 18.9% of energy N: 819 % Female: 64.4% Mean Age (SD): 58.9 (8.5) y Race/ Ethnicity: NR Menopausal status: NR Obesity status: NR Mean BMI (SD): 23.4 (3.1) kg/m^2^ Income level: NR Education level: NR Physical activity level: NR Health status/ Comorbidities: Diabetes mellitus: 6.2%  Medication use: Cholesterol-lowering medication: 9.7% Antihypertensive medication: 17.3%  Supplement use: NR Pregnant or lactating: NR | **Quartile 1:** Protein intake 12% of energy  Baseline Protein Amount Mean (SD): 12% (1.2) % of energy Carbohydrate Mean (SD): 58.6 (8.0) % of energy Fat Mean (SD): 21.3 (4.8) % of energy  Protein Amount at the end of the study Mean (SD): NR Carbohydrate Mean (SD): NR Fat Mean (SD): NR  **Quartile 2:** Protein intake 14.2% of energy  Baseline Protein Amount Mean (SD): 14.2 (0.5) % of energy Carbohydrate Mean (SD): 56.1 (5.7) % of energy Fat Mean (SD): 25.3 (4.1) % of energy  Protein Amount at the end of the study Mean (SD): NR Carbohydrate Mean (SD): NR Fat Mean (SD): NR  **Quartile 3:** Protein intake 15.9% of energy  Baseline Protein Amount Mean (SD): 15.9 (0.6) % of energy Carbohydrate Mean (SD): 53.6 (5.0) % of energy Fat Mean (SD): 27.4 (4.1) % of energy  Protein Amount at the end of the study Mean (SD): NR Carbohydrate Mean (SD): NR Fat Mean (SD): NR  **Quartile 4**: Protein intake 18.9% of energy  Baseline Protein Amount Mean (SD): 18.9 (2.0) % of energy Carbohydrate Mean (SD): 49.1 (5.4) % of energy Fat Mean (SD): 29.4 (4.3) % of energy  Protein Amount at the end of the study Mean (SD): NR Carbohydrate Mean (SD): NR Fat Mean (SD): NR  Protein type/source: Mixed  Energy balance status: Eucaloric  Study duration: 12 y | **Protein Assessment Method:** Brief-type self-administered diet history questionnaire (BDHQ) at the baseline survey between 2002 and 2006. Protein was measured only at baseline visit. | **Kidney Function** **—** Incident CKD  Measure/Method of Assessment: eGFR was calculated using the Chronic Kidney Disease Epidemiology Collaboration (CKD-EPI) equation with serum creatinine and the Japanese coefficient; Incidence of CKD was defined as appearance of reduced eGFR (<60 mL/min/1.73m2) during follow up.  eGFR CKD cut off point: eGFR< 60 ml/min/ 1.73m2   eGFR measurement (with or without race): with race |
| PMID: 35947164 **Kwon 2022 (53)** Location/Country: Korea HDI: Very high  Setting: Community dwelling Urban/ Rural: Other Study Design: Prospective cohort study Funding Source: Government **Risk of bias score: High** | Study of: Adults Total sample N: 7339   **Tertile 1**: Protein intake <0.8 g/kg/d  N: 2140 % Female: 52.9% Mean Age (SD): 53.1 (8.8) y Race/ Ethnicity: NR Menopausal status: NR Obesity status: 56.1% obese Mean BMI (SD): 25.5 (3.2) kg/m^2^ Income level: NR Education level: NR Physical activity level: <7.5 METs-h/wk: 8.1% 7.5–30 METs-h/wk: 56.6% >30 METs-h/wk: 35.2% Health status/ Comorbidities: Hypertension: 44.8% Diabetes mellitus: 12.7% Medication use: NR Mean supplement use (SD): Calcium intake: 279.7 (119.2) mg/day  Phosphorus intake: 670.7 (156.5) mg/day Pregnant or lactating: NR  **Tertile 2:** Protein intake 0.8–1.3 g/kg/d  N: 3531 % Female: 50.6% Mean Age (SD): 51.4 (8.5) y Race/ Ethnicity: NR Menopausal status: NR Obesity status: 41.6% obese Mean BMI (SD): 24.5 (2.9) kg/m^2^ Income level: NR Education level: NR Physical activity level: <7.5 METs-h/wk: 6.9%  7.5–30 METs-h/wk: 64.1% >30 METs-h/wk: 29.1% Health status/ Comorbidities: Hypertension: 34.8% Diabetes mellitus: 12.5% Medication use: NR Mean supplement use (SD):  Calcium intake: 463.2 (167.6) mg/day;  Phosphorus intake: 1003.1 (192.6) mg/day Pregnant or lactating: NR  **Tertile 3:** Protein intake >1.3 g/kg/d  N: 1668 % Female: 57.1% Mean Age (SD): 51.1 (8.6) y Race/ Ethnicity: NR Menopausal status: NR Obesity status: 30% obese Mean BMI (SD): 23.6 (3.0) kg/m^2^ Income level: NR Education level: NR Physical activity level:  <7.5 METs-h/wk: 7.0%  7.5–30 METs-h/wk: 59.3%, >30 METs-h/wk: 33.7% Health status/ Comorbidities: Hypertension: 32.0% Diabetes mellitus: 10.4% Medication use: NR Mean supplement use (SD):  Calcium intake: 748.4 (294.9) mg/day Phosphorus intake: 1508.6 (360.4) mg/day Pregnant or lactating: NR | **Tertile 1:** Protein intake <0.8 g/kg/d  Baseline Protein Amount Mean (SD): 11.9 (2.1) % of energy Carbohydrate Mean (SD): 72.5 (6.2) % of energy Fat Mean (SD): 13.9 (5.1) % of energy  Protein Amount at the end of the study Mean (SD): NR Carbohydrate Mean (SD): NR Fat Mean (SD): NR  **Tertile 2**: Protein intake 0.8–1.3 g/kg/d  Baseline Protein Amount Mean (SD): 13.5 (1.9) % of energy Carbohydrate Mean (SD): 67.2 (6.2) % of energy Fat Mean (SD): 18.1 (5.1) % of energy  Protein Amount at the end of the study Mean (SD): NR Carbohydrate Mean (SD): NR Fat Mean (SD): NR  **Tertile 3:** Protein intake >1.3 g/kg/d  Baseline Protein Amount Mean (SD): 14.9 (2.2) % of energy Carbohydrate Mean (SD): 62.5 (7.5) % of energy Fat Mean (SD): 21.8 (6.0) % of energy  Protein Amount at the end of the study Mean (SD): NR Carbohydrate Mean (SD): NR Fat Mean (SD): NR  Protein type/source: Mixed  Energy balance status: Eucaloric  Study duration: 16 y | **Protein Assessment Method:** The study used a semi-quantitative food frequency questionnaire (FFQ) with 103 items to assess dietary intake through in-person interviews conducted by well-trained dietitians every two years. Protein measurement from only baseline visit were used in this study. | **Kidney Function** **—** Incident CKD  Measure/Method of Assessment: Incident CKD was defined as eGFR < 60 ml/min/ 1.73m^2^ and eGFR was calculated using the Chronic Kidney Disease Epidemiology Collaboration (CKD-EPI) equation with serum creatinine.  eGFR CKD cut off point: eGFR < 60 ml/min/ 1.73m2  eGFR measurement (with or without race): with race   **Proteinuria**  Measure/Method of Assessment: presence of proteinuria determined with a dipstick urine test result of protein level equal to trace or more. |
| PMID: 27416946 **Lew 2017 (54)** Location/Country: Singapore HDI: Very high Setting: Community dwelling Urban/ Rural: Urban Study Design: Prospective cohort study Funding Source: Government **Risk of bias score: High** | Study of: Adults Total sample N: 60,198  **Quartile 1**: 12.5 g/d median red meat intake N: 15,143 % Female: 50% Mean Age (SD): 56.5 (7.8) y Race/ Ethnicity: NR Menopausal status: NR Obesity status: NR Mean BMI (SD): 23.0 (3.3) kg/m^2^ Income level: NR Education level: Secondary school or higher: 31% Physical activity level: 39% with weekly moderate activity, vigorous activity or strenuous sports lasting at least 30 minutes. Health status/ Comorbidities: Hypertension: 24% Diabetes: 7% 649 Coronary heart disease: 4%, Stroke: 2% Medication use: NR Supplement use: NR Pregnant or lactating: NR  **Quartile 2:** 24.2 g/d median red meat intake N: 15,199 % Female: 46% Mean Age (SD): 56.9 (8.1) y Race/ Ethnicity: NR Menopausal status: NR Obesity status: NR Mean BMI (SD): 23.1 (3.2) kg/m^2^ Income level: NR Education level: Secondary school or higher: 27% Physical activity level: 33% with weekly moderate activity, vigorous activity or strenuous sports lasting at least 30 minutes. Health status/ Comorbidities: Hypertension: 25% Diabetes: 9%  Coronary heart disease: 4%, Stroke: 2% Medication use: NR Supplement use: NR Pregnant or lactating: NR  **Quartile 3:** 33.4 g/d median red meat intake N: 14,909 % Female: 56% Mean Age (SD): 56.5 (8.1) y Race/ Ethnicity: NR Menopausal status: NR Obesity status: NR Mean BMI (SD): 23.2 (3.2) kg/m^2^ Income level: NR Education level: Secondary school or higher: 26% Physical activity level: 30% with weekly moderate activity, vigorous activity or strenuous sports lasting at least 30 minutes. Health status/ Comorbidities: Hypertension: 23% Diabetes: 10% Coronary heart disease: 4%, Stroke: 1% Medication use: NR Supplement use: NR Pregnant or lactating: NR  **Quartile 4**: 48.8 g/d median red meat intake N: 14,947 % Female: 55% Mean Age (SD): 55.7 (7.9) y Race/ Ethnicity: NR Menopausal status: NR Obesity status: NR Mean BMI (SD): 23.2 (3.3) kg/m^2^ Income level: NR Education level: Secondary school or higher: 30%, Physical activity level: 31% with weekly moderate activity, vigorous activity or strenuous sports lasting at least 30 minutes Health status/ Comorbidities: Hypertension: 22% Diabetes: 9% Coronary heart disease: 4% Stroke: 1% Medication use: NR Supplement use: NR Pregnant or lactating: NR | **Quartile 1**: 12.5 g/d median red meat intake  Baseline Protein Amount Mean (SD): 53.1 (10.3) g/d  Carbohydrate Mean (SD): NR Fat Mean (SD): NR  Protein Amount at the end of the study Mean (SD): NR Carbohydrate Mean (SD): NR Fat Mean (SD): NR  **Quartile 2:** 24.2 g/d median red meat intake  Baseline Protein Amount Mean (SD): 57.6 (7.9) g/d  Carbohydrate Mean (SD): NR Fat Mean (SD): NR  Protein Amount at the end of the study Mean (SD): NR Carbohydrate Mean (SD): NR Fat Mean (SD): NR  **Quartile 3:** 33.4 g/d median red meat intake  Baseline Protein Amount Mean (SD): 60.5 (7.6) g/d  Carbohydrate Mean (SD): NR Fat Mean (SD): NR  Protein Amount at the end of the study Mean (SD): NR Carbohydrate Mean (SD): NR Fat Mean (SD): NR  **Quartile 4**: 48.8 g/d median red meat intake  Baseline Protein Amount Mean (SD): 65.3 (9.0) g/d  Carbohydrate Mean (SD): NR Fat Mean (SD): NR  Protein Amount at the end of the study Mean (SD): NR Carbohydrate Mean (SD): NR Fat Mean (SD): NR  Protein type/source: Mixed  Energy balance status: Eucaloric  Study duration: 5 y | **Protein Assessment Method**: Semiquantitative food frequency questionnaire, the dietary nutrients of the food items were derived from the Singapore Food Composition Database, which was developed together with this cohort study and is a food- nutrient database that lists the levels of 96 nutritive/non-nutritive compounds per 100 g of cooked food and beverages in the Singaporean Chinese diet. Protein was measured at baseline. | **Kidney Function —** Incident ESRD   Measure/Method of Assessment: ESRD was defined using the following criteria: 1) serum creatinine level >880 mmol/L (10 mg/dl), 2) eGFR <15 ml/min per 1.73 m2, 3) hemodialysis or peritoneal dialysis, or 4) kidney transplant. Criteria 1–3 had to be persistent for 3 months to qualify as ESRD  eGFR ESRD cut off point: eGFR<15 ml/min per 1.73 m^2^  eGFR measurement (with or without race): with race |
| PMID: 27562875 **Malhotra 2016 (55)** Location/Country: USA HDI: Very high Setting: Community dwelling Urban/ Rural: NR Study Design: Prospective cohort study Funding Source: Government **Risk of bias score: Very high** | Study of: Adults Total sample N: 4255  **Arm 1**: Incident end-stage renal disease cases N: 1057 % Female: 54.4% Mean Age (SD): 54.5 (9.1) y Race/ Ethnicity: 86.7% Black Menopausal status: NR Obesity status: NR Mean BMI (SD): 31.8 (8.2) kg/m^2^ Income level: <$15,000 annually: 68.3% Education level:  <HS: 36.8% Physical activity level: NR Health status/ Comorbidities: Hypertension: 84.2% Diabetes: 63.7% Medication use: NR Supplement use: NR Pregnant or lactating: NR  **Arm 2:** Control group N: 3198 % Female: 55.2% Mean Age (SD): 54.6 (8.8) y Race/ Ethnicity: 86.8% Black Menopausal status: NR Obesity status: NR Mean BMI (SD): 30.3 (7.2) kg/m^2^ Income level: <$15,000 annually: 59.6% Education level: <HS: 34.2% Physical activity level: NR Health status/ Comorbidities: Hypertension: 61.4% Diabetes: 23.0% Medication use: NR Supplement use: NR Pregnant or lactating: NR | **Arm 1**: Incident end-stage renal disease cases   Baseline Protein Amount Mean (SD): 15.7 (3.3) % of energy Carbohydrate Mean (SD): 49.7 (9.1) % of energy  Fat Mean (SD): 31.1 (6.4) % of energy  Protein Amount at the end of the study Mean (SD): NR Carbohydrate: NR Fat: NR  **Arm 2:** Control group  Baseline Protein Amount Mean (SD): 15.1 (3.1) % of energy Carbohydrate Mean (SD): 49.6 (9.1) % of energy Fat Mean (SD): 30.9 (6.8) % of energy  Protein Amount at the end of the study Mean (SD): NR Carbohydrate: NR Fat: NR  Protein type/source: Mixed  Energy balance status: Eucaloric  Study duration: 7 y | **Protein Assessment Method:** Dietary intake was assessed using a validated food frequency questionnaire at baseline which showed strong agreement for protein intake estimated from the FFQ and 24 hour dietary recalls. Protein was measured at baseline only. | **Kidney Function —** Incident ESRD  Measure/Method of Assessment: Incidence of ESRD derived from the US Renal Data System (USRDS), a national disease registry.  eGFR measurement (with or without race): not reported |
| PMID: 29452887 **Malhotra 2018 (56)** Location/Country: USA HDI: Very high Setting: Community dwelling Urban/ Rural: NR Study Design: Prospective cohort study Funding Source: Government, academic **Risk of bias score: Very high** | Study of: Adults Total sample N: 3165  **Quintile 1:** 10.4 % of Energy From Protein Intake at Baseline N: 633 % Female: 69% Mean Age (SD): 55 (12) y Race/ Ethnicity: NR Menopausal status: NR Obesity status: NR Mean BMI (SD): 30.5 (7.0) kg/m^2^ Income level: NR Education level: NR Physical activity level: NR Health status/ Comorbidities: Hypertension: 54% Diabetes: 11% Medication use: NR Supplement use: NR Pregnant or lactating: NR  **Quintile 2:** 12.8% of Energy From Protein Intake at Baseline N: 633 % Female: 65% Mean Age (SD): 55 (12) y Race/ Ethnicity: NR Menopausal status: NR Obesity status: NR Mean BMI (SD): 31.1 (6.9) kg/m^2^ Income level: NR Education level: NR Physical activity level: NR Health status/ Comorbidities: Hypertension: 56% Diabetes: 13% Medication use: NR Supplement use: NR Pregnant or lactating: NR  **Quintile 3**: 14.3% of Energy From Protein Intake at Baseline N: 633 % Female: 63% Mean Age (SD): 54 (12) y Race/ Ethnicity: NR Menopausal status: NR Obesity status: NR Mean BMI (SD): 32.1 (7.2) kg/m^2^ Income level: NR Education level: NR Physical activity level: NR Health status/ Comorbidities: Hypertension: 57% Diabetes: 18% Medication use: NR Supplement use: NR Pregnant or lactating: NR  **Quintile 4:** 16.0% of Energy From Protein Intake at Baseline N: 633 % Female: 58% Mean Age (SD): 54 (11) y Race/ Ethnicity: NR Menopausal status: NR Obesity status: NR Mean BMI (SD): 32.2 (7.2) kg/m^2^ Income level: NR Education level: NR Physical activity level: NR Health status/ Comorbidities: Hypertension: 59% Diabetes: 20% Medication use: NR Supplement use: NR Pregnant or lactating: NR  **Quintile 5**: 19.4% of Energy From Protein Intake at Baseline N: 633 % Female: 66% Mean Age (SD): 54 (11) y Race/ Ethnicity: NR Menopausal status: NR Obesity status: NR Mean BMI (SD): 33.1 (6.8) kg/m^2^ Income level: NR Education level: NR Physical activity level: NR Health status/ Comorbidities: Hypertension: 59% Diabetes: 31% Medication use: NR Supplement use: NR Pregnant or lactating: NR | **Quintile 1:** 10.4 % of Energy From Protein Intake at Baseline  Baseline Protein Amount Mean (SD): 10.4 (1.3) % of energy Carbohydrate Mean (SD): NR Fat Mean (SD): NR  Protein Amount at the end of the study Mean (SD): NR Carbohydrate Mean (SD): NR Fat Mean (SD): NR  **Quintile 2:** 12.8% of Energy From Protein Intake at Baseline  Baseline Protein Amount Mean (SD): 12.8 (0.5) % of energy Carbohydrate Mean (SD): NR Fat Mean (SD): NR  Protein Amount at the end of the study Mean (SD): NR Carbohydrate Mean (SD): NR Fat Mean (SD): NR  **Quintile 3**: 14.3% of Energy From Protein Intake at Baseline  Baseline Protein Amount Mean (SD): 14.3 (0.4) % of energy Carbohydrate Mean (SD): NR Fat Mean (SD): NR  Protein Amount at the end of the study Mean (SD): NR Carbohydrate Mean (SD): NR Fat Mean (SD): NR  **Quintile 4:** 16.0% of Energy From Protein Intake at Baseline  Baseline Protein Amount Mean (SD): 16.0 (0.6) % of energy Carbohydrate: NR Fat: NR  Protein Amount at the end of the study Mean (SD): NR Carbohydrate Mean (SD): NR Fat Mean (SD): NR  **Quintile 5**: 19.4% of Energy From Protein Intake at Baseline  Baseline Protein Amount Mean (SD): 19.4 (2.5) % of energy Carbohydrate Mean (SD): NR Fat Mean (SD): NR  Protein Amount at the end of the study Mean (SD): NR Carbohydrate Mean (SD): NR Fat Mean (SD): NR  Protein type/source: Mixed  Energy balance status: Eucaloric  Study duration: 4 y | **Protein Assessment Method:** Protein intake was estimated from a validated FFQ administered at visit 1. | **Kidney Function —** eGFR  Measure/Method of Assessment: eGFR was calculated using the Chronic Kidney Disease Epidemiology Collaboration (CKD-EPI) equation with serum creatinine.  eGFR measurement (with or without race): with race |
| PMID: 35142012 **Sekiguchi 2022 (57)** Location/Country: Japan HDI: Very high Setting: Community dwelling Urban/ Rural: Other Study Design: Prospective cohort study Funding Source: Government, academic **Risk of bias score: High** | Study of: Adults Total sample N: 1960  **Quartile 1:** 1.01 g/kg/d Protein intake at baseline N: 290 % Female: 13% Mean Age (SD): 74.8 (5.4) y Race/ Ethnicity: NR Menopausal status: NR Obesity status: NR Mean BMI (SD): 24.3 (2.95) kg/m^2^ Income level: NR Education level: NR Physical activity level: NR Health status/ Comorbidities: Hypertension: 54% Diabetes: 11% Dyslipidemia: 35% Stroke: 6.0% Heart disease: 17% Medication use: NR Supplement use: NR Pregnant or lactating: NR  **Quartile 2:** 1.32 g/kg/d Protein intake at baseline  N: 290 % Female: 39% Mean Age (SD): 76.5 (5.8) y Race/ Ethnicity: NR Menopausal status: NR Obesity status: NR Mean BMI (SD): 23.1 (2.72) kg/m^2^ Income level: NR Education level: NR Physical activity level: NR Health status/ Comorbidities: Hypertension: 54% Diabetes: 12% Dyslipidemia: 33% Stroke: 5.9% Heart disease: 17% Medication use: NR Supplement use: NR Pregnant or lactating: NR  **Quartile 3**: 1.59 g/kg/d Protein intake at baseline N: 290 % Female: 70% Mean Age (SD): 76.5 (5.8) y Race/ Ethnicity: NR Menopausal status: NR Obesity status: NR Mean BMI (SD): 22.4 (2.51) kg/m^2^ Income level: NR Education level: NR Physical activity level: NR Health status/ Comorbidities: Hypertension: 46% Diabetes: 10% Dyslipidemia: 39% Stroke: 4.8% Heart disease:16% Medication use: NR Supplement use: NR Pregnant or lactating: NR  **Quartile 4**: 2.07 g/kg/d Protein intake at baseline N: 290 % Female: 91% Mean Age (SD): 77.4 (6.3) y Race/ Ethnicity: NR Menopausal status: NR Obesity status: NR Mean BMI (SD): 20.7 (2.48) kg/m^2^ Income level: NR Education level: NR Physical activity level: NR Health status/ Comorbidities: Hypertension: 46% Diabetes: 10% Dyslipidemia: 39% Stroke: 4.8% Heart disease: 16% Medication use: NR Supplement use: NR Pregnant or lactating: NR | **Quartile 1:** 1.01 g/kg/d Protein intake at baseline  Baseline Protein Amount Mean (SD): 1.01 (0.16) g/kg/d Carbohydrate Mean (SD): NR  Fat Mean (SD): NR  Protein Amount at the end of the study Mean (SD): NR Carbohydrate Mean (SD): NR Fat Mean (SD): NR  **Quartile 2:** 1.32 g/kg/d Protein intake at baseline   Baseline Protein Amount Mean (SD): 1.32 (0.07) g/kg/d Carbohydrate Mean (SD): NR Fat Mean (SD): NR  Protein Amount at the end of the study Mean (SD): NR Carbohydrate Mean (SD): NR Fat Mean (SD): NR  **Quartile 3**: 1.59 g/kg/d Protein intake at baseline  Baseline Protein Amount Mean (SD): 1.59 (0.08) g/kg/d Carbohydrate Mean (SD): NR Fat Mean (SD): NR  Protein Amount at the end of the study Mean (SD): NR Carbohydrate Mean (SD): NR Fat Mean (SD): NR  **Quartile 4**: 2.07 g/kg/d Protein intake at baseline  Baseline Protein Amount Mean (SD): 2.07 (0.30) g/kg/d Carbohydrate Mean (SD): NR Fat Mean (SD): NR  Protein Amount at the end of the study Mean (SD): NR Carbohydrate Mean (SD): NR Fat Mean (SD): NR  Protein type/source: Mixed  Energy balance: Eucaloric  Study duration: 3 y | **Protein Assessment Method:** Dietary intakes of protein (g/day) and other nutrients during the previous month were assessed using a brief-type self-administered diet history questionnaire (BDHQ) at the baseline survey. | **Kidney Function —** eGFR  Measure/Method of Assessment: eGFR was derived from an equation of the Japanese Society of Nephrology using serum creatinine:   eGFR measurement (with or without race): with race |
| PMID: 31430246  **Shu 2019 (58)** Location/Country: China HDI: High Setting: Community dwelling Urban/ Rural: Urban Study Design: Prospective cohort study Funding Source: Government **Risk of bias score: High** | Study of: Adults Total sample N: 127,220  **Arm 1:** SWHS Subjects without incident kidney stones N: 67,715 % Female: NR Mean Age (SD): 52.4 (9.0) y Race/ Ethnicity: NR Menopausal status: NR Obesity status: 5% Obese BMI: <18.5 kg/m^2^: 3.4% 18.5-24.9 kg/m^2^: 61.6% 25-29.9 kg/m^2^: 30%  ≥30 kg/m^2^: 5% Income level:  Low: 15.9% Middle: 74.6%  High: 9.5% Education level:  Less than 12^th^ grade: 57.4% High/Vocational school: 36.1%  College or above: 4.5% Physical activity level:  Rarely/none: 65.1%  Low: 12.8%  Median: 11.3%  High: 10.9% Health status/ Comorbidities: History of coronary heart disease/stroke: 9.6% History of type 2 diabetes: 4.1% History of hypertension: 23.8% History of cholelithiasis: 10.9% Medication use: NR Supplement use: Calcium supplementation: 19% Vitamin C supplementation: 6.9% Pregnant or lactating: NR  **Arm 2:** SWHS Subjects with incident kidney stones N: 1,451 % Female: NR Mean Age (SD): 51.4 (8.3) y Race/ Ethnicity: NR Menopausal status: NR Obesity status: 5.6% Obese BMI:  <18.5 kg/m^2^: 3.0% 18.5-24.9 kg/m^2^: 58.6% 25-29.9 kg/m^2^: 32.8% ≥30 kg/m^2^: 5.6% Income level:  Low: 13.6% Middle: 75.9% High: 10.5% Education level: Less than 12th grade: 55.0% High/Vocational School: 39.8% College or above: 5.2% Physical activity level: Rarely/none: 67.1%  Low: 12.4%  Median: 11%  High: 9.5% Health status/ Comorbidities: History of coronary heart disease/stroke: 11.6% History of type 2 diabetes: 4.4% History of hypertension: 25.8% History of cholelithiasis: 12.7% Medication use: NR Supplement use: Calcium supplementation: 21.4% Vitamin C supplementation: 7.1% Pregnant or lactating: NR  **Arm 3:** SMHS subjects without incident kidney stones N: 56,852 % Female: NR Mean Age (SD): 55.3 (9.7) y Race/ Ethnicity: NR Menopausal status: NR Obesity status: 2.5% Obese BMI:  <18.5 kg/m^2^: 4.3% 18.5-24.9 kg/m^2^: 62.9% 25-29.9 kg/m^2^: 30.3% ≥30 kg/m^2^: 2.5% Income level:  Low: 12.6% Middle: 77.8% High: 9.6% Education level: Less than 12th grade: 41.3% High/Vocational School: 47.6% College or above: 11.1% Physical activity level: 64.6% Rarely/none: 64.6%  Low: 11.9% Median: 12.4%  High: 11.1% Health status/ Comorbidities: History of coronary heart disease/stroke: 9.0%  History of type 2 diabetes: 6.2%  History of hypertension: 30.3% History of cholelithiasis: 7.4% Medication use: NR Supplement use: Calcium supplementation: 4.7% Vitamin C supplementation: 5.5% Pregnant or lactating: NR  **Arm 4:** SMHS Subjects with incident kidney stones N: 1,202 % Female: NR Mean Age (SD): 54.4 (9.1) y Race/ Ethnicity: NR Menopausal status: NR Obesity status: 3.4% Obese BMI: <18.5 kg/m^2^: 2.8% 18.5-24.9 kg/m^2^: 58.3% 25-29.9 kg/m^2^: 35.4% ≥30 kg/m^2^: 3.4% Income level:  Low: 11.5% Middle: 78.2% High:10.3% Education level: Less than 12th grade: 35.5% High/Vocational School: 52.1% College or above: 12.4% Physical activity level: Rarely/none: 66.1%  Low: 12.4%, 12.5%  Median: 12.5% High: 9.0%  Health status/ Comorbidities: History of coronary heart disease/stroke: 8.0% History of type 2 diabetes: 5.9% History of hypertension: 35.3% History of cholelithiasis: 7.6% Medication use: NR Supplement use: Calcium supplementation: 5.2% Vitamin C supplementation: 6.5% Pregnant or lactating: NR | **Arm 1**: SWHS Subjects without incident kidney stones   Baseline Protein Amount Mean (SD): 67.1 (20.6) g/d Carbohydrate Mean (SD): NR Fat Mean (SD): NR  Protein Amount at the end of the study Mean (SD): NR Carbohydrate Mean (SD): NR Fat Mean (SD): NR  **Arm 2:** SWHS Subjects with incident kidney stones   Baseline Protein Amount Mean (SD): 67.5 (20.7) g/d Carbohydrate Mean (SD): NR Fat Mean (SD): NR  Protein Amount at the end of the study Mean (SD): NR Carbohydrate Mean (SD): NR Fat Mean (SD): NR  **Arm 3**: SMHS subjects without incident kidney stones   Baseline Protein Amount Mean (SD): 78.4 (23.6) g/d Carbohydrate Mean (SD): NR Fat Mean (SD): NR  Protein Amount at the end of the study Mean (SD): NR Carbohydrate Mean (SD): NR Fat Mean (SD): NR  **Arm 4**: SMHS Subjects with incident kidney stones   Baseline Protein Amount Mean (SD): 79.6 (23.3) g/d Carbohydrate: NR Fat: NR  Protein Amount at the end of the study Mean (SD): NR Carbohydrate Mean (SD): NR Fat Mean (SD): NR  Protein type/source: Mixed  Energy balance status: Eucaloric  Study duration: 8 y | **Protein Assessment Method:** FFQ according to the China Food Composition table at baseline. | **Kidney stones**  Measure/Method of Assessment: Incident kidney stone was ascertained as the first report of a urinary tract stone located in the kidney or the ureter during follow-up visits. |
| PMID: 36532536 **Teymoori 2022 (59)** Location/Country: Iran HDI: High Setting: Community dwelling Urban/ Rural: Urban Study Design: Prospective cohort study  Funding Source: Academic, government **Risk of bias score: High** | Study of: Adults Total sample N: 6044  **Tertile 1:** Protein score (8.2 ± 2.8) N: 2561 % Female: 57.7% Mean Age (SD): 36.1 (12.1) y Race/ Ethnicity: NR Menopausal status: NR Obesity status: NR Mean BMI (SD): 26.4 (4.7) kg/m^2^ Income level: NR Education level: Academic education: 22.6% Mean physical activity level (SD): 72.7(60.8) MET/h/wk  Health status/ Comorbidities: NR  Medication use: NR Supplement use: NR Pregnant or lactating: NR  **Tertile 2**: Protein score (9.6 ± 2.8) N: 1714 % Female: 54.9% Mean Age (SD): 37.8 (12.8) y Race/ Ethnicity: NR Menopausal status: NR Obesity status: NR Mean BMI (SD): 27.0 (4.8) kg/m^2^ Income level: NR Education level: Academic education: 26.1% Mean physical activity level (SD): 74.7 (64.2) MET/h/wk Health status/ Comorbidities: NR  Medication use: NR Supplement use: NR Pregnant or lactating: NR  **Tertile 3**: Protein score (12.0 ± 3.1) N: 1769 % Female: 48.8% Mean Age (SD): 40.4 (13.3) y Race/ Ethnicity: NR Menopausal status: NR Obesity status: NR Mean BMI (SD): 27.1 (4.6) kg/m^2^ Income level: NR Education level: Academic education: 24.6% Mean physical activity level (SD): 75.4 (64.0) MET/h/wk Health status/ Comorbidities: NR  Medication use: NR Supplement use: NR Pregnant or lactating: NR | **Tertile 1:** Protein score (8.2 ± 2.8)  Baseline Protein Amount Mean (SD): 13.1 (1.8) % of energy Carbohydrate Mean (SD): 55.9 (6.7) % of energy Fat Mean (SD): 33.4 (6.5) % of energy  Protein Amount at the end of the study Mean (SD): NR Carbohydrate Mean (SD): NR Fat Mean (SD): NR  **Tertile 2:** Protein score (9.6 ± 2.8)  Baseline Protein Amount Mean (SD): 14.8 (3.2) % of energy Carbohydrate Mean (SD): 58.4 (6.7) % of energy Fat Mean (SD): 29.6 (5.7) % of energy  Protein Amount at the end of the study Mean (SD): NR Carbohydrate Mean (SD): NR Fat Mean (SD): NR  **Tertile 3**: Protein score (12.0 ± 3.1)  Baseline Protein Amount Mean (SD): 16.1 (9.8) % of energy Carbohydrate Mean (SD): 61.5 (9.8) % of energy Fat Mean (SD): 27.7 (21.4) % of energy  Protein Amount at the end of the study Mean (SD): NR Carbohydrate Mean (SD): NR Fat Mean (SD): NR  Protein type/source: Mixed  Energy balance status: Eucaloric  Study duration: 19 y | **Protein Assessment Method:** Dietary data were assessed using a valid and reliable semi-quantitative 168-item food frequency questionnaire. During a face-to-face interview, the frequency of consumption for each food item during the past year on a daily, weekly, or monthly basis was collected by trained and skilled dieticians. Protein was measured at baseline. | **Kidney Function** **—** Incident CKD  Measure/Method of Assessment: CKD was ascertained using eGFR < 60 ml/min/1.73 m^2^ and eGFR was calculated using the Chronic Kidney Disease Epidemiology Collaboration (CKD-EPI) equation with serum creatinine.  eGFR CKD cut off point: eGFR <60 ml/min/1.73 m2  eGFR measurement (with or without race): without race |

**Abbreviations:** BMI = Body Mass Index; CAD = coronary artery disease; CI = Confidence Interval; CHF = congestive heart failure; CKD = chronic kidney disease; CVD = cardiovascular disease; d = day; eGFR = estimated glomerular filtration rate; ESRD = end stage renal disease; FFQ = Food frequency questionnaire; g = grams; GFR = glomerular filtration rate; h = hours; HDI = human developement index; kg = kilograms; kg/m^2^ = kilogram per meters squared; m^2^ = meters squared; MDRD = Modification of Diet in Renal Disease; METs = metabolic equivalents; mg = milligrams; MI = myocardial infarction; min = minutes; ml = milliliter; mmol/L = millimols per liter; NR = not reported; PMID = PubMed Identification Number; RoB = Risk of Bias; SD = Standard deviation; SE = standard error; SMHS = Shanghai Men’s Health Study; SWHS = Shanghai Women’s Health Study; USA = United States of America; UUN = Urine urea nitrogen; wk = week; y = years
^1^Studies overlap KQs

Supplementary Table 6. Evidence table for Sarcopenia Randomized Controlled Trials (Adults)

| Study | Participants | Intervention(s) (Content) | Intervention(s) (Methods of administration and assessment) | Outcome (Measures and methods of assessment) |
| --- | --- | --- | --- | --- |
| PMID: 26471344 **Backx** **2016 (60)** Location/Country: Netherlands HDI: Very high Setting: Community dwelling Urban/Rural: NR Study design: RCT (parallel) Funding Source: Public-private partnership **Risk of bias score: Low** | Study of: Adults Total sample N: 61  **Intervention:** High Protein diet N: 31 % Female: 41.9% Mean Age (SD): 63 (4.8) y Race/ Ethnicity: NR Menopausal Status: Postmenopausal Obesity Status: All overweight or obese with BMI between 27 and 40 kg/m^2^ Mean BMI (SD): 31.3 (3.0) kg/m^2^ Income level: NR Education level: NR Mean physical activity level (SD): 916 (203) cpm Health status/ Comorbidities: Excluded renal insufficiency, type 1 or type 2 diabetes, cancer, COPD, previous gastric bypass Medication use: NR Supplement use: Excluded if used supplements or drugs known to interfere with energy balance used within 3 months prior Pregnant or lactating: NR  **Comparator:** Normal Protein diet N: 30 % Female: 40% Mean Age (SD): 62 (4.8) y Race/ Ethnicity: NR Menopausal Status: Postmenopausal Obesity Status: All overweight or obese with BMI between 27 and 40 kg/m^2^ Mean BMI (SD): 31.0 (2.9) kg/m^2^ Income level: NR Education level: NR Mean physical activity level (SD): 825 (258) cpm Health status/ Comorbidities: Excluded renal insufficiency, type 1 or type 2 diabetes, cancer, COPD, previous gastric bypass Medication use: NR Supplement use: Excluded if used supplements or drugs known to interfere with energy balance used within 3 months prior Pregnant or lactating: NR | **Intervention:** High Protein diet  Intended Protein Amount: 1.7 g/kg/d Carbohydrate: NR Fat: NR  Baseline Protein Amount  Mean (SD): 1.1 (0.4) g/kg/d; 14% of energy Carbohydrate Mean: 51% of energy Fat Mean: 31% of energy  Actual Protein Amount at the end of study  Mean: 1.69 g/kg/d; 34% of energy Carbohydrate Mean: 35% of energy Fat Mean: 27% of energy  Dietary Protein Intake Compliance (%): NR – high level of compliance stated for both intervention and comparator group  Protein type/source: Mixed  Energy balance status: Hypocaloric  **Comparator**: Normal Protein diet  Intended Protein Amount: 0.9 g/kg/day Carbohydrate: NR Fat: NR  Baseline Protein Amount Mean (SD): 1.1 (0.4) g/kg/d; 14% of energy Carbohydrate Mean: 51% of energy Fat Mean: 31% of energy  Actual Protein Amount at the end of study Mean: 0.92 g/kg/d; 19% of energy Carbohydrate Mean: 51% of energy Fat Mean: 24% of energy  Dietary Protein Intake Compliance (%): NR – high level of compliance stated for both intervention and comparator group  Protein type/source: Mixed  Energy balance status: Hypocaloric  Study duration: 12 weeks | **Intervention:** High Protein diet  **How protein was administered:** Provided 90% of diet and 2 supplements (20 g protein) per day to consume 1.7 g of protein/kg/day  **Protein Assessment Method:** Baseline protein amount was derived from a validated 177-item food frequency questionnaire.  Actual Protein Amount was derived from analysis of stored complete diet collected for each group throughout the intervention. Food diaries were used to assess the 10% diet chosen by the subjects.  **Dietary Protein Intake Compliance:** Compliance was assured via daily contact (weekdays) with the investigators and dietitians.  **Comparator:** Normal Protein diet  **How protein was administered:** Provided 90% of diet and 2 supplements (25 g carbohydrates) per day to consume 0.9 g of protein/kg/day  **Protein Assessment Method:** Same as above  **Dietary Protein Intake Compliance:** Same as above | **Muscle Strength -** 1-RM leg press  Measure/Method of Assessment: Maximum leg strength was assessed by 1-RM strength tests on leg press and leg strength machine  **Muscle Strength -** 1-RM leg extension  Measure/Method of Assessment: Maximum leg strength was assessed by 1-RM strength tests on leg press and leg strength machine  **Muscle Strength -** Handgrip Strength  Measure/Method of Assessment: Measured using handgrip dynamometer  **Physical Performance -** SPPB  Measure/Method of Assessment: The SPPB consists of three components: balance, gait speed and chair rise ability  **Physical Performance** - 400m walk speed Measure/Method of Assessment: The 400m walk test assessed the time it takes to walk 400m  **Muscle Mass -** Lean body mass  Measure/Method of Assessment: DXA (model DPX-L)  **Muscle Mass –** Appendicular lean body mass/ skeletal muscle mass  Measure/Method of Assessment: DXA (model DPX-L) |
| PMID: 33975325 **Englert** **2021 (61)** Location/Country: Germany HDI: Very high Setting: Community dwelling Urban/Rural: NR Study design: RCT (parallel) Funding source: Academic, industry **Risk of bias score: Low** | Study of: Adults Total sample N: 54  **Intervention:** High Protein N: 27 % Female: 100% Mean Age (SD): 59.0 (6) y Race/ Ethnicity: NR Menopausal Status: Postmenopausal Obesity Status: All women overweight, BMI ≥ 30 or ≥ 27 and waist circumference >88 cm Mean BMI (SD): 30.5 (2.8) kg/m^2^ Income level: NR Education level: NR Mean physical activity level (SD): PAL 1.4 (0.1)  Health status/ Comorbidities: Excluded type 2 diabetes; thyroid disease; kidney, heart, or liver failure; neurological disease; electronic implants; active prostheses; life-sustaining electronic devices Medication use: Excluded medications like steroids, diuretics, thyroid drugs, statins, weight loss medication, beta blockers Supplement use: Excluded protein supplementation during the last 3 months Pregnant or lactating: NR  **Comparator:** Normal Protein N: 27 % Female: 100% Mean Age (SD): 58.7 (6) y Race/ Ethnicity: NR Menopausal Status: Postmenopausal Obesity Status: All women overweight, BMI ≥ 30 or ≥ 27 and waist circumference >88 cm Mean BMI (SD): 31.3 (4) kg/m^2^ Income level: NR Education level: NR Mean physical activity level (SD): PAL 1.4 (0.1) Health status/ Comorbidities: Excluded type 2 diabetes; thyroid disease; kidney, heart, or liver failure; neurological disease; electronic implants; active prostheses; life-sustaining electronic devices Medication use: Excluded medications like steroids, diuretics, thyroid drugs, statins, weight loss medication, beta blockers Supplement use: Excluded protein supplementation during the last 3 months Pregnant or lactating: NR | **Intervention:** High Protein  Intended Protein Amount: 1.5 g/kg/d Carbohydrate: NR Fat: NR  Baseline Protein Amount Mean (SD): NR Carbohydrate Mean (SD): NR Fat Mean (SD): NR  Actual Protein Amount at the end of study Mean (SD): 113 (17) g/d Carbohydrate Mean (SD): 127 (18) g/d Fat Mean (SD): 46 (9) g/d  Dietary Protein Intake Compliance (%): Compliance was the same in both intervention and comparator groups – good compliance rate stated for both intervention and comparator group  Protein type/source: Mixed  Energy balance status: Hypocaloric  **Comparator**: Normal Protein  Intended Protein Amount: 0.8 g/kg/d Carbohydrate: NR Fat: NR  Baseline Protein Amount Mean (SD): NR Carbohydrate Mean (SD): NR Fat Mean (SD): NR  Actual Protein Amount at the end of study Mean (SD): 63 (9) g/day Carbohydrate Mean (SD): 136 (29) g/day Fat Mean (SD): 48 (11) g/day  Dietary Protein Intake Compliance (%): Compliance was the same in both intervention and comparator groups – good compliance rate stated for both intervention and comparator group  Protein type/source: Mixed  Energy balance status: Hypocaloric  Study duration: 12 weeks intervention, 6 months of follow-up | **Intervention:** High Protein  **How protein was administered:** 2 provided meal replacement shakes, 3^rd^ meal and/or snack chosen from the individual’s diet plan to reach 1.5 g of protein/kg/day  **Protein Assessment Method:** Participants kept a food diary after the first and third session with dietitian for 7 consecutive days and food checklists on the remaining days. Dietary energy and macronutrient intake data were recorded in the first and third quarters of the intervention. The mean value for macronutrients was reported.  **Dietary Protein Intake Compliance:** 4 nutrition training sessions for both groups separately and telephone interviews to enhance compliance  **Comparator:** Normal Protein  **How protein was administered:** 2 provided meal replacement shakes, 3^rd^ meal and/or snack chosen from the individual’s diet plan to reach 0.8 g of protein/kg/day  **Protein Assessment Method:** Same as above  **Dietary Protein Intake Compliance:** Same as above | **Physical Performance -**SPPB Measure/Method of Assessment: The SPPB consists of three components: balance, gait speed and chair rise ability  **Physical Performance -** 400 m walk speed  Measure/Method of Assessment: Walked as fast as they could without running for 400 meters   **Muscle Strength -** Handgrip strength   Measure/Method of Assessment: Measured using handgrip dynamometer  **Muscle Mass -** Fat Free Mass  Measure/Method of Assessment: BIA (seca mBCA 515/514) |
| PMID: 20578205 **Flechtner-Mors** **2010^1^ (36)** Location/Country: Germany HDI: Very high Setting: Outpatient clinic Urban/Rural: NR Study Design: RCT (parallel) Funding source: Industry, academic **Risk of bias score: High** | Study of: Adults Total sample N: 110  **Intervention:** High Protein  N: 55 % Female: 78.2% Mean Age (SD): 49.3 (12.3) y Race/ Ethnicity: NR Menopausal status: NR Obesity status: Obese Mean BMI (SD): 36.2 (4.4) kg/m^2^ Income level NR Education level: NR Physical activity level: Received instructions to maintain their usual physical activity during the study and not to undertake any new exercise programs, but exercise was not monitored Health status/ Comorbidities: Included: Those that met the criteria for metabolic syndrome Medication use: Exclude: anti-obesity medications Supplement use: NR Pregnant or lactating: Excluded  **Comparator:** Conventional Diet N: 55% Female: 81.2% Mean Age (SD): 50 (13) y Race/ Ethnicity: NR Menopausal status: NR Obesity status: Obese Mean BMI (SD): 36.3 (5.0) kg/m^2^ Income level NR Education level: NR Physical activity level: Received instructions to maintain their usual physical activity during the study and not to undertake any new exercise programs, but exercise was not monitored Health status/ Comorbidities: Included: Those that met the criteria for metabolic syndrome Medication use: Exclude: anti-obesity medications Supplement use: NR Pregnant or lactating: Excluded | **Intervention:** High Protein  Intended Protein Amount: 1.34 g/kg/d; 30% of energy Carbohydrate: 40% of energy Fat: 30% of energy  Baseline Protein Amount Mean (SD):18.0 (4.9) % of energy; 72.7 (24.3) g/d Carbohydrate Mean (SD): 46.7 (9.4) % of energy; 194 (73) g/d Fat: 35.2 (7.6) % of energy; 64 (25) g/d  Actual Protein Amount at the end of the study Mean (SD): 30.0 (7.0) % of energy; 92.2 (14.8) g/d Carbohydrate Mean (SD): 36.9 (7.9) % of energy; 119 (45) g/d Fat Mean (SD): 29.9 (5.7) % of energy; 42 (13) g/d  Dietary Protein Intake Compliance (%): 56.3%  Protein type/source: Mixed  Energy balance status: Hypocaloric  **Comparator:** Conventional Diet  Intended Protein Amount: 0.8 g/kg/d; 15% energy  Carbohydrate: 30% energy Fat: 55% energy  Baseline Protein Amount Mean (SD): 17.0 (4.7) % of energy; 66.4 (22.7) g/d Carbohydrate Mean (SD): 48.2 (9.4) % of energy; 188 (64) g/d Fat Mean (SD): 34.6 (7.3) % of energy; 60 (26) g/d  Actual Protein Amount at the end of the study Mean (SD): 21.4 (7.4) % of energy; 65.7 (14.7) g/d Carbohydrate Mean (SD): 47.6 (7.5) % of energy; 154 (44) g/d Fat: 29.6 (5.7) % of energy; 44 (16) g/d  Dietary Protein Intake Compliance (%): NR  Protein type/source: Mixed  Energy balance status: Hypocaloric  Study duration: 12 months | **Intervention:** High Protein   **How protein was administered:**  First 3 months: Consumed two protein-enriched meal replacements, one conventional meal, and two snacks as either a protein bar or a low-fat curd with fruit.  After the first 3 months: Consumed one protein-enriched meal replacement, two meals, and two snacks  **Protein Assessment Method:** Subjects kept 3-day food records at baseline, 3 months, 6 months, 9 months, and 12 months. Food quantities were recorded using standard household measures, and a trained assessment dietician reviewed the food records in person. Nutrient calculations were carried out using the PRODI program which is based on German food-composition tables.  **Dietary Protein Intake Compliance:** Food records yielded data that revealed adherence to the dietary recommendations during the study  **Comparator:** Conventional Diet  **How protein was administered:**  First 3 months: Consumed three meals and two snacks with no replacements  After 3 months: Consumed one standard meal replacement, two meals, and two snacks per day  **Protein Assessment Method:** Same as above  **Dietary Protein Intake Compliance:** Same as above | **Muscle Mass –** Fat-free mass  Measure/Method of Assessment: BIA (Bioimpedance Analyzer 450, Biodynamics, Seattle, Washington, USA) |
| PMID: 18371214 **Frestedt** **2008^1^** **(37)** Location/Country: USA HDI: Very high Setting: Community dwelling Urban/Rural: NR Study Design: RCT (parallel) Funding source: Industry **Risk of bias score: High** | Study of: Adults Total sample N: 59  **Intervention**: Prolibra  N: 31 % Female: NR Mean Age (SE): 43.6 (1.1) y Race/ Ethnicity: NR Menopausal status: NR Obesity status: Yes Mean BMI (SE): 35.7 (0.7) kg/m^2^ Income level NR Education level: NR Physical activity level: NR Health status/ Comorbidities: NR Medication use: NR Supplement use: NR Pregnant or lactating: NR  **Comparator**: Placebo N: 28 % Female: NR Mean Age (SE): 42 (1.2) y Race/ Ethnicity: NR Menopausal status: NR Obesity status: Yes Mean BMI (SE): 35.4 (0.7) kg/m^2^ Income level NR Education level: NR Physical activity level: NR Health status/ Comorbidities: NR Medication use: NR Supplement use: NR Pregnant or lactating: NR | **Intervention:** Prolibra  Intended Protein Amount: Supplement 20 g of protein/d (1-10g protein supplement twice daily); 15% of energy Carbohydrate: 55% of energy Fat: 30% of energy  Baseline Protein Amount Mean (SD): 73 (3) g/d; 0.74 g/kg/d Carbohydrate Mean (SD): 222 (11) g/d Fat Mean (SD): 75 (5) g/d  Actual Protein Amount at the end of the study Mean (SD): 0.81 g/kg/d (with supplement); 0.60 g/kg/d and 57 (3) g/d (w/o supplement) Carbohydrate Mean (SD): 178 (8) g/d (w/o supplement) Fat Mean (SD): 49 (3) g/d (w/o supplement)  Dietary Protein Intake Compliance (%): NR  Protein type/source: Animal; whey protein   Energy balance status: Hypocaloric  **Comparator:** Placebo  Intended Protein Amount: 15% of energy Carbohydrate: 55% of energy Fat: 30% of energy  Baseline Protein Amount Mean (SD): 74 (4) g/d; 0.76 g/kg/d Carbohydrate Mean (SD): 211 (10) g/d Fat Mean (SD): 71 (5) g/d  Actual Protein Amount at the end of the study Mean (SD): 0.61 g/kg/d (with supplement); 58 (2) g/d (w/o supplement) Carbohydrate Mean (SD): 182 (9) g/d (w/o supplement) Fat Mean (SD): 47 (3) g/d (w/o supplement)  Dietary Protein Intake Compliance (%): NR  Protein type/source: Mixed  Energy balance status: Hypocaloric  Study duration: 12 weeks | **Intervention:** Prolibra   **How protein was administered:** One Prolibra supplement before breakfast and one before dinner. Each supplement contained 10 g of protein. Subjects were assigned a diet plan with a certain number of servings for various food groups similar to the standard paradigm set by the American Heart Association.  **Protein Assessment Method:** Total protein in Prolibra was measuring using Kjeldahl (AOAC 945.01). Subjects completed diet diaries on at least 5 days each month.  **Dietary Protein Intake Compliance:** Compliance was assessed by supplement count and diet diary review. Participants were also contacted by telephone between visits to review diet and supplement compliance.  **Comparator:** Placebo  **How protein was administered:** Subjects received an iso-caloric beverage containing maltodextrin. Subjects were assigned a diet plan with a certain number of servings for various food groups similar to the standard paradigm set by the American Heart Association.  **Protein Assessment Method:** Same as above  **Dietary Protein Intake Compliance:** Same as above | **Muscle Mass -** Lean muscle mass  Measure/Method of Assessment: DXA (Lunar Prodigy Advance Plus, General Electric, Madison, WI) |
| PMID: 34208986 **Haghighat** **2021 (62)** Location/Country: Iran HDI: High Setting: Community dwelling Urban/Rural: NR Study design: RCT (parallel) Funding source: Academic  **Risk of bias score: Moderate** | Study of: Adults Total sample N: 120  **Intervention:** High Protein N: 60 % Female: 100% Mean Age (SD): 24 (3) y (total sample average) Race/ Ethnicity: NR Menopausal Status: Premenopausal Obesity Status: Normal weight obesity (body fat percentage >30%) Mean BMI (SD): NR Income level: NR Education level: NR Physical activity level: 26% low, 63% moderate Health status/ Comorbidities: Excluded history or presence of bariatric surgery, any acute or chronic diseases, psychiatric disorders Medication use: Excluded “medication use” Supplement use: Excluded those that consumed more than 300 mg of caffeine daily Pregnant or lactating: Excluded  **Comparator:** Low Protein N: 60 % Female: 100% Mean Age (SD): 24 (3) y (total sample average) Race/ Ethnicity: NR Menopausal Status: Premenopausal Obesity Status: Normal weight obesity (body fat percentage >30%) Mean BMI (SD): NR Income level: NR Education level: NR Physical activity level: 31% low, 69% moderate Health status/ Comorbidities: Excluded history or presence of bariatric surgery, any acute or chronic diseases, psychiatric disorders Medication use: Excluded “medication use” Supplement use: Excluded those that consumed more than 300 mg of caffeine daily Pregnant or lactating: Excluded | **Intervention**: High Protein  Intended Protein Amount: 18.2 g of protein during snack, no goal for total dietary protein Carbohydrate: NR Fat: NR  Baseline Protein Amount Mean (SD): 51.37 (7.36) g/d; 0.84 (0.15) g/kg/d Carbohydrate Mean (SD): 253.48 (39.24) g/d Fat Mean (SD): 48.87 (5.42) g/d  Actual Protein Amount at the end of the study Mean (SD): 74.94 (6.40) g/d; 1.28 (0.2) g/kg/d Carbohydrate Mean (SD): 195.04 (33.47) g/d Fat Mean (SD): 45.88 (8.37) g/d  Dietary Protein Intake Compliance (%): 86.6%  Protein type/source: Plant  Energy balance status: Eucaloric  **Comparator**: Low Protein  Intended Protein Amount: <2 g of protein during snack, no goal for total dietary protein Carbohydrate: NR Fat: NR  Baseline Protein Amount Mean (SD): 48.80 (7.21) g/d; 0.79 (0.14) g/kg/d Carbohydrate Mean (SD): 247.05 (57.55) g/d Fat Mean (SD): 46.36 (7.97) g/d  Actual Protein Amount at the end of the study Mean (SD): 55.02 (6.30) g/d; 0.87 (0.12) g/kg/d Carbohydrate Mean (SD): 253.45 (55.55) g/d Fat Mean (SD): 50.91 (9.56) g/d  Dietary Protein Intake Compliance (%): 91.6%  Protein type/source: Mixed  Energy balance status: Eucaloric  Study duration: 6 months | **Intervention:** High Protein  **How protein was administered**: High protein content snack (50 g of soybeans equaling 18.2 g protein) daily at 10 a.m.  **Protein Assessment Method:** 24-h dietary recalls were completed by all participants on three occasions over a week time period (one weekday and two weekend days) prior to and at the end of study. Calorie and macronutrient combinations were assessed using the Nutritionist IV for Windows software program (The Hearst Corporation, San Bruno, CA)  **Dietary Protein Intake Compliance:** Laboratory on two other occasions (at the end of months 2 and 4) to report snack compliance. Additional snack compliance reporting was performed once per week by phone or WhatsApp software.  **Comparator:** Low Protein  **How protein was administered:** Low protein content snack (~3.5 servings of fruit equaling <2 g protein) daily at 10 a.m.  **Protein Assessment Method:** Same as above  **Dietary Protein Intake Compliance:** Same as above | **Muscle Mass -** Skeletal Muscle Mass   Measure/Method of Assessment: BIA (SECA Model 222; Seca, Germany) |
| PMID: 24047916 **Jesudason** **2013^1^ (8)** Location/Country: Australia HDI: Very high Setting: Community dwelling Urban/Rural: NR Study Design:  RCT (Parallel)  Funding source: Government **Risk of bias score: High** | Study of: Adults Total sample N: 323   **Intervention:** High protein N: 164 % Female: 100% Mean Age (SE): 59.5 (0.4) y Race/ Ethnicity: NR Menopausal status: Postmenopausal Pubertal status: NA Obesity status: Obese Mean BMI (SD): 34.0 (0.4) kg/m^2^ Income level: NR Education level: NR Physical activity level: NR Health status/Co-morbidities: Subjects with parathyroid disease, a vitamin D concentration, 60 nmol/L with secondary hyperparathyroidism, or unstable metabolic, cardiac, gastrointestinal, renal, or other significant disease, including malignancies, were excluded Medication use: Women were ineligible if they were taking hormone-replacement therapy, bisphosphonates, steroids, diuretics, calcium, or vitamin D Supplement use: Women were ineligible if they were taking calcium or vitamin D Pregnant or lactating: NA  **Comparator**: Normal protein N: 159 % Female: 100% Mean Age (SD): 59.4 (0.4) y Race/Ethnicity: NR Menopausal status: Postmenopausal Pubertal status: NA Obesity status: Obese Mean BMI at baseline: 33.4 (0.4) kg/m^2^ Income level: NR Education level: NR Physical activity level: NR Health status/Co-morbidities: Subjects with parathyroid disease, a vitamin D concentration, 60 nmol/L with secondary hyperparathyroidism, or unstable metabolic, cardiac, gastrointestinal, renal, or other significant disease, including malignancies, were excluded Medication use: Women were ineligible if they were taking hormone-replacement therapy, bisphosphonates, steroids, diuretics, calcium, or vitamin D Supplement use: Women were ineligible if they were taking calcium or vitamin D Pregnant or lactating: NA | **Intervention:** High protein  Intended Protein Amount: 32% of energy Carbohydrate: 44% of energy Fat: 24% of energy  Baseline Protein Amount Mean (SD): 92.5 (2.2) g/day; 18.6 (0.2) % of energy Carbohydrate Mean (SD): 230 (6) g/day; 42.9 (0.5) % of energy Fat Mean (SD): 79.2 (2.7) g/day; 33.3 (0.4) % of energy  Actual Protein Amount at the end of the study Mean (SD): 91.5 (2.2) g/day; 21.9 (0.3) % of energy Carbohydrate Mean (SD): 196 (6) g/day; 43.9 (0.7) % of energy Fat Mean (SD): 55.5 (2.3) g/day; 28.2 (0.7) % of energy  Dietary Protein Intake Compliance (%): NR  Protein type/source: Mixed  Energy balance status: Hypocaloric  **Comparator:** Normal protein   Intended Protein Amount: 22% of energy Carbohydrate: 55% of energy Fat: 23% of energy  Baseline Protein Amount Mean (SD): 91.2 (1.9) g/day; 18.4 (0.2) % of energy Carbohydrate Mean (SD): 228 (5) g/day; 42.9 (0.5) % of energy Fat Mean (SD): 77.7 (2.1) g/day; 33.4 (0.4) % of energy  Actual Protein Amount at the end of the study Mean (SD): 80.6 (2.2) g/day; 18.9 (0.3) % of energy Carbohydrate Mean (SD): 214 (5) g/day; 47.2 (0.6) % of energy Fat Mean (SD): 57.9 (2.5) g/day; 28.6 (0.7) % of energy  Dietary Protein Intake Compliance (%): NR  Protein type/source: Mixed  Energy balance status: Hypocaloric  Study duration: 24 months | **Intervention:** High protein  **How protein was administered:** Participants received monthly group dietetic education and support for the first 6 months and then every 3 months for the next 18 months. Sample food packs of $20 vouchers were provided to participants at baseline and 12 and 26 weeks. Each diet group was allocated to a protein target that was based on key protein foods as a compliance measure.   **Protein Assessment Method:** Participants recorded dietary intakes using a protein counter and checklist. Protein compliance checklists were collected from each participant at each group session. Subjects also completed a FFQ at baseline and 1 and 2 y.   **Dietary Protein Intake Compliance:** Compliance was assessed by (1) blood urea nitrogen and 24h urine for urea nitrogen excretion (2) allocated to a protein target for each diet group and (3) protein-compliance checklists were collected from each participant at each group session.   **Comparator:** Normal protein  **How protein was administered:** Participants received monthly group dietetic education and support for the first 6 months and then every 3 months for the next 18 months. Sample food packs of $20 vouchers were provided to participants at baseline and 12 and 26 weeks. Each diet group was allocated to a protein target that was based on key protein foods as a compliance measure.  **Protein Assessment Method:** Same as above **Dietary Protein Intake Compliance:** Same as above | **Muscle Mass -** Lean Mass  Measures/Method of Assessment: NR |
| PMID: 25844619 **Kerstetter 2015^1^**^,2^ **(9)** Location/Country: USA HDI: Very high Setting: NR Urban/ Rural: NR Study design: RCT (parallel) Funding source: Government, academic  **Risk of bias score: Low** | Study of: Adults Total sample N: 208  **Intervention:** High Protein N: 106 % Female: 84% Mean Age (SD): 69.9 (6.1) y Race/ Ethnicity: NR Menopausal status: NR Obesity status: NR Mean BMI (SD): 26.1 (3.4) kg/m^2^  Income level: NR Education level: NR Mean physical activity level score (SD): 6.7 (2.1) Health status/ Comorbidities: Healthy older adults Medication use: Excluded if using long-term chemotherapeutic drugs, aromatase inhibitors or tamoxifen, methotrexate, phenytoin, phenobarbital or inhaled corticosteroids (greater than 800 ug/day), actively being treated for leukemia or multiple myeloma, a change in thyroid medications, medications known to affect calcium metabolism or use of proton pump inhibitors twice daily Supplement use: Daily multivitamin mineral supplement (contained 400 IU of vitamin D); Ca carbonate supplement (300 mg tablets) Pregnant or lactating: NR  **Comparator:** Low Protein N: 102 % Female: 87.3% Mean Age (SD): 70.5 (6.4) y Race/ Ethnicity: NR Menopausal status: NR Obesity status: NR Mean BMI (SD): 26.4 (4.0) kg/m^2^ Income level: NR Education level: NR Mean physical activity level score (SD): 6.8 (1.9) Health status/ Comorbidities: Healthy older adults Medication use: Excluded if using long-term chemotherapeutic drugs, aromatase inhibitors or tamoxifen, methotrexate, phenytoin, phenobarbital or inhaled corticosteroids (greater than 800 ug/day), actively being treated for leukemia or multiple myeloma, a change in thyroid medications, medications known to affect calcium metabolism or use of proton pump inhibitors twice daily Supplement use: Daily multivitamin mineral supplement (contained 400 IU of vitamin D); Ca carbonate supplement (300 mg tablets) Pregnant or lactating: NR | **Intervention:** High Protein Intended Protein Amount: 40 g of protein from the supplement; total daily protein goal NR Carbohydrate: Test food protein NR Fat: Test food protein NR  Baseline Protein Amount Least Square Mean (SEM): 73.8 (1.9) g/d Carbohydrate Least Square Mean (SEM): 214.1 (5.2) g/d Fat Least Square Mean (SEM): 59.4 (2.1) g/d  Actual Protein Amount at the end of the study Least Square Mean (SEM): 90.7 (3.3) g/d Carbohydrate Least Square Mean (SEM): 196.9 (6.6) g/d Fat Least Square Mean (SEM): 55.6 (2.0) g/d  Dietary Protein Intake Compliance (%): NR  Protein type/source: Animal; whey supplement   Energy balance status: Eucaloric  **Comparator:** Low Protein  Intended Protein Amount: Test food protein NR Carbohydrate: Test food protein NR Fat: Test food protein NR  Baseline Protein Amount  Least Square Mean (SEM): 72.9 (1.8) g/d; 1.06 (0.03) g/kg/d (total daily) Carbohydrate Least Square Mean (SEM): 206.2 (5.8) g/d (total daily) Fat Least Square Mean (SEM): 61.3 (2.5) g/d (total daily)  Actual Protein Amount at end of the study  Least Square Mean (SEM): 72.7 (2.4) g/d; 1.05 (0.04) g/kg/d (total daily) Carbohydrate Least Square Mean (SEM): 229.0 (9.5) g/d (total daily) Fat Least Square Mean (SEM): 58.8 (2.4) g/d (total daily)  Dietary Protein Intake Compliance (%): NR  Protein type/source: Mixed  Energy balance status: Eucaloric   Study duration: 18 months | **Intervention:** High Protein  **How protein was administered:** Participants received a dietary whey protein supplement (protein group; Provon 290; Glambia Nutritionals) that was closely matched for composition, color, kilocalories, sodium, potassium, phosphorus, fiber, and calcium. **Protein Assessment Method:** Participants completed a 3-day food record prior to baseline, 6 months, and 18 months and were analyzed using the ESHA Food Processor software program (ESHA Research; version 10.1.0).  **Dietary Protein Intake Compliance:** Urinary area was a compliance measure.   **Comparator:** Low Protein  **How protein was administered:**  Participants received a maltodextrin supplement Maltrin M100; Grain Processing Corp) that was closely matched for composition, color, kilocalories, sodium, potassium, phosphorus, fiber, and calcium.  **Protein Assessment Method:** Same as above **Dietary Protein Intake Compliance:** Same as above | **Muscle Mass -** Total lean body mass  Measure/Method of Assessment: DXA using either a Hologic 4500W machine (Yale University School of Medicine) or a Lunar Prodigy DPX-IQ (University of Connecticut Health Center) |
| PMID: 37739678 **Kruger 2023 (63)** Location/Country: New Zealand HDI: Very high Setting: Community dwelling  Urban/ Rural: NR Study design: RCT (parallel) Funding source: Industry  **Risk of bias score: High** | Study of: Adults Total sample N: 114  **Whole cohort** N: 103 % Female: 100% Mean Age (SD):70.1 (3.51) y Race/ Ethnicity:  89% New Zealand European Menopausal Status: NR Obesity Status: NR Mean BMI (SD): 24.8 (2.65) kg/m^2^ Income level: NR Education level:  Tertiary level of education: 34% Percent physical activity level:  Light: 38% Moderate: 55% Vigorous: 7% Health status/ Comorbidities: History of cancer: 18% Cardiovascular disease: 8% Hypertension: 24% Hypercholesterolemia: 30% Musculoskeletal conditions: 27%  Diabetes: 1% Gastrointestinal conditions: 16% Respiratory conditions: 8% Renal diseases: 1% Thyroid diseases: 6% Autoimmune diseases: 3% Psychological conditions: 16%  Medication use: Cholesterol medication: 14% Supplement use: NR Pregnant or lactating: NR | **Intervention 1:** Deer Milk  Intended Protein Amount: Protein supplement contained 15.0 g of protein per 200 ml Carbohydrate: Supplement contained 9.0 g of carbohydrate per 200 ml Fat: Supplement contained 19.6 g of fat per 200 ml  Baseline Protein Amount (whole cohort):  <1 g/kg: 29% 1-3 g/kg: 39% ≥1.3 g/kg: 32% Carbohydrate Mean (SD): NR Fat Mean (SD): NR  Actual Protein Amount at the end of the study  Mean (SD): NR Carbohydrate Mean (SD) NR Fat Mean (SD): NR  Dietary Protein Intake Compliance (%): 98.7%  Protein type/source: Animal  Energy balance status: Eucaloric   **Comparator:** Oral Nutritional Supplement  Intended Protein Amount: Protein supplement contained 12.0 g of protein per 200 ml Carbohydrate: Supplement contained 36.8 g of carbohydrate per 200 ml Fat: Supplement contained 11.6 g of fat per 200 ml  Baseline Protein Amount (whole cohort):  <1 g/kg: 29% 1-3 g/kg: 39% ≥1.3 g/kg: 32% Carbohydrate Mean (SD): NR Fat Mean (SD): NR  Actual Protein Amount at the end of the study  Mean (SD): NR Carbohydrate Mean (SD) NR Fat Mean (SD): NR Dietary Protein Intake Compliance (%): 96.8%  Protein type/source: Animal  Energy balance status: Eucaloric  Study duration: 11 weeks | **Intervention 1:** Deer Milk  **How protein was administered:** Participants consumed 200 ml of dear milk  **Protein Assessment Method:** Habitual dietary intake data was collected by 3-day estimated food diary at baseline. Average energy and macronutrient intake was assessed using FoodWorks Professional Edition 10.   **Dietary Protein Intake Compliance:** Throughout the study period, participants were also asked to complete a diary to record consumption of the beverages.  **Comparator:** Oral Nutritional Supplement  **How protein was administered:** Participants consumed 200 ml of oral nutritional supplement  **Protein Assessment Method:** Same as above   **Dietary Protein Intake Compliance:** Same as above | **Muscle Mass -** Fat-free mass  Measure/Method of Assessment: BIA (Inbody 320, Seoul, Korea)  **Muscle Mass** – Skeletal muscle mass  Measure/Method of Assessment: BIA (Inbody 320, Seoul, Korea)  **Muscle Strength** - Handgrip strength   Measure/Method of Assessment: Hand dynamometer  **Muscle Strength** - Chair stand test  Measure/Method of Assessment: Maximum number of chair stand repetitions possible in 30 s period was recorded  **Physical Performance** - 40 m fast-paced walk test  Measure/Method of Assessment: Fast-paced walking was timed over 4 x 10 m for a total of 40 m. |
| PMID: 33612439 **Li** **2021 (64)** Location/Country: China HDI: High Setting: Community dwelling Urban/ Rural: Urban Study design: RCT (parallel) Funding source: Nonprofit, government, academic **Risk of bias score: Low** | Study of: Adults Total sample  N: 123  **Intervention 1:** Whey Protein N: 31 % Female: 48.4% Mean Age (SD): 71 (4) y Race/ Ethnicity: NR Menopausal Status: NR Obesity Status: NR Mean BMI (SD): 21.8 (2.0) kg/m^2^ Income level: NR Education level:  High school or below: 67.7% College or above: 32.3% Mean physical activity level (SD): 38.8 (13.0) MET-h/d Health status/ Comorbidities: Low lean muscle mass; excluded disease with movement disorders such as stroke, fracture, and arthritis; previous osteoporotic fracture or joint replacement; musculoskeletal injuries; allergies to whey or soy protein supplements Medication use: NR Supplement use: Excluded those that used protein and antioxidant supplements within the past 12 months prior to enrollment Pregnant or lactating: NR  **Intervention 2:** Soy Protein N: 31 % Female: 51.6% Mean Age (SD): 69 (4) y Race/ Ethnicity: NR Menopausal Status: NR Obesity Status: NR Mean BMI (SD): 21.2 (2.3) kg/m^2^ Income level: NR Education level:  High school or below: 58% College or above: 42% Mean physical activity level (SD): 35.5 (15.7) MET-h/d Health status/ Comorbidities: Low lean muscle mass; excluded disease with movement disorders such as stroke, fracture, and arthritis; previous osteoporotic fracture or joint replacement; musculoskeletal injuries; allergies to whey or soy protein supplements Medication use: NR Supplement use: Excluded those that used protein and antioxidant supplements within the past 12 months prior to enrollment Pregnant or lactating: NR  **Intervention 3:** Whey-Soy protein group N: 31 % Female: 45.2% Mean Age (SD): 70 (4) y Race/ Ethnicity: NR Menopausal Status: NR Obesity Status: NR Mean BMI (SD): 20.6 (1.8) kg/m^2^ Income level: NR Education level:  High school or below: 54.8% College or above: 45.2% Mean physical activity level (SD): 38.9 (11.5) MET-h/d Health status/ Comorbidities: Low lean muscle mass; excluded disease with movement disorders such as stroke, fracture, and arthritis; previous osteoporotic fracture or joint replacement; musculoskeletal injuries; allergies to whey or soy protein supplements Medication use: NR Supplement use: Excluded those that used protein and antioxidant supplements within the past 12 months prior to enrollment Pregnant or lactating: NR  **Comparator:** Control N: 30 % Female: 56.7% Mean Age (SD): 71 (4) y Race/ Ethnicity: NR Menopausal Status: NR Obesity Status: NR Mean BMI (SD): 20.8 (2.2) kg/m^2^ Income level: NR Education level:  High school or below: 63.3% College or above: 36.7% Mean physical activity level (SD): 33 (14) MET-h/d Health status/ Comorbidities: Low lean muscle mass; excluded disease with movement disorders such as stroke, fracture, and arthritis; previous osteoporotic fracture or joint replacement; musculoskeletal injuries; allergies to whey or soy protein supplements Medication use: NR Supplement use: Excluded those that used protein and antioxidant supplements within the past 12 months prior to enrollment Pregnant or lactating: NR | **Intervention 1**: Whey Protein  Intended Protein Amount: Protein supplement contained 7.98 g of protein; dosage intended to increase participant protein consumption to 1.5 g/kg/d Carbohydrate: NR Fat: NR  Baseline Protein Amount Mean (SD): 62.7 (20.7) g/d; 1.14 (0.36) g/kg/d Carbohydrate Mean (SD): 200.9 (65.9) g/d Fat Mean (SD): 56.0 (21.2) g/d  Actual Protein Amount at the end of the study Mean (SD): 75.3 (13.8) g/d; 1.39 (0.24) g/kg/d (total) Carbohydrate Mean (SD): 185.5 (43.1) g/d Fat Mean (SD): 50.1 (11.7) g/d  Dietary Protein Intake Compliance (%): 99%  Protein type/source: Animal; whey  Energy balance status: Eucaloric  **Intervention 2:** Soy Protein  Intended Protein Amount: Protein supplement contained 8.80 g of protein; dosage intended to increase participant protein consumption to 1.5 g/kg/d Carbohydrate: NR Fat: NR  Baseline Protein Amount Mean (SD): 59.6 (19.1) g/d; 1.11 (0.33) g/kg/d Carbohydrate Mean (SD): 191.9 (56.5) g/d Fat Mean (SD): 52.8 (26.8) g/d  Actual Protein Amount at the end of the study  Mean (SD): 79.3 (20.5) g/d; 1.51 (0.41) g/kg/d (total) Carbohydrate Mean (SD): 195.7 (34.3) g/d Fat Mean (SD): 50.8 (16.8) g/d  Dietary Protein Intake Compliance (%): 91.5%  Protein type/source: Plant; soy  Energy balance status: Eucaloric  **Intervention 3**: Whey-Soy protein group  Intended Protein Amount: Protein supplement contained 8.39 g of protein; dosage intended to increase participant protein consumption to 1.5 g/kg/d Carbohydrate: NR Fat: NR  Baseline Protein Amount Mean (SD): 61.1 (19.1) g/d; 1.14 (0.37) g/kg/d Carbohydrate Mean (SD): 188.6 (50.9) g/d Fat Mean (SD): 51.7 (19.5) g/d  Actual Protein Amount at the end of the study Mean (SD): 80.2 (18.2) g/d; 1.49 (0.34) g/kg/d (total) Carbohydrate Mean (SD): 197.5 (51.8) g/d Fat Mean (SD): 51.1 (16.9) g/d  Dietary Protein Intake Compliance (%): 94.5%  Protein type/source: Mixed; whey and soy (1:1 ratio)  Energy balance status: Eucaloric  **Comparator:** Control  Intended Protein Amount: Followed habitual diet; total daily protein goal NR Carbohydrate: NR Fat: NR  Baseline Protein Amount Mean (SD): 59.3 (18.8) g/d; 1.17 (0.30) g/kg/d Carbohydrate Mean (SD): 221 (45.4) g/d Fat Mean (SD): 49.0 (17.3) g/d  Actual Protein Amount at the end of the study  Mean (SD): 56.3 (11.0) g/d; 1.11 (0.25) g/kg/d (total) Carbohydrate Mean (SD): 212.0 (88.1) g/d Fat Mean (SD): 49.0 (11.3) g/d  Dietary Protein Intake Compliance (%): NA  Protein type/source: Mixed  Energy balance status: Eucaloric  Study duration: 6 months | **Intervention 1:** Whey Protein  **How protein was administered:** Participants consumed a whey protein supplement twice daily  **Protein Assessment Method:** Measured at baseline and at 6 months using a 79-item semi quantitative FFQ. Baseline FFQ was used to collect dietary intake in the past year. FFQ at 6 months was used to collect the dietary intake during the 6-month intervention. Daily dietary intakes calculated based on the China Food Composition Table 2004.  **Dietary Protein Intake Compliance:** For Whey Protein, Soy Protein, and Whey- Soy protein groups compliance was assessed by counting the number of protein packets returned by the participants.  **Intervention 2**: Soy Protein  **How protein was administered:** Participants consumed a soy protein supplement twice daily  **Protein Assessment Method:** Same as above  **Dietary Protein Intake Compliance:** Same as above  **Intervention 3:** Whey-soy protein group  **How protein was administered:** Participants consumed a whey-soy supplement (1:1 ratio) twice daily  **Protein Assessment Method:** Same as above  **Dietary Protein Intake Compliance:** Same as above  **Comparator:** Control  **How protein was administered:** Participants consumed habitual diet  **Protein Assessment Method:** Same as above  **Dietary Protein Intake Compliance:** Same as above | **Muscle Mass -** Total body lean mass  Measure/Method of Assessment: DXA (Discovery W; Hologic Inc)  **Muscle Mass -** Appendicular lean mass/ skeletal muscle mass   Measure/Method of Assessment: DXA (Discovery W; Hologic Inc)  **Muscle Mass –** Appendicular skeletal muscle index  Measure/Method of Assessment: Appendicular lean mass divided by height squared   **Muscle Strength -** Handgrip strength  Measure/Method of Assessment: Measured using handgrip dynamometer  **Physical Performance** **-** 4 m gait speed  Measure/Method of Assessment: Walk 8m at usual pace and time used for walking through the central 4 m was measured  **Muscle Strength -** Chair stand test  Measure/Method of Assessment: Participants to stand up from a chair and sit down 5 times as quickly as possible with arms folded across their chests.  **Physical Performance -**SPPB  Measure/Method of Assessment: The SPPB consists of three components: balance, gait speed and chair rise ability |
| PMID: 33871558 **Murphy** **2021^1^ (40)** Location/Country: Ireland HDI: Very high Setting: Community dwelling Urban/ Rural: Urban  Study design: RCT (parallel) Funding source: Government **Risk of bias score: High** | Study of: Adults Total sample N: 107  **Intervention 1:** Leucine-enriched Protein N: 38 % Female: 52.6% Mean Age (SD): 70 (5) y Race/Ethnicity: 100% White Menopausal status: NR Obesity status: NR Mean BMI (SD): 24.8 (3.4) kg/m^2^ Income level: NR Education level: NR Mean physical activity level (SD): 8354 (4125) steps/day  Health status/ Comorbidities:  Included: Low skeletal muscle mass; generally healthy according to responses to a standard health screening questionnaire Excluded: malignancy in the past 5 years, diabetes, advanced renal disease, neuromuscular disease, total walking incapacity Medication use: Mean (SD) number of mediations: 1 (2); Excluded if taking medications that interfere with the nutrition intervention - corticosteroids for systemic use, hormone replacement therapy, insulin, high-dose anti-inflammatories, simvastatin Supplement use: Excluded if consumed LC n-3 PUFA supplementation and were not willing to cease consumption ≥ 6 weeks prior to and for the duration of the 24-wk study Pregnant or lactating: NR  **Intervention 2**: Leucine-enriched Protein+ PUFAs N: 38 % Female: 55.3% Mean Age (SD): 73 (6) y Race/Ethnicity: 100% White Menopausal status: NR Obesity status: NR Mean BMI (SD): 26.7 (3.2) kg/m^2^  Income level: NR Education level: NR Mean physical activity level (SD): 8257 (3906) steps/day Health status/ Comorbidities:  Included: Low skeletal muscle mass; generally healthy according to responses to a standard health screening questionnaire Excluded: malignancy in the past 5 years, diabetes, advanced renal disease, neuromuscular disease, total walking incapacity Medication use: Mean (SD) number of medications: 2 (2); Excluded if taking medications that interfere with the nutrition intervention - corticosteroids for systemic use, hormone replacement therapy, insulin, high-dose anti-inflammatories, simvastatin Supplement use: Excluded if consumed LC n-3 PUFA supplementation and were not willing to cease consumption ≥ 6 weeks prior to and for the duration of the 24-wk study Pregnant or lactating: NR  **Comparator:** Normal Protein N: 31 % Female: 45.2% Mean Age (SD): 73 (7) y Race/Ethnicity: 100% White Menopausal status: NR Obesity status: NR Mean BMI (SD): 25.4 (2.8) kg/m^2^ Income level: NR Education level: NR Mean physical activity level (SD): 8192 (5142) steps/day Health status/ Comorbidities:  Included: Low skeletal muscle mass; generally healthy according to responses to a standard health screening questionnaire Excluded: malignancy in the past 5 years, diabetes, advanced renal disease, neuromuscular disease, total walking incapacity Medication use: Mean (SD) number of medications: 2 (3); Excluded if taking medications that interfere with the nutrition intervention - corticosteroids for systemic use, hormone replacement therapy, insulin, high-dose anti-inflammatories, simvastatin Supplement use: Excluded if consumed LC n-3 PUFA supplementation and were not willing to cease consumption ≥ 6 weeks prior to and for the duration of the 24-wk study Pregnant or lactating: NR | **Intervention 1:** Leucine-enriched Protein  Intended Protein Amount: 21.2 g in supplemental protein per day; total intake goals NR Carbohydrate: NR Fat: NR  Baseline Protein Amount Mean (SD): 84 (26) g/d; 17.1 (3.9) % of energy Carbohydrate Mean (SD): 226 (78) g/d; 45.0 (9.7) % of energy Fat Mean (SD): 82 (32) g/d; 36.3 (7.7) % of energy  Actual Protein Amount at the end of the study Mean (SD): 100 (23) g/d; 19.6 (3.3) % of energy Carbohydrate Mean (SD): 229 (60) g/d; 44.6 (6.7) % of energy Fat Mean (SD): 80 (24) g/d; 34.8 (6.3) % of energy  Dietary Protein Intake Compliance (%): Median (IQR): 89% (83-94%)  Protein type/source: Whey protein and a peptide carrier enriched with free leucine  Energy balance status: Eucaloric  **Intervention 2:** Leucine-enriched Protein +PUFAS  Intended Protein Amount: 21.2 g in supplemental protein per day; total intake goals NR Carbohydrate: NR Fat: NR  Baseline Protein Amount Mean (SD): 77 (25) g/d; 17.6 (4.5) % of energy Carbohydrate Mean (SD): 200 (66) g/d; 45.6 (8.4) % of energy Fat Mean (SD): 69 (25) g/d; 35.4 (8.8) % of energy  Actual Protein Amount at the end of the study Mean (SD): 92 (25) g/d; 19.9 (4.0) % of energy Carbohydrate Mean (SD): 200 (57) g/d; 43.5 (8.0) % of energy Fat Mean (SD): 76 (28) g/d; 36.2 (7.8) % of energy  Dietary Protein Intake Compliance (%): Median (IQR): 92% (87-97%)  Protein type/source: Animal; whey protein and a peptide carrier enriched with free leucine   Energy balance status: Eucaloric  **Comparator:** Normal Protein  Intended Protein Amount: NR Carbohydrate: NR Fat: NR  Baseline Protein Amount Mean (SD): 79 (34) g/d; 16.7 (5.3) % of energy Carbohydrate Mean (SD): 214 (62) g/d; 45.6 (7.5) % of energy Fat Mean (SD): 80 (34) g/d; 37.4 (9.3) % of energy  Actual Protein Amount at the end of the study Mean (SD): 83 (23) g/d; 15.2 (3.2) % of energy Carbohydrate Mean (SD): 268 (68) g/d; 49.8 (5.6) % of energy Fat Mean (SD): 85 (30) g/d; 34.8 (5.4) % of energy  Dietary Protein Intake Compliance (%): Median (IQR): 93% (87-95%)  Protein type/source: Mixed  Energy balance status: Eucaloric  Study duration: 24 weeks | **Intervention 1:** Leucine-enriched Protein  **How protein was administered:** Two supplements daily equaling 21.2 g protein per day (including 6.2 g leucine); one was consumed before breakfast and one before their second light meal of the day with habitual diet  **Protein Assessment Method:** Dietary intake was assessed via a 24-h recall using the 5-step multiple-pass method at pre-, mid-, and post intervention visits  **Dietary Protein Intake Compliance:** Compliance was derived using the self-report supplement logs  **Intervention 2:** Normal Protein  **How protein was administered:** Two supplements daily equaling 21.2 g protein per day (including 6.2 g leucine and 4 g LC n-3 PUFAs); one was consumed before breakfast and one before their second light meal of the day with habitual diet  **Protein Assessment Method:** Same as above  **Dietary Protein Intake Compliance:** Same as above  **Comparator:** Normal Protein  **How protein was administered:** Isocaloric maltodextrin supplement  **Protein Assessment Method:** Same as above  **Dietary Protein Intake Compliance:** Same as above | **Muscle Mass -** Adjusted appendicular lean mass/ skeletal Muscle Mass  Measure/Method of Assessment: DXA (GE-LUNAR iDXA; Aymes Medical)  **Muscle Strength -** Handgrip strength  Measure/Method of Assessment: Measured using handgrip dynamometer   **Muscle Strength -** Isometric knee extension peak torque  Measure/Method of Assessment: Self-reported dominant leg using a dynamometer; warm-up and 4 maximal leg extensions at 90 degrees with 60s between (also did 3 rounds at 60 degrees and 120 degrees); highest result used in analysis  **Muscle Strength -** Isometric knee flexion peak torque  Measure/Method of Assessment: Self-reported dominant leg using a dynamometer; warm-up and 4 maximal leg flexion at 90 degrees with 60s between; highest result used in analysis  **Physical Performance -**SPPB  Measure/Method of Assessment: The SPPB consists of three components: balance, gait speed and chair rise ability   **Physical Performance -** Gait speed  Measure/Method of Assessment: Per standard SPPB protocols  **Physical Performance -** TUG  Measure/Method of Assessment: Per standard protocols; repeated twice with the average of the tests used in analysis  **Muscle Strength-** 5 times sit-to-stand  Method/Measure of Assessment: Per standard SPPB protocols |
| PMID: 34098214 **Peng**  **2021^1^ (41)** Location/Country: Taiwan/China HDI: High Setting: Community dwelling  Urban/ Rural: NR Study design: RCT (parallel) Funding source: Academic, industry **Risk of bias score: High** | Study of: Adults Total sample N: 52  **Intervention:** High Protein N: 27 % Female: 48.1% Mean Age (SD): 53.4 (8.1) y Race/ Ethnicity: NR Menopausal status: NR Obesity status: NR Mean BMI (SD): 25.1 (3.9) kg/m^2^ Income level: NR Mean education level (SD): 14.1 (2.9) y Mean physical activity level (SD): 1567.3 (1244.9) kcal/wk Health status/ Comorbidities: Excluded: (1) history of fracture or severe arthritis in recent 6 months, (2) known history of chronic kidney disease stage III and over, i.e. estimated glomerular filtered rate (eGFR) < 60 ml/min/1.73 m^2^, (3) contraindicated for magnetic resonance imaging, (4) using anabolic hormones in the past 3 months, (5) were disability or limited functional ability, (6) having advanced, active or uncontrolled diseases, and (6) dementia, cognitive impairment or other sensory impairment that limited communication and understanding of the study  Medication use: Excluded those using anabolic hormones Supplement use: NR Pregnant or lactating: NR  **Comparator:** Normal Protein N: 25 % Female: 44% Mean Age (SD): 54 (8.6) y Race/Ethnicity: NR Menopausal status: NR Obesity status: NR Mean BMI (SD): 25.6 (3.8) kg/m^2^  Income level: NR Mean education level (SD): 15.5 (2.7) y Mean physical activity level (SD): 1954.0 (1646.4) kcal/wk Health status/ Comorbidities: Excluded: (1) history of fracture or severe arthritis in recent 6 months, (2) known history of chronic kidney disease stage III and over, i.e. estimated glomerular filtered rate (eGFR) < 60 ml/min/1.73 m^2^, (3) contraindicated for magnetic resonance imaging, (4) using anabolic hormones in the past 3 months, (5) were disability or limited functional ability, (6) having advanced, active or uncontrolled diseases, and (6) dementia, cognitive impairment or other sensory impairment that limited communication and understanding of the study  Medication use: Excluded those using anabolic hormones Supplement use: NR Pregnant or lactating: NR | **Intervention:** High Protein  Intended Protein Amount: 25% of energy Carbohydrate: NR Fat: NR  Baseline Protein Amount Mean (SD): NR Carbohydrate Mean (SD): NR Fat Mean (SD): NR  Actual Protein Amount at the end of the study Mean (SD): NR Carbohydrate Mean (SD): NR Fat Mean (SD): NR  Dietary Protein Intake Compliance (%): 91.2%  Protein type/source: Mixed  Energy balance status: Eucaloric  **Comparator:** Normal Protein  Intended Protein Amount: 15% of energy Carbohydrate: NR Fat: NR  Baseline Protein Amount Mean (SD): NR Carbohydrate Mean (SD): NR Fat Mean (SD): NR  Actual Protein Amount at the end of the study Mean (SD): NR Carbohydrate Mean (SD): NR Fat Mean (SD): NR  Dietary Protein Intake Compliance (%): 79.5%  Protein type/source: Mixed  Energy balance status: Eualoric  Study duration: 12 weeks | **Intervention:** High Protein  **How protein was administered:** Received 10 frozen meals per week for 12 weeks containing 25% energy in protein.  **Protein Assessment Method:** NR  **Dietary Protein Intake Compliance:** Insufficient compliance to the study protocol (e.g low meal complete rate and vigorous changes of lifestyle)  **Comparator:** Normal Protein  **How protein was administered:** Received 10 frozen meals per week for 12 weeks containing 15% energy in protein.  **Protein Assessment Method:**  Same as above  **Dietary Protein Intake Compliance:** Same as above | **Muscle Strength -** Handgrip strength  Measure/Method of Assessment: Measured using handgrip dynamometer  **Muscle Strength-** 5-time chair rise test  Measure/Method of Assessment: NR  **Physical Performance -** 6 min walking distance  Measure/Method of Assessment: NR  **Physical Performance -** 6 meter walking speed  Measure/Method of Assessment: Usual pace  **Muscle Mass -** Lean body mass  Measure/Method of Assessment: BIA (Inbody S10, Biospace device, USA)  **Muscle Mass -** Relative Appendicular Skeletal Muscle mass   Measure/Method: Appendicular muscle mass divided by squared height in meters. |
| PMID: 34609621 **Reinders** **2022 (65)** Location/Country: Finland, Netherlands HDI: Very High Setting: Community Dwelling, Urban and Rural Study Design: RCT (Parallel) Funding Source: Government **Risk of bias score: Low** | Study of: Adults Total sample N: 187  **Intervention**: Protein advice N: 96 % Female: 52.1% Mean Age (SD): 75.9 (5.0) y Race/ Ethnicity: NR Menopausal Status: NR Obesity Status: NR Mean BMI (SD): 26.3 (2.9) kg/m^2^ Income level: NR Education level:  Lower education: 5.2% Middle education: 18.8% Higher education: 76% Physical activity level: NR Health status/ Comorbidities: Self-perceived health:  Very poor/poor: 0% Not poor/not good: 19.8% Good/ very good: 80.2% Medication use: NR Supplement use: NR Pregnant or lactating: NR  **Comparator:** Control N: 91 % Female: 54.9% Mean Age (SD): 75.0 (4.4) y Race/ Ethnicity: NR Menopausal Status: NR Obesity Status: NR Mean BMI (SD): 26.9 (2.9) kg/m^2^ Income level: NR Education level:  Lower education: 5.5% Middle education: 24.2% Higher education: 70.3% Physical activity level: NR Health status/ Comorbidities: Self-perceived health:  Very poor/poor: 0% Not poor/not good: 19.8% Good/ very good: 80.3% Medication use: NR Supplement use: NR Pregnant or lactating: NR | **Intervention:** Protein advice  Intended Protein Amount: ≥1.2 g/kg aBW/d Carbohydrate: NR Fat: NR  Baseline Protein Amount Mean (SD): 60.4 (1.3) g/d; 0.82 (0.01) g/kg aBW/d Carbohydrate Mean (SD): NR Fat Mean (SD): NR  Actual Protein Amount at the end of the study Mean (SD): 89.1 (2.3) g/d; 1.21 (0.03) g/kg aBW/d Carbohydrate Mean (SD): NR Fat Mean (SD): NR  Dietary Protein Intake Compliance (%): <0.8 g/kg aBW/d – 4.4% 0.8–1.0 g/kg aBW/d –14.4% 1.0–1.2 g/kg aBW/d – 32.2% ≥1.2 g/kg aBW/d – 48.9%  Protein type/source: Mixed  Energy balance status: Eucaloric  **Comparator:** Control  Intended Protein Amount: NR Carbohydrate: NR Fat: NR  Baseline Protein Amount Mean (SD): 60.5 (1.2) g/d; 0.82 (0.01) g/kg aBW/d Carbohydrate Mean (SD): NR Fat Mean (SD): NR  Actual Protein Amount at the end of the study Mean (SD): 63.7 g/d; 0.86 (0.02) g/kg aBW/d Carbohydrate Mean (SD): NR Fat Mean (SD): NR  Dietary Protein Intake Compliance (%): <0.8 g/kg aBW/d – 40.5% 0.8–1.0 g/kg aBW/d – 36.9% 1.0–1.2 g/kg aBW/d – 15.5% ≥1.2 g/kg aBW/d – 7.1%  Protein type/source: Mixed  Energy balance status: Eucaloric  Study duration: 6 months | **Intervention:** Protein advice  **How protein was administered:** Participants received personalized dietary advice by nutritionist to increase protein intake to ≥ 1.2 g/kg aBW/d using regular protein-rich foods purchased by the respondents and protein enriched food products freely provided by the research team  **Protein Assessment Method:** Assessed prior to each clinic visit through a full dietary assessment using food diaries on three days, followed by a 24h dietary recall to assess habitual protein intake. Protein intake assessed at 3 months and 6 months.   **Dietary Protein Intake Compliance:** Compliance of study participants to adhere to the advice to increase protein intake was indicated by the percentage of participants reaching a certain protein intake (<0.8 g/kg aBW/d, 0.8–1.0 g/kg aBW/d, 1.0–1.2 g/kg aBW/d or≥1.2 g/kg aBW/d) for each study group at each clinic visit.  **Comparator:** Control  **How protein was administered:** Did not receive any protein advice or protein enriched foods  **Protein Assessment Method:** Same as above  **Dietary Protein Intake Compliance:** Same as above | **Physical Performance -** 400 m walk speed  Measure/Method of Assessment: After 40-m warmup, participants were instructed to walk as fast as possible at a pace they could maintain for 400 m  **Physical Performance -**SPPB  Measure/Method of Assessment: The SPPB consists of three components: balance, gait speed and chair rise ability  **Muscle Strength -** Handgrip strength  Measure/Method of Assessment: Measured using handgrip dynamometer  **Muscle Strength -** Leg extension strength  Measure/Method of Assessment: NR  **Muscle Mass -** Fat Free Mass  Measure/Method of Assessment: BIA (BodyStat 1500MDD, Bodystat Ltd, Douglas, Isle of Men, United Kingdom) |
| PMID: 29687650 **Smith** **2018 (66)** Location/Country: USA HDI: Very high Setting: Community dwelling Urban/Rural: NR Study design: RCT (parallel) Funding source: Nonprofit, government **Risk of bias score: Low** | Study of: Adults Total sample N: 52  **Intervention:** Weight loss plus whey protein N: 25 % Female: 100% Mean Age (SD): NR Race/ Ethnicity: NR Menopausal Status: Postmenopausal Obesity Status: 100% Obese Mean BMI (SD): NR Income level: NR Education level: NR Physical activity level: Excluded if engaged in ≥ 1.5 hours of exercise/week Health status/ Comorbidities: Excluded if they had serious chronic disease (e.g. neuromuscular, cardiopulmonary, chronic kidney disease, diabetes, cancer) or a condition that could interfere with body composition imaging (e.g., certain metal implants) Medication use: Excluded those that were taking medications that could affect muscle mass and/or function (e.g., HMG-CoA reductase inhibitors, steroids) within 1 year before enrolling in the study. Supplement use: NR Pregnant or lactating: NR  **Comparator**: Weight loss plus recommended protein N: 27 % Female: 100% Mean Age (SD): NR Race/ Ethnicity: NR Menopausal Status: Postmenopausal Obesity Status: 100% Obese Mean BMI (SD): NR Income level: NR Education level: NR Physical activity level: Excluded if engaged in ≥ 1.5 hours of exercise/week Health status/ Comorbidities: Excluded if they had serious chronic disease (e.g. neuromuscular, cardiopulmonary, chronic kidney disease, diabetes, cancer) or a condition that could interfere with body composition imaging (e.g., certain metal implants) Medication use: Excluded those that were taking medications that could affect muscle mass and/or function (e.g., HMG-CoA reductase inhibitors, steroids) within 1 year before enrolling in the study. Supplement use: NR Pregnant or lactating: NR | **Intervention:** Weight loss plus whey protein  Intended Protein Amount: 1.2 g/kg/d Carbohydrate: NR Fat: NR  Baseline Protein Amount Mean (SD): NR Carbohydrate Mean (SD): NR Fat Mean (SD): NR  Actual Protein Amount at the end of the study Mean (SD): 31 (1) % of energy; 105 (2) g/d; 1.22 (0.03) g/kg/d Carbohydrate Mean (SD): 44 (1) % of energy Fat Mean (SD): 24 (1) % of energy  Dietary Protein Intake Compliance (%): NR  Protein type/source: Mixed  Energy balance status: Hypocaloric  **Comparator:** Weight loss plus recommended protein  Intended Protein Amount: 0.8 g/kg/d Carbohydrate: NR Fat: NR  Baseline Protein Amount  Mean (SD): NR Carbohydrate Mean (SD): NR Fat Mean (SD): NR  Actual Protein Amount at the end of the study Mean (SD): 22 (1) % of energy; 74 (3) g/d; 0.86 (0.03) g/kg/d Carbohydrate Mean (SD): 50 (1) % of energy Fat Mean (SD): 28 (1) % of energy  Dietary Protein Intake Compliance (%): NR  Protein type/source: Mixed  Energy balance status: Hypocaloric  Study duration: About 6 months (when participant lost 10% of body weight) | **Intervention**: Weight loss plus whey protein  **How protein was administered:** Two nutrition bars per day for breakfast and frozen entrees for lunch and dinner were provided to the participants. Individuals also received two servings of whey protein isolate per day with breakfast and as a midafternoon snack.  **Protein Assessment Method:** Dietary intake was monitored by reviewing subjects’ daily diet records during weekly visits with the study dietician.  **Dietary Protein Intake Compliance:** Dietary compliance: (1) all meals and the protein supplement were provided to the study subjects, (2) dietary intake was monitored by reviewing subjects’ daily diet records during weekly visits with the study dietician, and (3) blood urea nitrogen and, in a subset of participants, urinary urea nitrogen excretion were measured as objective markers of protein intake.  **Comparator:** Weight loss plus recommended protein N: 27  **How protein was administered:** Two nutrition bars per day for breakfast and frozen entrees for lunch and dinner were provided to the participants. Individuals also received isocaloric foods compared to the whey protein isolate in the increased protein group that provided mostly carbohydrates and fat per day with breakfast and as a midafternoon snack.  **Protein Assessment Method:** Same as above  **Dietary Protein Intake Compliance:** Same as above | **Muscle Mass -** Total fat-free mass  Measure/Method of Assessment: DXA (Lunar iDA; GE Healthcare Lunar; Madison, Wisconsin)  **Muscle Mass -** Total body lean mass  Measure/Method of Assessment: DXA (Lunar iDA; GE Healthcare Lunar; Madison, Wisconsin)  **Muscle Strength -** Sum 1-RM strength  Measure/Method of Assessment: The maximal amount of weight each participant was able to lift just once, evaluated with a Hoist multi-station weight machine  **Muscle Strength -** Sum knee extension peak torque  Measure/Method of Assessment: Peak isometric and isokinetic (608/s & 1808/s) torque of the knee extensors and flexors of the dominant leg were evaluated using Biodex 3 dynamometer. Exercise repeated 3x, the mean of the 2 highest torque recordings for each exercise used in analysis.  **Muscle Strength** - Sum knee flexion peak torque  Measure/Method of Assessment: Peak isometric and isokinetic (608/s & 1808/s) torque of the knee extensors and flexors of the dominant leg were evaluated using Biodex 3 dynamometer. Exercise repeated 3x, the mean of the 2 highest torque recordings for each exercise used in analysis. |
| PMID: 28492492 **Stojkovic** **2017 (67)** Location/Country: USA HDI: Very high Setting: Community dwelling Urban/Rural: NR Study design: Ancillary study of an RCT (parallel) Funding Source: Academic, government **Risk of bias score: High** | Study of: Adults Total sample N: 84  **Intervention:** Protein Group N: 38 % Female: 100% Mean Age (SD): 68.9 (0.9) y Race/ Ethnicity: NR Menopausal Status: Postmenopausal Obesity Status: NR Mean BMI (SD): 26 (0.6) kg/m^2^ Income level: NR  Education level: NR Mean physical activity level (SD): NR Health status/ Comorbidities: NR Medication use: NR Supplement use: NR Pregnant or lactating: NR  **Comparator**: Carbohydrate Group N: 46 % Female: 100% Mean Age (SD): 69.3 (0.09) y Race/ Ethnicity: NR Menopausal Status: Postmenopausal Obesity Status: NR Mean BMI (SD): 25.8 (0.6) kg/m^2^ Income level: NR Education level: NR Mean physical activity level (SD): NR Health status/ Comorbidities: NR  Medication use: NR  Supplement use: NR Pregnant or lactating: NR | **Intervention**: Protein Group  Intended Protein Amount: NR Carbohydrate: NR Fat: NR  Baseline Protein Amount Mean (SD): 73.5 (2.7) g/d Carbohydrate Mean (SD): 207.2 (9.0) g/d Fat Mean (SD): 56.1 (2.7) g/d  Actual Protein Amount at the end of the study Mean (SD): 98.5 (2.8) g/d (total protein) Carbohydrate Mean (SD): 198.9 (8.9) g/d Fat Mean (SD): 51.6 (2.5) g/d  Dietary Protein Intake Compliance (%): NR  Protein type/source: Animal; whey protein  Energy balance status: Eucaloric  **Comparator:** Carbohydrate Group  Intended Protein Amount: NR Carbohydrate: NR Fat: NR  Baseline Protein Amount Mean (SD): 71.5 (2.2) g/d Carbohydrate Mean (SD): 201.2 (6.9) g/d Fat Mean (SD): 62.5 (3.9) g/d  Actual Protein Amount at the end of the study Mean (SD): 69.8 (2.5) g/d Carbohydrate Mean (SD): 232.3 (8.7) g/d (total) Fat Mean (SD): 57.1 (2.8) g/d  Dietary Protein Intake Compliance (%): NR  Protein type/source: Mixed  Energy balance status: Eucaloric  Study duration: 18 months | **Intervention:** Protein Group  **How protein was administered:** Subjects consumed a minimum of 20 g of protein supplement for 18 months  **Protein Assessment Method:** Participants completed a 3-day food record prior to each study visit. Food records were analyzed using the ESHA Food Processor software program (ESHA Research, Salem, OR, USA, version 10.1.0)  **Dietary Protein Intake Compliance:** Supplement adherence and diet were carefully monitored by dietitians.  **Comparator:** Carbohydrate Group  **How protein was administered:** Received an isocaloric maltodextrin control supplement  **Protein Assessment Method:** Same as above  **Dietary Protein Intake Compliance:** Same as above | **Muscle Mass -** Body lean mass  Measure/Method of Assessment: DXA, using either a Hologic 4500 W machine (Yale University School of Medicine) or a Lunar Prodigy DPX-IQ (University of Connecticut Health Center) |
| PMID: 22406907 **Wycherley** **2012^1^ (42)** Location/Country: Australia HDI: Very high Setting: Community dwelling Urban/Rural: NR Study design: RCT (parallel) Funding source: Industry **Risk of bias score: Moderate** | Study of: Adults Total sample N: 68  **Intervention:** High Protein N: 33 % Female: 0% Mean Age (SD): 51.3 (9.4) y Race/Ethnicity: NR  Menopausal status: NA  Obesity status: 100% overweight or obese Mean BMI (SD): 33.0 (3.9) kg/m^2^ (total study population mean) Income level: NR Education level: NR Physical activity level: NR Health status/ Comorbidities: Excluded: diabetes, uncontrolled hypertension; history of GI, renal, coronary, metabolic, or hepatic disease or malignancy Medication use: Excluded those taking hypoglycemic medication or drugs which affect insulin sensitivity Supplement use: NR Pregnant or lactating: NA  **Comparator:** Low Protein N: 35 % Female: 0% Mean Age (SD): 50.2 (9.3) y Race/Ethnicity: NR Menopausal status: NA Obesity status: 100% overweight or obese Mean BMI (SD): 33.0 (3.9) kg/m^2^ (total study population mean)  Income level: NR Education level: NR Physical activity level: NR Health status/ Comorbidities: Excluded: diabetes, uncontrolled hypertension; history of GI, renal, coronary, metabolic, or hepatic disease or malignancy Medication use: Excluded those taking hypoglycemic medication or drugs which affect insulin sensitivity Supplement use: NR Pregnant or lactating: NA | **Intervention:** High Protein  Intended Protein Amount: 35% of energy; 142 g/d; ~1.30 g/kg/d Carbohydrate: 40% of energy; 135 g/d Fat: 25% of energy (total 53 g/d, saturated 14 g/d)  Baseline Protein Amount Mean (SD): NR Carbohydrate Mean (SD): NR Fat Mean (SD): NR  Actual Protein Amount at the end of the study: Mean (SD): 0-12 weeks: 131.1 (15.4) g/d; 32.5 (3.3) % of energy 12-52 weeks: 132 (13.9) g/d; 30.7 (3.1) % of energy Carbohydrate Mean (SD):  0-12 weeks: 154.4 (31.8) g/d; 37.4 (3.8) % of energy 12-52 weeks: 157.9 (28.1) g/d; 35.9 (3.4) % of energy Fat Mean (SD):  0-12 weeks: 50.6 (6.5) g/d; 27.3 (3.0) % of energy 12-52 weeks: 60.0 (12.6) g/d; 29.8 (3.6) % of energy  Dietary Protein Intake Compliance (%): NR – good compliance rate stated  Protein type/source: Mixed  Energy balance status: Hypocaloric  **Comparator:** Low Protein  Intended Protein Amount: 17% of energy; 88 g/d; ~0.85 g/kg/d Carbohydrate: 58% of energy; 198 g/d Fat: 25% of energy (total 51 g/d, saturated 14 g/d)  Baseline Protein Amount Mean (SD): NR Carbohydrate Mean (SD): NR Fat Mean (SD): NR  Actual Protein Amount at the end of the study Mean (SD):  0-12 weeks: 82.7 (6.7) g/d; 20.5 (1.4) % of energy 12-52 weeks: 83.3 (10.3) g/d; 20.4 (1.0) % of energy Carbohydrate Mean (SD):  0-12 weeks: 208.4 (16.3) g/d; 51.0 (3.6) % of energy 12-52 weeks: 195.2 (23.4) g/d; 47.3 (3.9) % of energy Fat Mean (SD): 0-12 weeks: 46.7 (7.5) g/d; 25.0 (3.3) % of energy 12-52 weeks: 52.2 (8.7) g/d; 27.7 (3.2) % of energy  Dietary Protein Intake Compliance (%): NR – good compliance rate stated  Protein type/source: Mixed  Energy balance status: Hypocaloric  Study duration: 52 weeks | **Intervention:** High Protein  **How protein was administered:** Participants met with dietitian and received detailed dietary prescription, meal planning advice, and recipe information every 2 weeks for the first 12 weeks. They were supplied with a 2-week provision of diet-specific key foods (60% of energy intake) for the first 12 weeks. Participants met with dietician monthly and received detailed dietary prescription, meal planning advice, and recipe information for remainder of study duration.  **Protein Assessment Method:** Participants kept a daily semi-quantitative food record. Dietary intake was assessed using a computerized database (Foodworks Professional Edition, version 4, 1998; Xyris Software, Highgate Hill, Australia) based on the analysis of 3 non-consecutive days (1 weekend day and 2 weekdays) of each 2-week period. The intake was calculated as an average of the 2-week diet record data blocks for 0-12 weeks and 12-52 weeks.   **Dietary Protein Intake Compliance:** Food checklist  **Comparator:** Low Protein  **How protein was administered:** Participants met with dietitian and received detailed dietary prescription, meal planning advice, and recipe information every 2 weeks for the first 12 weeks. They were supplied with a 2-week provision of diet-specific key foods (60% of energy intake) for the first 12 weeks. Participants met with dietician monthly and received detailed dietary prescription, meal planning advice, and recipe information for remainder of study duration.  **Protein Assessment Method:** Same as above  **Dietary Protein Intake Compliance:** Same as above | **Muscle Mass -** Total body fat free mass  Measure/Method of Assessment: DXA (Lunar Prodigy; General Electric, Madison, WI, USA) |
| PMID: 26400966 **Zhu** **2015 (68)** Location/Country: Australia HDI: Very high Setting: Community dwelling Urban/Rural: Metropolitan Study design: RCT (parallel) Funding source: Academic, government **Risk of bias score: Low** | Study of: Adults Total sample N: 196  **Intervention**: High Protein N: 101 % Female: 100% Mean Age (SD): 74.2 (2.8) y Race/ Ethnicity: NR Menopausal Status: Postmenopausal Obesity Status: NR Mean BMI (SD): 26.1 (3.8) kg/m^2^ Income level: NR Education level: NR Mean physical activity level (SD): 453 (390) MET -min/wk Health status/ Comorbidities: Excluded those with a previous osteoporotic fracture or metabolic bone disease, or any other condition that may affect the participation of the study Medication use: Excluded those taking medication for osteoporosis (including hormone replacement therapy) apart from calcium or vitamin D either currently or within the last year, or were taking steroid tablets in the previous 3 months or had taken >7 g in total in their lifetime Supplement use: Excluded those with a high protein intake (>1.5 g/kg/d) Pregnant or lactating: NR  **Comparator:** Placebo supplement N: 95 % Female: 100% Mean Age (SD): 74.3 (2.6) y Race/ Ethnicity: NR Menopausal Status: Postmenopausal Obesity Status: NR Mean BMI (SD): 27.2 (4.0) kg/m^2^ Income level: NR Education level: NR Mean physical activity level (SD): 398 (376) MET- min/wk Health status/ Comorbidities: Excluded those with a previous osteoporotic fracture or metabolic bone disease, or any other condition that may affect the participation of the study Medication use: Excluded those taking medication for osteoporosis (including hormone replacement therapy) apart from calcium or vitamin D either currently or within the last year, or were taking steroid tablets in the previous 3 months or had taken >7 g in total in their lifetime Supplement use: Excluded those with a high protein intake (>1.5 g/kg/d) Pregnant or lactating: NR | **Intervention:** High Protein  Intended Protein Amount: Supplement with 30 g of protein Carbohydrate: NR Fat: NR  Baseline Protein Amount Mean (SD): 76 (18) g/d; 1.2 (0.3) g/kg/d Carbohydrate Mean (SD): 190 (45) g/d Fat Mean (SD): 63 (19) g/d  Actual Protein Amount at the end of the study Mean (SD): 95.9 (19.9) g/d Carbohydrate Mean (SD): NR Fat Mean (SD): NR  Dietary Protein Intake Compliance (%): 87.1%  Protein type/source: Animal: whey protein isolate supplement  Energy balance status: Eucaloric  **Comparator:** Placebo supplement  Intended Protein Amount: Supplement with 2.1 g of protein Carbohydrate: NR Fat: NR  Baseline Protein Amount Mean (SD): 76 (16) g/day; 1.1 (0.3) g/kg/d Carbohydrate Mean (SD): 190 (42) g/d Fat Mean (SD): 61 (20) g/d  Actual Protein Amount at the end of the study Mean (SD): 73.1 (16.9) g/d Carbohydrate Mean (SD): NR Fat Mean (SD): NR  Dietary Protein Intake Compliance (%): 80.8%  Protein type/source: Mixed  Energy balance status: Eucaloric  Study duration: 2 y | **Intervention:** High Protein   **How protein was administered:** Daily whey supplement protein shake before breakfast (30 g of protein)  **Protein Assessment Method:** 3 day weighed food record (2 weekdays and 1 weekend day) analyzed with AUSNUT99 database (Foodworks Professional edition version 3.02) by nutritionists trained in dietary assessment.  **Dietary Protein Intake Compliance:** Urinary nitrogen excretion was a compliance measure. Empty test containers returned by the participants.  **Comparator:** Placebo supplement  **How protein was administered:** Daily placebo supplement shake before breakfast (2.1 g of protein)  **Protein Assessment Method:** Same as above  **Dietary Protein Intake Compliance:** Same as above | **Muscle Mass** – Appendicular lean mass/ skeletal muscle mass  Measure/Method of Assessment: DXA (Hologic Discovery A fan-beam densitometer)   **Muscle Mass -** Adjusted appendicular lean mass/ skeletal muscle mass  Measure/Method of Assessment: Appendicular skeletal muscle mass divided by height squared  **Muscle Strength -** Handgrip strength  Measure/Method of Assessment: Measured using handgrip dynamometer  **Muscle Strength -** Knee flexion  Measure/Method of Assessment: Maximal muscle contraction against a strain gauge with the best of 3 attempts recorded.  **Muscle Strength -** Knee extension  Measure/Method of Assessment: Maximal muscle contraction against a strain gauge with the best of 3 attempts recorded.  **Physical Performance -** TUG  Measure/Method of Assessment: Timed while getting up, walking 3 meters, turning, returning to the chair, and sitting down again |

**Abbreviations**: aBW = adjusted body weight; BIA = bioelectrical impedance analysis; BMI = body mass index; COPD = chronic obstructive pulmonary disease; cpm = counts per minute d = days; DXA = Dual-energy x-ray absorptiometry; FFQ = Food frequency questionnaire; g = gram; h = hour; HDI = human development index; kg = kilograms; kg/m^2^ = kilograms per meters squared; LC n-3 PUFA = long chain n-3 polyunsaturated fatty acids; m = meters; METs = metabolic equivalents; min = minutes; ml = milliliters; mg = milligrams; NA = not applicable; NR = not reported; nmol/L = nanomoles per liter; NR = not reported PMID = PubMed Identification Number; PUFA = polyunstaturated fatty acids; RCT = randomized controlled trial; RM: Rep maximum; RoB = Risk of Bias; SD = standard deviation; SE = standard error; SEM = standard error of the mean; SPPB = Short Physical Performance Battery; TUG = Timed-Up-and-Go; wk = week; w/o = without; WI = wisconsin; y = year
^1^Studies overlap KQs

Supplementary Table 7. Evidence table for Sarcopenia Non-Randomized Controlled Trials (Adults)

| Study | Participants | Intervention (s) (Content) | Intervention (s) (Methods (of assessment) | Outcome (Measures and methods of assessment) |
| --- | --- | --- | --- | --- |
| PMID: 24219187 **Beasley 2013 (69)** Location/Country: USA HDI: Very high Setting: NR Urban/Rural: NR Study design: Prospective cohort study  Funding source: Nonprofit, government  **Risk of bias score: High** | Study of: Adults Total sample N: 134,961  **Quintile 1**: 6.6-13.1% of energy N: 26,994 % Female: 100% Mean Age (SD): 66.0 (7.2) y Race/ Ethnicity:  White: 78.1%  Black: 14.1%  Hispanic: 3.70%  American Indian: 0.54%  Asian/Pacific Islander: 2.32%  Other: 1.27% Menopausal status: Postmenopausal  Obesity status: NR Mean BMI (SD): 29.2 (7.0) kg/m^2^ Income level: < $20,000: 25.5%  $20,000-$49,999: 48.7% $50,000-$74,999: 15.1%  ≥ $75,000: 10.8% Education level: ≤ High school diploma or GED: 28.4%  Some college: 40.5%  ≥ College degree: 31.1% Mean physical activity level (SD): 9.9 (12) MET-hr/wk Health Status/ Comorbidities: Arthritis: 53.0%  Diabetes: 4.05%  Cancer: 10.4% Hypertension: 39.4% Emphysema: 5.40%  Hip fracture: 1.05%  Medication use: Unopposed estrogen use: 20.2 % Estrogen + progesterone use:11.4%  Supplement use: NR Pregnant or lactating: NR  **Quintile 2**: 13.1-13.8% of energy N: 26,991 % Female: 100% Mean Age (SD): 64.9 (7.0) y Race/ Ethnicity:  White: 83.4% Black: 9.04%  Hispanic: 3.29%  American Indian: 0.46%  Asian/Pacific Islander: 2.66%  Other: 1.15% Menopausal status: Postmenopausal  Obesity status: NR Mean BMI (SD): 28.6 (6.1) kg/m^2^ Income level: < $20,000: 18.2%  $20,000-$49,999: 48.5% $50,000 - $74,999: 18.4% ≥ $75,000: 14.9% Education level: ≤ High school diploma or GED: 23.7% Some college: 38.8% ≥College degree: 37.5 % Mean physical activity level (SD): 11.5 (13.1) MET-hr/wk Health Status/ Comorbidities: Arthritis: 49.9%  Diabetes: 4.30%  Cancer: 9.81% Hypertension: 36.7% Emphysema: 3.83% Hip fracture: 1.02%  Medication use: Unopposed estrogen use: 22.6%  Estrogen + progesterone use: 15.3%  Supplement use: NR Pregnant or lactating women: NR  **Quintile 3**: 13.9-14.6% of energy N: 26,992 % Female: 100% Mean Age (SD): 63.6 (6.9) y Race/ Ethnicity:  White: 85.4%  Black: 7.28%  Hispanic: 3.18%  American Indian: 0.34%  Asian/Pacific Islander: 2.71%  Other: 1.11%  Menopausal status: Postmenopausal  Obesity status: NR Mean BMI (SD): 28.0 (5.7) kg/m^2^ Income level: < $20,000: 15.0% $20,000-$49,999: 46.3% $50,000 - $74,999: 20.4% ≥ $75,000: 18.2% Education level: ≤ High school diploma or GED: 21.2% Some college: 37.7% ≥ College degree: 41.2% Mean physical activity level (SD): 12.5 (13.6) MET-hr/wk  Health Status/ Comorbidities: Arthritis: 48.5%  Diabetes: 4.45%  Cancer: 9.34% Hypertension: 34.3% Emphysema: 3.47% Hip fracture: 0.91%  Medication use: Unopposed estrogen use: 23.7% Estrogen + progesterone use: 18.2%  Supplement use: NR Pregnant or lactating: NR  **Quintile 4:** 14.7-15.4% of energy N: 26,992 % Female: 100% Mean Age (SD): 61.9 (6.7) y Race/ Ethnicity:  White: 86.5%  Black: 6.09%  Hispanic: 3.63%  American Indian: 0.31%, Asian/Pacific Islander: 2.54%  Other: 0.96% Menopausal status: Postmenopausal  Obesity status: NR Mean BMI (SD): 27.5 (5.4) kg/m^2^ Income level: < $20,000: 12.0%  $20,000-$49,999: 43.1% $50,000- $74,999: 22.6% ≥ $75,000: 22.3% Education level: ≤ High school diploma or GED: 18.6% Some college: 36.7% ≥ College degree: 44.8% Mean physical activity level (SD): 13.4 (13.7) MET-hr/wk Health Status/ Comorbidities: Arthritis: 46.0%  Diabetes: 4.42%  Cancer: 8.84%  Hypertension: 31.2% Emphysema: 2.96% Hip fracture: 0.86%  Medication use: Unopposed estrogen use: 24.5%  Estrogen + progesterone use: 21.0% Supplement use: NR Pregnant or lactating: NR  **Quintile 5:** 15.4-22.3% of energy N: 26,992 % Female: 100% Mean Age (SD): 59.5 ± 6.3 y Race/ Ethnicity:  White: 85.6%  Black: 5.90%  Hispanic: 3.86%  American Indian: 0.41% Asian/Pacific Islander: 3.15%  Other:1.08% Menopausal status: Postmenopausal  Obesity status: NR Mean BMI (SD): 26.6 (4.9) kg/m^2^ Income level: < $20,000: 9.40%  $20,000-$49,999: 37.7% $50,000 -$74,999: 24.1% ≥ $75,000: 28.9% Education level: ≤ High school diploma or GED: 15.6% Some college: 36.0% ≥ College degree: 48.5% Mean physical activity level (SD): 14.9 (14.8) MET-hr/wk Health Status/ Comorbidities: Arthritis: 40.6%  Diabetes: 4.46%  Cancer: 8.95% Hypertension: 27.1% Emphysema: 2.76% Hip fracture: 0.57% Medication use: Unopposed estrogen use: 24.4%  Estrogen + progesterone use: 24.5% Supplement use: NR Pregnant or lactating: NR | **Quintile 1:** 6.6-13.1% of energy  Baseline Protein Amount Mean (SD): 71.5 (12.1) g/d; 0.97 (0.17) g/kg/d Carbohydrate Mean (SD): NR Fat Mean (SD): NR  Protein Amount at the end of the study Mean (SD): NR Carbohydrate Mean (SD): NR Fat Mean (SD): NR  **Quintile 2:** 13.1-13.8% of energy  Baseline Protein Amount Mean (SD): 74.7 (11.0) g/d; 1.03 (0.17) g/kg/d Carbohydrate Mean (SD): NR Fat Mean (SD): NR  Protein Amount at the end of the study Mean (SD): NR Carbohydrate Mean (SD): NR Fat Mean (SD): NR  **Quintile 3:** 13.9-14.6% of energy  Baseline Protein Amount Mean (SD): 76.7 (10.5) g/d; 1.07 (0.17) g/kg/d Carbohydrate Mean (SD): NR Fat Mean (SD): NR  Protein Amount at the end of the study Mean (SD): NR Carbohydrate Mean (SD): NR Fat Mean (SD): NR  **Quintile 4**: 14.7-15.4% of energy  Baseline Protein Amount Mean (SD): 79.0 (10.1) g/d; 1.12 (0.18) g/kg/d Carbohydrate Mean (SD): NR Fat Mean (SD): NR  Protein Amount at the end of the study Mean (SD): NR Carbohydrate Mean (SD): NR Fat Mean (SD): NR  **Quintile 5:** 15.4-22.3% of energy  Baseline Protein Amount Mean (SD): 81.7 (9.9) g/d; 1.19 (0.20) g/kg/d Carbohydrate Mean (SD): NR Fat Mean (SD): NR  Protein Amount at the end of the study Mean (SD): NR Carbohydrate Mean (SD): NR Fat Mean (SD): NR  Protein type/ source: Mixed   Energy balance: Eucaloric  Study duration: 11.5 y | **Protein Assessment Method:** At baseline protein amount was derived from self-administered 122-item WHI FFQ. The WHI Nutritional Biomarkers Study was conducted to evaluate accuracy of self-reported protein consumption. | **Muscle Strength** - Grip strength   Measure/Method of Assessment: Measured using handgrip dynamometer   **Muscle Strength** - Chair stand test  Measure/Method of Assessment: Two 15-second trials of repeated chair stands with arms folded across the chest were conducted, with a 1–2-minute rest between trials.   **Physical Performance** - 6-m timed walk  Measure/Method of Assessment: Duration of the walk was measured at usual pace |
| PMID: 24522470 **Chan 2014 (70)** Location/Country: Hong Kong/ China HDI: Very high Setting: Community dwelling Urban/ Rural: NR Study design: Prospective cohort study  Funding source: Academic, nonprofit **Risk of bias score: High** | Study of: Adults Total sample N: 2,726   **Quartile 1**: ≤0.9 g of protein/kg/d N: 617 % Female: 62.1% Age Range: ≤69 y: 37.9% 70-74 y: 35.8%  75+ y: 26.3% Race/ Ethnicity: NR Menopausal status: Postmenopausal  Obesity status: NR BMI: <18.5 kg/m^2^: 1.6% 18.5-<23 kg/m^2^: 25.9% 23-24.9 kg/m^2^: 24.8% 25-29.9 kg/m^2^: 41.3% ≥30 kg/m^2^: 6.3% Income level: NR Education level: Primary or below: 80.9% Secondary/matriculation: 12.8%  University or above: 6.3% Mean physical activity level (SD): 90.2 (38.5) PASE score  Health status/ Comorbidities: NR Medication use: NR Supplement use: NR Pregnant or lactating: NR  **Quartile 2**: 0.91-1.2 g of protein/kg/d N: 677 % Female: 52.7% Age Range: ≤ 69 y: 37.2% 70-74 y: 35.5  75+ y: 27.3% Race/ Ethnicity: NR Menopausal status: Postmenopausal  Obesity status: NR BMI: <18.5 kg/m^2^: 3.1% 18.5-<23 kg/m^2^: 33.7% 23-24.9 kg/m^2^: 26.9% 25-29.9 kg/m^2^: 34.3% ≥30 kg/m^2^: 2.1% Income level: NR Education level: Primary or below: 71.3% Secondary/matriculation: 19.8%,  University or above: 8.9% Mean physical activity level (SD): 94.5 (44.9) PASE score Health status/ Comorbidities: NR Medication use: NR Supplement use: NR Pregnant or lactating: NR  **Quartile 3:** 1.21-1.6 g or protein/kg/day  N: 705 % Female: 44.1% Age Range:  ≤69 y: 39.0% 70-74 y: 38.0%  75+ y: 23.0% Race/ Ethnicity: NR Menopausal status: Postmenopausal  Obesity status: NR BMI: <18.5 kg/m^2^: 4.3% 18.5-<23 kg/m^2^: 42.0% 23-24.9 kg/m^2^: 27.7% 25-29.9 kg/m^2^: 23.8% ≥30 kg/m^2^: 2.3% Income level: NR Education level: Primary or below: 65% Secondary/matriculation: 21.8% University or above: 13.2% Mean physical activity level (SD): 95.7 (44.2) PASE score Health status/ Comorbidities: NR Medication use: NR Supplement use: NR Pregnant or lactating: NR  **Quartile 4:** ≥1.61 g of protein/kg/day N: 727 % Female: 36.3% Age Range:  ≤69 y: 40.7% 70-74 y: 35.1%  75+ y: 24.2% Race/ Ethnicity: NR Menopausal status: Postmenopausal  Obesity status: NR BMI:  <18.5 kg/m^2^: 8.1% 18.5-<23 kg/m^2^: 47.9% 23-24.9 kg/m^2^: 21.5% 25-29.9 kg/m^2^: 21.2% ≥30 kg/m2: 1.4% Income level: NR Education level: Primary or below: 60.9% Secondary/matriculation: 24.2% University or above: 14.9% Mean physical activity level (SD): 103.1(47.6) PASE score Health status/ Comorbidities: NR Medication use: NR Supplement use: NR Pregnant or lactating: NR | **Quartile 1**: ≤0.9 g of protein/kg/d  Baseline Protein Amount Mean (SD):  Animal protein:0.35 (0.11) g/kg/d Plant protein: 0.37 (0.10) g/kg/d Carbohydrate Mean (SD): NR Fat Mean (SD): NR  Protein Amount at the end of the study Mean (SD): NR Carbohydrate Mean (SD): NR Fat Mean (SD): NR  **Quartile 2**: 0.91-1.2 g of protein/kg/d  Baseline Protein Amount Mean (SD):  Animal protein: 0.56 (0.15) g/kg/d Plant protein: 0.50 (0.14) g/kg/d Carbohydrate Mean (SD): NR Fat Mean (SD): NR  Protein Amount at the end of the study Mean (SD): NR Carbohydrate Mean (SD): NR Fat Mean (SD): NR  **Quartile 3**: 1.21-1.6 g of protein/kg/day  Baseline Protein Amount Mean (SD): Animal protein: 0.77 (0.20) g/kg/d Plant protein: 0.63 (0.19) g/kg/d Carbohydrate Mean (SD): NR Fat Mean (SD): NR  Protein Amount at the end of the study Mean (SD): NR Carbohydrate Mean (SD): NR Fat Mean (SD): NR  **Quartile 4**: ≥1.61 g of protein/kg/day  Baseline Protein Amount Mean (SD): Animal protein: 1.21 (0.48) g/kg/d Plant protein: 0.89 (0.36) g/kg/d Carbohydrate Mean (SD): NR Fat Mean (SD): NR  Protein Amount at the end of the study Mean (SD): NR Carbohydrate Mean (SD): NR Fat Mean (SD): NR  Protein type/ source: Mixed  Energy balance: Eucaloric  Study duration: 4 y | **Protein Assessment Method:** At baseline, dietary intake was measured with a validated semi-quantitative FFQ. Using food tables from the Chinese Medical Sciences Institute and McCance and Widdowson, the mean daily quantitation of nutrients was determined. The quantity of animal and vegetable proteins consumed was calculated in addition to the overall protein intake. | **Physical Performance** - 6-m timed walk   Measure/Method of Assessment: Duration of the walk was measured as well as the number of steps.  **Physical Performance-** 20 cm narrow walk  Measure/Method of Assessment: Participants walked the 6-m course within a 20-cm narrow path and performance was scored for time.   **Muscle Mass** – Appendicular lean mass/ skeletal muscle mass  Measure/Method of Assessment: DXA (Hologic QDR-4500W, software version 11.2; hologic, Inc., Waltham, Ma, USA) |
| PMID: 37922694 **Chen 2023 (71)** Location/Country: China HDI: Very high Setting: Community dwelling Urban/Rural: NR Study design: Prospective cohort study Funding source: Other **Risk of bias score: High** | Study of: Adults Total sample N: 2709  **Arm 1:** Men N: 855 % Female: 0% Mean Age (SD): 60.4 (6.4) y Race/ Ethnicity: NR Menopausal status: NA Obesity status: NR Mean BMI (SD): 23.9 (2.8) kg/m^2^ Income level: NR Education level:  High school or below 65% College or above: 35% Mean physical activity level (SD): 34.9 (6.4) METs h/d Health status/ Comorbidities:  Type 2 diabetes: 9.8% Dyslipidemia: 20.5% Medication use: NR Supplement use: 13.5% Pregnant or lactating: NA  **Arm 2:** Women N: 1854 % Female: 100% Mean Age (SD): 57.5 (5.5) y Race/ Ethnicity: NR Menopausal status: 96.5% Obesity status: NR Mean BMI (SD): 23.3 (3.1) kg/m^2^ Income level: NR Education level:  High school or below 77.5% College or above: 22.5% Mean physical activity level (SD): 34.8 (5.6) METs h/d Health status/ Comorbidities:  Type 2 diabetes: 6.9% Dyslipidemia: 22.2% Medication use: NR Supplement use: 22.5% Pregnant or lactating: NR | **Arm 1:** Men  Baseline Protein Amount Mean (SD): 1.29 (0.24) g/d/kg  Carbohydrate Mean (SD): 279.4 (49.9) g/d Fat Mean (SD): 53.5 (16.0) g/d  Protein Amount at the end of the study Mean (SD): NR Carbohydrate Mean (SD): NR Fat Mean (SD): NR  **Arm 2:** Women  Baseline Protein Amount Mean (SD): 1.43 (0.28) g/d/kg  Carbohydrate Mean (SD): 217.7 (40.1) g/d Fat Mean (SD): 48.5 (13.8) g/d  Protein Amount at the end of the study Mean (SD): NR Carbohydrate Mean (SD): NR Fat Mean (SD): NR  Protein type/ source: Mixed  Energy balance status: Eucaloric  Study duration: 3.2 y | **Protein Assessment Method:** The dietary intake was assessed using a validated 79-item semi-quantitative, interviewer-administered, and paper-based food frequency questionnaire at baseline. | **Muscle Mass –** Appendicular lean mass/ skeletal muscle mass Measure/Method of Assessment: DXA (Hologic Inc. Discovery W, USA)  **Muscle Mass –** ASMI Measure/Method of Assessment: Appendicular muscle mass divided by squared height in meters  **Muscle Strength** – Handgrip strength Measure/Method of Assessment: Hand dynamometer  **Muscle Strength** – Chair stand test  Measure/Method of Assessment: Recorded how long it took participants to stand up and sit down five times |
| PMID: 32520344 **Elstgeest 2020 (72)** Location/Country: USA HDI: Very high Setting: Community dwelling Urban/Rural: Metropolitan Study design: Prospective cohort study Funding source: NR **Risk of bias score: High** | Study of: Adults Total sample N: 3075  **Arm 1:** Men N: 1163 % Female: 0% Mean Age (SD): 74.8 (2.9) y Menopausal status: NA Race/ Ethnicity: White: 68.8% Obesity status: NR Mean BMI (SD): 26.9 (3.8) kg/m^2^ Income level: NR Education level: Less than high school: 22.5% High school graduation: 25.9% Postsecondary education: 51.6% Mean physical activity level (SD): Walking 165 (295) min/wk Health status/ Comorbidities: 0 diseases: 14.8% 1 disease: 27.4% ≥2 diseases: 57.8% Medication use: Oral steroid use: 2.1% Supplement use: NR Pregnant or lactating: NA  **Arm 2**: Women  N: 1237 % Female: 51.5% Mean Age (SD): 74.4 (2.8) y  Menopausal status: Postmenopausal  Race/ Ethnicity: White: 59.1% Obesity status: NR Mean BMI (SD): 27.4 (5.4) kg/m^2^ Income level: NR Education level: Less than high school: 20.4% High school graduation: 39.1% Postsecondary education: 40.5% Mean physical activity level (SD): Walking 116 (228) min/wk Health status/ Comorbidities: 0 diseases: 11.2% 1 disease: 28.7% ≥2 diseases: 60.1% Medication use: Oral steroid use: 3.2% Supplement use: NR Pregnant or lactating: NR | **Arm 1:** Men  Baseline Protein Amount Mean (SD): 71.3 (26.6) g/d; 0.94 (0.36) g/kg aBW/d Carbohydrate Mean (SD): NR Fat Mean (SD): NR  Protein Amount at the end of the study Mean (SD): NR Carbohydrate Mean (SD): NR Fat Mean (SD): NR  **Arm 2:** Women  Baseline Protein Amount Mean (SD): 60.7 (22.3) g/d; 0.95 (0.36) g/kg aBW/d Carbohydrate Mean (SD): NR Fat Mean (SD): NR  Protein Amount at the end of the study Mean (SD): NR Carbohydrate Mean (SD): NR Fat Mean (SD): NR  Protein type/ source: Mixed  Energy balance status: Eucaloric  Study duration: 5 y | **Protein Assessment Method**: At baseline, dietary intake was assessed using a 108-item modified version of the Block FFQ. Block Dietary Data Systems were used to determine nutrient intake. | **Muscle Mass** - Appendicular lean mass/ skeletal muscle mass   Measure/Method of Assessment: DXA (Hologic 4500A, version 8.20a)  **Physical Performance**- 20-m walk  Measure/Method of Assessment: Participants were asked to walk a 20-m course at their usual walking pace. |
| PMID: 27465379 **Farsijani 2016 (73)** Location/Country: Canada HDI: Very high Setting: Community dwelling Urban/Rural: Urban and suburban  Study design: Prospective cohort study Funding source: Nonprofit, academic **Risk of bias: High** | Study of: Adults  Total sample N: 1793  **Quartile 1 (Men)**: Protein intake ≤62.12 g/d N: 88 % Female: 0% Mean Age (SD): 73.8 (4.1) y Race/ Ethnicity: NR Menopausal status: NA Obesity status: NR Mean BMI (SD): 27.1 (4.0) kg/m^2^ Income level: NR Mean education level (SD): 10.8 (5.5) y Physical activity level: NR Health status/ Comorbidities: Subjects free from cognitive impairment, COPD, class II heart failure and inflammatory digestive diseases, cancer  Medication use: NR Supplement use: NR Pregnant or lactating: NA  **Quartile 2 (Men)**: Protein intake 62.13-71.62 g/d N: 87 % Female: 0% Mean Age (SD): 74.4 (4.4) y Race/ Ethnicity: NR Menopausal status: NA Obesity status: NR Mean BMI (SD): 28.5 (4.4) kg/m^2^ Income level: NR Mean education level (SD): 10.0 (4.6) y Physical activity level: NR Health status/ Comorbidities: Subjects free from cognitive impairment, COPD, class II heart failure and inflammatory digestive diseases, cancer  Medication use: NR Supplement use: NR Pregnant or lactating: NA  **Quartile 3 (Men):** Protein intake 71.63-80.66 g/d N: 88 % Female: 0% Mean Age (SD): 73.4 (4.3) y Race/ Ethnicity: NR Menopausal status: NA Obesity status: NR Mean BMI (SD): 28.3 (3.9) kg/m^2^ Income level: NR Mean education level (SD): 10.1 (4.7) y Physical activity level: NR Health status/ Comorbidities: Subjects free from cognitive impairment, COPD, class II heart failure and inflammatory digestive diseases, cancer  Medication use: NR Supplement use: NR Pregnant or lactating: NA  **Quartile 4 (Men):** Protein intake ≥80.67 g/d N: 88 % Female: 0% Mean Age (SD): 72.6 (3.8) y Race/ Ethnicity: NR Menopausal status: NA Obesity status: NR Mean BMI (SD): 28.5 (4.1) kg/m^2^ Income level: NR Mean education level (SD): 11.5 (4.7) y Physical activity level: NR Health status/ Comorbidities: Subjects free from cognitive impairment, COPD, class II heart failure and inflammatory digestive diseases, cancer  Medication use: NR Supplement use: NR Pregnant or lactating: NA  **Quartile 1 (Women):** Protein intake ≤64.81 g/d N: 90 % Female: 100% Mean Age (SD): 73.6 (4.0) y Race/ Ethnicity: NR Menopausal status: NR Obesity status: NR Mean BMI (SD): 27.4 (4.3) kg/m^2^ Income level: NR Mean education level (SD): 10.6 (3.4) y Physical activity level: NR Health status/ Comorbidities: Subjects free from cognitive impairment, COPD, class II heart failure and inflammatory digestive diseases, cancer  Medication use: NR Supplement use: NR Pregnant or lactating: NR  **Quartile 2 (Women):** Protein intake 64.82-73.46 g/d N: 91 % Female: 100% Mean Age (SD): 74.6 (4.0) y Race/ Ethnicity: NR Menopausal status: NR Obesity status: NR Mean BMI (SD): 28.1 (5.3) kg/m^2^ Income level: NR Mean education level (SD): 10.6 (3.6) y Physical activity level: NR Health status/ Comorbidities: Subjects free from cognitive impairment, COPD, class II heart failure and inflammatory digestive diseases, cancer  Medication use: NR Supplement use: NR Pregnant or lactating: NR  **Quartile 3 (Women):** Protein intake 73.47-82.29 g/d N: 90 % Female: 100% Mean Age (SD): 73.4 (3.9) y Race/ Ethnicity: NR Menopausal status: NR Obesity status: NR Mean BMI (SD): 27.7 (4.4) kg/m^2^ Income level: NR Mean education level (SD): 10.5 (3.8) y Physical activity level: NR Health status/ Comorbidities: Subjects free from cognitive impairment, COPD, class II heart failure and inflammatory digestive diseases, cancer  Medication use: NR Supplement use: NR Pregnant or lactating: NR  **Quartile 4 (Women):** Protein intake ≥82.30 g/d N: 90 % Female: 100% Mean Age (SD): 72.8 (4.0) y Race/ Ethnicity: NR Menopausal status: NR Obesity status: NR Mean BMI (SD): 28.1 (4.9) kg/m^2^ Income level: NR Mean education level (SD): 10.8 (3.9) year Physical activity level: NR Health status/ Comorbidities: Subjects free from cognitive impairment, COPD, class II heart failure and inflammatory digestive diseases, cancer  Medication use: NR Supplement use: NR Pregnant or lactating: NR | **Quartile 1 (Men)**: Protein intake ≤62.12 g/d  Baseline Protein Amount Mean (SD): 64.3 (14.3) g/d Carbohydrate Mean (SD): NR Fat Mean (SD): NR  Protein Amount at the end of the study Mean (SD): NR Carbohydrate Mean (SD): NR Fat Mean (SD): NR  **Quartile 2 (Men)**: Protein intake 62.13-71.62 g/d  Baseline Protein Amount Mean (SD): 71.5 (16.0) g/d Carbohydrate Mean (SD): NR Fat Mean (SD): NR  Protein Amount at the end of the study Mean (SD): NR Carbohydrate Mean (SD): NR Fat Mean (SD): NR  **Quartile 3 (Men):** Protein intake 71.63-80.66 g/d  Baseline Protein Amount Mean (SD): 83.2 (17.3) g/d Carbohydrate Mean (SD): NR Fat Mean (SD): NR  Protein Amount at the end of the study Mean (SD): NR Carbohydrate Mean (SD): NR Fat Mean (SD): NR  **Quartile 4 (Men):** Protein intake ≥80.67 g/d  Baseline Protein Amount Mean (SD): 101.8 (21.3) g/d Carbohydrate Mean (SD): NR Fat Mean (SD): NR  Protein Amount at the end of the study Mean (SD): NR Carbohydrate Mean (SD): NR Fat Mean (SD): NR  **Quartile 1 (Women):** Protein intake ≤64.81 g/d  Baseline Protein Amount Mean (SD): 51.1 (12.5) g/d Carbohydrate Mean (SD): NR Fat Mean (SD): NR  Protein Amount at the end of the study Mean (SD): NR Carbohydrate Mean (SD): NR Fat Mean (SD): NR  **Quartile 2 (Women):** Protein intake 64.82-73.46 g/d  Baseline Protein Amount Mean (SD): 62.6 (12.0) g/d Carbohydrate Mean (SD): NR Fat Mean (SD): NR  Protein Amount at the end of the study Mean (SD): NR Carbohydrate Mean (SD): NR Fat Mean (SD): NR  **Quartile 3 (Women):** Protein intake 73.47-82.29 g/d  Baseline Protein Amount Mean (SD): 69.8 (10.6) g/d Carbohydrate Mean (SD): NR Fat Mean (SD): NR  Protein Amount at the end of the study Mean (SD): NR Carbohydrate Mean (SD): NR Fat Mean (SD): NR  **Quartile 4 (Women):** Protein intake ≥82.30 g/d  Baseline Protein Amount Mean (SD): 86.7 (16.7) g/d Carbohydrate Mean (SD): NR Fat Mean (SD): NR  Protein Amount at the end of the study Mean (SD): NR Carbohydrate Mean (SD): NR Fat Mean (SD): NR   Protein type/ source: Mixed  Energy balance status: Eucaloric   Study duration: 2 y | **Protein Assessment Method:** Protein intake was assessed using 24-h dietary recalls. A total of 6 nonconsecutive recalls were collected: 3 at baseline and 3 at the 2-y follow-up. Recalls were analyzed with the CANDAT nutrient analysis software | **Muscle Mass** - Lean mass   Measure/ Method of Assessment: DXA (Lunar Prodigy; GE Medical)  **Muscle Mass** - Appendicular lean mass/ skeletal muscle mass  Measure/Method of Assessment: Calculated as the sum of nonbone LM of arms and legs. |
| PMID: 29191494 **Granic  2017 (74)** Location/Country: UK HDI: Very high Setting: Community dwelling Urban/Rural: NR Study design: Prospective cohort study Funding source: Nonprofit, academic **Risk of bias score: High** | Study of: Adults  Total sample N: 722  **Arm 1:** Low protein intake (<1 g of protein/kg aBW/d)  N: 390 % Female: 66.9% Mean Age (SD): NR Race/ Ethnicity: NR Menopausal status:  Postmenopausal  Obesity status: NR Mean BMI (SD): NR Income level: NR Education level: 0-9 y: 68.0% 10-11 y: 21.4% ≥12 y:10.6% Physical activity level:  Low: 18.5 %  Moderate: 47.7%  High: 33.8 % Health status/ Co-morbidities:  Mean multimorbidity (SD): 2.28 (1.21) Depressive symptoms: 0 to 5 (none): 78.1% 6 to 7 (mild): 13.5% >8 (severe): 8.2% Arthritis in hands: 6.7% Medication use: NR Supplement use: NR Pregnant or lactating: NR  **Arm 2:** Good protein intake (≥1 g of protein/kg aBW/d)  N: 390 % Female: 51.8% Mean Age (SD): NR Race/ Ethnicity: NR Menopausal status: Postmenopausal Obesity status: NR Mean BMI (SD): NR Income level: NR Education level: 0-9 y: 58.8% 10-11 y: 26.4% ≥12 y:14.8% Physical activity level: Low: 16.3% Moderate: 42.3% High: 41.4% Health status/ Comorbidities: Mean multimorbidity (SD): 2.19 (1.25) Depressive symptoms: 0 to 5 (none): 83.2% 6 to 7 (mild): 10.9% >8 (severe): 5.9% Arthritis in hands: 6.4% Medication use: NR Supplement use: NR Pregnant or lactating: NR | **Arm 1:** Low protein intake (<1 g of protein/kg aBW/d)    Baseline Protein Intake: <1 g of protein/ kg aBW/d Carbohydrate: NR Fat: NR  Protein Amount at the end of the study: NR Carbohydrate: NR Fat: NR  **Arm 2**: Good protein intake (≥1 g of protein/kg aBW/d)   Baseline Protein Intake: ≥1 g of protein/ kg aBW/d Carbohydrate: NR Fat: NR  Protein Amount at the end of the study: NR Carbohydrate: NR Fat: NR  Protein type/ source: Mixed  Energy balance status: Eucaloric   Study duration: 5 y | **Protein Assessment Method:** At baseline, protein intake was estimated with a validated 24-hr multiple pass dietary recall (24-h MPR). A food code was assigned to each food and 2-day intakes were entered in a Microsoft Access based dietary data system. The codes were further grouped in118 food groups based on McCance and Widdowson's composition of-foods 6th edition. | **Muscle Strength-** Grip strength   Measure/Method of Assessment: Measured using handgrip dynamometer   **Physical Performance** - TUG  Measure/Method of Assessment: Measures the time that takes to rise from a chair without using arms, walk 3 m at usual pace, turn, return to the chair, and sit down. |
| PMID: 33515002 **Hengeveld 2021 (75)** Location/Country: Canada HDI: Very high Setting: Community dwelling Urban/Rural: Urban Study design: Prospective cohort study Funding source: NR **Risk of bias score: High** | Study of: Adults Total sample N: 1754  **Arm 1**: Men N: 524 % Female: 0% Mean Age (SD): 74.8 (4.0) y Race/ Ethnicity: NR Menopausal status: NA Obesity status: NR Mean BMI (SD): 28.1 (4.0) kg/m^2^ Income level: NR Mean education level (SD): 12.0 (5.1) y Mean physical activity level (SD): 118 (55) PASE score Health status/ Comorbidities:  Chronic diseases 0: 10.3% 1-2: 33.8% ≥3: 55.9% Medication use: 0: 10.7%  1-4: 49.8%   ≥5: 39.5% Supplement use: NR Pregnant or lactating: NA  **Arm 2**: Women N: 574 % Female: 32.72% Mean Age (SD): 75.2 (4.2) y Race/ Ethnicity: NR Menopausal status: NR Obesity status: NR Mean BMI (SD): 27.4 (4.7) kg/m^2^ Income level: NR Mean education level (SD): 11.6 (3.9) y Mean physical activity level (SD): 94 (45) PASE score Health status/ Comorbidities: Chronic diseases 0: 3.5% 1–2: 26.1% ≥3: 70.4% Medication use: 0: 5.2% 1-4: 43.9% ≥5: 50.9% Supplement use: NR Pregnant or lactating: NR | **Arm 1:** Men  Baseline Protein Amount Mean (SD): 82.7 (19.4) g/d; 1.06 (0.28) g/kg/d; 1.13 (0.27) g/kg aBW/d; 16.1 (2.5) % of energy Carbohydrate Mean (SD): NR Fat Mean (SD): NR  Protein Amount at the end of the study Mean (SD): NR Carbohydrate Mean (SD): NR Fat Mean (SD): NR  **Arm 2:** Women   Baseline Protein Intake Mean (SD): 68.3 (15.0) g/d; 1.07 (0.30) g/ kg BW/d; 1.12 (.26) g/kg aBW/d; 16.6 (2.5) of energy Carbohydrate Mean (SD): NR Fat Mean (SD): NR  Protein Amount at the end of the study Mean (SD): NR Carbohydrate Mean (SD): NR Fat Mean (SD): NR  Protein type/ source: Mixed  Energy balance status: Eucaloric  Study duration: 3 y | **Protein Assessment Method**: 3 nonconsecutive 24-h dietary recalls were collected. | **Muscle Strength** - Handgrip strength   Measure/Method of Assessment: Measured using handgrip dynamometer   **Muscle Strength** - Knee extensor  Measure/Method of Assessment: Measured using isometric contraction of the knee extensors.  **Physical Performance** - TUG   Measure/Method of Assessment: Measures the time that takes to rise from a chair without using arms, walk 3 m at usual pace, turn, return to the chair, and sit down. |
| PMID: 18175749 **Houston 2008 (76)** Location/Country: USA  HDI: Very high Setting: Community dwelling Urban/ Rural: Metropolitan Study design: Prospective cohort study Funding source: Government  **Risk of bias score: High** | Study of: Adults Total sample N: 2066  **Quintile 1**: Protein intake cut-offs NR N: NR % Female: 53.3% Mean Age (SD): 74.4 (2.8) y Race/ Ethnicity: Black: 46.7% Menopausal status: Postmenopausal Obesity status: NR Mean BMI (SD): 27.2 (4.8) kg/m^2^ Income level: NR Education level: <High school: 25.9% Mean physical activity level (SD): Walking 115.8 (185.7) min/wk Health status/ Comorbidities: Diabetes: 15% Ischemic heart disease: 19.4% Congestive heart failure: 1.7% Cerebrovascular disease: 7% COPD: 10.9% Cancer: 16.2% Medication use:  Oral steroids use: 2.7% Supplement use: NR Pregnant or lactating: NR  **Quintile 2:** Protein intake cut-offs NR N: NR % Female: 53.3% Mean Age (SD): 74.7 (2.9) y Race/ Ethnicity: Black: 36.6% Menopausal status: Postmenopausal Obesity status: NR Mean BMI (SD): 27.1 (4.5) kg/m^2^ Income level: NR Education level: <High school: 20.8% Mean physical activity level (SD): Walking 131.5 (281.0) min/wk Health status/ Comorbidities: Diabetes: 17% Ischemic heart disease: 20.8% Congestive heart failure: 1.7% Cerebrovascular disease: 9.2% COPD: 11.6% Cancer: 18.4% Medication use: Oral steroids use: 2.2% Supplement use: NR Pregnant or lactating: NR  **Quintile 3:** Protein intake cut-offs NR N: NR % Female: 53.1% Mean Age (SD): 74.5 (2.9) y Race/ Ethnicity: Black: 32.4% Menopausal status: Postmenopausal Obesity status: NR Mean BMI (SD): 27.0 (4.6) kg/m^2^ Income level: NR Education level: <High school: 19.8% Mean physical activity level (SD): Walking 137.6 (231.5) min/wk Health status/ Comorbidities: Diabetes: 20.8% Ischemic heart disease: 17.6%  Congestive heart failure: 2.4%  Cerebrovascular disease: 6.3% COPD: 12.1%  Cancer: 19.3% Medication use:  Oral steroid use: 3.6% Supplement use: NR Pregnant or lactating: NR  **Quintile 4:** Protein intake cut-offs NR N: NR % Female: 53.3% Mean Age (SD): 74.6 (2.9) y Race/ Ethnicity: Black: 29.8% Menopausal status: Postmenopausal Obesity status: NR Mean BMI (SD): 26.9 (4.3) kg/m^2^ Income level: NR Education level: <High school: 20.6% Mean physical activity level (SD): Walking 147.5 (298.2) min/wk Health status/ Comorbidities: Diabetes: 20.8% Ischemic heart disease: 19.8%  Congestive heart failure: 2.9%  Cerebrovascular disease: 6.8% COPD: 9.9%  Cancer: 18.6% Medication use:  Oral steroid use: 2.9% Supplement use: NR Pregnant or lactating: NR  **Quintile 5**: Protein intake cut-offs NR N: NR % Female: 53.3% Mean Age (SD): 74.5 (2.8) y Race/ Ethnicity: Black: 31.7% Menopausal status: Postmenopausal  Obesity status: NR Mean BMI (SD): 28.0 (5.1) kg/m^2^ Income level: NR Education level: <High school: 18.2% Mea physical activity level (SD): Walking 155.7 (265.4) min/wk Health status/ Comorbidities: Diabetes: 22.8% Ischemic heart disease: 21.6%  Congestive heart failure: 2.9%  Cerebrovascular disease: 7.5% COPD: 9.2%  Cancer:19.4% Medication use:  Oral steroids use: 3.4% Supplement use: NR Pregnant or lactating: NR | **Quintile 1**: Protein intake cut-offs NR  Baseline Protein Amount Mean (SD): 56.9 (18.6) g/d; 0.8 (0.3) g/kg/d; 10.9% of energy Carbohydrate Mean (SD): 55.1% of energy Fat Mean (SD): 34.8% of energy  Protein Amount at the end of the study Mean (SD): NR Carbohydrate Mean (SD): NR Fat Mean (SD): NR  **Quintile 2:** Protein intake cut-offs NR  Baseline Protein Amount Mean (SD): 53.6 (19.8) g/d; 0.7 (0.3) g/kg/d; 12.7% of energy Carbohydrate Mean (SD): 55.1% of energy Fat Mean (SD): 33.2% of energy  Protein Amount at the end of the study Mean (SD): NR Carbohydrate Mean (SD): NR Fat Mean (SD): NR  **Quintile 3**: Protein intake cut-offs NR  Baseline Protein Amount Mean (SD): 59.2 (18.1) g/d; 0.8 (0.3) g/kg/d; 14.2% of energy Carbohydrate Mean (SD): 53.5% of energy Fat Mean (SD): 33.6% of energy  Protein Amount at the end of the study Mean (SD): NR Carbohydrate Mean (SD): NR Fat Mean (SD): NR  **Quintile 4:** Protein intake cut-offs NR  Baseline Protein Amount Mean (SD): 67.1 (19.2) g/d; 0.9 (0.3) g/kg/d; 15.9% of energy Carbohydrate Mean (SD): 52.7% of energy Fat Mean (SD): 32.5% of energy  Protein Amount at the end of the study Mean (SD): NR Carbohydrate Mean (SD): NR Fat Mean (SD): NR  **Quintile 5:** Protein intake cut-offs NR  Baseline Protein Amount Mean (SD): 91.0 (27.1) g/d; 1.2 (0.4) g/kg/d; 18.6% of energy Carbohydrate Mean (SD): 50.4% of energy Fat Mean (SD): 32.1% of energy  Protein Amount at the end of the study Mean (SD): NR Carbohydrate Mean (SD): NR Fat Mean (SD): NR  Protein type/ source: Mixed  Energy balance status: Eucaloric   Study duration: 3 y | **Protein Assessment Method:** Study participants completed a 108-item interviewer-administered FFQ. The FFQ was analyzed for micronutrient and macronutrient content by Block Dietary Data Systems | **Muscle Mass** - Total body lean mass  Measure/Method of Assessment: DXA (Hologic 4500A, version 8.20a) |
| PMID: 26857389 **Isanejad  2016 (77)** Location/Country: Finland HDI: Very high Setting: Community dwelling Urban/ Rural: Urban Study design: Prospective cohort study Funding source: Nonprofit, academic **Risk of bias score: Very high** | Study of: Adults  Total sample N: 552  **Tertile 1**: Protein intake ≤0.8 g of protein/kg/d N: 171 % Female: 100% Mean Age (SD): 68.0 (1.9) y Race/ Ethnicity: NR Menopausal status: Postmenopausal  Obesity status: NR Mean BMI (SD): 29.9 (4.4) kg/m^2^ Income level: NR Education level: NR Mean physical activity level (SD): 100.2 (112.6) times/month x strenuousness Health status/ Comorbidities: NR Medication use: NR Supplement use: Ca and vitamin D  Pregnant or lactating: NR  **Tertile 2:** Protein intake 0.81–1.19 g of protein/kg/d N: 269 % Female: 100% Mean Age (SD): 67.8 (1.9) y Race/ Ethnicity: NR Menopausal status: Postmenopausal Obesity status: NR Mean BMI (SD): 27.1 (3.9) kg/m^2^ Income level: NR Education level: NR Mean physical activity level (SD): 106.4 (72.5) times/month x strenuousness Health status/ Comorbidities: NR Medication use: NR Supplement use:  Ca and vitamin D  Pregnant or lactating: NR  **Tertile 3**: Protein intake ≥1.2 g of protein/kg/d N: 112 % Female: 100% Mean Age (SD): 67.7 (1.8) y Race/ Ethnicity: NR Menopausal status: Postmenopausal Obesity status: NR Mean BMI (SD): 25.3 (3.4) kg/m^2^ Income level: NR Education level: NR Mean physical activity level (SD): 111.4 (140.3) times/month x strenuousness Health status/ Comorbidities: NR Medication use: NR Supplement use: Ca and Vitamin D Pregnant or lactating: NR | **Tertile 1**: Protein intake ≤0.8 g of protein/kg/d  Baseline Protein Amount Mean (SD): 51.4 (10.3) g/d; 16.4 (3.1) % of energy Carbohydrate Mean (SD): 165.7 (45.5) g/d Fat Mean (SD): 43.6 (14.5) g/d  Protein Amount at the end of the study Mean (SD): NR Carbohydrate Mean (SD): NR Fat Mean (SD): NR  **Tertile 2:** Protein intake 0.81–1.19 g of protein/kg/d  Baseline Protein Amount Mean (SD): 65.0 (10.2) g/d; 17.4 (2.5) % of energy Carbohydrate Mean (SD): 187.6 (37.0) g/d Fat Mean (SD): 53.9 (15.1) g/d  Protein Amount at the end of the study Mean (SD): NR Carbohydrate Mean (SD): NR Fat Mean (SD): NR  **Tertile 3**: Protein intake ≥1.2 g of protein/kg/d  Baseline Protein Amount Mean (SD): 83.4 (14.1) g/d; 18.6 (3.1) % of energy Carbohydrate Mean (SD): 219.1 (46.3) g/d Fat Mean (SD): 63.1 (18.2) g/d  Protein Amount at the end of the study Mean (SD): NR Carbohydrate Mean (SD): NR Fat Mean (SD): NR  Protein type/ source: Mixed  Energy balance status: Eucaloric  Study duration: 3 y | **Protein Assessment Method**: Baseline dietary intake was collected by using 3-d food record. Subjects were instructed to write down everything they ate and drank and to evaluate the amount of food consumed using household measures. Nutritional intake from food was calculated using Nutrica program. Collected data provided. | **Muscle Mass** - Lean mass  Measure/Method of  Assessment: DXA (Lunar Prodigy)  **Muscle Strength** - Handgrip strength    Measure/Method of Assessment: Measured using handgrip dynamometer   **Muscle Strength** - Knee extension   Measure/ Method of Assessment: NR  **Muscle Strength** - Chair rise test   Measure/Method of Assessment: Number of chair rises in 30 seconds |
| PMID: 33740517 **Kim 2021 (78)** Location/Country: Korea HDI: Very high Setting: NR Urban/ Rural: NR Study design: Prospective cohort study Funding source: NR **Risk of bias score: High** | Study of: Adults  Total sample N: 32,458  **Tertile 1 (Male):** Protein intake <0.8 g/kg/d N: 5126 % Female: 0% Median Age (IQR): 57.0 (50.0-62.0) y Race/ Ethnicity: NR Menopausal status: NA Obesity status: NR Median BMI (IQR): 24.9 (23.3-26.7) kg/m^2^ Income level:  Low income: 11.4% Education level: ≤Elementary school: 1.3% Middle or high school: 23.3% ≥College: 75.4% Physical activity level: NR Health status/ Comorbidities: Diabetes: 11.6% Hypertension: 28.9% Dyslipidemia:12.9%  Stroke: 2.1% Coronary artery disease: 5.4% Cancer: 3.1% Medication use: NR Supplement use: NR Pregnant or lactating: NA  **Tertile 2 (Male):** Protein intake 0.8-1.2 g/kg/d N: 4449 % Female: 0% Median Age (IQR): 56.0 (49.0-62.0) y Race/ Ethnicity: NR Menopausal status: NA Obesity status: NR Median BMI (IQR): 24.1 (22.5-25.8) kg/m^2^ Income level: Low income: 8.0% Education level: ≤Elementary school: 0.9% Middle or high school: 17.6% ≥College: 81.5% Physical activity level: NR Health status/ Comorbidities: Diabetes: 10.1%  Hypertension: 23.7% Dyslipidemia: 12.2%  Stroke: 1.8% Coronary artery disease: 4.3% Cancer: 2.8% Medication use: NR Supplement use: NR Pregnant or lactating: NA  **Tertile 3 (Male):** Protein intake >1.2 g/kg/d N: 1783 % Female: 0 Median Age (IQR): 55.0 (48.0-62.0) y Race/ Ethnicity: NR Menopausal status: NA Obesity status: NR Median BMI (IQR): 23.6 (21.8-25.3) kg/m^2^ Income level:  Low income: 8.3% Education level: ≤Elementary school: 1.1%, Middle or high school: 16.8%,  ≥College: 82.1% Physical activity level: NR Health status/ Comorbidities: Diabetes: 7.2%  Hypertension: 20.5% Dyslipidemia: 9.0% Stroke: 1.1% Coronary artery disease: 2.7%  Cancer: 3.5% Medication use: NR Supplement use: NR Pregnant or lactating: NA  **Tertile 1 (Female):** Protein intake <0.8 g/kg/d N: 7545 % Female: 23.2% Median Age (IQR): 54.0 (49.-60.0) y Race/ Ethnicity: NR Menopausal status: NR Obesity status: NR Median BMI (IQR): 24.2 (22.4-26.3) kg/m^2^ Income level:  Low income: 15.3% Education level: ≤Elementary school: 3.4% Middle or high school: 40.6% ≥College: 56.0% Physical activity level: NR Health status/ Comorbidities: Diabetes: 6.9%  Hypertension: 22.7% Dyslipidemia:13.4%  Stroke: 0.9% Coronary artery disease: 2.5% Cancer: 4.8% Medication use: NR Supplement use: NR Pregnant or lactating: NR  **Tertile 2 (Female):** Protein intake 0.8-1.2 g/kg/d N: 8644 % Female: 26.6% Median Age (IQR): 52.0 (47.0-58.0) y Race/ Ethnicity: NR Menopausal status: NR Obesity status: NR Median BMI (IQR): 23.1 (21.5-25.0) kg/m^2^ Income level:  Low income: 9.1% Education level: ≤Elementary school: 1.8% Middle or high school: 29.9%  ≥College: 68.3% Physical activity level: NR Health status/ Comorbidities: Diabetes: 4.5%  Hypertension: 16.7% Dyslipidemia: 10.7%  Stroke: 0.6%  Coronary artery disease: 1.9% Cancer: 4.5% Medication use: NR Supplement use: NR Pregnant or lactating: NR  **Tertile 3 (Female):** Protein intake >1.2 g/kg/d N: 4911 % Female: 15.1% Median Age (IQR): 51.0 (46.0-57.0) y Race/ Ethnicity: NR Menopausal status: NR Obesity status: NR Median BMI (IQR): 22.3 (20.7-24.1) kg/m^2^ Income level: Low income: 6.9% Education level: ≤Elementary school: 1.1% Middle or high school: 21.1%,  ≥College: 77.8% Physical activity level: NR Health status/ Comorbidities: Diabetes: 3.3% Hypertension: 12.4% Dyslipidemia: 9.5%  Stroke: 0.6% Coronary artery disease: 1.2% Cancer: 4.6% Medication use: NR Supplement use: NR Pregnant or lactating: NR | **Tertile 1 (Male)**: Protein intake <0.8 g/kg/d  Baseline Protein Amount Median (IQR): 45.3 (38.9-51.7) g/d; 12.5 (11.3-14.0) % of energy Carbohydrate Median (IQR): 73.0 (68.3-76.7) % of energy Fat Median (IQR): 13.1 (10.0-16.8) % of energy  Protein Amount at the end of the study Median (IQR): NR Carbohydrate Median (IQR): NR Fat Median (IQR): NR  **Tertile 2 (Male):** Protein intake 0.8-1.2 g/kg/d  Baseline Protein Amount Median (IQR): 65.6 (58.8-73.4) g/d; 13.1 (11.8-14.7) % of energy Carbohydrate Median (IQR): 70.9 (66.1-74.9) % of energy Fat Median (IQR): 14.9 (11.6-18.5) % of energy  Protein Amount at the end of the study Median (IQR): NR Carbohydrate Median (IQR): NR Fat Median (IQR): NR  **Tertile 3 (Male):** Protein intake >1.2 g/kg/d  Baseline Protein Amount Median (IQR): 94.8 (83.9-109.7) g/d; 13.7 (12.3-15.6) % of energy Carbohydrate Median (IQR): 69.2 (63.7-73.3) % of energy Fat Median (IQR): 16.2 (12.8-20.4) % of energy  Protein Amount at the end of the study Median (IQR): NR Carbohydrate Median (IQR): NR Fat Median (IQR): NR  **Tertile 1 (Female):** Protein intake <0.8 g/kg/d  Baseline Protein Amount Median (IQR): 38.5 (32.8-43.6) g/d; 12.6 (11.2-14.3) % of energy Carbohydrate Median (IQR): 73.5 (68.5-77.7) % of energy Fat Median (IQR): 12.8 (9.4-16.9) % of energy  Protein Amount at the end of the study Median (IQR): NR Carbohydrate Median (IQR): NR Fat Median (IQR): NR  **Tertile 2 (Female):** Protein intake 0.8-1.2 g/kg/d  Baseline Protein Amount Median (IQR): 55.4 (49.9-61.7) g/d; 13.2 (11.8-14.9) % of energy Carbohydrate Median (IQR): 71.6 (66.7-75.7) % of energy Fat Median (IQR): 14.4 (11.0-18.3) % of energy  Protein Amount at the end of the study Median (IQR): NR Carbohydrate Median (IQR): NR Fat Median (IQR): NR  **Tertile 3 (Female):** Protein intake >1.2 g/kg/d  Baseline Protein Amount Median (IQR): 80.4 (71.1-3.7) g/d; 14.0 (12.5-15.7) % of energy Carbohydrate Median (IQR): 69.1 (64.0-73.8) % of energy Fat Median (IQR): 16.4 (12.6-20.3) % of energy  Protein Amount at the end of the study Median (IQR): NR Carbohydrate Median (IQR): NR Fat Median (IQR): NR  Protein type/ source: Mixed  Energy balance status: Eucaloric   Study duration: 4 y | **Protein Assessment Method**: Subjects’ dietary protein intake was assessed using the 103-item semi-quantitative FFQ at baseline and the follow-up surveys. The FFQ is used to estimate nutrient intake from portion size and the frequency of food consumption. Protein was estimated from the sum of the intake of each food item, based on the food composition tables. | **Muscle Strength** - Handgrip strength  Measure/Method of Assessment: Measured using handgrip dynamometer |
| PMID: 28179224 **Mangano 2017^1^ (26)** Location/Country: USA HDI: Very high Setting: NR Urban/ Rural: NR Study design: Prospective cohort study Funding source: Government **Risk of bias score: High** | Study of: Adults  Total sample N: 2,986   **Arm 1**: Protein food cluster (Fast food, full-fat dairy) N: 458 % Female: 44% Mean Age (SD): 39.3 (8.5) y Race/ Ethnicity: NR Menopausal status: 6% nonestrogenic  Obesity status: NR Mean BMI (SD): 26.5 (5.0) kg/m^2^ Income level: NR Education level: NR Mean physical activity level: 37.2 (7.4) PAI Health status/ Comorbidities: NR Medication use: NR Supplement use: Calcium supplements: 19%, Vitamin D supplements: 40% Pregnant or lactating: NR   **Arm 2**: Protein food cluster 2 (Fish) N: 605 % Female: 58% Mean Age (SD): 42.2 (9.0) y Race/ Ethnicity: NR Menopausal status: 14% nonestrogenic Obesity status: NR Mean BMI (SD): 26.8 (5.3) kg/m^2^ Income level: NR Education level: NR Mean physical activity level: 37.4 (7.6) PAI Health status/ Comorbidities: NR Medication use: NR Supplement use:  Calcium supplements: 43%, Vitamin D supplements: 53% Pregnant or lactating: NR   **Arm 3**: Protein food cluster 3 (Red meat) N: 640  % Female: 48% Mean Age (SD): 41.5 (8.3) y Race/ Ethnicity: NR Menopausal status: 13% nonestrogenic Obesity status: NR Mean BMI (SD): 27.4 (5.6) kg/m^2^ Income level: NR Education level: NR Mean physical activity level: 37.5 (8.3) PAI Health status/ Comorbidities: NR Medication use: NR Supplement use: Calcium supplements: 30% Vitamin D supplements: 39% Pregnant or lactating: NR   **Arm 4**: Protein food cluster 4 (Chicken) N: 735 % Female: 58% Mean Age (SD): 39.3 (8.3) y Race/ Ethnicity: NR Menopausal status: 7% nonestrogenic Obesity status: NR Mean BMI (SD): 26.7 (5.3) kg/m^2^ Income level: NR Education level: NR Mean physical activity level: 37.0 (7.2) PAI Health status/ Comorbidities: NR Medication use: NR Supplement use: Calcium supplements: 36%, Vitamin D supplements: 46% Pregnant or lactating: NR   **Arm 5**: Protein food cluster 5 (Low-fat milk) N: 434 % Female: 58% Mean Age (SD): 40.9 (8.6) y Race/ Ethnicity: NR Menopausal status: 11% nonestrogenic Obesity status: NR Mean BMI (SD): 26.8 (5.0) kg/m^2^ Income level: NR  Education level: NR Mean physical activity level: 37.8 (7.3) PAI Health status/ Comorbidities: NR Medication use: NR Supplement use:  Calcium supplements: 40%, Vitamin D supplements: 50% Pregnant or lactating: NR   **Arm 6**: Protein food cluster 6 (Legumes)  N: 114 % Female: 79% Mean Age (SD): 38.6 (9.4) y Race/ Ethnicity: NR Menopausal status: 7% nonestrogenic Obesity status: NR Mean BMI (SD): 23.9 (4.6) kg/m^2^ Income level: NR Education level: NR Mean physical activity level: 36.1 (5.8) PAI Health status/ Comorbidities: NR Medication use: NR Supplement use: Calcium supplements: 47%, Vitamin D supplements: 56% Pregnant or lactating: NR | **Arm 1:** Protein food cluster (Fast food, full-fat dairy)   Baseline Protein Amount Mean (SD): 88 (31) g/d Carbohydrate Mean (SD): NR Fat Mean (SD): NR   Protein Amount at the end of the study Mean (SD): NR Carbohydrate Mean (SD): NR Fat Mean (SD): NR    **Arm 2**: Protein food cluster 2 (Fish)   Baseline Protein Amount Mean (SD): 90 (31) g/d Carbohydrate Mean (SD): NR  Fat Mean (SD): NR   Protein Amount at the end of the study Mean (SD): NR Carbohydrate Mean (SD): NR Fat Mean (SD): NR    **Arm 3:** Protein food cluster 3 (Red meat)   Baseline Protein Amount Mean (SD): 97 (29) g/d Carbohydrate Mean (SD): NR Fat Mean (SD): NR   Protein Amount at the end of the study Mean (SD): NR Carbohydrate Mean (SD): NR Fat Mean (SD): NR    **Arm 4**: Protein food cluster 4 (Chicken)   Baseline Protein Amount Mean (SD): 95 (35) g/d Carbohydrate Mean (SD): NR Fat Mean (SD): NR   Protein Amount at the end of the study Mean (SD): NR Carbohydrate Mean (SD): NR Fat Mean (SD): NR    **Arm 5**: Protein food cluster 5 (Low-fat milk)   Baseline Protein Amount Mean (SD): 98 (31) g/d Carbohydrate Mean (SD): NR Fat Mean (SD): NR   Protein Amount at the end of the study Mean (SD): NR Carbohydrate Mean (SD): NR Fat Mean (SD): NR    **Arm 6**: Protein food cluster 6 (Legumes)   Baseline Protein Amount Mean (SD): 83 (34) g/d Carbohydrate: NR Fat: NR   Protein Amount at the end of the study Mean (SD): NR Carbohydrate Mean (SD): NR Fat Mean (SD): NR    Protein type/source: Mixed   Energy balance status: Eucaloric    Study duration: 9 y | **Protein Assessment Method:** Typical dietary intakes of foods and nutrients were assessed with the use of the Harvard 126-item semiquantitative and validated general population 88 FFQ. Protein intake was assessed during the years 2002-2005. | **Muscle Mass** - Lean mass   Measure/Method of Assessment: DXA Fan beam densitometer (GE Lunar Prodigy)  **Muscle Mass** - Appendicular lean mass/ skeletal muscle mass  Measure/Method of Assessment: DXA Fan beam densitometer (GE Lunar Prodigy) |
| PMID: 33829238 **Mendonca 2021 (79)** Location/Country: USA, Canada, Netherlands and UK  HDI: Very high Setting: Mixed Urban/ Rural: Mixed Study Design:  Pooled analysis of longitudinal observational study Funding source: NR **Risk of bias: Very high** | Study of: Adults Total sample N: 5725  **Quartile 1:** Protein intake <0.8 g/kg aBW/d N:1579 % Female: 53.8% Median Age (IQR): 75.0 (72.0–79.0) y Race/ Ethnicity: NR Menopausal status: Postmenopausal  Obesity status: NR Mean BMI (SD): NR Income level: NR Education level:  Low: 31.1%  Medium: 36.8%  High: 32.1% Physical activity level:  Low: 36.7%  Medium: 34.1%  High: 29.1% Health status/ Comorbidities: Multimorbidity: 49.6% Medication use: NR Supplement use: NR Pregnant or lactating: NR  **Quartile 2:** Protein intake 0.8–0.99 g/kg aBW/d N:1335 % Female: 57.7% Median Age (IQR): 75.0 (72.0–79.2) y Race/ Ethnicity: NR Menopausal status: Postmenopausal  Obesity status: NR Mean BMI (SD): NR Income level: NR Education level: Low: 33.2%  Medium: 36.4% High: 30.4% Physical activity level: Low: 30.0%  Medium: 35.5% High: 34.5% Health status/ Comorbidities: Multimorbidity: 52.6% Medication use: NR Supplement use: NR Pregnant or lactating: NR  **Quartile 3:** Protein intake 1.0–1.19 g/kg aBW/d N: 1218 % Female: 53.5% Median Age (IQR): 75.0 (71.0–79.0) y Race/ Ethnicity: NR Menopausal status: Postmenopausal Obesity status: NR Mean BMI (SD): NR Income level: NR Education level: Low: 30.1%  Medium: 37.8% High: 32.1% Physical activity level: Low: 32.6%  Medium: 34.7% High: 32.7% Health status/ Comorbidities: Multimorbidity: 53.0% Medication use: NR Supplement use: NR Pregnant or lactating: NR  **Quartile 4**: Protein intake ≥1.2 g/kg aBW/d N:1593 % Female: 51.3% Median Age (IQR): 75.0 (71.0–79.0) y Race/ Ethnicity: NR Menopausal status: Postmenopausal Obesity status: NR Mean BMI (SD): NR Income level: NR Education level:  Low: 32.8%  Medium: 39.5% High: 27.6% Physical activity level: Low: 29.6%  Medium: 31.5% High: 38.9% Health status/ Comorbidities: Multimorbidity: 49.6% Medication use: NR Supplement use: NR Pregnant or lactating: NR | **Pooled analysis**  **Quartile 1:** Protein intake <0.8 g/kg aBW/d  Baseline Protein Amount Mean (SD): <0.8 g/kg aBW/d Carbohydrate Mean (SD): NR Fat Mean (SD): NR  Protein Amount at the end of the study Mean (SD): NR Carbohydrate Mean (SD): NR Fat Mean (SD): NR  **Quartile 2:** Protein intake 0.8–0.99 g/kg aBW/d  Baseline Protein Amount Mean (SD): 0.8–0.99 g/kg aBW/d Carbohydrate Mean (SD): NR Fat Mean (SD): NR  Protein Amount at the end of the study Mean (SD): NR Carbohydrate Mean (SD): NR Fat Mean (SD): NR  **Quartile 3:** Protein intake 1.0–1.19 g/kg aBW/d Baseline Protein Amount Mean (SD): 1.0–1.19 g/kg aBW/d Carbohydrate Mean (SD): NR Fat Mean (SD): NR  Protein Amount at the end of the study Mean (SD): NR Carbohydrate Mean (SD): NR Fat Mean (SD): NR  **Quartile 4:** Protein intake ≥1.2 g/kg aBW/d Baseline Protein Amount Mean (SD): ≥1.2 g/kg aBW/d Carbohydrate Mean (SD): NR Fat Mean (SD): NR  Protein Amount at the end of the study Mean (SD): NR Carbohydrate Mean (SD): NR Fat Mean (SD): NR  Protein type/ source: Mixed  Energy balance status: Eucaloric  Study duration: 8.5y | **Protein Assessment Method:** Dietary intake was assessed by an FFQ and multiple 24-h recalls. Protein intake was expressed as a categorical variable using cut points of (<0.8,0.8–0.99, 1.0–1.19, ≥1.2) based on expert recommendations for optimal protein intake on currently used RDAs for protein. | **Physical Performance -** Walking speed  Measure/Method of Assessment: Measured as the time taken to walk a distance that varied between cohorts. One cohort did not measure walking speed so the formula [6/TUG (s)]×1.62 was used to yield walking speed. |
| PMID: 35791789 **Mendonca 2023 (80)** Location/Country: USA, Canada, Netherlands, UK HDI: Very high Setting: Mixed Urban/Rural: Mixed Study design:  Pooled analysis of longitudinal observational study  Funding source: Nonprofit **Risk of bias score: Very high** | Study of: Adults Total sample N: 5584  **Quartile 1:** Protein intake <0.8 g of protein/kg aBW/d N: 1530 % Female: 53.5% Median Age (IQR): 75.0 (72.0-79.0) y Race/ Ethnicity: NR Menopausal status: Postmenopausal  Obesity status: NR Mean BMI (SD): 27.7 (4.5) kg/m^2^ Income level: NR Education level: Lower: 31.8%  Medium: 37.0% Higher: 32.0% Physical activity level: Lower: 36.5% Medium: 34.2% Higher: 29.3% Health status/ Comorbidities: Multimorbidity: 49.4% Medication use: NR Supplement use: NR Pregnant or lactating: NR  **Quartile 2:** Protein intake 0.8– <1.0g/kg aBW/d N: 1304 % Female: 53·2% Median Age (IQR): 75.0 (72.0-79.5) y Race/ Ethnicity: NR Menopausal status: Postmenopausal  Obesity status: NR Mean BMI (SD): 27.1 (4.7) kg/m^2^ Income level: NR Education level: Lower: 33.4% Medium: 36.3% Higher: 30.3%  Physical activity level: Lower: 30.2% Medium: 35.2% Higher: 34.6% Health status/ Comorbidities: Multimorbidity: 52.2% Medication use: NR Supplement use: NR Pregnant or lactating: NR  **Quartile 3**: Protein intake 1.0–<1.2 g/kg aBW/d  N: 1195 % Female: 53·3% Median Age (IQR): 75.0 (71.0-79.0) y Race/ Ethnicity: NR Menopausal status: Postmenopausal  Obesity status: NR Mean BMI (SD): 26.9 (4.7) kg/m^2^ Income level: NR Education level: Lower: 29.9% Medium: 38.1% Higher: 32.0%  Physical activity level: Lower: 32.6% Medium: 34.4% Higher: 33.0% Health status/ Comorbidities: Multimorbidity: 52.9% Medication use: NR Supplement use: NR Pregnant or lactating: NR  **Quartile 4:** Protein intake ≥1.2 g/kg aBW/d N: 1555 % Female: 50.7% Median Age (IQR): 74.0 (70.8-79.0) y Race/ Ethnicity: NR Menopausal status: Postmenopausal  Obesity status: NR Mean BMI (SD): 26.3 (5.0) kg/m^2^ Income level: NR Education level: Lower: 32.8% Medium: 39.5% Higher: 27.7% Physical activity level:  Lower: 29.4% Medium: 31.5% Higher: 39.1% Health status/ Comorbidities:  Multimorbidity: 49.4% Medication use: NR Supplement use: NR Pregnant or lactating: NR | **Quartile 1:** Protein intake <0.8 g of protein/kg aBW/d  Baseline Protein Amount Mean (SD): 44.1 (10.9) g/d; 0.6 (0.1) g/kg aBW/d; 13.6 (2.8) % of energy Carbohydrate Mean (SD): NR Fat Mean (SD): NR  Protein Amount at the end of the study Mean (SD): NR Carbohydrate Mean (SD): NR Fat Mean (SD): NR  **Quartile 2**: Protein intake 0.8– <1.0g/kg aBW/d  Baseline Protein Amount Mean (SD): 62.3 (9.1) g/d; 0.9 (0.1) g/kg aBW/d; 14.8 (3.0) % of energy Carbohydrate Mean (SD): NR Fat Mean (SD): NR  Protein Amount at the end of the study Mean (SD): NR Carbohydrate Mean (SD): NR Fat Mean (SD): NR  **Quartile 3:** Protein intake 1.0–<1.2 g/kg aBW/d  Baseline Protein Amount Mean (SD): 75.2(10.7) g/d; 1.1 (0.1) g/kg aBW/d; 15.8 (2.9) % of energy Carbohydrate Mean (SD): NR Fat Mean (SD): NR  Protein Amount at the end of the study Mean (SD): NR Carbohydrate Mean (SD): NR Fat Mean (SD): NR  **Quartile 4:** Protein intake ≥1.2 g/kg aBW/d  Baseline Protein Amount Mean (SD): 97.9 (20.4) g/d; 1.5 (0.3) g/kg aBW/d; 17.1 (3.2) % of energy Carbohydrate Mean (SD): NR Fat Mean (SD): NR  Protein Amount at the end of the study Mean (SD): NR Carbohydrate Mean (SD): NR Fat Mean (SD): NR  Protein type/ source: Mixed  Energy balance status: Eucaloric  Study duration: 8.5 y | **Protein Assessment Method:** Dietary intake was assessed by an FFQ and multiple 24-h recalls. Protein intake was expressed as a categorical variable using cut points of (<0.8,0.8–0.99, 1.0–1.19, ≥1.2) based on expert recommendations for optimal protein intake on currently used RDAs for protein. | **Muscle Strength -** Grip strength   Measure/Method of Assessment: Measured using handgrip dynamometer |
| PMID: 19419320 **Meng 2009^1^ (27)** Location/Country: Australia HDI: Very high Setting: Community dwelling Urban/ Rural: NR Study design: Prospective cohort study Funding source: Nonprofit, government  **Risk of bias score: High** | Study of: Adults Total sample N: 862   **Tertile 1**: Protein intake <66 g of protein/ d N: 287 % Female: 100% Mean Age (SD): 74.9 (2.5) y Race/ Ethnicity: 100% white origin Menopausal status: Postmenopausal  Obesity status: NR Mean BMI (SD): 26.4 (4.2) kg/m^2^ Income level: NR Education level: NR Physical activity level: 466 (median kilojoules expended per day) Health status/ Comorbidities: Participants were excluded if they had a medical condition likely to influence 5-year survival.  Medication use: Participants were excluded if they were taking bone active medications including calcium supplements, estrogen, bisphosphonates, and vitamin D.  Supplement use: Participants were excluded if they were taking bone active medications including calcium supplements, estrogen, bisphosphonates, and vitamin D. Pregnant or lactating: NR   **Tertile 2:** Protein intake 66-87 g of protein/ d N: 287 % Female: 100% Mean Age (SD): 75.0 (2.6) y  Race/ Ethnicity: 100% white origin Menopausal status: Postmenopausal Obesity status: NR Mean BMI (SD): 26.7 (4.7) kg/m^2^ Income level: NR Education level: NR Physical activity level: 530 (median kilojoules expended per day) Health status/ Comorbidities: Participants were excluded if they had a medical condition likely to influence 5-year survival. Medication use: Participants were excluded if they were taking bone active medications including calcium supplements, estrogen, bisphosphonates, and vitamin D. Supplement use: Participants were excluded if they were taking bone active medications including calcium supplements, estrogen, bisphosphonates, and vitamin D. Pregnant or lactating: NR   **Tertile 3:** Protein intake >87 g of protein/ d N: 288 % Female: 100%  Mean Age (SD): 74.7 (2.7) y Race/ Ethnicity: 100% white origin Menopausal status: Postmenopausal Obesity status: NR Mean BMI (SD): 27.3 (4.3) kg/m^2^ Income level: NR Education level: NR Physical activity level: 614 (median kilojoules expended per day) Health status/ Comorbidities: Participants were excluded if they had a medical condition likely to influence 5-year survival. Medication use: Participants were excluded if they were taking bone active medications including calcium supplements, estrogen, bisphosphonates, and vitamin D. Supplement use: Participants were excluded if they were taking bone active medications including calcium supplements, estrogen, bisphosphonates, and vitamin D. Pregnant or lactating: NR | **Tertile 1:** Protein intake <66 g of protein g/d   Baseline Protein Amount Mean (SD): 54.4 (9.1) g/d Carbohydrate Mean (SD): 146.8 (30.9) g/d Fat Mean (SD): 46.4 (13.3) g/d   Protein Amount at the end of the study Mean (SD): NR Carbohydrate Mean (SD): NR Fat Mean (SD): NR    **Tertile 2**: Protein intake 66-87 g of protein/ d   Baseline Protein Amount Mean (SD): 76.6 (6.2) g/d Carbohydrate Mean (SD): 186.4 (34.1) g/d Fat Mean (SD): 63.0 (13.3) g/d   Protein Amount at the end of the study Mean (SD): NR Carbohydrate Mean (SD): NR Fat Mean (SD): NR    **Tertile 3:** Protein intake >87 g of protein/d   Baseline Protein Amount Mean (SD): 110.9 (23.4) g/d Carbohydrate Mean (SD): 249.5 (61.9) g/d Fat Mean (SD): 85.1 (25.7) g/d   Protein Amount at the end of the study Mean (SD): NR Carbohydrate Mean (SD): NR Fat Mean (SD): NR    Protein type/source: Mixed   Energy balance status: Eucaloric    Study duration: 5 y | **Protein Assessment Method**: Participants completed a self-administered, quantitative FFQ. This FFQ has been designed to measure eating habits over the past 12-mo period and calibrated and validated according to the foods and on intake for a 12-mo period. The daily dietary intakes were derived from the questionnaire. Protein intake was assessed at baseline. | **Muscle Mass** - Lean mass    Measure/Method of Assessment: DXA (Hologic 4500A machine, Hologic, Boston, MA, USA)  **Muscle Mass** - Appendicular lean mass/ skeletal muscle mass  Measure/Method of Assessment: DXA (Hologic 4500A machine, Hologic, Boston, MA, USA) |
| PMID: 22923606 **Mulla 2013 (81)** Location/Country: UK HDI: Very high Setting: NR Urban/ Rural: NR Study design: Prospective cohort design Funding source: Nonprofit **Risk of bias score: High** | Study of: Adults  Total sample N: 1771  **Arm** 1: Men  N: 867 % Female: 0% Age: 36 Race/ Ethnicity: NR Menopausal status: NA Obesity status: NR Mean BMI (SD): NR Income level: NR Education level: NR Physical activity level: NR Health status/ Comorbidities: NR Medication use: NR Supplement use: NR Pregnant or lactating: NA  **Arm 2**: Women N: 904 % Female: 100% Age: 36 Race/ Ethnicity: NR Menopausal status: NR Obesity status: NR Mean BMI (SD): NR Income level: NR Education level: NR Physical activity level: NR Health status/ Comorbidities: NR Medication use: NR Supplement use: NR Pregnant or lactating: NR | **Arm 1:** Men  Baseline Protein Amount Mean (SD): 84 (19) g/d; 14.4 (2.4) % of energy Carbohydrate Mean (SD): 263 (75) g/d Fat Mean (SD):103 (30) g/d  Protein Amount at the end of the study Mean (SD): 88 (21) g/d; 14.6 (2.3) % of energy Carbohydrate Mean (SD): 267 (73) g/d Fat Mean (SD):  108 (32) g/d  **Arm 2:** Women  Baseline Protein Amount Mean (SD): 64 (15) g/d; 15.7 (3.6) % of energy Carbohydrate Mean (SD):  191 (64) g/d  Fat intake Mean (SD): 77 (25) g/d  Protein Amount at the end of the study  Mean (SD): 70 (16) g/d; 15.5 (3.0) % of energy Carbohydrate Mean (SD): 208 (60) g/d Fat Mean (SD): 82 (26) g/d  Protein type/source: Mixed  Energy balance status: Eucaloric  Study duration: 16 y | **Protein Assessment Method**: Food record (5-day estimated diaries) nutrient intakes for both time points were calculated based on McCance and Widdowson’s. | **Muscle Strength** - Grip strength  Measure/Method of Assessment: Measured using handgrip dynamometer   **Muscle Strength** - Chair rise test  Measure/Method of Assessment: Measured as the time taken to rise from a sitting to standing position with a straight back and legs and then sit down again. |
| PMID: 31608843 **Otsuka 2020 (82)** Location/Country: Japan HDI: Very high Setting: Community dwelling Urban/ Rural: NR Study design: Prospective cohort study Funding source: Government  **Risk of bias score: High** | Study of: Adults Total sample N: 655  **Arm 1:** Men  N: 292 % Female: 0% Mean Age (SD): 68.8 (6) y Race/ Ethnicity: NR Menopausal status: NA Obesity status: NR Mean BMI (SD): 24.3 (2.2) kg/m^2^ Income level:  Annual family income <3,500,000 Yen: 27.4% Education level:  ≤ 9 years: 27.4% Mean physical activity level (SD): 34.1 (3.80) MET x h/d Health status/ Comorbidities: Heart disease: 7.9%  Hypertension: 46.9% Dyslipidemia: 23% Diabetes: 12% Medication use: NR Supplement use: NR Pregnant or lactating: NA  **Arm 2**: Women  N: 363 % Female: 100% Mean Age (SD): 69.8 (6.5) y Race/ Ethnicity: NR Menopausal status: Postmenopausal  Obesity status: NR Mean BMI (SD): 23.5 (2.8) kg/m^2^ Income level:  Annual family income <3,500,000 Yen: 41.3% Education level:  ≤ 9 years: 32.5% Mean physical activity level (SD): 35.1 (2.6) MET x h/d Health status/ Comorbidities: Heart disease: 4.7% Hypertension: 41.3% Dyslipidemia: 30.0% Diabetes: 11.6% Medication use: NR Supplement use: NR Pregnant or lactating: NR | **Arm 1:** Men   Baseline Protein Amount Mean (SD): 86.7 (17.4) g/d Carbohydrate Mean (SD):  317.7 (60.5) g/d Fat Mean (SD): 56.6 (15.6) g/d   Protein Amount at the end of the study Mean (SD): NR  Carbohydrate Mean (SD): NR Fat Mean (SD): NR  **Arm 2**: Women  Baseline Protein Amount Mean (SD): 71·0 (13·4) g/d Carbohydrate Mean (SD):  266·3 (49·8) g/d Fat Mean (SD):  48·8 (12·4) g/d  Protein Amount at the end of the study: NR  Carbohydrate intake: NR Fat intake: NR  Protein type/ source: mixed type.  Energy balance status: Eucaloric  Study duration: 2 y | **Protein Assessment Method**: Three-day dietary record nutrient intakes were calculated according to the Standard Tables of Food Composition in Japan 2010. Within each meal sheet, i.e., the breakfast, lunch and dinner sheet, most participants recorded the time and all foods containing seasonings that they consumed. | **Muscle Mass** – Appendicular lean mass/ skeletal muscle mass  Measure/Method of Assessment: DXA (QDR-4500; Hologic, Bedford, MA, USA) |
| PMID: 26179475 **Rahi 2016 (83)** Location/Country: Canada  HDI: Very high  Setting: Community dwelling Urban/ Rural: NR Study design: Prospective cohort study Funding source: Government  **Risk of bias score: High** | Study of: Adults Total sample N: 172  **Arm 1 (Men):** Protein intake ≥1 g/kg/d N: 43 % Female: 0% Mean Age (SD): 74.9 (4.4) y Race/ Ethnicity: NR Menopausal status: NA  Obesity status: NR Mean BMI (SD): 28.4 (3.6) kg/m^2^ Income level: NR Mean education level (SD): 12.5 (5.5) y  Physical activity level: NR Health status/ Comorbidities: Participants free from heart failure greater than class II, chronic obstructive pulmonary disease requiring oxygen therapy or oral steroids, inflammatory digestive diseases, or cancer treated by radiation therapy, chemotherapy, or surgery  Medication use: NR Supplement use: NR Pregnant or lactating: NA  **Arm 2 (Men):** Protein intake <1 g/kg/d N: 63 % Female: 0% Mean Age (SD): 74.9 (4.4) y Race/ Ethnicity: NR Menopausal status: NA  Obesity status: NR Mean BMI (SD): 31.4 (4.4) kg/m^2^ Income level: NR Mean education level (SD): 11.5 (5.2) y Physical activity level: NR Health status/ Comorbidities: Participants free from heart failure greater than class II, chronic obstructive pulmonary disease requiring oxygen therapy or oral steroids, inflammatory digestive diseases, or cancer treated by radiation therapy, chemotherapy, or surgery. Medication use: NR Supplement use: NR Pregnant or lactating: NA  **Arm 3 (Women):** Protein intake ≥1 g/kg/d N: 30 % Female: 100% Mean Age (SD): 74.2 (4.1) y Race/ Ethnicity: NR Menopausal status: NR  Obesity status: NR Mean BMI (SD): 27.1 (3.2) kg/m^2^ Income level: NR Mean education level (SD): 10.2 (3.1) y Physical activity level: NR Health status/ Comorbidities: Participants free from heart failure greater than class II, chronic obstructive pulmonary disease requiring oxygen therapy or oral steroids, inflammatory digestive diseases, or cancer treated by radiation therapy, chemotherapy, or surgery. Medication use: NR Supplement use: NR Pregnant or lactating: NR  **Arm 4 (Women):** Protein intake <1 g/kg/d N: 30 % Female: 100% Mean Age (SD): 75.8 (4.3) y Race/ Ethnicity: NR Menopausal status: NR  Obesity status: NA Mean BMI (SD): 31.4 (4.6) kg/m^2^ Income level: NR Mean education level (SD): 10.2 (3.0) y Physical activity level: NR Health status/ Comorbidities: Participants free from heart failure greater than class II, chronic obstructive pulmonary disease requiring oxygen therapy or oral steroids, inflammatory digestive diseases, or cancer treated by radiation therapy, chemotherapy, or surgery. Medication use: NR Supplement use: NR Pregnant or lactating: NR | **Arm 1 (Men):** Protein intake ≥1 g/kg/d  Baseline Protein Amount Mean (SD): 97.6 (18.0) g/d; 1.24 (0.22) g/kg/d; 18.7 (3.3) % of energy Carbohydrate Mean (SD): 45.9 (7.4) % of energy Fat Mean (SD): 35.3 (6.0) % of energy  Protein Amount at the end of the study Mean (SD): NR  Carbohydrate Mean (SD): NR Fat Mean (SD): NR  **Arm 2 (Men):** Protein intake <1g/kg/d  Baseline Protein Intake Mean (SD): 70.0 (16.0) g/d; 0.78 (0.15) g/kg/d; 16.1 (3.3) % of energy Carbohydrate Mean (SD):  48.1 (7.6) % of energy Fat Mean (SD): 35.2 (6.4) % of energy  Protein Amount at the end of the study Mean (SD): NR  Carbohydrate Mean (SD): NR Fat Mean (SD): NR  **Arm 3 (Women):** Protein intake ≥1 g/kg/d  Baseline Protein Amount Mean (SD): 84.4 (20.6) g/d; 1.32 (0.31) g/kg/d; 19.2 (3.4) % of energy Carbohydrate Mean (SD): 45.8 (5.4) % of energy Fat Mean (SD): 35.3 (4.9) % of energy  Protein Amount at the end of the study Mean (SD): NR  Carbohydrate Mean (SD): NR Fat Mean (SD): NR  **Arm 4 (Women):** Protein intake <1 g/kg/d  Baseline Protein Amount Mean (SD): 58.6 (12.5) g/d; 0.78 (0.14) g/kg/d; 16.4 (3.6) % of energy Carbohydrate Mean (SD): 51.0 (6.9) % of energy Fat Mean (SD): 33.1 (6.1) % of energy  Protein Amount at the end of the study Mean (SD): NR  Carbohydrate Mean (SD): NR Fat Mean (SD): NR  Protein type/ source: Mixed  Energy balance status: Eucaloric  Study duration: 3 y | **Protein Assessment Method**: Dietary assessments were conducted by three non-consecutive 24-h dietary recalls (24-HR) on two randomly chosen weekdays and 1 weekend day. Recalls were processed using the CANDAT nutrient analysis program based on the then-current Canadian Nutrient File (CNF) database version 2001b. | **Muscle Strength** - Knee extensors   Measure/Method of Assessment: Measured using isometric contraction of the knee extensors. |
| PMID: 21054294 **Scott 2010 (84)** Location/Country: Australia HDI: Very high Setting: Community dwelling Urban/ Rural: NR Study design: Prospective cohort study Funding source: Nonprofit, government, academic  **Risk of bias score: High** | Study of: Adults  Total sample N: 1,099   **Whole Cohort** N: 1099 % Female: 50.1% Mean Age (SD: 61.9 (7.1) y Race/ Ethnicity: NR Menopausal status: NR  Obesity status: NR Mean BMI (SD): 27.5 (4.5) kg/m^2^ Income level: NR Education level: NR Mean physical activity level (SD): 8,877.0 (3,546) steps/d Health status/ Comorbidities: Excluded those with contraindication for MRI or who were institutionalized Medication use: NR Supplement use: NR Pregnant or lactating: NR | **Whole Cohort**  Baseline Protein Amount Mean (SD): 87.6 (33.7) g/d Carbohydrate Mean (SD): 213.7 (73.9) g/d Fat Mean (SD): 73.0 (29.6) g/d  Protein Amount at the end of the study  Mean (SD): 88.0 (33.7) g/d Carbohydrate Mean (SD):  205.3 (76.7) g/d Fat Mean (SD): 72.2 (30.9) g/d  Protein type/ source: Mixed  Energy balance status: Eucaloric  Study duration: 3 y | **Protein Assessment Method:** Dietary nutrient intake was assessed using The Cancer Council Victoria’s self-administered Food Frequency Ques (FFQ). The output included average daily estimates for total energy intake and for 28 dietary nutrients. Macronutrient intakes for baseline and follow-up reported. | **Muscle Mass** - Appendicular lean mass/ skeletal muscle mass  Measure/Method of Assessment: DXA (Hologic Delphi densitometer, Hologic, Waltham, MA)  **Muscle Strength** - Knee extension  Measure/Method of Assessment: Measured using isometric contraction of the knee extensors. |
[truncated: 71,742 more chars]
